# Analytical practices, use and needs of standard and reference materials in the German-speaking metabolomics community: results of an online survey

**DOI:** 10.1007/s11306-025-02360-x

**Published:** 2025-11-15

**Authors:** Carsten Jaeger, Jutta Lintelmann, Raimo Franke, Anna Artati, Alexander Cecil, Frank Broda, Frank Klawonn, Alexander Erban, Joachim Kopka, Beate Fuchs, Ulf Sommer, Meina Neumann-Schaal, Gavin O’Connor

**Affiliations:** 1 Federal Institute for Materials Research and Testing (BAM), 12489 Berlin, Germany; 2 Helmholtz Zentrum München, 85764 Neuherberg, Germany; 3 Helmholtz Centre for Infection Research, 38124 Braunschweig, Germany; 4 Leibniz Institute of Plant Biochemistry (IPB), 06120 Halle, Germany; 5 Ostfalia University of Applied Sciences, 38302 Wolfenbüttel, Germany; 6 Max Planck Institute of Molecular Plant Physiology, 14476 Potsdam, Germany; 7 Research Institute for Farm Animal Biology (FBN), 18196 Dummerstorf, Germany; 8 biocrates life sciences ag, Innsbruck, 6020 Austria; 9 Leibniz Institute DSMZ – German Collection of Microorganisms and Cell Cultures, 38124 Braunschweig, Germany; 10 Physikalisch-Technische Bundesanstalt (PTB), 38116 Braunschweig, Germany; 11 Ingolstädter Landstraße 1, 85764 Neuherberg, Germany

**Keywords:** Metabolomics, Standardization, Harmonization, Reference materials, QA/QC

## Abstract

**Introduction:**

Since the early 2000s, metabolomics has grown rapidly, becoming integral to fields like life sciences, health, and environmental research. This expansion has led to the formation of national and international societies, such as Germany’s DGMet, to tackle emerging challenges. One of DGMet’s goals is to improve measurement quality by assessing community needs for harmonization and standardization. A recent survey within the German-speaking community aimed to identify current practices and gaps in the use of chemical standards and reference materials, to guide future standardization efforts and collaborative initiatives.

**Methods:**

An online survey was conducted between June 2023 and April 2024. The survey consisted of 38 key questions and was open to research institutions from Germany, Austria, and Switzerland.

**Results:**

The survey was accessed by 68 laboratories, with 23 institutes providing complete or partial responses (34% response rate), which is comparable to rates reported in similar surveys within the metabolomics and lipidomics communities. Respondents were mainly experienced researchers from Germany, focusing mainly on health-related (“red”) metabolomics, as indicated by 78% of the respondents, followed by microbial (“grey”, 48%) and plant (“green”, 39%) metabolomics (multiple answers possible). The use of targeted methods was reported more frequently (91%) than that of non-targeted methods (78%), whereas metabolite fractions studied were equally split between polar, midpolar and lipid fractions (83% each). Human (74%), mouse (61%) and Arabidopsis (30%) were the most frequently studied organisms. Most participants used synthetic chemical standards for instrument qualification (83%), calibration (78%), and metabolite identification (74%), while matrix reference materials were mainly applied for quality control (52%) and method validation (44%). There was a strong demand for more standards, especially for metabolite identification and quantification, with cost being a major barrier, particularly for isotopically labelled standards and certified reference materials.

**Conclusions:**

Valuable insights into the use of standards and reference materials within the German-speaking metabolomics community were obtained. Moving forward, the community should address critical gaps in metabolomics standardization. To achieve this, it must share its knowledge, articulate its needs clearly, and actively engage in joint efforts with national metrology institutes and international standardization initiatives.

**Supplementary Information:**

The online version contains supplementary material available at 10.1007/s11306-025-02360-x.

## Introduction

Since the early 2000s, metabolomics has rapidly evolved and established itself across a wide range of research fields and practical applications, including life sciences, clinical research, nutrition, agriculture, biotechnology, and environmental studies (Nicholson & Wilson, 2003; Hall et al., 2002; Goodacre et al., 2004; German et al., 2004). This dynamic growth continues, marked by both groundbreaking discoveries and emerging challenges. To address the growing challenges in the field, the International Metabolomics Society was established in 2004 (Zanetti et al., 2019). Since then, numerous regional and national societies have emerged, contributing to a global network of collaboration. Examples include the Korea Metabolomics Society (KoMetS, founded 2012), the French-Speaking Metabolomics and Fluxomics Network (RFMF, 2005), the Thailand Metabolomics Society (TMS, 2017), the Latin American Metabolomic Profiling Society (LAMPS, 2014), the Nordic Metabolomics Society (2017), and the Deutsche Gesellschaft für Metabolomforschung e.V. (DGMet, 2019). A comprehensive list and further details are available at the website of the metabolomics society which these societies are affiliated with, forming a cohesive network that facilitates the effective management of challenges and initiatives at the international and national levels.

National communities play a vital role in setting and pursuing clearly defined goals tailored to their specific regional needs. Ongoing collaboration and knowledge exchange within and between national and international organizations help advance metabolomics research and support its application in areas of significant societal relevance. Within DGMet, these efforts are distributed among several working groups, one of which is the “Standards and Reference Materials” group.

This group is notable for its diverse membership, bringing together researchers from universities, Leibniz association, Max Planck and Helmholtz societies as well as representatives from governmental institutions involved in standardization, including the National Metrology Institute of Germany (PTB) and the Federal Institute for Material Research and Testing (BAM). Together, they identify, discuss, and address scientific needs related to standards and reference materials in metabolomics.

The group’s mission is to support the metabolomics community by assessing its needs for reference standards and materials. These resources are essential for developing and implementing strategies that enhance the accuracy, precision, reliability, and comparability of metabolomic measurement results. As a first step, it is crucial to assess the current state of practice within the German-speaking metabolomics community.

In this context, “standards” refer to chemically defined substances or mixtures whose chemical structure, quantity, and, where applicable, isotopic composition have been verified. In contrast, “reference materials” refer to homogeneous biological materials that are typically used for method validation and demonstration of measurement quality. Based on this framework of definitions, the logical next step was to design and distribute a survey within the German-speaking metabolomics community to evaluate the current status and specific needs regarding standards and reference materials. Specifically, the survey was aimed at answering the following questions:

1. What analytical methods and strategies are used in the (German-speaking) metabolomics community?
2. What expertise and specializations can be found within this community?
3. What standards and reference materials are currently used?
4. For what analytical purposes are standards and reference materials currently used?
5. What standards and reference materials are considered lacking, or are currently too costly?
6. Does the metabolomics community consider ring trials or proficiency testing schemes useful and what scientific goals should be pursued with such trials?

Here, the findings of this survey are presented with the goal of sparking dialogue on the future directions and priorities for standardization in the field (Jenkins et al., 2004; Fiehn et al., 2007; Dunn et al., 2013; Lippa et al., 2022; Alseekh et al., 2021). These efforts may involve initiating, supporting, or submitting proposals for national, European, or international projects, as well as organizing and coordinating ring trials across various metabolomics (sub)communities. Ideally, such initiatives will lead to - or be accompanied by - the development of universal or customized reference materials facilitating the transition of metabolomics strategies from “research only” tools to those applicable in regulated environments.

## Materials and methods

Current use cases and demands for standards and reference materials in Metabolomics analyses were discussed in a series of online meetings of the DGMet “Standards and Reference Materials” group. Following discussions, topics were summarized in the form of 38 questions, from which a web survey was created using an online survey platform (Lamapoll, Germany). Multiple choice forms were used for most questions while free text forms were used in only few cases. Answers from questions asking for personal data were saved independently to ensure data protection. The complete layout of the survey can be found in the Supplementary Material SM1.

Participating institutions and laboratories were asked to fill out the questionnaire only once to avoid multiple participation from the same institute. After completion and analysis of the survey, no redundant participations were found.

The survey was launched on 2023-06-07 and advertised through the DGMet website (www.dgmet.de) and DGMet mailing list, encouraging recipients to spread the invitation. The survey language was English. Participation was independent from DGMet membership. After closing the survey on 2024-04-30, data was exported to spreadsheet format and analyzed using the R statistical environment (R Core Team, 2024).

## Results

### Participation in the survey

**Fig. 1:**
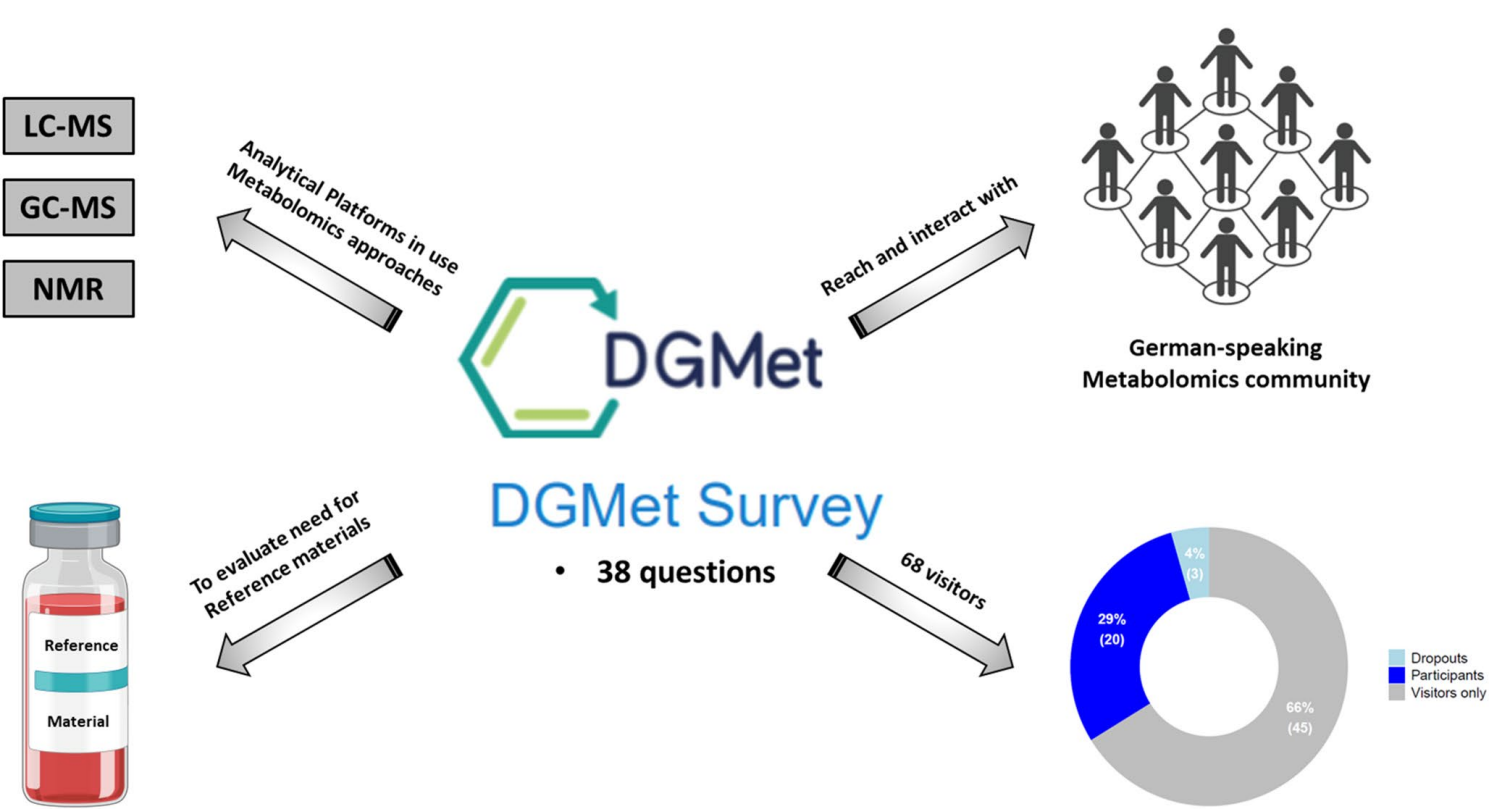
Focus and goals of the DGMet survey. The online survey consisted of 38 questions addressing the use and need of standards and reference materials in metabolomics. The number of visitors and active participants is indicated

A total number of 68 institutions/scientists (laboratories) visited the survey, 20 of them chose to complete the survey and another three to answer at least partially (Fig. 1). Unless otherwise noted, percent calculations are always based on these 23 participants (100%).

Even though the questionnaire was distributed in all German-speaking countries, most participants came from Germany (21 responses, or 91%), only two came from Austria. For reasons of data protection, responses on the participants (institution, position) were recorded separately, and information on this was voluntary. Those questions were answered by 17 participants. Nine (53%) of those respondents were from major German non-university research organizations (Leibniz Association, Helmholtz Association and Max Planck Society), four (23%) were from universities, three (18%) were from government institutions and one (6%) was from industry.

Most respondents had a group leader, project leader or senior scientist role (17, 74%). Only one postdoc was among the respondents, and no doctoral or other students or technicians were recorded. Overall, a highly experienced group of principal investigators (PIs) and decision makers completed the questionnaire.

### Metabolomics strategies and metabolite fractions

**Fig. 2:**
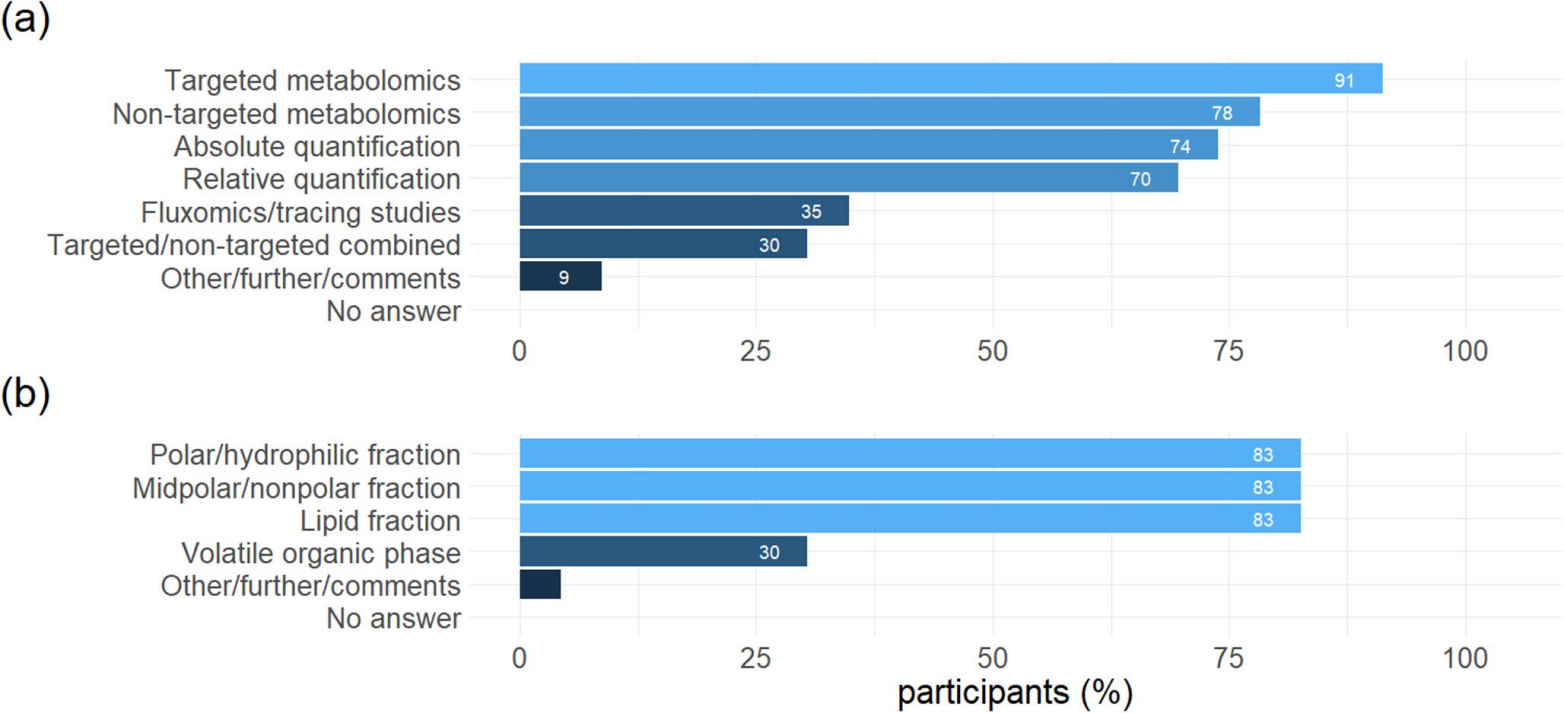
Metabolomics strategies used (a), and metabolite fractions investigated (b) by the respondents. Multiple answers possible

Most of the 23 respondents used targeted metabolomics (21, 91%), closely followed by non-targeted metabolomics (18, 78%; Fig. 2a). Correspondingly, absolute quantification was performed by 17 (74%) respondents while relative quantification was performed by 16 (70%) respondents. Fluxomics was only used by roughly a third of the respondents (8, 35%). Regarding the metabolite fractions that were investigated by the respondents, there was a tie between polar, midpolar and lipid fractions, all of which were investigated by 19 (83%) respondents (Fig. 2b). The volatile organic (VOC) phase on the other hand was only investigated by seven (30%) respondents.

### Research areas and organisms investigated

**Fig. 3:**
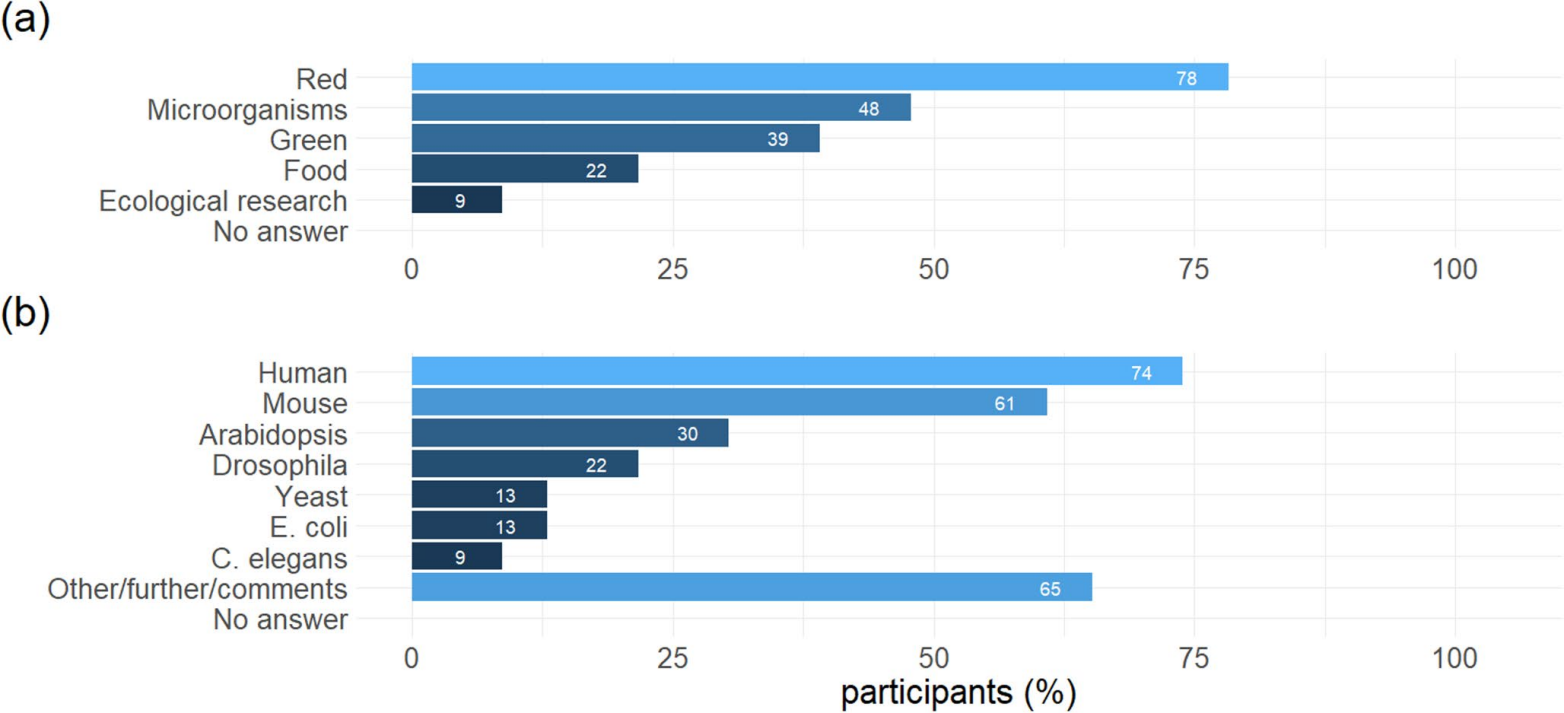
Research areas in which the respondents are active (a) and organisms they study (b). Multiple answers possible. 100% = 23 participants

Most respondents (18, 78%) focused on the “red” research area, investigating human health, diseases, and clinical applications for instance (Fig. 3). Eleven (48%) investigated microbial metabolism working with microorganisms. Nine (39%) respondents found themselves in the “green” category of metabolomics research, referring to research related to plants, algae, green biotechnology. Only a very small fraction chose the categories “food” (5, 22%) and ecological research (2, 9%) respectively. In terms of the organisms investigated, human (17, 74%) and mouse (14, 61%) samples were studied most frequently, followed by Arabidopsis (7, 30%), Drosophila (5, 22%) and Yeast as well as *E. coli* (each 3, 13%). Many participants indicated further organisms in the free-text field, including zebra fish, rat, farm animals, cultured mammalian and bacterial cells as well as different crop species, highlighting the diversity of research questions to which metabolomics is applied in the participating labs.

When participants were asked for a self-assessment of their expertise and specialization, answers ranged from analytical expertise such as development of new analytical strategies to expertise in particular research questions like “biomarker” and “organ cross communication” studies. The full list of answers is provided in the Supplementary Material SM2.

### Analytical platforms and strategies used

**Fig. 4:**
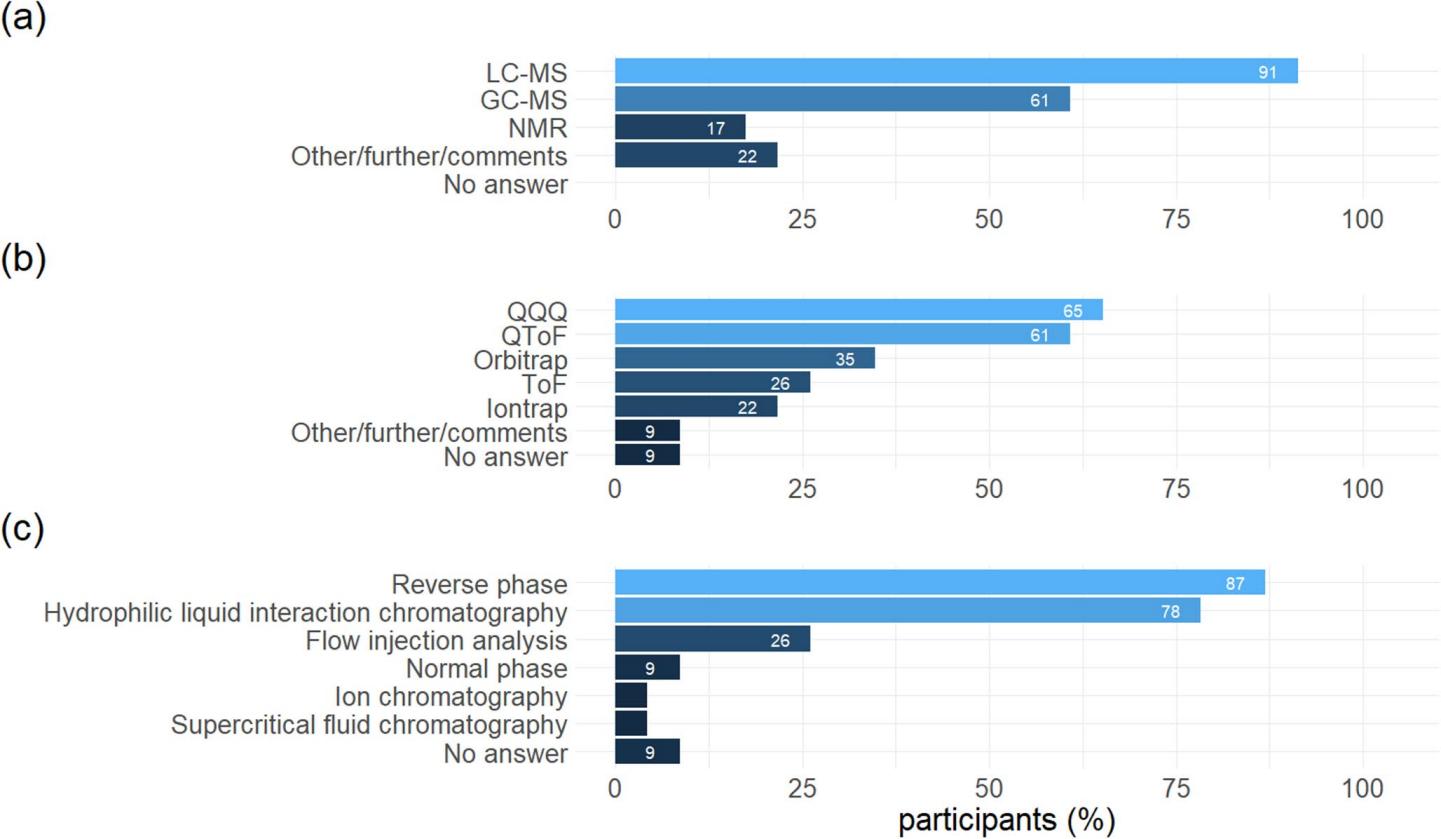
Analytical platforms (a), LC-MS instrument types (b) and chromatography systems (c) used by the respondents. Multiple answers possible. For GC-MS instrument types and chromatography systems, see Supplementary Material SM2, p. 23

A clear majority of the respondents chose LC-MS as their preferred analytical platform (21, 91%), followed by GC-MS (14, 61%; Fig. 4). NMR and other techniques were only used by few respondents with four (17%) NMR users and five (22%) users of other methods. Among the 21 LC-MS users, the majority (15, 71%) used triple quadrupole mass spectrometers (QQQ), closely followed by quadrupole time-of-flight devices (QToF; 14, 67%). Orbitrap (8, 35%), time-of-flight (ToF; 6, 29%) and ion trap (5, 24%) mass spectrometers were used less frequently. The most used chromatographic separation mode for LC-MS was reverse phase (RP; 20, 95%), followed by hydrophilic liquid interaction chromatography (HILIC; 18, 86%). Direct infusion without prior chromatographic separation (flow injection analysis) was applied in six laboratories (29%). Normal phase, ion exchange and supercritical fluid chromatography were each used in less than 10% of the laboratories. Typical LC-MS ion sources were mentioned, including electrospray ionization (ESI; 19, 90%) and atmospheric pressure chemical ionization (APCI; 9, 43%).

Among the 14 GC-MS users, single-quad (9, 64%) and QQQ (7, 50%) instruments were most popular, predominantly used in conjunction with nonpolar (e.g. DB-5; 12, 85%) and midpolar (e.g. DB-17; 6, 43%) capillary columns (see SM2). Electron ionization (EI) was the predominant ion source; only one GC-MS user used chemical ionization or negative chemical ionization (CI, NCI).

Ten (43%) participants indicated that they also used ion mobility as an analytical technique. Among them, Differential Mobility Separation (DMS) and Trapped Ion Mobility Spectrometry (TIMS) appeared to be more popular than other approaches, including Drift Time Ion Mobility Spectrometry (DTIMS), Travelling Wave Ion Mobility (TWIMS) and cyclic IMS.

### Use of commercial metabolomics kits

How widespread is the use of commercially available kits in the community? This was the focus of questions 16 to 18 that were answered by eight (35%) participants. Six of them (75%) employed kits from biocrates life sciences ag while one (13%) laboratory used the Lipidyzer TM platform and another one (13%) specified the application of an NMR method provided by Bruker. It is remarkable that seven of eight (88%) users of commercial kits indicated that they modified the technical application protocol in terms of mass spectrometric and chromatographic parameters as well as in terms of sample preparation steps.

### Use of chemical standards and reference materials

**Fig. 5:**
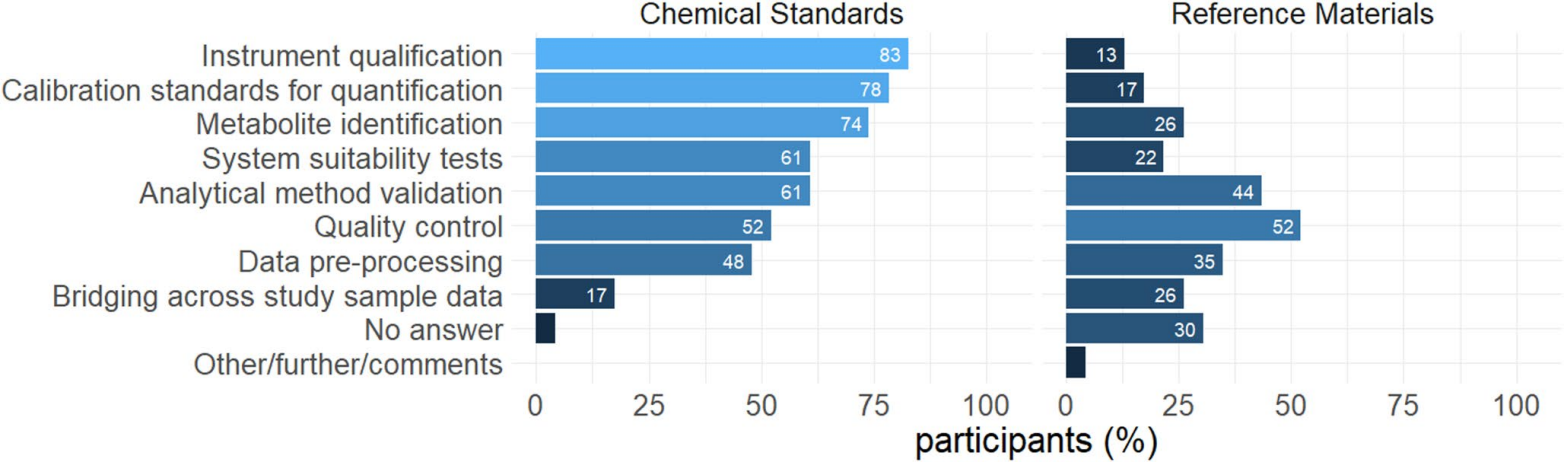
Use of synthetic chemical standards (left) and matrix reference materials (right) in metabolomics protocols. Participants were asked to select all options that applied to their lab’s use of chemical standards and reference materials, respectively. Multiple answers possible, 100% = 23 participants

In metabolomics, chemical standards and matrix reference materials can both serve different analytical purposes such as metabolite identification, instrument calibration, or quality control (Mosley et al., 2024; Mandal et al., 2025). When asked about the use of synthetic chemical standards in their labs, most participants (19, 83%) responded, “Instrument qualification”, followed by “Calibration standards for quantification” (18, 78%) and “Metabolite identification” (17, 74%; Fig. 5). “System suitability tests” and “Analytical method validation” were also indicated frequently (both 61%). When asked the same question about matrix reference materials, the order of answers differed noticeably. Here, “Quality control purposes” and “Analytical method validation” were mentioned most often (52% and 44%, respectively) while “Calibration standards for quantification” (4, 17%) and “Instrument qualification” (3, 13%) were indicated less frequently. Several respondents (7, 30%) did not specify any purpose in the context of reference materials, suggesting that reference materials are not routinely used at all in some labs.

Participants were also asked if they only used commercially available standard mixtures, or if they also produced and used their own mixtures. Nineteen (83%) participants answered that they made use of commercial products, while a similar number (17, 74%) said they used in-house prepared mixtures. Among the commercial products, standard mixtures from “Avanti polar lipids, Inc.” (12, 52%), “Merck/Sigma” (11, 48%) and “Cambridge Isotope Laboratories, Inc.” (9, 39%) appeared to be the most popular options. Notably, a large majority of participants did not use custom chemical synthesis (15, 65%) while only five (22%) participants used either commercial synthesis services or a synthesis facility at their institute. Concerning the origin of reference materials, a similar picture emerged. 12 (52%) participants used commercial materials (either certified or non-certified), while the same percentage indicated that they produced their own reference materials. Among the commercial materials, “NIST SRM 1950” (metabolites in human plasma) was mentioned by seven (30%) respondents. In agreement with a previous answer, seven (30%) laboratories neither used commercial or own reference materials. Non-certified reference materials (“research-grade testing materials”) did not appear to be widely used; only three participants mentioned the use of e.g. “Iso-1 (Isotopic Solutions, Vienna)” or an unspecified “reference plasma”.

### Need for chemical standards and reference materials

**Fig. 6:**
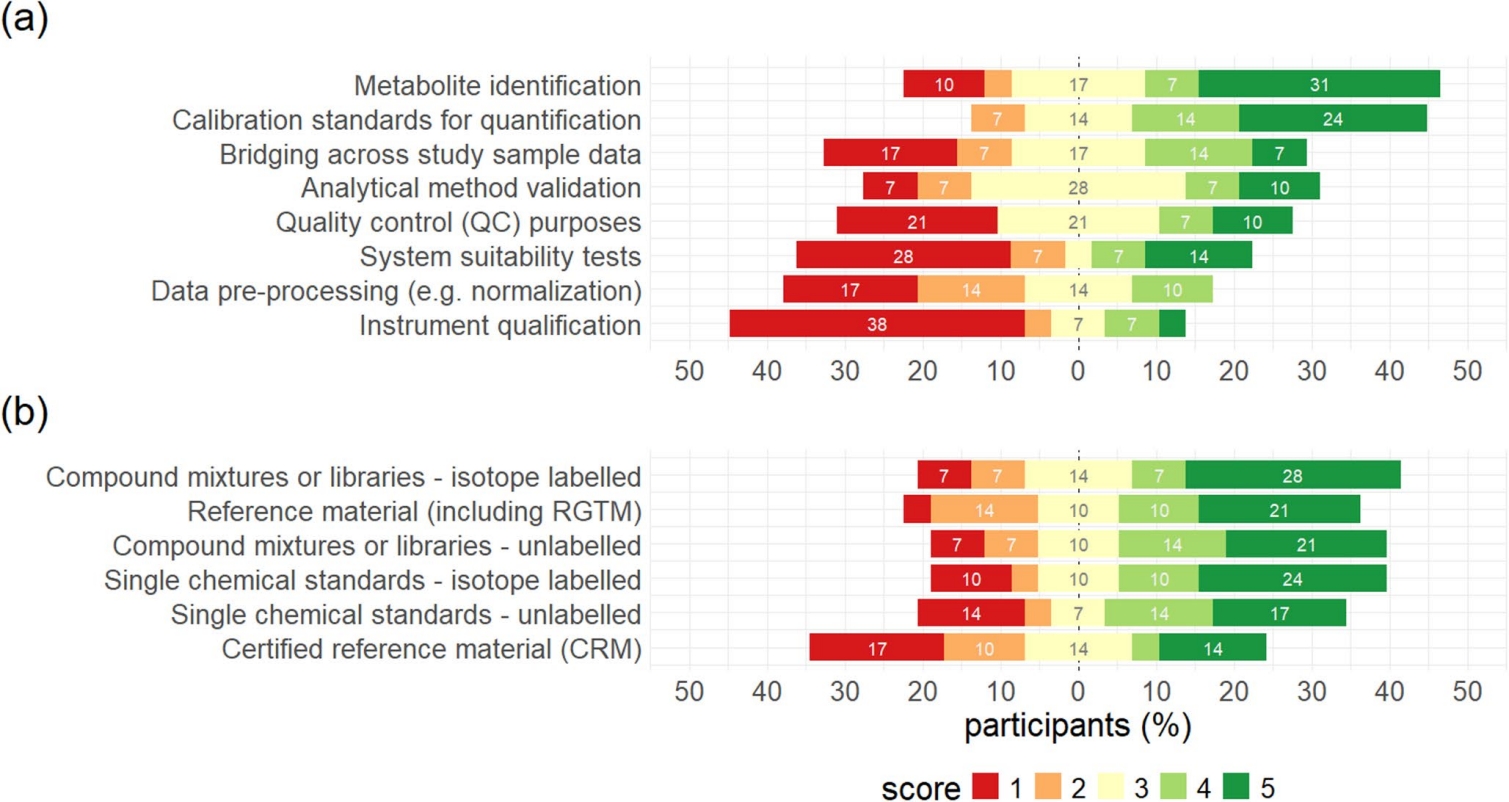
Perceived lack of chemical standards and reference materials. Participants were asked if they missed any standards, standard mixtures or reference materials that could support their metabolomics tools (a). Needs should be rated from 1 (no need) to 5 (strong need), multiple answers possible. In addition, they were asked to indicate the type of new standards or reference materials that are needed (b). RGTM, research grade testing material. 100% = 23 participants, % missing from 100: no answer

Participants were next asked if they considered the current offer of standards, standard mixtures and reference materials sufficient, or if they felt there was any lack of such materials for certain applications. The largest lack was seen in the context of “Metabolite Identification”, where a clear majority of participants expressed a need for a wider offer (Fig. 6). A similar assessment was obtained regarding “Calibration standards for quantification”. By contrast, less need was seen for additional products in the areas “System suitability tests”, “Data pre-processing” and “Instrument qualification”. Regarding the type of standard material needed, the only clear trend observed was that all types of standard materials were equally lacking. The offer of synthetic standards, both single substances and mixtures in either labelled or unlabeled form, as well as uncertified reference materials (i.e. research grade testing materials, RGTM) were all considered similarly incomplete by most participants. Notably, certified reference materials (CRMs) were an exception, where a slight majority saw no need for new products.

Materials that participants explicitly desired included bacterial metabolites and lipids, bacterial secondary metabolites, a primary metabolite library, a fecal standard, and gut metabolites (microbiome) as well as a pooled organ-specific plant material. A depleted/stripped plasma for method validation and blanks was also mentioned by one participant.

Seventeen laboratories answered the question if costs are prohibiting them from using chemical standards or reference materials in their metabolomic workflow. This seemed to be especially true for labelled compound mixtures or libraries, that were highest rated with higher scores (82% score 4 and 5, cost is problematic) considering the cost issue, followed by isotope labelled single chemical standards (81% score 4 and 5) and certified reference materials (58% score 4 and 5).

### Interest in ring trials and workshops

An interest in metabolomics ring trials was expressed by 12 (52%) participants while eight (35%) participants were undecided. Four (17%) participants said they had no interest in taking part in a ring trial. Among the interested labs, evaluating, comparing, and improving analytical methods was the main motivation for a potential participation in a ring trial.

## Discussion

In times of increasingly sophisticated technical instrumentation for rapid generation of metabolomics data, the need for improving the comparability of results via standardization and harmonization approaches hence increasing the reliability and thus quality of this data must evolve even faster. Trustworthy data forms the basis for all further steps. The creation, dissemination and evaluation of surveys is a useful tool in this regard and has already proven successful to meet specific questions.

This study represents the first targeted survey of the German-speaking metabolomics community with a focus on the use and perceived needs of standards and reference materials. In contrast to previous international surveys (Dunn et al., 2017; Evans et al., 2020), which primarily assessed general QA/QC practices in metabolomics, our study delved specifically into analytical standardization materials and their application across national research community. This narrower but deeper scope provides a critical complementary perspective to the broader efforts of the international metabolomics community. It also forms a basis by which standardization bodies and institutes can be informed on the needs and wishes of this community.

The response rate for the survey (23/68, 34%) is in line with those reported in previous community surveys. Evans et al. (2020) reported 23 laboratories completing their metabolomics questionnaire; Bowden et al. (2018) achieved 39% participation (125/322 laboratories) in a lipidomics survey; Dunn et al. (2017) collected 97 responses across 84 institutions for the Metabolomics Society Data Quality Task Group questionnaire; and more recently, Fisher et al. (2025) reported 61 participants in the Best Practices for Nontargeted Analysis (BP4NTA) survey that was distributed to a broad international audience including e.g. researchers from environmental analysis. Taken together, these examples show that participation numbers in metabolomics-related surveys are typically modest, yet sufficient to capture representative community perspectives. In our case, the predominance of group leaders and senior scientists among respondents further strengthens the validity of the insights obtained. The results reflect both alignment and divergence with global trends. The predominant use of LC-MS platforms and a strong biomedical focus is consistent with the global metabolomics landscape. However, compared to the international survey by Evans et al. (2020), which included a broader range of institutions and a more balanced representation of QA/QC practices, our sample was heavily weighted toward senior scientists in academic and non-profit research institutes. This likely influenced the high awareness of standard reference materials, such as NIST SRM 1950, the first metabolomics-specific matrix material released in 2013 (Phinney et al., 2013). It probably also affected the pronounced interest in improved materials.

NIST SRM 1950 emerged as the most frequently cited reference material in both our study and prior international surveys (Lippa et al., 2022). While its utility as a widely studied and quantified plasma reference is undisputed - with more than 300 metabolites analyzed in a recent study (Mandal et al., 2025) - participants in our survey expressed several limitations: high cost, limited relevance beyond human plasma, long-term storage concerns, and uncertainty regarding its representativeness for European populations. These concerns are consistent with recent evaluations and highlight the need for community-driven development of supplemental or alternative materials tailored to specific organisms, matrices, or study goals.

Respondents also emphasized the need for simple, modular standard mixtures for routine tasks such as ionization profiling or method validation. This underscores a dual requirement within the community: on one hand, accessible and purpose-built chemical standards for everyday QA/QC routines, and on the other, high-quality biological matrices that enable benchmarking across platforms and laboratories. These priorities echo recommendations from the mQACC workshop series (Mandal et al., 2020; Mosley et al., 2024) and mQACC guidelines (Kirwan et al., 2022), which have called for a tiered strategy in deploying reference materials depending on study scope and instrumentation, and transparently reporting their use within studies.

Compared to the studies by Dunn et al. (2017) and Evans et al. (2020) that focused on QA/QC practices, our findings indicate a lower adoption rate of formal SOPs, proficiency testing, and external audits. However, the motivation to close these gaps is strong: more than half of the respondents expressed interest in participating in ring trials. At the same time, a significant portion remained undecided, a pattern also observed in earlier surveys, suggesting that hesitancy may stem from limited resources, unclear incentives, or the absence of coordination mechanisms. Addressing these obstacles will be crucial to strengthen collaboration and harmonize analytical practices.

The survey also revealed a strong desire for materials that support emerging needs, including microbiome research, fecal and gut metabolite standards, plant organ-specific matrices, and depleted plasma pools. These proposals extend beyond current offerings and point toward a more dynamic and application-specific standardization strategy. This perspective aligns closely with current efforts to anchor metabolomics infrastructures in the FAIR principles (Wilkinson et al., 2016), which emphasize data findability, accessibility, interoperability, and reusability. While FAIR compliance was not an explicit topic in the survey, the responses clearly suggest a growing awareness of its relevance and an openness to practical implementation.

Several limitations of our study must be acknowledged. The number of participants was modest, which may affect the generalizability of the findings. In addition, the respondent pool was largely limited to Germany, despite efforts to reach Austria and Switzerland as well. The results also reflect a strong bias toward LC-MS-based workflows and biomedical applications, leaving other technologies and research fields underrepresented. Nonetheless, the professional profile of respondents, predominantly senior scientists, and group leaders, suggests a high degree of subject-matter expertise. As with other international surveys based on voluntary participation, such as that of Evans et al. (2020), our results should be seen as a focused snapshot that highlights key trends and concrete needs rather than a fully comprehensive census. The survey was advertised via a single formal platform (DGMet website and mailing list), which is primarily accessed by metabolomics experts. This likely explains the high proportion of PIs and senior scientists among respondents. While this ensured targeted, high-quality responses from decision makers, it may have limited input from students or technical staff, who might have provided more diverse perspectives on day-to-day practices.

Despite these constraints, the findings of our study offer a valuable basis for follow-up activities. The community has articulated clear needs for better materials, more coordinated approaches, and more transparent workflows. National metrology institutes such as PTB and BAM, already involved in this working group, can serve as important partners in bridging the gap between scientific communities and the formal measurement infrastructure. Their expertise could help accelerate the development and dissemination of certified and fit-for-purpose materials, and support broader adoption through shared validation data, training, and certification schemes.

In conclusion, the German-speaking metabolomics community shows both awareness and ambition regarding the role of standards and reference materials. While international efforts such as mQACC provide a valuable umbrella, regional initiatives remain essential to address context-specific requirements and to operationalize best practices in ways that reflect local research priorities and infrastructures. Our study lays the groundwork for such efforts by identifying concrete needs, potential collaborators, and directions for future development. Moving forward, coordinated national and international initiatives will be needed to ensure that reference materials and QA/QC strategies truly meet the diverse and evolving needs of metabolomics research.

## Conclusions

This first targeted survey of the German-speaking metabolomics community highlights both the strong awareness of standardization practices but also the limited availability of suitable reference materials. In particular, there is a clear need for more tailored and affordable solutions, ranging from matrix-specific reference materials to simple standard mixtures for daily QA/QC routines. The community is motivated to engage in ring trials and collaborative efforts. To move forward, coordinated national and international initiatives are needed, ones that reflect real-world workflows, foster FAIR data practices, and support the development of next-generation reference materials that truly meet the needs of diverse metabolomics applications.

Our survey has provided valuable insights into the use of standards and reference materials within the German-speaking metabolomics community. While certain limitations exist, the study highlights the strong foundation of expertise in clinical applications and the importance of continued collaboration to advance the field.

Moving forward, the community must take action and address critical gaps in metabolomics standardization. In natural products research, there is an urgent need for better and more comprehensive reference compounds and spectral libraries to improve compound identification. Additionally, the development of new reference materials, similar to the SRM 1950 plasma but better reflecting the European population, is essential.

A call for action is needed to establish common standards and reference materials in cooperation with national metrology institutes (NMIs). To achieve this, the community must share its knowledge, articulate its needs clearly, and actively engage in joint efforts to drive standardization forward. Strengthening collaboration and resource sharing will be key to ensuring a more robust and harmonized approach to metabolomics research.

## Supplementary Information

Below is the link to the electronic supplementary material.

Supplementary Material 1

Supplementary Material 2

## Data Availability

The survey results are made available in the Supplementary Information.
