# Supplementary material for: Analytical practices, use and needs of standard and reference materials in the German-speaking metabolomics community: results of an online survey
Source: Metabolomics. 2025 Nov 15;21(6):171. doi: 10.1007/s11306-025-02360-x (PMC12619739; doi:10.1007/s11306-025-02360-x)
Supplement: Supplementary file 1 — Supplementary Material 1 [file 11306_2025_2360_MOESM1_ESM.pdf]

DGMet Survey

Survey for the DGMet members and Metabolomics groups in German speaking countries

The DGMet working group for "Reference Materials and Standards" has set itself the goal of supporting the metabolomics community in its work by evaluating your requirements for reference standards and materials. The aims of such materials and standards are to allow strategies to be developed and implemented that will enable the community to generate more reliable and comparable measurement results. In this first step, it is important to map the status quo within the (German) metabolomics community. By "reference materials and standards" we mean chemically or biologically defined substances or mixtures of substances whose identity, quantity and, where applicable, isotopic composition on has been authenticated.

This questionnaire is therefore intended to answer the following questions:

1. What methods, expertise and fields of activity can be found within the (German) metabolomics community?
2. How are standards and reference materials used currently?
3. What can or should the scientific objective of future standardization initiatives look like?
4. For special expertise and techniques, we ask you to use the free text fields.
5. Furthermore, we would like to determine whether the DGMet members consider ring trials or proficiency testing schemes useful and if you would be interested to participate in them, as well as to develop the scientific objectives of such a trial.
6. What scientific problems could form the basis of joint grant applications?

It would be helpful if you could answer as many questions as possible. If this is not possible in individual cases, please go to the next question. Please consider *only the current state* of your lab activities.

Some questions cannot be answered by ticking. Free text fields are available here.

Our aim is to establish what support is necessary for a thriving metabolomics community. For this we need your input. Therefore, we hope we can rely on your kind support.

We assure you that your data will be treated with the utmost confidentiality. Your personal data (optional contact information) will be stored separately from your answers.

When starting the survey, please take about **15 min**.
Saving of intermediate results is possible by clicking the link at the bottom of each page.

DGMet Survey: Metabolomics Portfolio

Question 1 - Country

Please indicate the country where your lab is located.

- ◯ Germany
- ◯ Austria
- ◯ Switzerland
- ◯ Other

Question 2 - Metabolomics Strategies

Which Metabolomics strategy / approaches do you use in your lab?

- [ ] Targeted metabolomics
- [ ] Non-targeted metabolomics
- [ ] Combination of targeted and non-targeted approaches in one method
- [ ] Relative quantification
- [ ] Absolute quantification
- [ ] Fluxomics and/or tracing studies
- [ ] Other/further/comments

Question 3 - Metabolite Fractions

Which fractions of the metabolome do you analyze in your lab?

- ☐ Polar/hydrophilic fraction
- ☐ Midpolar/nonpolar fraction
- ☐ Lipid fraction (Lipidomics)
- ☐ Volatile organic phase (Volatilome, exhaled breath, etc.)
- ☐ Other/further/comments

Question 4 - Research Area(s)

Please indicate the research area(s) you are working in.

- [ ] Red (clinical, cohort, human, animal models, toxicology, nutrition, ...)
- [ ] Green (plants, algae, green biotechnology)
- [ ] Microorganisms
- [ ] Food
- [ ] Ecological research
- [ ] Other/further/comments

Question 5 - Organisms investigated in your lab

What organisms/samples are studied in your lab?

- [ ] Human
- [ ] Mouse
- [ ] *C. elegans*
- [ ] *Drosophila*
- [ ] Yeast
- [ ] *E. coli*
- [ ] *Arabidopsis*
- [ ] Other/further/comments

DGMet Survey: Analytical Platforms

Question 6 - Analytical platform(s) used in your lab

What analytical platform(s) do you use for metabolomics?

- [ ] LC-MS (LC-HRMS, LC-MS/MS, ...)
- [ ] GC-MS (GC-HRMS, GC-MS/MS, ...)
- [ ] NMR
- [ ] Other/further/comments

Question 7 - **Type(s) of Mass Spectrometer (LC-MS)**

Please specifiy the type(s) of MS detector used in your LC-MS setup(s).

- [ ] QQQ
- [ ] ToF
- [ ] QToF
- [ ] Orbitrap
- [ ] Iontrap
- [ ] Other/further/comments

Question 8 - Type(s) of Chromatography (LC-MS)

Please specify the type(s) of chromatography used in your LC-MS setup(s).

- [ ] Reverse phase (C18, C8 and similar)
- [ ] Hydrophilic liquid interaction chromatography (HILIC)
- [ ] Normal phase (NP)
- [ ] Ion chromatography (IC)
- [ ] Supercritical fluid chromatography (SFC)
- [ ] FIA (no chromatography)
- [ ] Other/further/comments

Question 9 - Type(s) of Mass Spectrometer (GC-MS)

Please specifiy the type(s) of MS detector used in your GC-MS setup(s).

- [ ] SingleQ
- [ ] QQQ
- [ ] ToF
- [ ] QToF
- [ ] Orbitrap
- [ ] Iontrap
- [ ] Other/further/comments

Question 10 - Type(s) of Chromatography (GC-MS)

Please specify the column type(s) or configuration(s) used in your GC-MS setup(s).

- ☐ nonpolar (e.g. HP-5, DB-5, HP-1)
- ☐ midpolar (e.g. DB-17, DB-35, VF-624)
- ☐ polar (e.g. DB-WAX , Carbowax)
- ☐ GCxGC
- ☐ Other/further/comments

Question 11 - Ion source(s) used in your lab

What type(s) of ion sources are used in your lab? This includes both LC-MS, GC-MS or standalone MS setups.

- [ ] ESI
- [ ] APCI
- [ ] EI
- [ ] APPI
- [ ] (N)CI
- [ ] DESI
- [ ] DART
- [ ] MALDI
- [ ] Other/further/comments

Question 12 - Ion Mobility Usage

Do you use ion mobility? If yes, please select the technique(s) you use.

- [ ] DMS
- [ ] TIMS
- [ ] Drift tube (DTIMS)
- [ ] TWIMS/cyclic IMS
- [ ] Other/further/comments

Question 13 - Tell us which analysis strategies you use and give a score from 1 to 5 for how frequently you use it

Rate from **1 = rarely** to **5 = very often**

LC-MS		(1 - 5)	○ No answer
LC-UV or other detectors		(1 - 5)	○ No answer
Direct infusion-MS		(1 - 5)	○ No answer
GC-MS		(1 - 5)	○ No answer
GC-FID or other detectors		(1 - 5)	○ No answer
Ion mobility-MS		(1 - 5)	○ No answer
NMR		(1 - 5)	○ No answer
Raman spectroscopy		(1 - 5)	○ No answer

DGMet Survey: Expertise

Question 14 - Expertise and Specialization

Optionally, please tell us about your main areas of expertise:

(1)

(2)

(3)

Question 15 - In-house Methods

If you have published any of your own analytical methods, please indicate the most relevant ones.
Please give the **DOI** if available, otherwise a free text citation.

(1)

(2)

(3)

DGMet Survey: Use of Commercial Kits

Question 16 - Commercial Kits (covering the complete workflow)

Several commercial kits are available for targeted metabolomics. If you use any of the following kits in your lab, please indicate so. The list below is not meant to be comprehensive. If you use other commercial kit options, we do encourage you to specify those in the free text field.

- ☐ AbsoluteIDQ(R) p180
- ☐ AbsoluteIDQ(R) p400 HR
- ☐ MxP(R) Quant 500
- ☐ MxP(R) Quant HR XpressTM
- ☐ AbsoluteIDQ(R) Stero 17
- ☐ AbsoluteIDQ(R) Bile acids
- ☐ LipidyzerTM platform
- ☐ Other/further/comments

Question 17 - **Do you use a modified kit protocol?**

- [ ] Yes
- [ ] No

Question 18 - Please let us know, what part of the protocol you modified

- [ ] Mass spectrometric parameters (e.g. collision energy, gases)
- [ ] Chromatographic parameters (e.g. injection volume, flow rate)
- [ ] Sample preparation
- [ ] Other/further/comments

DGMet Survey: Use of Chemical Standards

Question 19 - Use of chemical standards

Chemical standards are often used for quality control, normalization, calibration, quantification etc. If you use chemical standards or standard mixtures in your metabolomics protocol, please indicate their intended purpose(s).

Please note: this question refers only to the use of **chemical standards** and **standard mixtures**. The use of **matrix reference materials** will be addressed in the next section.

- ☐ Instrument qualification *(e.g. mass calibration with tune mix)*
- ☐ System suitability tests *(e.g. test mix of standards)*
- ☐ Analytical method validation
- ☐ Quality control (QC) purposes
- ☐ Calibration standards for quantification
- ☐ Metabolite identification
- ☐ Bridging across study sample data
- ☐ Data pre-processing *(e.g. normalization)*
- ☐ Other/further/comments

Question 20 - Chemical standard mixtures used in your lab

For the standard mixtures, please indicate if you use commercial products or inhouse prepared mixtures.

- ☐ Commercially available compound mixtures or libraries
- ☐ In-house prepared compound mixtures

Question 21 - Commercially available standard mixtures

Please select the manufacturers of the commercial standard mixture products used in your lab. The list is not meant to be comprehensive. We do encourage you to use the free text field in case you order from other manufacturers.

- ☐ Cambridge Isotope Laboratories, Inc.
- ☐ IROA Technologies
- ☐ Merck/Sigma
- ☐ biocrates life science ag
- ☐ MetaSci
- ☐ Avanti polar lipids, Inc.
- ☐ Sciex
- ☐ Metrological institutes *(e.g. JRC, NIST)*
- ☐ Other/further/comments

Question 22 - Commercially available compound mixtures

Please let us know the standard mixture product(s) you are using. Please also indicate if these are native or isotopically labelled compound mixtures. If you use a nonlabelled and labelled variant of the same product, please tick both.

Product name<br>*e.g. Bacterial metabolite library*	Isotope labelling (^{13}C, ^{15}N, ...)	
	Native	Labelled
...	☐	☐
...	☐	☐
...	☐	☐
...	☐	☐
...	☐	☐
...	☐	☐

Question 23 - In-house prepared compound mixtures

Please let us know some details about your standard mixture(s) prepared in-house. Please also indicate if these are native or isotopically labelled compound mixtures. If you use a non-labelled and labelled variant of the same mixture, please tick both.

Mixture<br>*e.g. Amino acid mix, ESI test mix*	Isotope labelling (^{13}C, ^{15}N, ...)	
	Native	Labelled
...	☐	☐
...	☐	☐
...	☐	☐
...	☐	☐
...	☐	☐
...	☐	☐

Question 24 - **Do you use custom synthesis of standard compounds?**

Please indicate if you use chemicals synthesized on demand that may not be available as a standard product.

- [ ] Yes, I have compounds synthesized by a commercial lab.
- [ ] Yes, I use the synthesis facility at my institute.
- [ ] No, I don't use custom chemical synthesis.

DGMet Survey: Use of Reference Materials

Question 25 - Use of matrix reference materials

Similar to chemical standards, **matrix reference materials** such as human blood plasma can be used for different purposes along the metabolomics workflow. If you use matrix reference materials for any of the below purposes, please indicate so. We do encourage you to use the free text field if you use matrix reference materials for different purposes.

- ☐ Instrument qualification *(e.g. mass calibration)*
- ☐ System suitability tests
- ☐ Quality control (QC) purposes
- ☐ Analytical method validation
- ☐ Calibration standards for quantification
- ☐ Metabolite identification
- ☐ Bridging across study sample data
- ☐ Data pre-processing *(e.g. normalization)*
- ☐ Other/further/comments

Question 26 - Reference materials used in your lab

Please share with us which type of reference material you are using. *Certified reference materials (CRM)* are reference materials, accompanied by documentation issued by an

authoritative body and providing one or more specified property values with associated uncertainties and traceabilities, using valid procedures. *Non-certified reference materials* are materials, sufficiently homogeneous and stable with reference to specified properties, which have been established to be fit for their intended use in measurement. These often lack the measurement traceability and/or uncertainties associated with CRMs but are typically less costly and sufficient for many testing purposes. *In-house prepared reference materials* including custom testing materials, such as large batches of cell culture or human plasma used for method testing, quality assurance etc.

- [ ] Certified reference material (CRM, e.g. NIST SRM 1950)
- [ ] Non-certified reference material (including "research grade testing materi-als")
- [ ] In-house prepared reference material

Question 27 - Certified reference materials used in your lab

Which *certified* reference materials do you use?

- [ ] NIST SRM1950 human plasma pool
- [ ] Other/further/comments

Question 28 - Certified reference materials used in your lab – applications

What do you use it for specifically? This information will be important to CRM producers. If CRMs are predominantly used for non-certified purposes producers can simply generate large batches of common materials, making it cheaper and globally available.

- [ ] Quantification of measurands that CRM has been certified for
- [ ] Quantification of measurands that CRM has not been certified for
- [ ] General method quality control
- [ ] Comparability within or between labs
- [ ] Other/further

Question 29 - Non-certified reference materials (including "research grade testing materials")

We would be glad about some details on the *non-certified* reference materials (including "research grade testing materials") you are using.

Product		Isotope labelling (^{13}C, ^{15}N, ...)	
Name	Species/Matrix	Native	Labelled
		☐	☐
		☐	☐
		☐	☐
		☐	☐
		☐	☐

Question 30 - In-house prepared reference materials

We would be glad about some details on the *in-house prepared* reference materials you are using.

Material		Isotope labelling (^{13}C, ^{15}N, ...)	
Name	Species/Matrix	Native	Labelled
		☐	☐
		☐	☐
		☐	☐
		☐	☐
		☐	☐

DGMet

DGMet Survey: Need for New Standards and Reference Materials

Question 31 - **Are you missing standards, standard mixtures or reference materials that could support your metabolomics tools?**

Please indicate the application area(s) where you see the need for **new** standards and/or reference materials.

Rate from **1 = no need** to **5 = strong need.**

Instrument qualification	O	O	O	O	O
System suitability tests	O	O	O	O	O
Quality control (QC) purposes	O	O	O	O	O
Analytical method validation	O	O	O	O	O
Calibration standards for quantification	O	O	O	O	O
Metabolite identification	O	O	O	O	O
Bridging across study sample data	O	O	O	O	O
Data pre-processing (e.g. normalization)	O	O	O	O	O

Question 32 - **Please indicate the type of new standards or reference materials that you feel are needed.**

Rate from **1 = no need** to **5 = strong need.**

Single chemical standards - unlabelled	O	O	O	O	O
Single chemical standards - isotope labelled	O	O	O	O	O
Compound mixtures or libraries - unlabelled	O	O	O	O	O
Compound mixtures or libraries - isotope labelled	O	O	O	O	O
Reference material (including research grade testing materials)	O	O	O	O	O
Certified reference material (CRM)	O	O	O	O	O

Question 33 - Is cost prohibiting you from using chemical standards or reference materials in your metabolomics workflow?

Please rate if high prices are prohibiting you from using more chemical standards and reference materials, from **1 = cost is no issue** to **5 = cost is problematic.**

Single chemical standards - unlabelled	O	O	O	O	O
Single chemical standards - isotope labelled	O	O	O	O	O
Compound mixtures or libraries - unlabelled	O	O	O	O	O
Compound mixtures or libraries - isotope labelled	O	O	O	O	O
Reference material (including research grade testing materials)	O	O	O	O	O
Certified reference material (CRM)	O	O	O	O	O

Question 34 - **Are there particular standard mixtures or reference materials that you are interested in?**

Please let us know some specific materials that you would wish to include in your metabolomics workflow.

(1) ☐

(2) ☐

(3) ☐

(4) ☐

(5) ☐

DGMet Survey: DGMet Ring Trial

Question 35 - Ring Trial Participation

DGMet is planning to perform ring trials aiming at improved harmonization of metabolomics methods as well harmonization of metabolomics data.

Are you interested in taking part in such ring trials?

- [ ] Yes
- [ ] No
- [ ] Don't know yet

Question 36 – Motivation

Please share your main scientific goals and motivation to participate in a ring trial.

(1)

(2)

(3)

Question 37 - Did you participate in ring trials in the past?

○ Yes ○ No

DGMet Survey: Results Workshop

Question 38 - Workshop

Would you be interested in taking part in a workshop in which the results and future strategies that derive from this survey are discussed?

◯ Yes ◯ No

Question 39 - Contact Information

Please leave your contact information if you want to keep in touch with DGMet. Your data will be stored separately from your answers.

Name of your research group / department: ☐

Institute or company: ☐

Name of the contact person: ☐

Email address: ☐

Question 40 - **Position of the Contact Person**

Please make your choice.

- ◯ Group leader
- ◯ Project leader
- ◯ (Senior) Scientist
- ◯ Technician
- ◯ Postdoc
- ◯ Doctoral Student
- ◯ Student
- ◯ Other

Thank you for your participation!

Please check our homepage for news:
DGMet – Deutsche Gesellschaft für Metabolom Forschung
