# Supplementary material for: Analytical practices, use and needs of standard and reference materials in the German-speaking metabolomics community: results of an online survey
Source: Metabolomics. 2025 Nov 15;21(6):171. doi: 10.1007/s11306-025-02360-x (PMC12619739; doi:10.1007/s11306-025-02360-x)

Question 1 - Country

Status: July 18, 2024, 13:37, Survey: "DGMet-Survey"

Number of participants evaluated: 29 (all participants)

Status data

of 29 participants	Number	Percent
Question seen	29	100%
Question answered	23	79.31%
Question not answered	6	20.69%

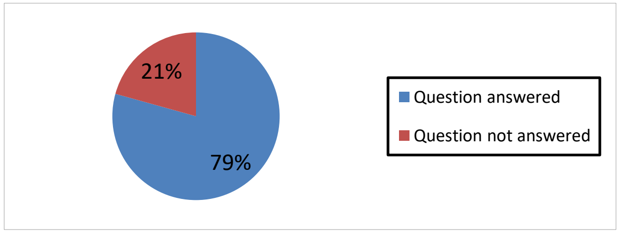

Results

Options	Variable	Code	Number	Frequency
Germany	V188	1	21	91.30%
Austria	V188	2	1	4.35%
Switzerland	V188	3	0	0%
Other	V188	4	1	4.35%
**Total**			23 Answers	23 Participants

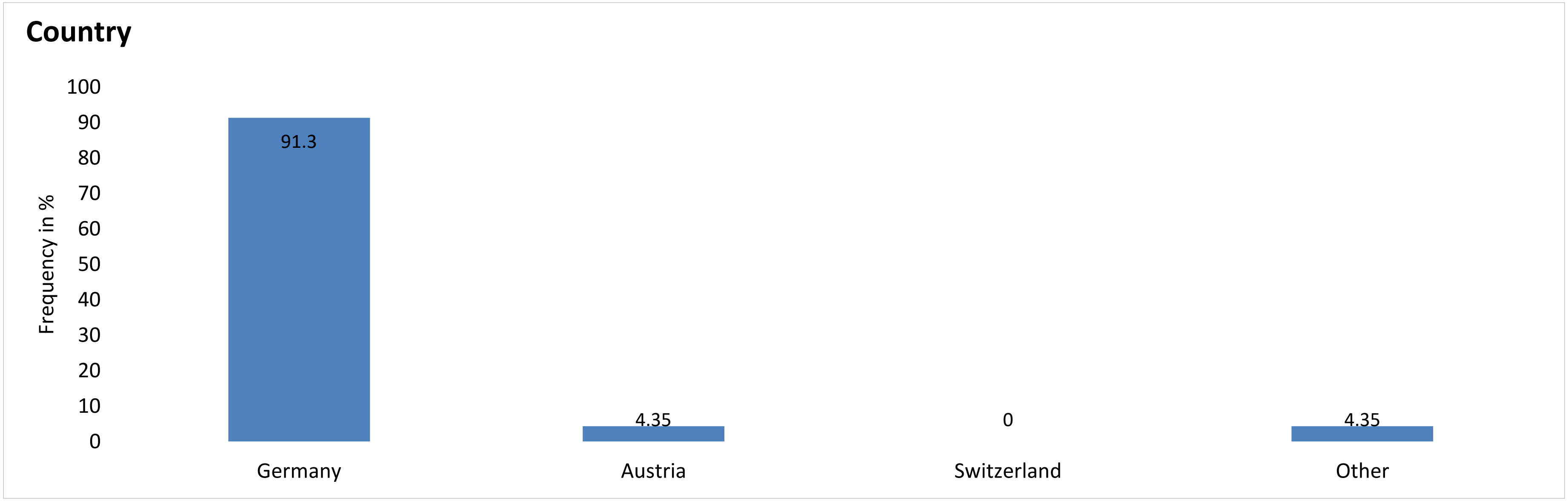

Question 2 - Metabolomics Strategies

Status: July 18, 2024, 13:37, Survey: "DGMet-Survey"

Number of participants evaluated: 29 (all participants)

Status data

of 29 participants	Number	Percent
Question seen	29	100%
Question answered	23	79.31%
Question not answered	6	20.69%

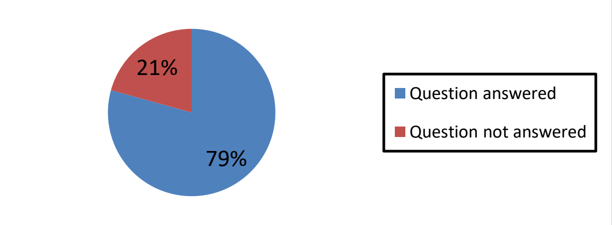

Results

Options	Variable	Code	Number	Frequency by participant	Frequency by answers
Targeted metabolomics	V1	1	21	91.30%	23.60%
Non-targeted metabolomics	V2	1	18	78.26%	20.22%
Combination of targeted and non-targeted approaches in one method	V3	1	7	30.43%	7.87%
Relative quantification	V4	1	16	69.57%	17.98%
Absolute quantification	V5	1	17	73.91%	19.10%
Fluxomics and/or tracing studies	V6	1	8	34.78%	8.99%
Other/further/comments	V63	1	2	8.70%	2.25%
**Total**			89 Answers	23 Participants	

Question 2 - Metabolomics Strategies

Status: July 18, 2024, 13:37, Survey: "DGMet-Survey"

Number of participants evaluated: 29 (all participants)

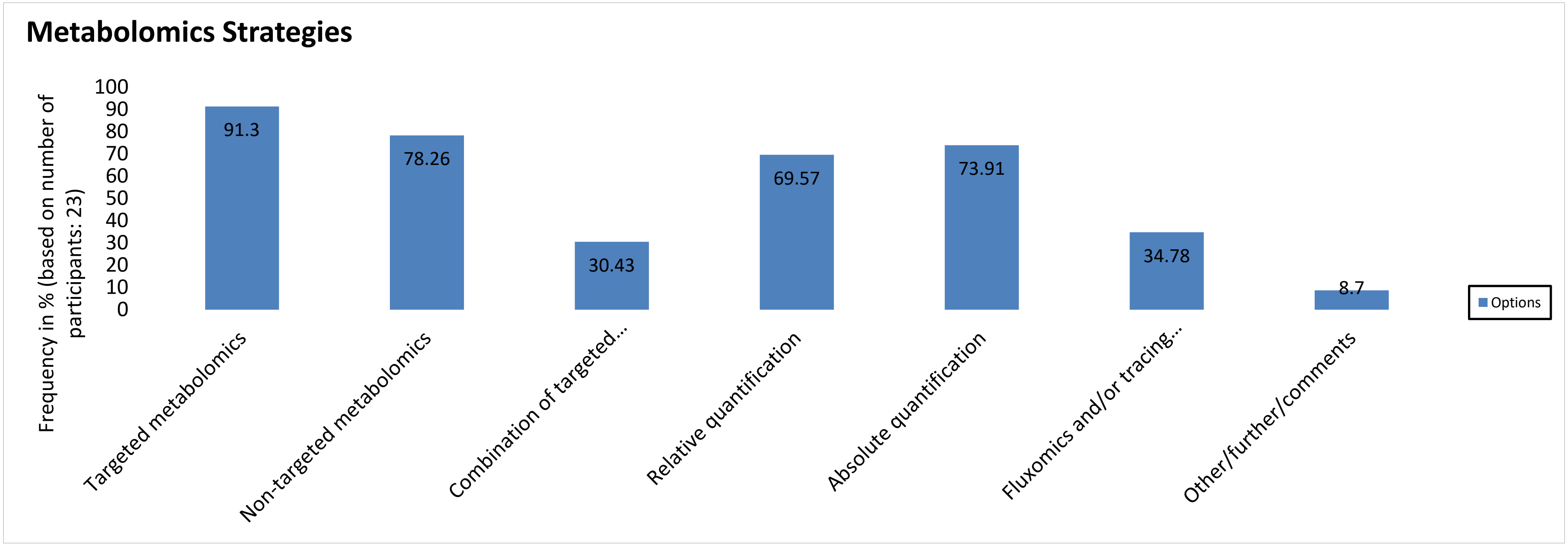

Question 2 - Metabolomics Strategies

Status: July 18, 2024, 13:37, Survey: "DGMet-Survey"

Number of participants evaluated: 29 (all participants)

Detailed results for entry field of Other/further/comments

Number Answers	2	Number unique	2

Value/Answer	Number	Frequency
Imaging	1	50%
special focus on lipids	1	50%
**Total**	**2**	**100%**

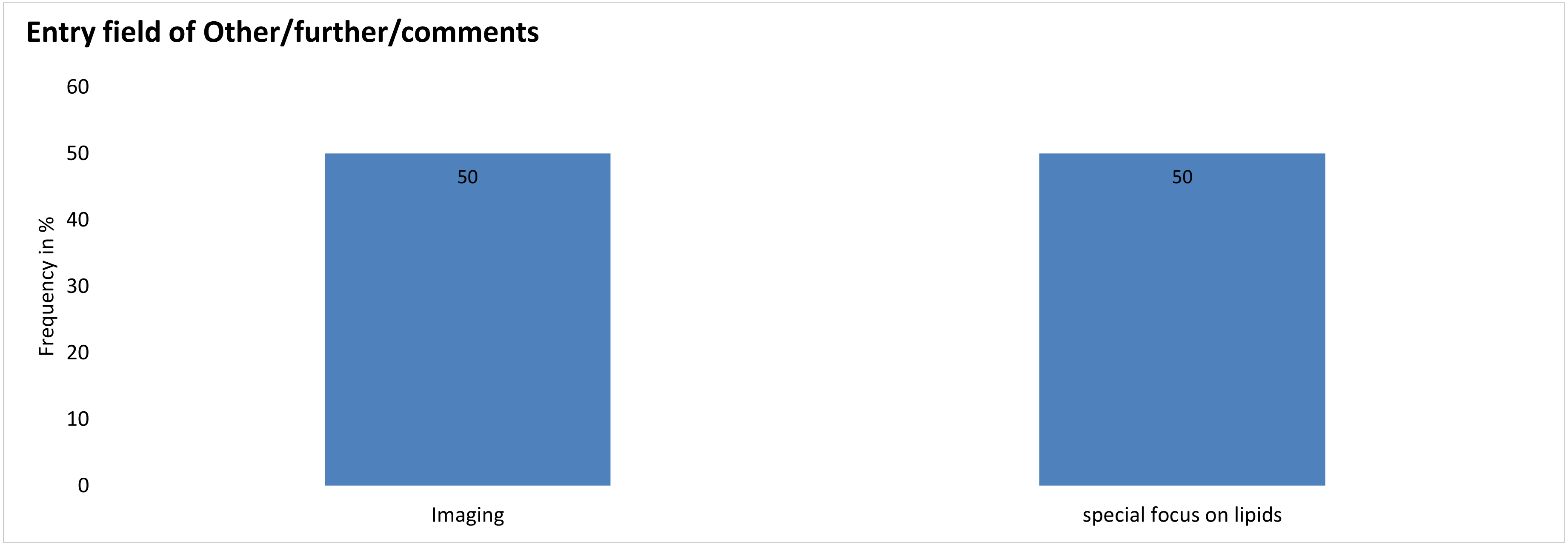

Question 3 - Metabolite Fractions

Status: July 18, 2024, 13:37, Survey: "DGMet-Survey"

Number of participants evaluated: 29 (all participants)

Status data

of 29 participants	Number	Percent
Question seen	29	100%
Question answered	23	79.31%
Question not answered	6	20.69%

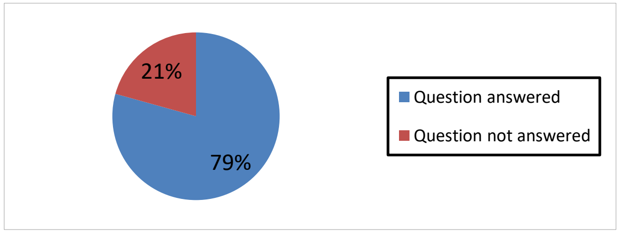

Results

Options	Variable	Code	Number	Frequency by participant	Frequency by answers
Polar/hydrophilic fraction	V7	1	19	82.61%	29.23%
Midpolar/nonpolar fraction	V8	1	19	82.61%	29.23%
Lipid fraction (Lipidomics)	V9	1	19	82.61%	29.23%
Volatile organic phase (Volatilome, exhaled breath, etc.)	V10	1	7	30.43%	10.77%
Other/further/comments	V11	1	1	4.35%	1.54%
**Total**			65 Answers	23 Participants	

Question 3 - Metabolite Fractions

Status: July 18, 2024, 13:37, Survey: "DGMet-Survey"

Number of participants evaluated: 29 (all participants)

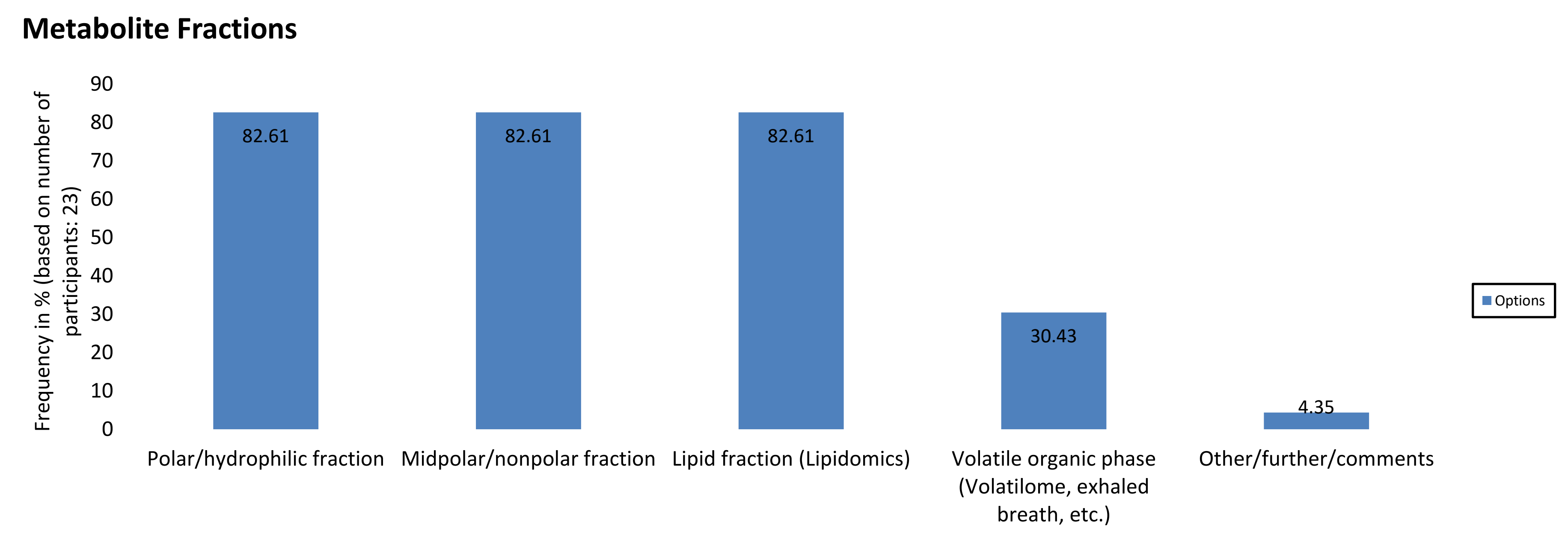

Question 3 - Metabolite Fractions

Status: July 18, 2024, 13:37, Survey: "DGMet-Survey"

Number of participants evaluated: 29 (all participants)

Detailed results for entry field of Other/further/comments

Number Answers	1	Number unique	1

Value/Answer	Number	Frequency
NMR spectroscopy	1	100%
**Total**	**1**	**100%**

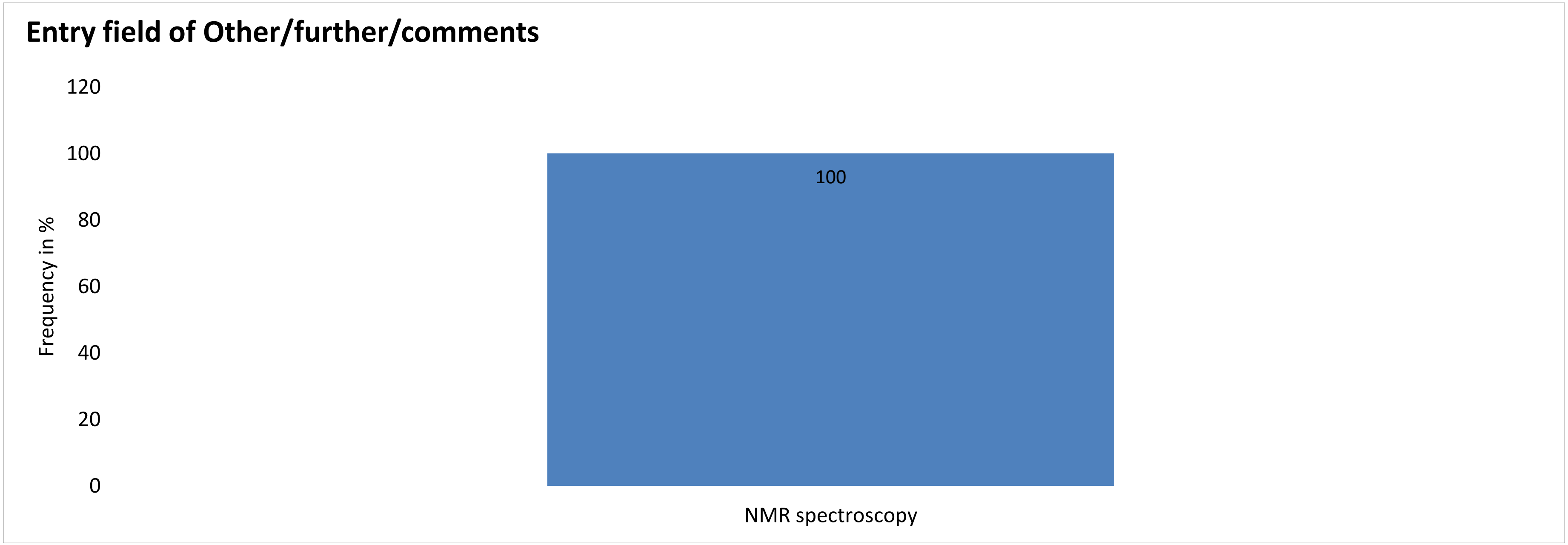

Question 4 - Research Area(s)

Status: July 18, 2024, 13:37, Survey: "DGMet-Survey"

Number of participants evaluated: 29 (all participants)

Status data

of 29 participants	Number	Percent
Question seen	29	100%
Question answered	23	79.31%
Question not answered	6	20.69%

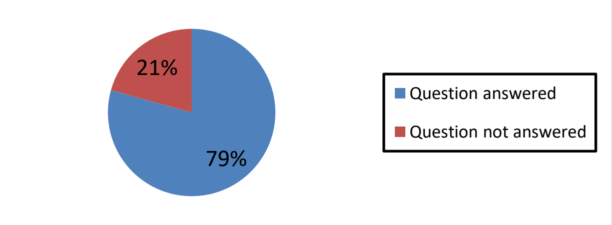

Results

Options	Variable	Code	Number	Frequency by participant	Frequency by answers
Red (clinical, cohort, human, animal models, toxicology, nutrition, ...)	V50	1	18	78.26%	40%
Green (plants, algae, green biotechnology)	V51	1	9	39.13%	20%
Microorganisms	V52	1	11	47.83%	24.44%
Food	V53	1	5	21.74%	11.11%
Ecological research	V54	1	2	8.70%	4.44%
Other/further/comments	V56	1	0	0%	0%
**Total**			45 Answers	23 Participants	

Question 4 - Research Area(s)

Status: July 18, 2024, 13:37, Survey: "DGMet-Survey"

Number of participants evaluated: 29 (all participants)

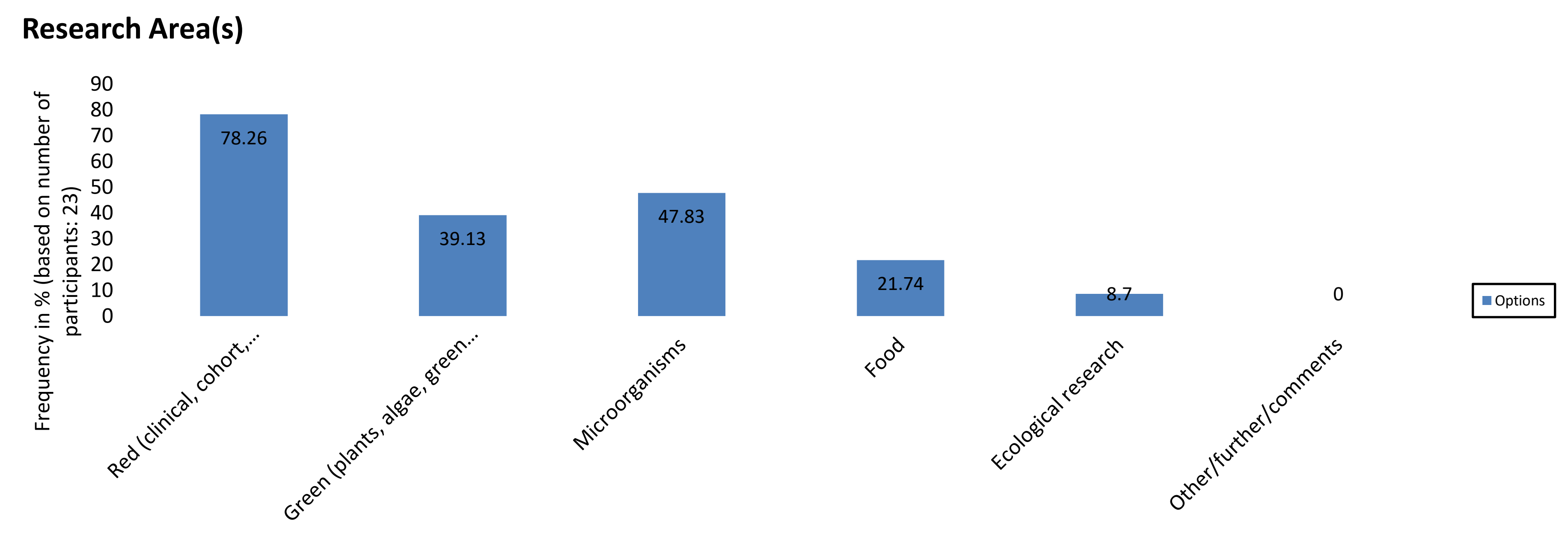

Question 4 - Research Area(s)

Status: July 18, 2024, 13:37, Survey: "DGMet-Survey"

Number of participants evaluated: 29 (all participants)

Detailed results for entry field of Other/further/comments

Number Answers	0	Number unique	0

Value/Answer	Number	Frequency

Question 5 - Organisms investigated in your lab

Status: July 18, 2024, 13:37, Survey: "DGMet-Survey"

Number of participants evaluated: 29 (all participants)

Status data

of 29 participants	Number	Percent
Question seen	29	100%
Question answered	23	79.31%
Question not answered	6	20.69%

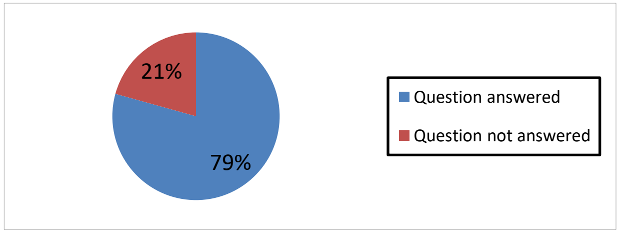

Results

Options	Variable	Code	Number	Frequency by participant	Frequency by answers
Human	V55	1	17	73.91%	25.76%
Mouse	V57	1	14	60.87%	21.21%
C. elegans	V58	1	2	8.70%	3.03%
Drosophila	V59	1	5	21.74%	7.58%
Yeast	V60	1	3	13.04%	4.55%
E. coli	V61	1	3	13.04%	4.55%
Arabidopsis	V62	1	7	30.43%	10.61%
Other/further/comments	V148	1	15	65.22%	22.73%
**Total**			66 Answers	23 Participants	

Question 5 - Organisms investigated in your lab

Status: July 18, 2024, 13:37, Survey: "DGMet-Survey"

Number of participants evaluated: 29 (all participants)

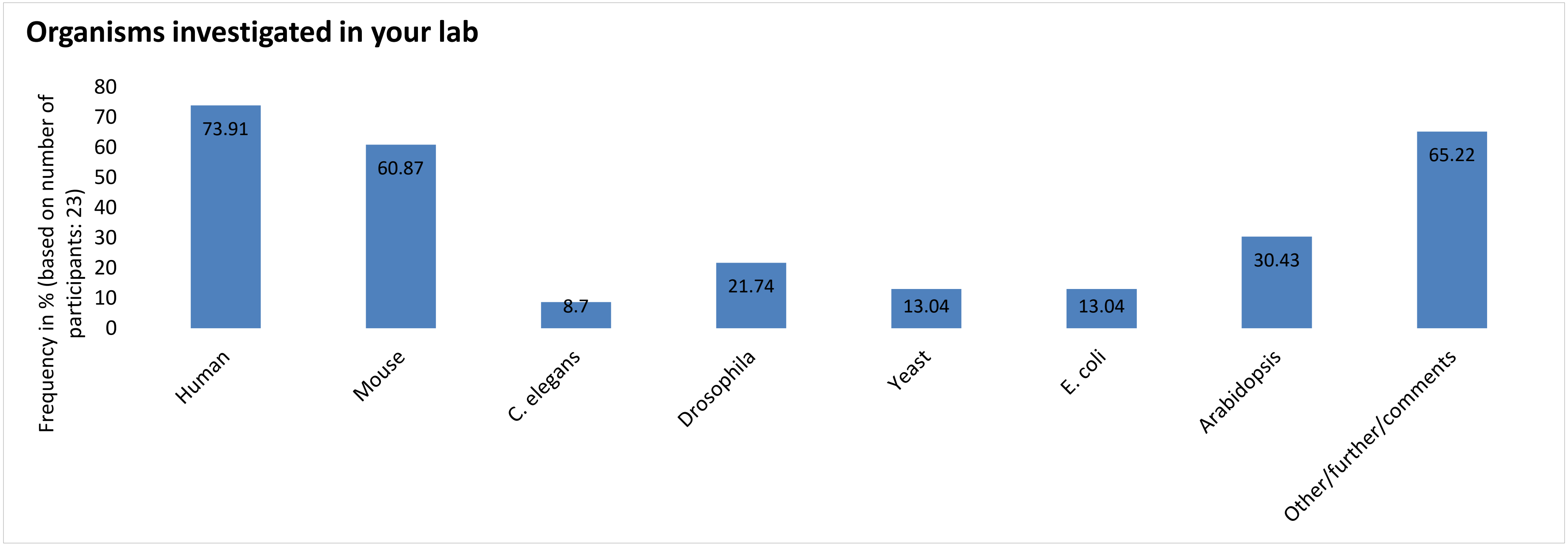

Question 5 - Organisms investigated in your lab

Status: July 18, 2024, 13:37, Survey: "DGMet-Survey"

Number of participants evaluated: 29 (all participants)

Detailed results for entry field of Other/further/comments

Number Answers	15	Number unique	13

Value/Answer	Number	Frequency
Zebrafish	1	6.67%
Our Samples are roughly 90% Mouse, 9% Human, 1% Drosophila	1	6.67%
Different type of plants, Human microbiome, Mussels and other animals	1	6.67%
algae, other animals including invertebrates, different cell types	1	6.67%
C. difficile<br>S. solfataricus<br>P. aeruginosa<br>P. inhibens	1	6.67%
rat, cell cultures	1	6.67%
Zebrafish, rat, mammalian cell lines, Lemna, field crops and vegetables, microorganisms other than Saccharomyces	1	6.67%
many bacteria	1	6.67%
Brassica species<br>corn<br>wheat<br>ficus<br>oak<br>tomato<br>moringa peregrina<br>sugar cane<br>cassava<br>conyzia canadenisis<br>plantago lanceolata<br>white clover	1	6.67%

Question 5 - Organisms investigated in your lab

Status: July 18, 2024, 13:37, Survey: "DGMet-Survey"

Number of participants evaluated: 29 (all participants)

farm animals	2	13.33%
P. aeruginosa	1	6.67%
other plants and crops<br>cyanobacteria<br>eukaryotic algae	1	6.67%
trees; agriculture plants (e.g. wheat, tomato, rice etc.); microbes (bacteria isolates, fungi).	1	6.67%
**Total**	**14**	**93.33%**

Created with LamaPoll | https://www.lamapoll.de

Question 5 - Organisms investigated in your lab

Status: July 18, 2024, 13:37, Survey: "DGMet-Survey"

Number of participants evaluated: 29 (all participants)

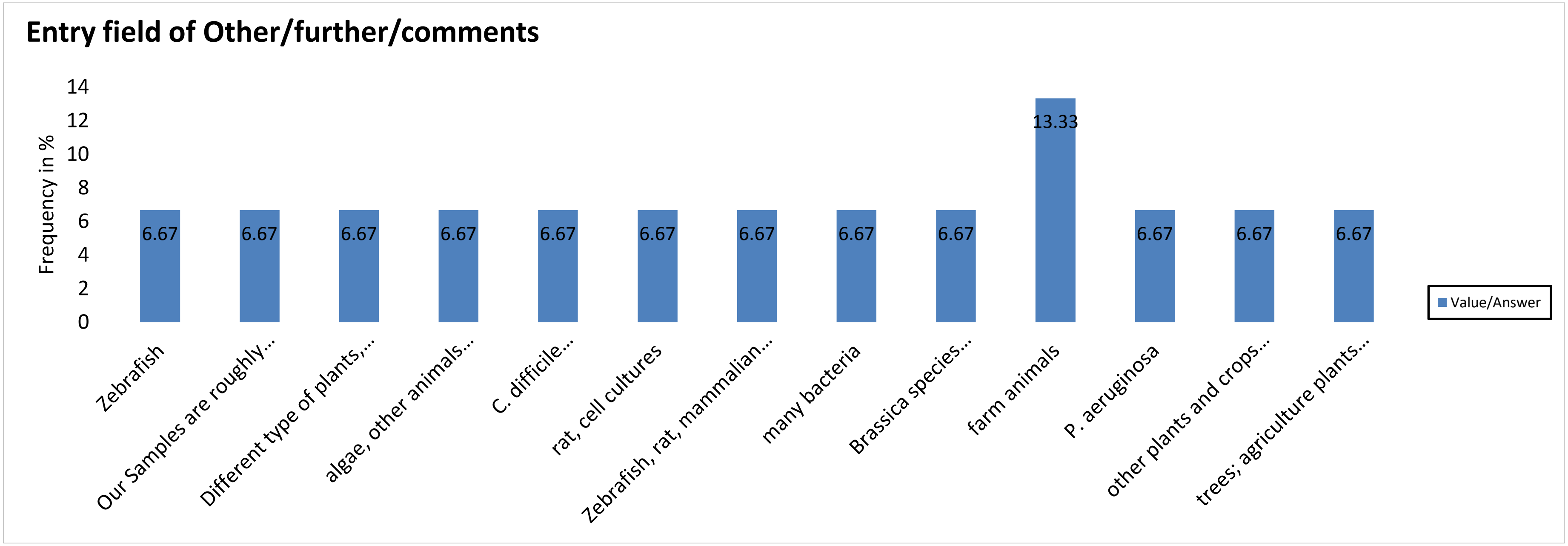

Question 6 - Analytical platform(s) used in your lab

Status: July 18, 2024, 13:37, Survey: "DGMet-Survey"

Number of participants evaluated: 29 (all participants)

Status data

of 29 participants	Number	Percent
Question seen	23	79.31%
Question answered	23	79.31%
Question not answered	6	20.69%

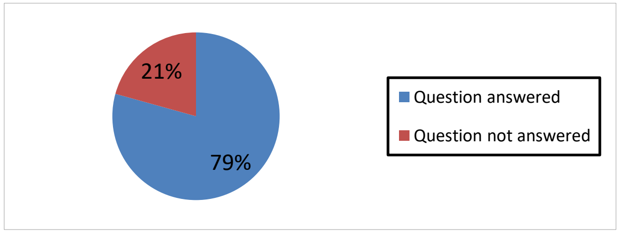

Results

Options	Variable	Code	Number	Frequency by participant	Frequency by answers
LC-MS (LC-HRMS, LC-MS/MS, ...)	V69	1	21	91.30%	47.73%
GC-MS (GC-HRMS, GC-MS/MS, ...)	V70	1	14	60.87%	31.82%
NMR	V71	1	4	17.39%	9.09%
Other/further/comments	V121	1	5	21.74%	11.36%
**Total**			44 Answers	23 Participants	

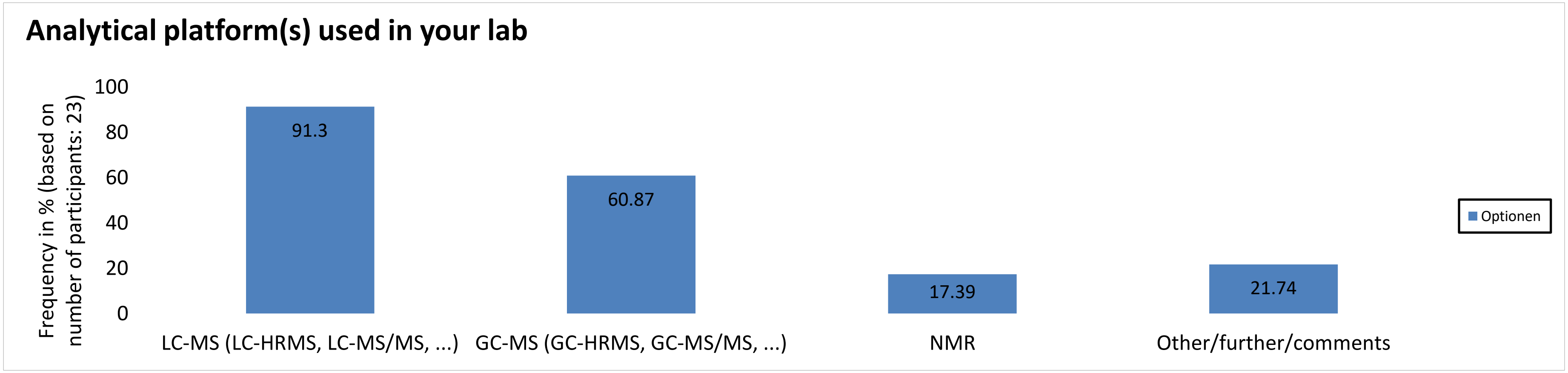

Question 6 - Analytical platform(s) used in your lab

Status: July 18, 2024, 13:37, Survey: "DGMet-Survey"

Number of participants evaluated: 29 (all participants)

Detailed results for entry field of Other/further/comments

Number Answers	5	Number unique	5

Value/Answer	Number	Frequency
DI-FT-ICR-MS	1	20%
DESI, TD	1	20%
Direct Infusion	1	20%
PTR-MS; IRMS	1	20%
GC, TLC	1	20%
**Total**	**5**	**100%**

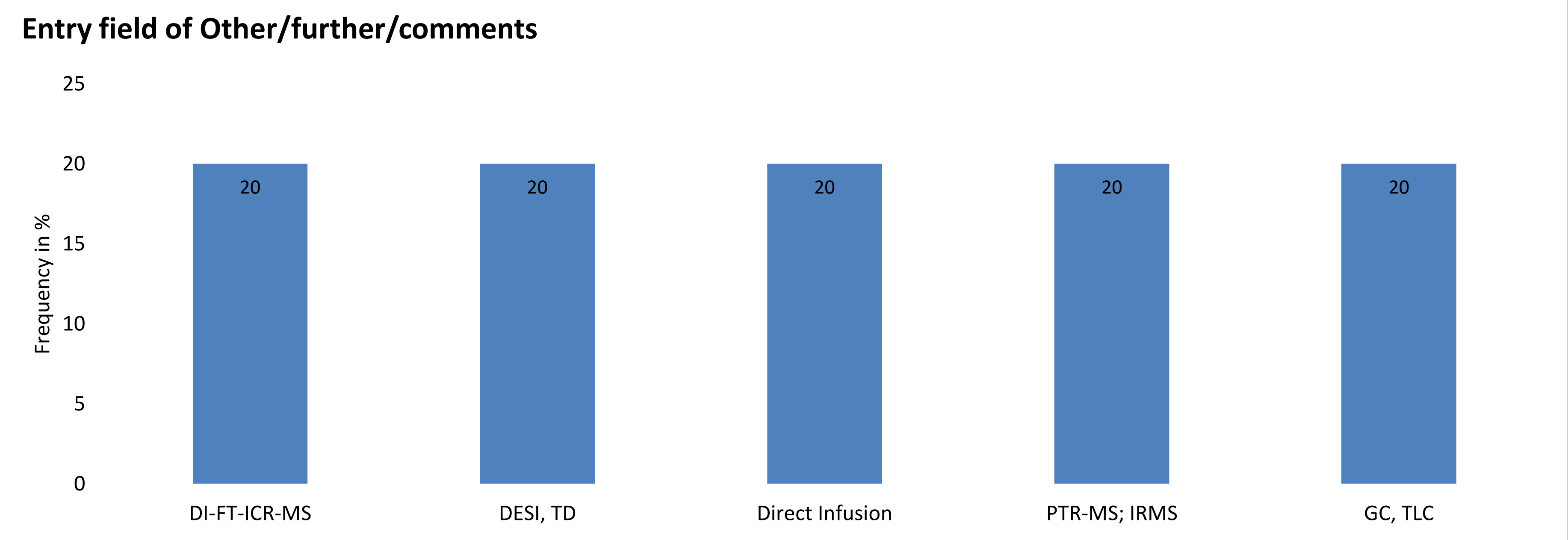

Question 7 - Type(s) of Mass Spectrometer (LC-MS)

Status: July 18, 2024, 13:37, Survey: "DGMet-Survey"

Number of participants evaluated: 29 (all participants)

Status data

of 29 participants	Number	Percent
Question seen	21	72.41%
Question answered	21	72.41%
Question not answered	8	27.59%

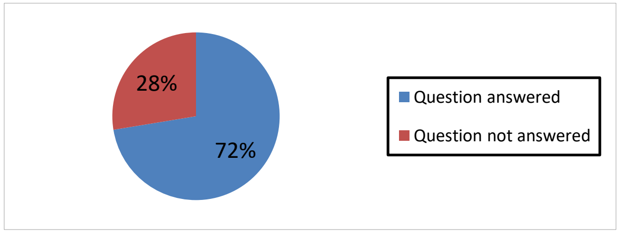

Results

Options	Variable	Code	Number	Frequency by participant	Frequency by answers
QQQ	V72	1	15	71.43%	30%
ToF	V73	1	6	28.57%	12%
QToF	V74	1	14	66.67%	28%
Orbitrap	V75	1	8	38.10%	16%
Iontrap	V124	1	5	23.81%	10%
Other/further/comments	V146	1	2	9.52%	4%
**Total**			50 Answers	21 Participants	

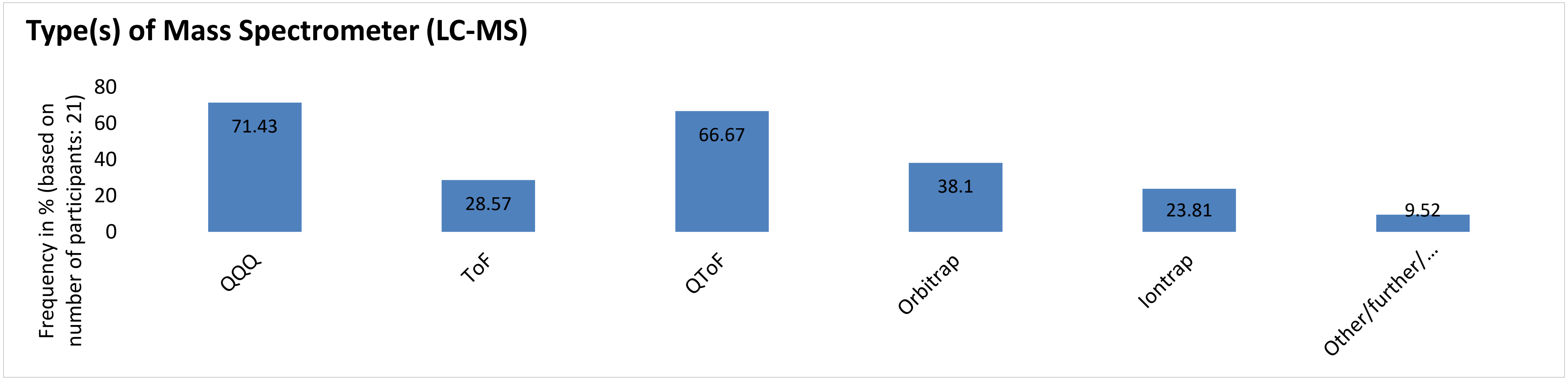

Question 7 - Type(s) of Mass Spectrometer (LC-MS)

Status: July 18, 2024, 13:37, Survey: "DGMet-Survey"

Number of participants evaluated: 29 (all participants)

Detailed results for entry field of Other/further/comments

Number Answers	2	Number unique	2

Value/Answer	Number	Frequency
FT-ICR	1	50%
Ionmobility	1	50%
**Total**	**2**	**100%**

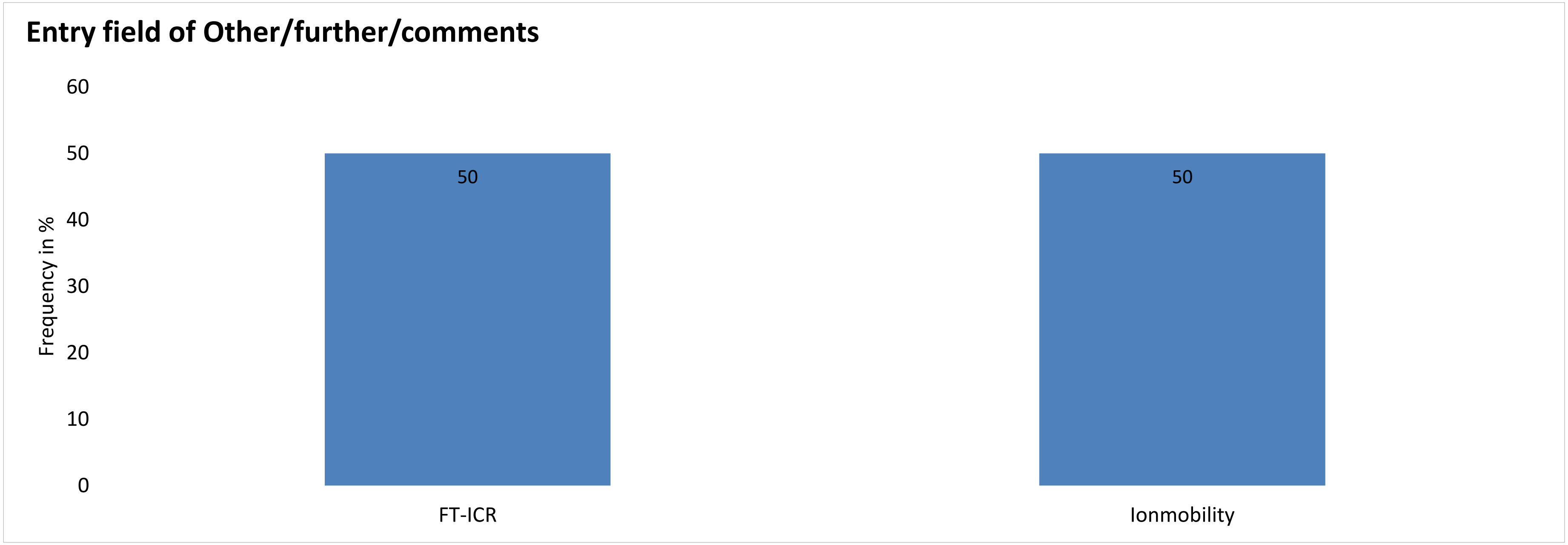

Question 8 - Type(s) of Chromatography (LC-MS)

Status: July 18, 2024, 13:37, Survey: "DGMet-Survey"

Number of participants evaluated: 29 (all participants)

Status data

of 29 participants	Number	Percent
Question seen	21	72.41%
Question answered	21	72.41%
Question not answered	8	27.59%

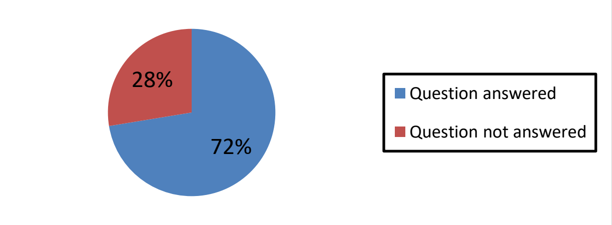

Results

Options	Variable	Code	Number	Frequency by participant	Frequency by answers
Reverse phase (C18, C8 and similar)	V80	1	20	95.24%	41.67%
Hydrophilic liquid interaction chromatography (HILIC)	V81	1	18	85.71%	37.50%
Normal phase (NP)	V82	1	2	9.52%	4.17%
Ion chromatography (IC)	V83	1	1	4.76%	2.08%
Supercritical fluid chromatography (SFC)	V141	1	1	4.76%	2.08%
FIA (no chromatography)	V142	1	6	28.57%	12.50%
Other/further/comments	V147	1	0	0%	0%
**Total**			48 Answers	21 Participants	

Question 8 - Type(s) of Chromatography (LC-MS)

Status: July 18, 2024, 13:37, Survey: "DGMet-Survey"

Number of participants evaluated: 29 (all participants)

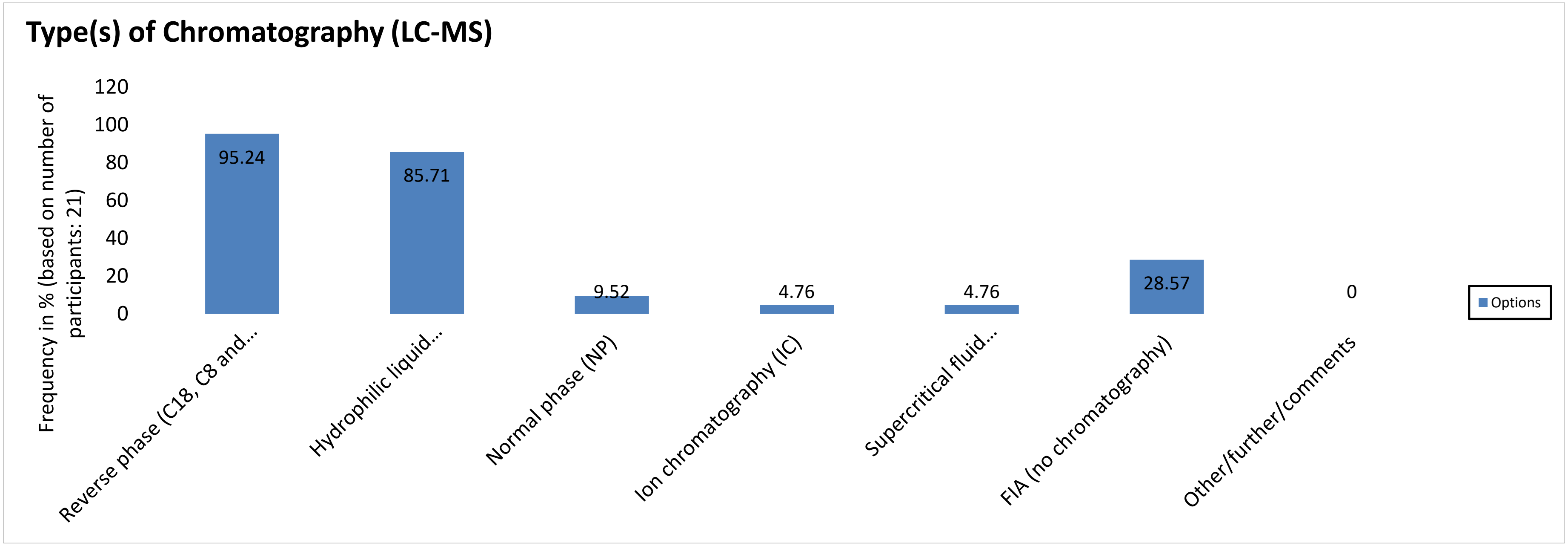

Question 8 - Type(s) of Chromatography (LC-MS)

Status: July 18, 2024, 13:37, Survey: "DGMet-Survey"

Number of participants evaluated: 29 (all participants)

Detailed results for entry field of Other/further/comments

Number Answers	0	Number unique	0

Value/Answer	Number	Frequency

Question 9 - Type(s) of Mass Spectrometer (GC-MS)

Status: July 18, 2024, 13:37, Survey: "DGMet-Survey"

Number of participants evaluated: 29 (all participants)

Status data

of 29 participants	Number	Percent
Question seen	14	48.28%
Question answered	14	48.28%
Question not answered	15	51.72%

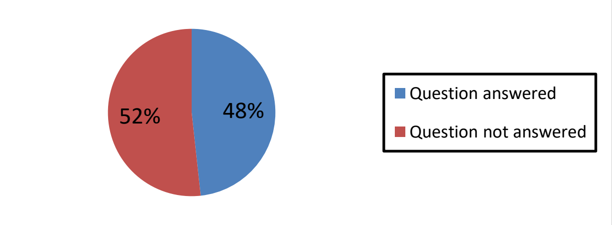

Results

Options	Variable	Code	Number	Frequency by participant	Frequency by answers
SingleQ	V76	1	9	64.29%	39.13%
QQQ	V77	1	7	50%	30.43%
ToF	V78	1	3	21.43%	13.04%
QToF	V79	1	2	14.29%	8.70%
Orbitrap	V125	1	1	7.14%	4.35%
Iontrap	V140	1	1	7.14%	4.35%
Other/further/comments	V145	1	0	0%	0%
**Total**			23 Answers	14 Participants	

Question 9 - Type(s) of Mass Spectrometer (GC-MS)

Status: July 18, 2024, 13:37, Survey: "DGMet-Survey"

Number of participants evaluated: 29 (all participants)

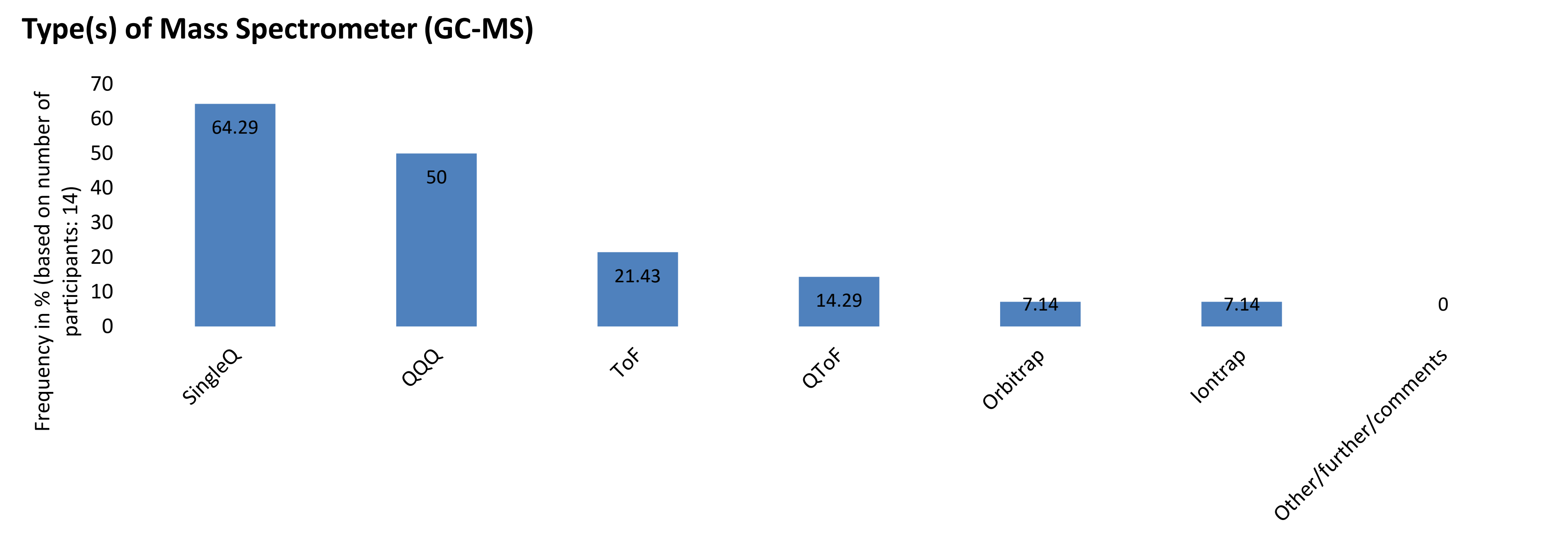

Question 9 - Type(s) of Mass Spectrometer (GC-MS)

Status: July 18, 2024, 13:37, Survey: "DGMet-Survey"

Number of participants evaluated: 29 (all participants)

Detailed results for entry field of Other/further/comments

Number Answers	0	Number unique	0

Value/Answer	Number	Frequency

Question 10 - Type(s) of Chromatography (GC-MS)

Status: July 18, 2024, 13:37, Survey: "DGMet-Survey"

Number of participants evaluated: 29 (all participants)

Status data

of 29 participants	Number	Percent
Question seen	14	48.28%
Question answered	14	48.28%
Question not answered	15	51.72%

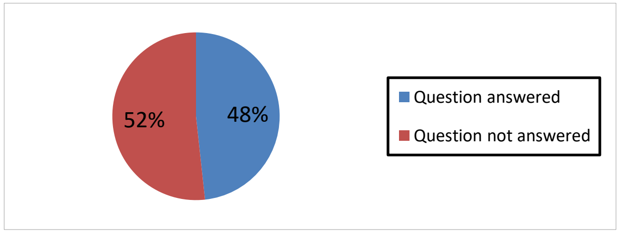

Results

Options	Variable	Code	Number	Frequency by participant	Frequency by answers
nonpolar (e.g. HP-5, DB-5, HP-1)	V84	1	12	85.71%	50%
midpolar (e.g. DB-17, DB-35, VF-624)	V85	1	6	42.86%	25%
polar (e.g. DB-WAX , Carbowax)	V86	1	5	35.71%	20.83%
GCxGC	V87	1	1	7.14%	4.17%
Other/further/comments	V99	1	0	0%	0%
**Total**			24 Answers	14 Participants	

Question 10 - Type(s) of Chromatography (GC-MS)

Status: July 18, 2024, 13:37, Survey: "DGMet-Survey"

Number of participants evaluated: 29 (all participants)

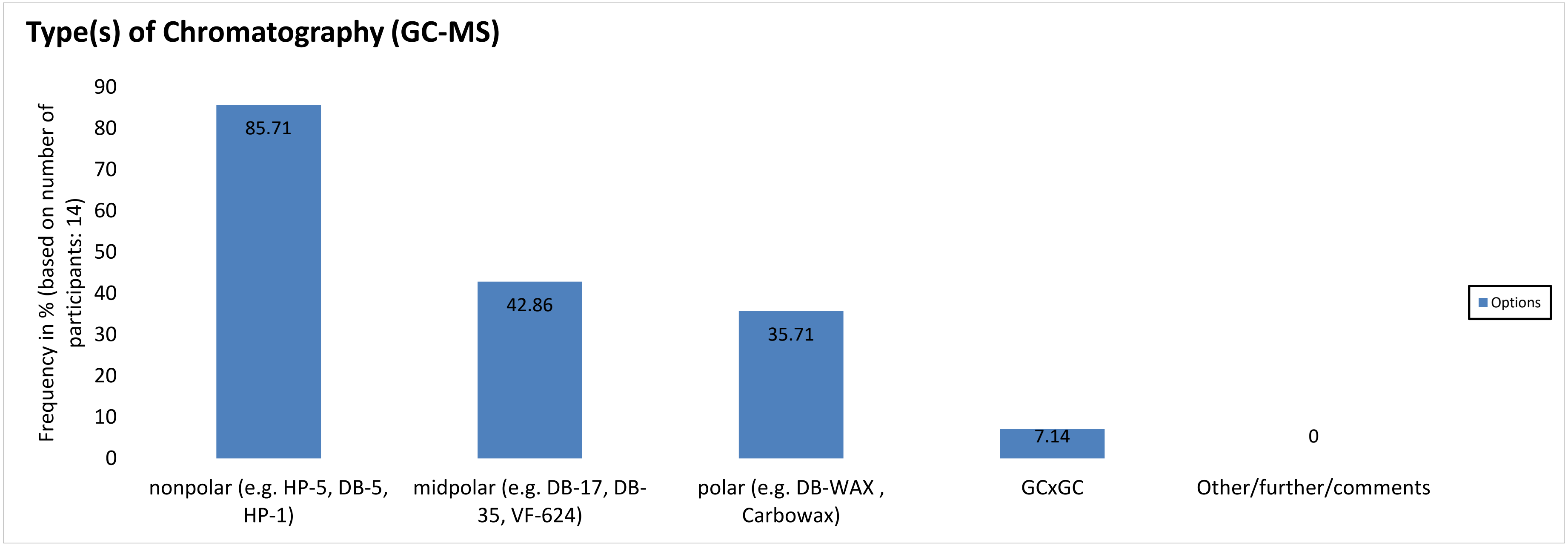

Question 10 - Type(s) of Chromatography (GC-MS)

Status: July 18, 2024, 13:37, Survey: "DGMet-Survey"

Number of participants evaluated: 29 (all participants)

Detailed results for entry field of Other/further/comments

Number Answers	0	Number unique	0

Value/Answer	Number	Frequency

Created with LamaPoll | https://www.lamapoll.de

Question 11 - Ion source(s) used in your lab

Status: July 18, 2024, 13:37, Survey: "DGMet-Survey"

Number of participants evaluated: 29 (all participants)

Status data

of 29 participants	Number	Percent
Question seen	23	79.31%
Question answered	21	72.41%
Question not answered	8	27.59%

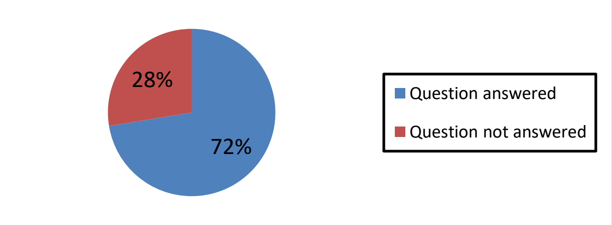

Results

Options	Variable	Code	Number	Frequency by participant	Frequency by answers
ESI	V88	1	19	90.48%	39.58%
APCI	V91	1	9	42.86%	18.75%
EI	V92	1	13	61.90%	27.08%
APPI	V93	1	0	0%	0%
(N)CI	V94	1	1	4.76%	2.08%
DESI	V95	1	1	4.76%	2.08%
DART	V96	1	0	0%	0%
MALDI	V97	1	4	19.05%	8.33%
Other/further/comments	V98	1	1	4.76%	2.08%
**Total**			48 Answers	21 Participants	

Created with LamaPoll | https://www.lamapoll.de

Question 11 - Ion source(s) used in your lab

Status: July 18, 2024, 13:37, Survey: "DGMet-Survey"

Number of participants evaluated: 29 (all participants)

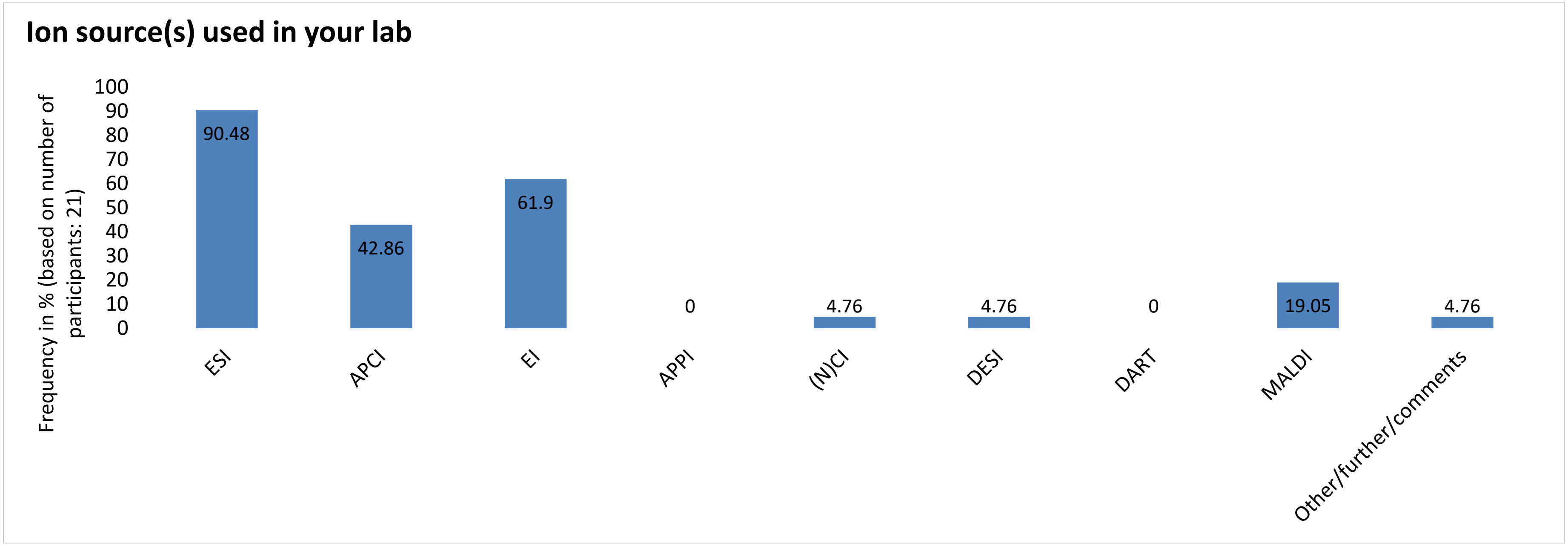

Question 11 - Ion source(s) used in your lab

Status: July 18, 2024, 13:37, Survey: "DGMet-Survey"

Number of participants evaluated: 29 (all participants)

Detailed results for entry field of Other/further/comments

Number Answers	1	Number unique	1

Value/Answer	Number	Frequency
PTR	1	100%
**Total**	**1**	**100%**

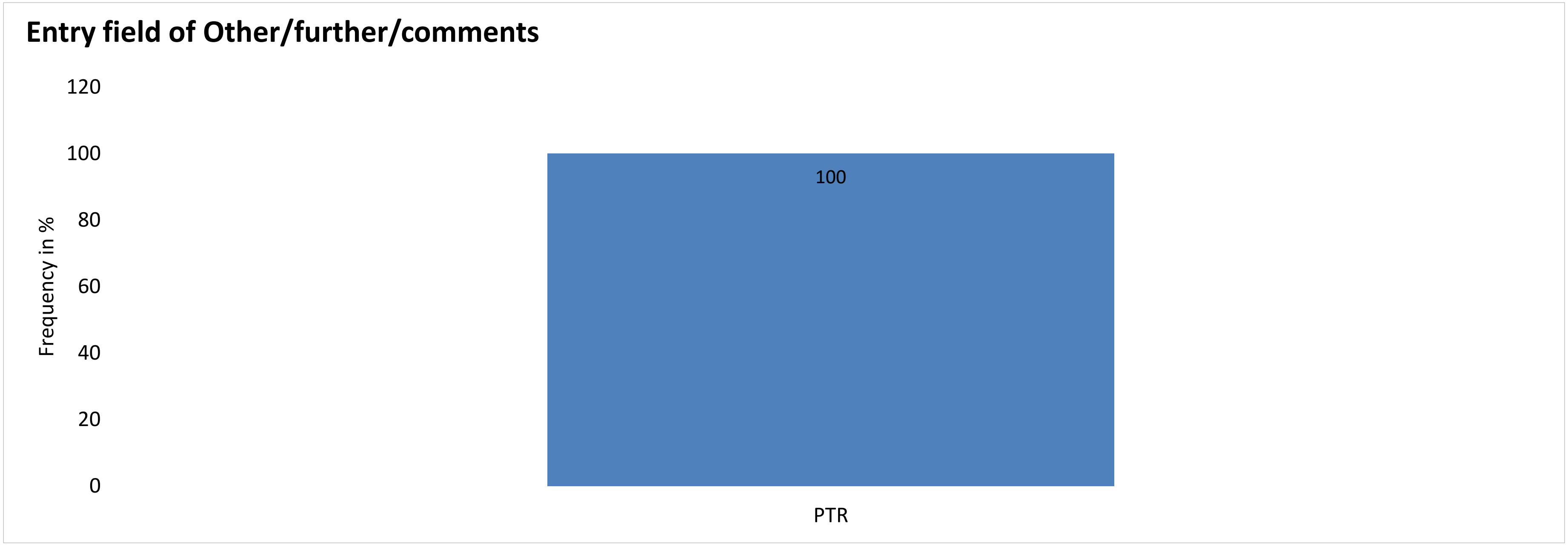

Question 12 - Ion Mobility Usage

Status: July 18, 2024, 13:37, Survey: "DGMet-Survey"

Number of participants evaluated: 29 (all participants)

Status data

of 29 participants	Number	Percent
Question seen	23	79.31%
Question answered	10	34.48%
Question not answered	19	65.52%

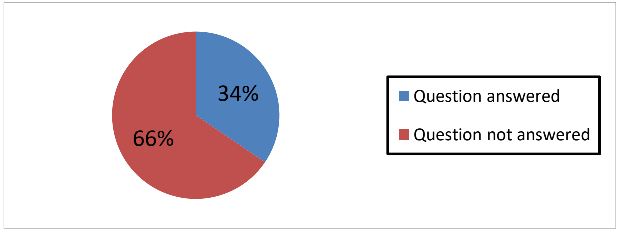

Results

Options	Variable	Code	Number	Frequency by participant	Frequency by answers
DMS	V20	1	4	40%	40%
TIMS	V21	1	3	30%	30%
Drift tube (DTIMS)	V22	1	1	10%	10%
TWIMS/cyclic IMS	V23	1	2	20%	20%
Other/further/comments	V66	1	0	0%	0%
**Total**			10 Answers	10 Participants	

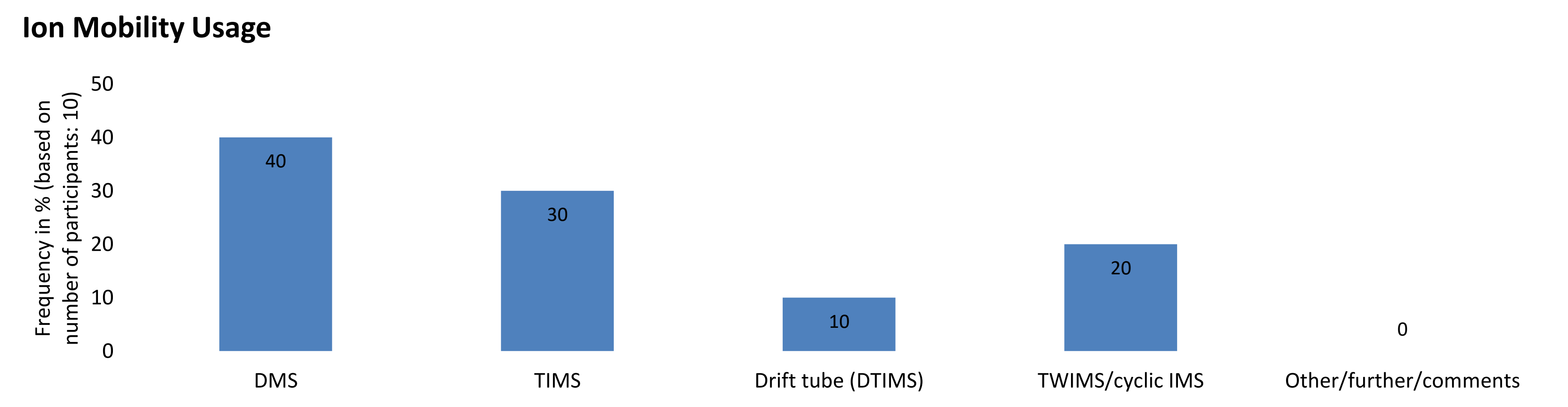

Question 12 - Ion Mobility Usage

Status: July 18, 2024, 13:37, Survey: "DGMet-Survey"

Number of participants evaluated: 29 (all participants)

Detailed results for entry field of Other/further/comments

Number Answers	0	Number unique	0

Value/Answer	Number	Frequency

Question 13 - Tell us which analysis strategies you use and give a score from 1 to 5 for how frequently you use it

Status: July 18, 2024, 13:37, Survey: "DGMet-Survey"

Number of participants evaluated: 29 (all participants)

Status data

of 29 participants	Number	Percent
Question seen	23	79.31%
Question answered	23	79.31%
Question not answered	6	20.69%

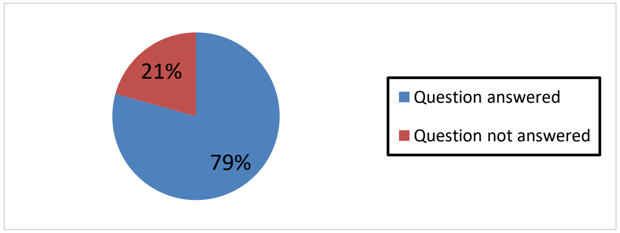

Rate from 1 = rarely to 5 = very often

Results (Total)

Frequency in %	Variable	Value 1	Value 2	Value 3	Value 4	Value 5	Total	no answer
LC-MS	V12	0%	0%	5.26%	10.53%	84.21%	19	17.39%
LC-UV or other detectors	V13	50%	18.75%	6.25%	6.25%	18.75%	16	30.43%
Direct infusion-MS	V14	29.41%	23.53%	0%	11.76%	35.29%	17	26.09%
GC-MS	V15	22.22%	22.22%	0%	16.67%	38.89%	18	21.74%
GC-FID or other detectors	V16	50%	8.33%	8.33%	16.67%	16.67%	12	47.83%
Ion mobility-MS	V67	33.33%	25%	8.33%	25%	8.33%	12	47.83%
NMR	V123	70%	10%	10%	0%	10%	10	56.52%
Raman spectroscopy	V131	100%	0%	0%	0%	0%	8	65.22%

Frequency Number	Variable	Value 1	Value 2	Value 3	Value 4	Value 5	Total	no answer	Average	Median
LC-MS	V12	0	0	1	2	16	19	4	4.79	5
LC-UV or other detectors	V13	8	3	1	1	3	16	7	2.25	1.50
Direct infusion-MS	V14	5	4	0	2	6	17	6	3	2
GC-MS	V15	4	4	0	3	7	18	5	3.28	4
GC-FID or other detectors	V16	6	1	1	2	2	12	11	2.42	1.50
Ion mobility-MS	V67	4	3	1	3	1	12	11	2.50	2
NMR	V123	7	1	1	0	1	10	13	1.70	1
Raman spectroscopy	V131	8	0	0	0	0	8	15	1	1
**Total**		42	16	5	13	36	**112**	72	**2.87**	**2**

Question 13 - Tell us which analysis strategies you use and give a score from 1 to 5 for how frequently you use it

Status: July 18, 2024, 13:37, Survey: "DGMet-Survey"

Number of participants evaluated: 29 (all participants)

Rate from 1 = rarely to 5 = very often

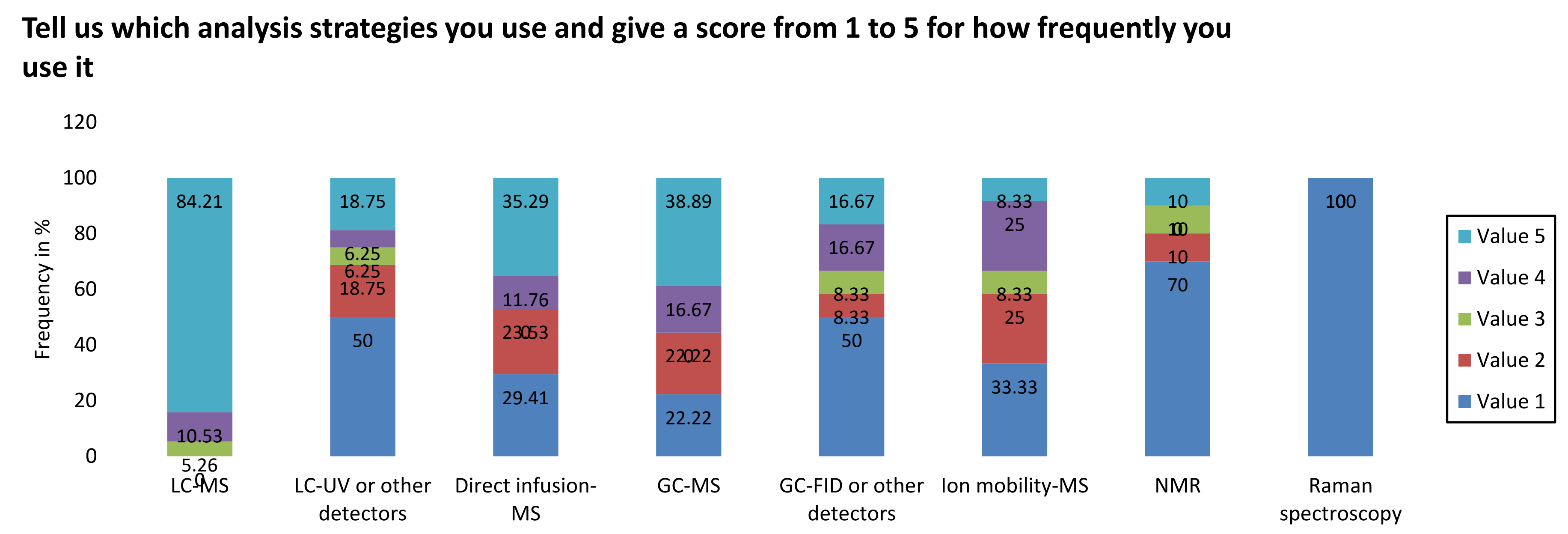

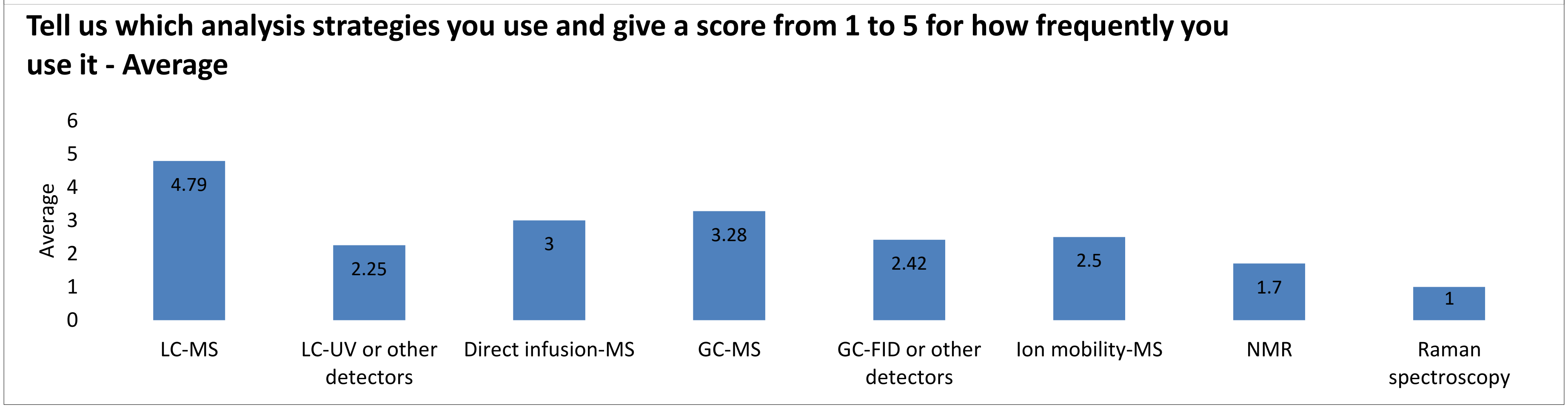

Created with LamaPoll | https://www.lamapoll.de

Question 13 - Tell us which analysis strategies you use and give a score from 1 to 5 for how frequently you use it

Status: July 18, 2024, 13:37, Survey: "DGMet-Survey"

Number of participants evaluated: 29 (all participants)

Detailed results for LC-MS

Variable	V12	Number of answers	19
Average	4.79	Median	5
Variance	0.27	Standard deviation	0.52
Smallest Value	3	Highest Value	5

Value/Answer	Number	Frequency
1	0	0%
2	0	0%
3	1	4.35%
4	2	8.70%
5	16	69.57%
no answer	4	17.39%
**Total**	**23**	**79.31%**

Rate from 1 = rarely to 5 = very often

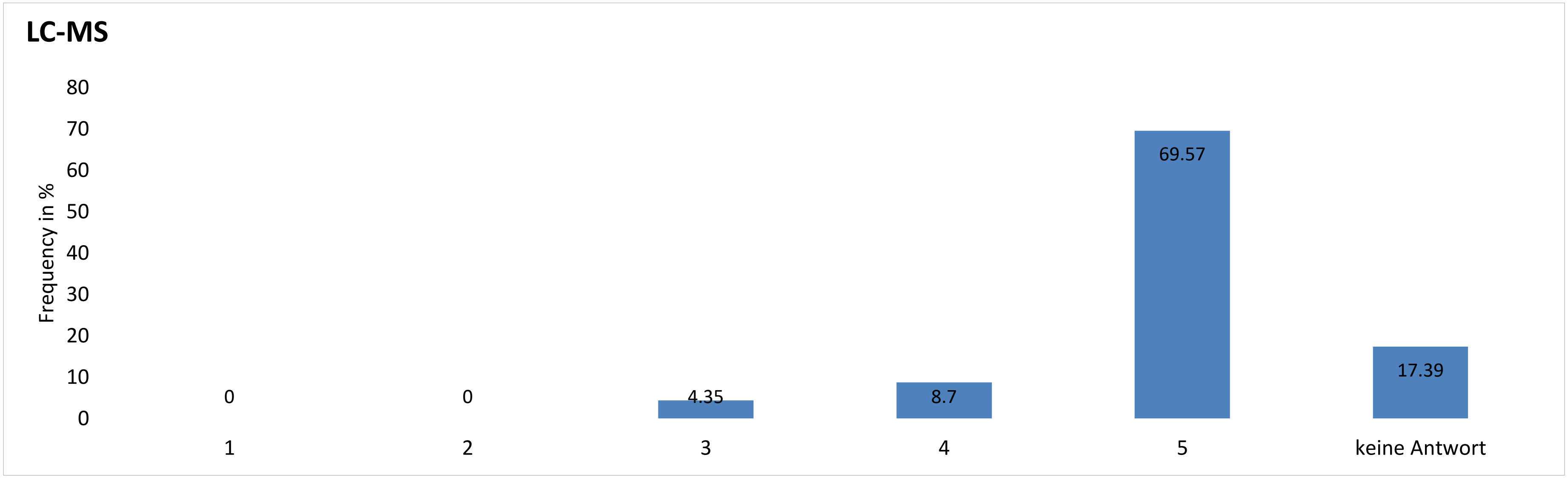

Question 13 - Tell us which analysis strategies you use and give a score from 1 to 5 for how frequently you use it

Status: July 18, 2024, 13:37, Survey: "DGMet-Survey"

Number of participants evaluated: 29 (all participants)

Detailed results for LC-UV or other detectors

Variable	V13	Number of answers	16
Average	2.25	Median	1.50
Variance	2.44	Standard deviation	1.56
Smallest Value	1	Highest Value	5

Value/Answer	Number	Frequency
1	8	34.78%
2	3	13.04%
3	1	4.35%
4	1	4.35%
5	3	13.04%
no answer	7	30.43%
**Total**	**23**	**79.31%**

Rate from 1 = rarely to 5 = very often

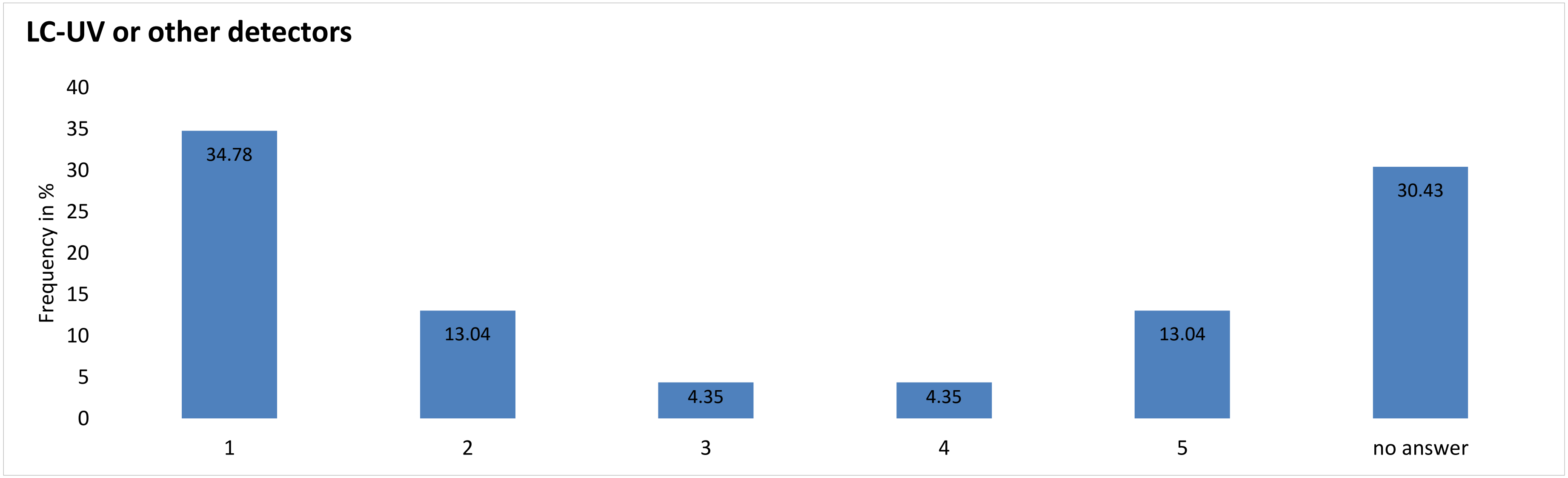

Question 13 - Tell us which analysis strategies you use and give a score from 1 to 5 for how frequently you use it

Status: July 18, 2024, 13:37, Survey: "DGMet-Survey"

Number of participants evaluated: 29 (all participants)

Detailed results for Direct infusion-MS

Variable	V14	Number of answers	17
Average	3	Median	2
Variance	2.94	Standard deviation	1.71
Smallest Value	1	Highest Value	5

Value/Answer	Number	Frequency
1	5	21.74%
2	4	17.39%
3	0	0%
4	2	8.70%
5	6	26.09%
no answer	6	26.09%
**Total**	**23**	**79.31%**

Rate from 1 = rarely to 5 = very often

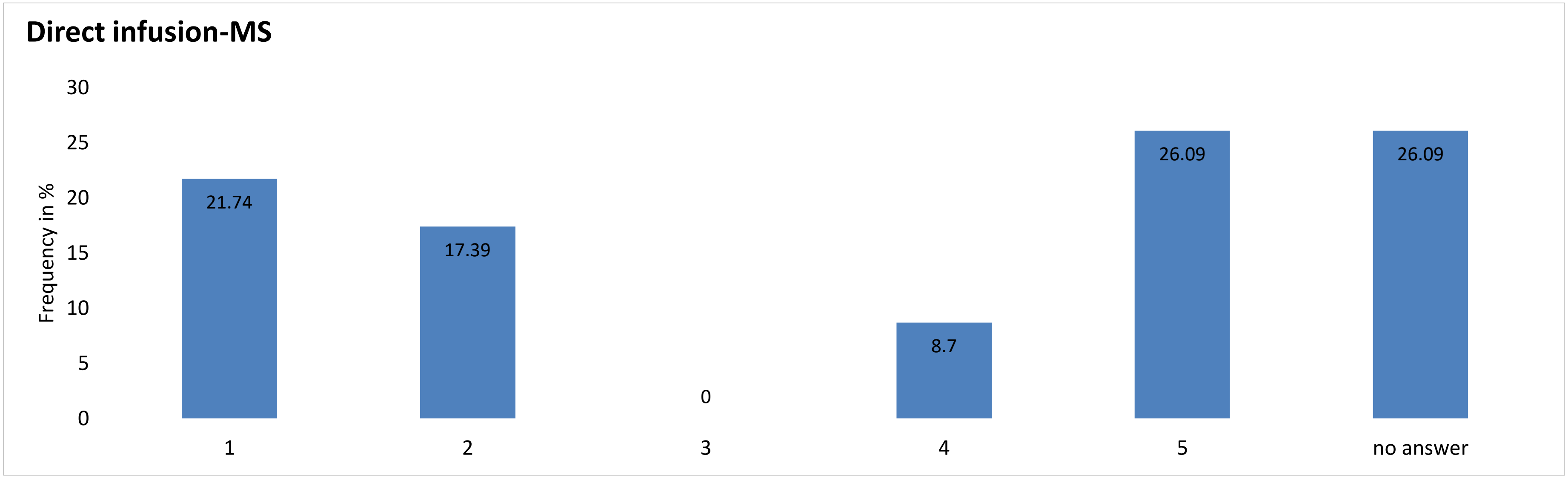

Question 13 - Tell us which analysis strategies you use and give a score from 1 to 5 for how frequently you use it

Status: July 18, 2024, 13:37, Survey: "DGMet-Survey"

Number of participants evaluated: 29 (all participants)

Detailed results for GC-MS

Variable	V15	Number of answers	18
Average	3.28	Median	4
Variance	2.76	Standard deviation	1.66
Smallest Value	1	Highest Value	5

Value/Answer	Number	Frequency
1	4	17.39%
2	4	17.39%
3	0	0%
4	3	13.04%
5	7	30.43%
no answer	5	21.74%
**Total**	**23**	**79.31%**

Rate from 1 = rarely to 5 = very often

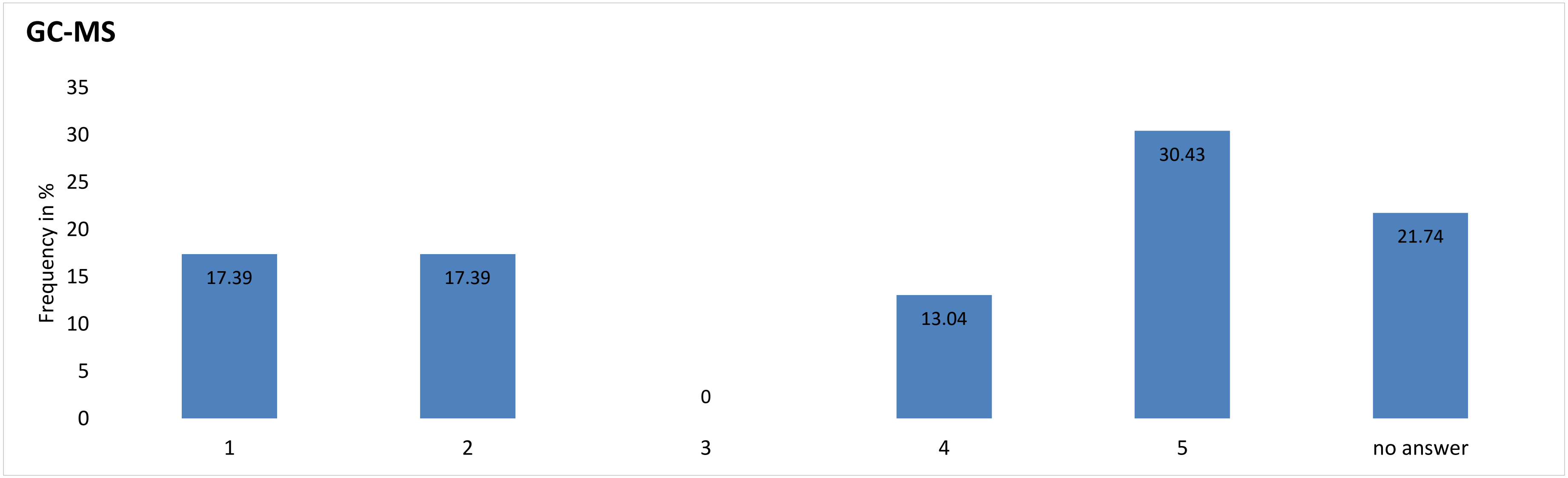

Question 13 - Tell us which analysis strategies you use and give a score from 1 to 5 for how frequently you use it

Status: July 18, 2024, 13:37, Survey: "DGMet-Survey"

Number of participants evaluated: 29 (all participants)

Detailed results for GC-FID or other detectors

Variable	V16	Number of answers	12
Average	2.42	Median	1.50
Variance	2.58	Standard deviation	1.61
Smallest Value	1	Highest Value	5

Value/Answer	Number	Frequency
1	6	26.09%
2	1	4.35%
3	1	4.35%
4	2	8.70%
5	2	8.70%
no answer	11	47.83%
**Total**	**23**	**79.31%**

Rate from 1 = rarely to 5 = very often

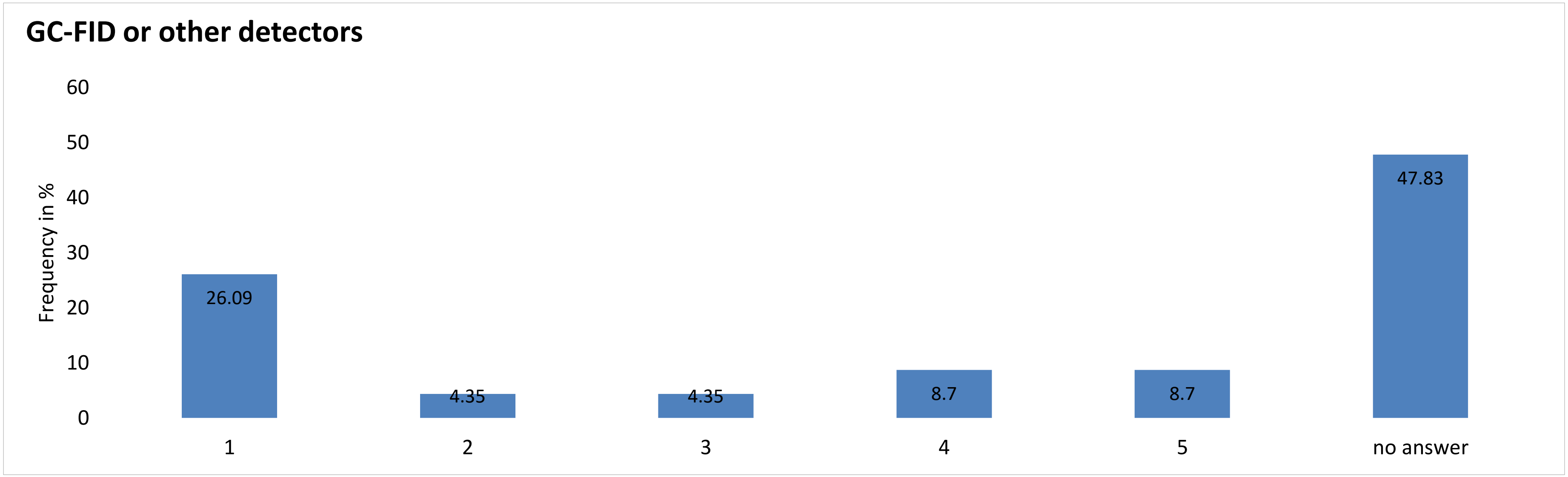

Question 13 - Tell us which analysis strategies you use and give a score from 1 to 5 for how frequently you use it

Status: July 18, 2024, 13:37, Survey: "DGMet-Survey"

Number of participants evaluated: 29 (all participants)

Detailed results for Ion mobility-MS

Variable	V67	Number of answers	12
Average	2.50	Median	2
Variance	1.92	Standard deviation	1.38
Smallest Value	1	Highest Value	5

Value/Answer	Number	Frequency
1	4	17.39%
2	3	13.04%
3	1	4.35%
4	3	13.04%
5	1	4.35%
no answer	11	47.83%
**Total**	**23**	**79.31%**

Rate from 1 = rarely to 5 = very often

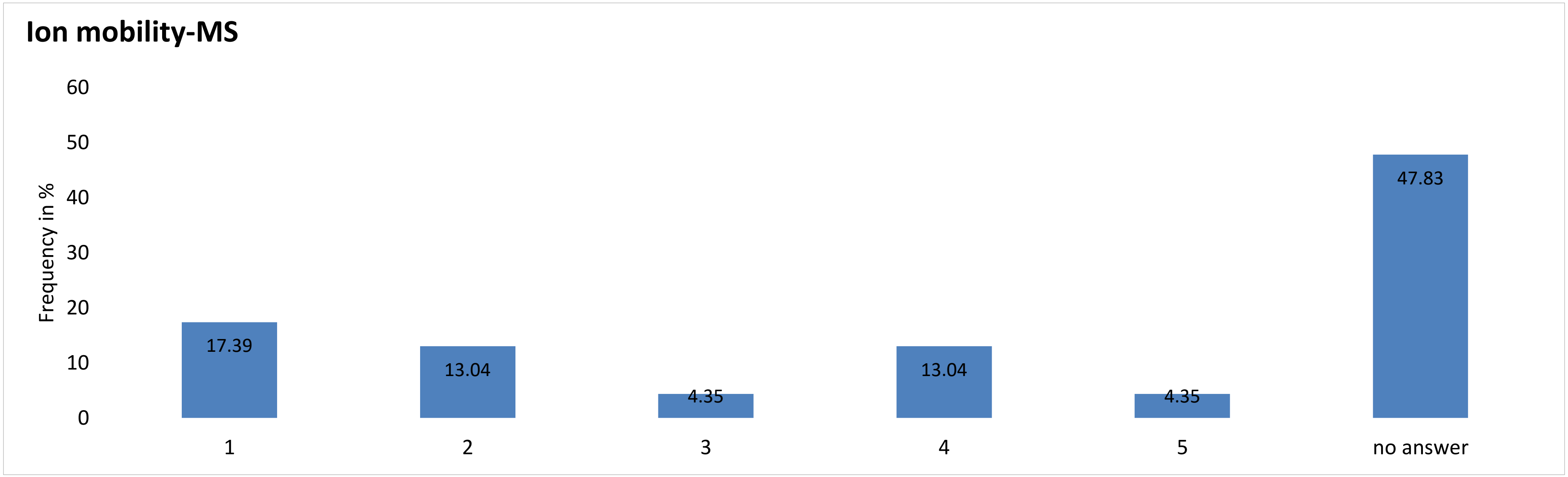

Created with LamaPoll | https://www.lamapoll.de

Question 13 - Tell us which analysis strategies you use and give a score from 1 to 5 for how frequently you use it

Status: July 18, 2024, 13:37, Survey: "DGMet-Survey"

Number of participants evaluated: 29 (all participants)

Detailed results for NMR

Variable	V123	Number of answers	10
Average	1.70	Median	1
Variance	1.61	Standard deviation	1.27
Smallest Value	1	Highest Value	5

Value/Answer	Number	Frequency
1	7	30.43%
2	1	4.35%
3	1	4.35%
4	0	0%
5	1	4.35%
no answer	13	56.52%
**Total**	**23**	**79.31%**

Rate from 1 = rarely to 5 = very often

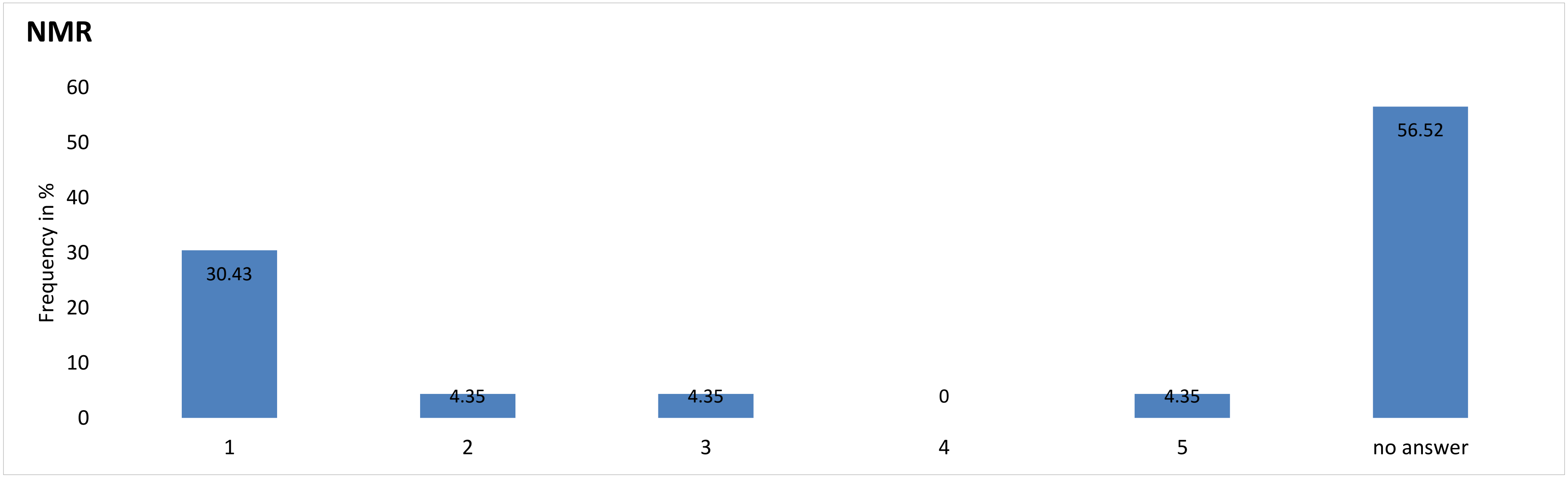

Question 13 - Tell us which analysis strategies you use and give a score from 1 to 5 for how frequently you use it

Status: July 18, 2024, 13:37, Survey: "DGMet-Survey"

Number of participants evaluated: 29 (all participants)

Detailed results for Raman spectroscopy

Variable	V131	Number of answers	8
Average	1	Median	1
Variance	0	Standard deviation	0
Smallest Value	1	Highest Value	1

Value/Answer	Number	Frequency
1	8	34.78%
2	0	0%
3	0	0%
4	0	0%
5	0	0%
no answer	15	65.22%
**Total**	**23**	**79.31%**

Rate from 1 = rarely to 5 = very often

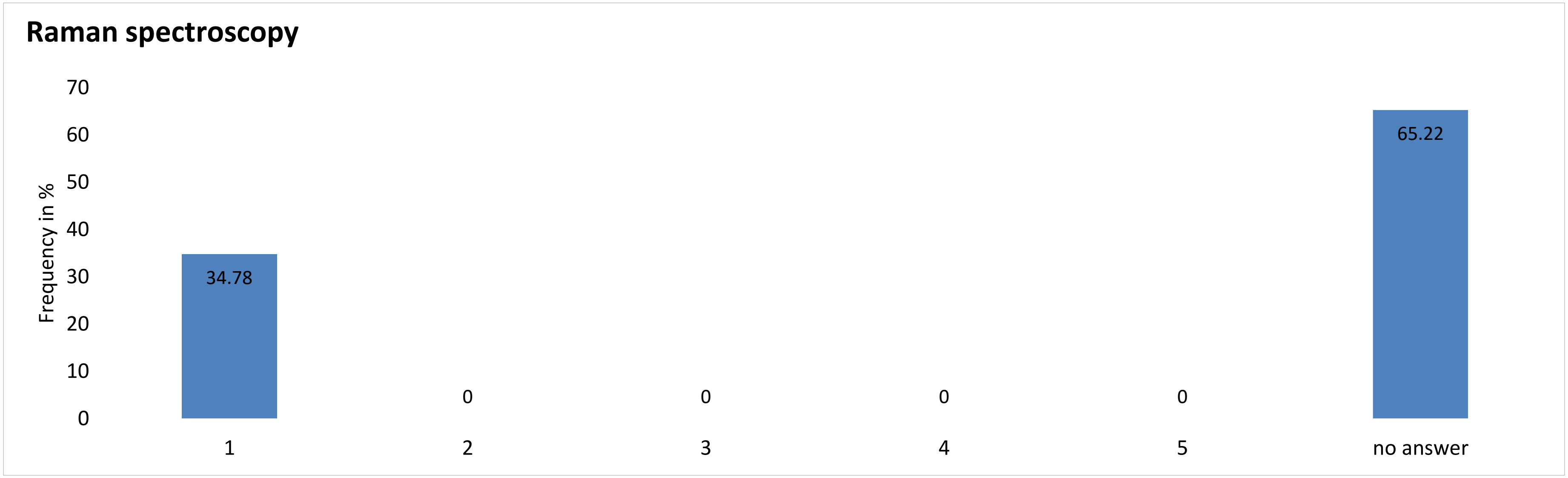

Question 14 - Expertise and Specialization

Status: July 18, 2024, 13:37, Survey: "DGMet-Survey"

Number of participants evaluated: 29 (all participants)

Status data

of 29 participants	Number	Percent
Question seen	23	79.31%
Question answered	16	55.17%
Question not answered	13	44.83%

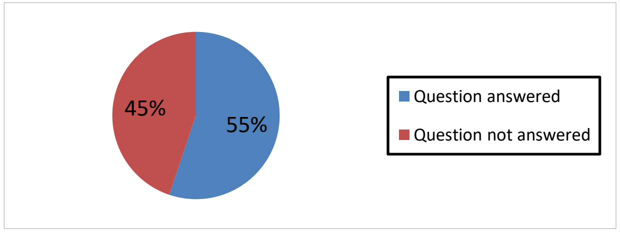

Detailed results for (1)

Number Answers	16	Number unique	16

Value/Answer	Number	Frequency
Most Projects: few broad and robust methods applied to a great variety of samples and questions	1	6.25%
High-throughput Metabolomics for cohorts (human, animal, plant, microorgansim)	1	6.25%
Targeted Methods for collaboration partner	1	6.25%
high-throughput metabolomics of large cohorts > 500 samples	1	6.25%
proteomics in combination with metabolomics	1	6.25%
microbial metabolomics with special focus on thermoacidophilic archaea	1	6.25%
organ cross communication	1	6.25%
biomarkers	1	6.25%
Broad targeted metabolomics	1	6.25%
lipidomics	1	6.25%
Microbial Metabolomics	1	6.25%
high-throughput metabolomics by NMR	1	6.25%
GC-MS based metabolite profiling, database and data processing	1	6.25%
Development of new analytical strategies	1	6.25%
volatilomics	1	6.25%
high throughput metabolomics of large farm animal cohorts	1	6.25%
**Total**	**16**	**100%**

Question 14 - Expertise and Specialization

Status: July 18, 2024, 13:37, Survey: "DGMet-Survey"

Number of participants evaluated: 29 (all participants)

(1)

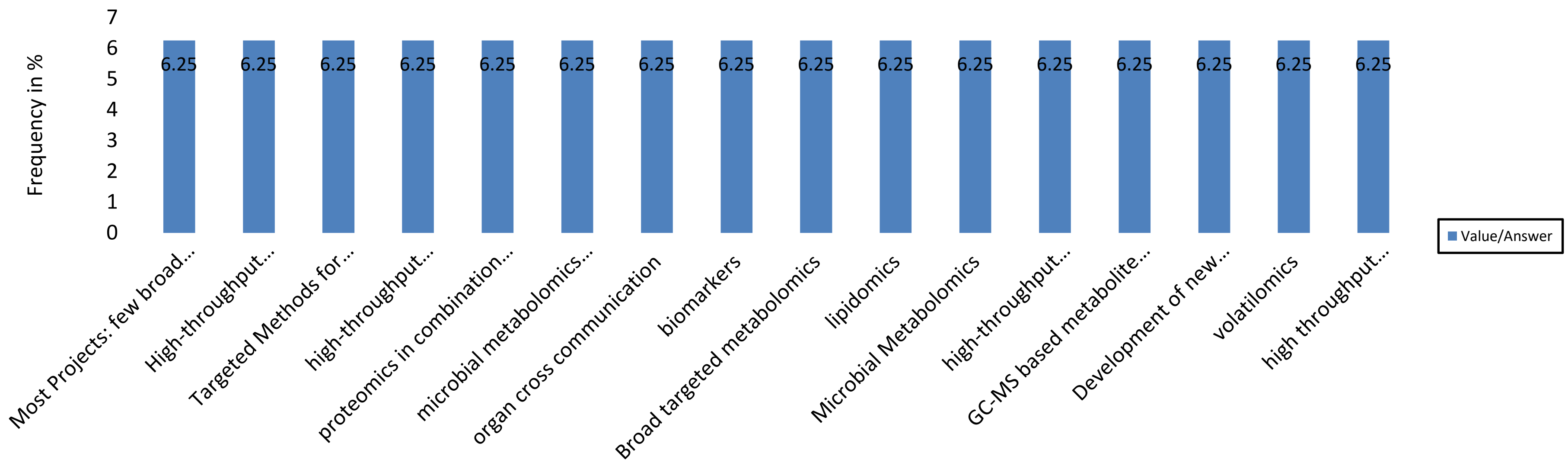

Question 14 - Expertise and Specialization

Status: July 18, 2024, 13:37, Survey: "DGMet-Survey"

Number of participants evaluated: 29 (all participants)

Detailed results for (2)

Number Answers	14	Number unique	14

Value/Answer	Number	Frequency
Special Expertise: Low input samples (≤ 5000 cells/sample)	1	7.14%
Establishing and validation of bioinformatic pipelines	1	7.14%
Development of new annotation workflows	1	7.14%
targeted methods which are user-specific	1	7.14%
lipid profiles of archaeal cell walls	1	7.14%
improving metabolomics quality	1	7.14%
Metabolomics applications for toxicology	1	7.14%
antibiotics quantitation	1	7.14%
Quantification of uptake of small molecules into bacterial cells	1	7.14%
development of computational metabolomics methods	1	7.14%
Specialized applications of GC-MS and LC-MS based metabolite profiling protocols in combination	1	7.14%
Metabolic flux in the big clinical picture	1	7.14%
non-targeted metabolomics	1	7.14%
high throughput lipidomics of large farm animal cohorts	1	7.14%
**Total**	**14**	**100%**

Created with LamaPoll | https://www.lamapoll.de

Question 14 - Expertise and Specialization

Status: July 18, 2024, 13:37, Survey: "DGMet-Survey"

Number of participants evaluated: 29 (all participants)

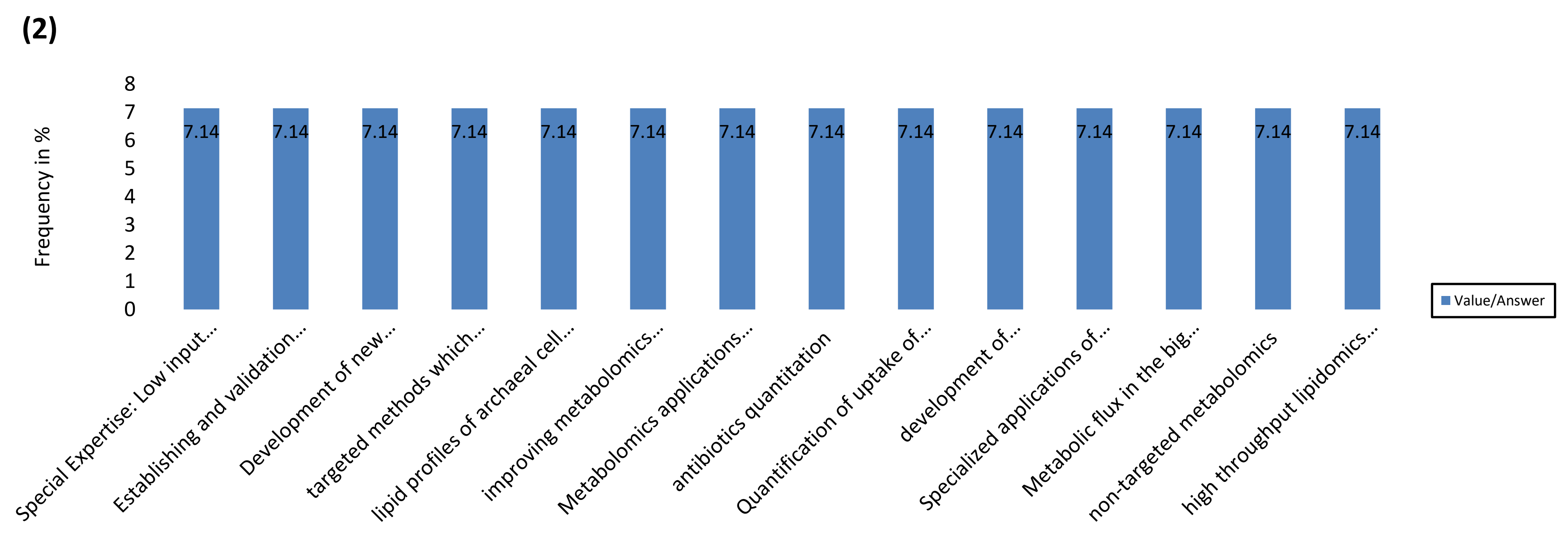

Created with LamaPoll | https://www.lamapoll.de

Question 14 - Expertise and Specialization

Status: July 18, 2024, 13:37, Survey: "DGMet-Survey"

Number of participants evaluated: 29 (all participants)

Detailed results for (3)

Number Answers	8	Number unique	8

Value/Answer	Number	Frequency
Method development for sample prep	1	12.50%
Method adaption for various biological matrices (e.g. cell culture, (animal) tissues...)	1	12.50%
inflammatory diseases	1	12.50%
Metabolomics applications for agricultural research	1	12.50%
structural analyses	1	12.50%
Method development for the analysis of untargeted metabolomics data	1	12.50%
Applications for 13C or 15N dynamic flux studies	1	12.50%
stable isotope technique	1	12.50%
Total	8	100%

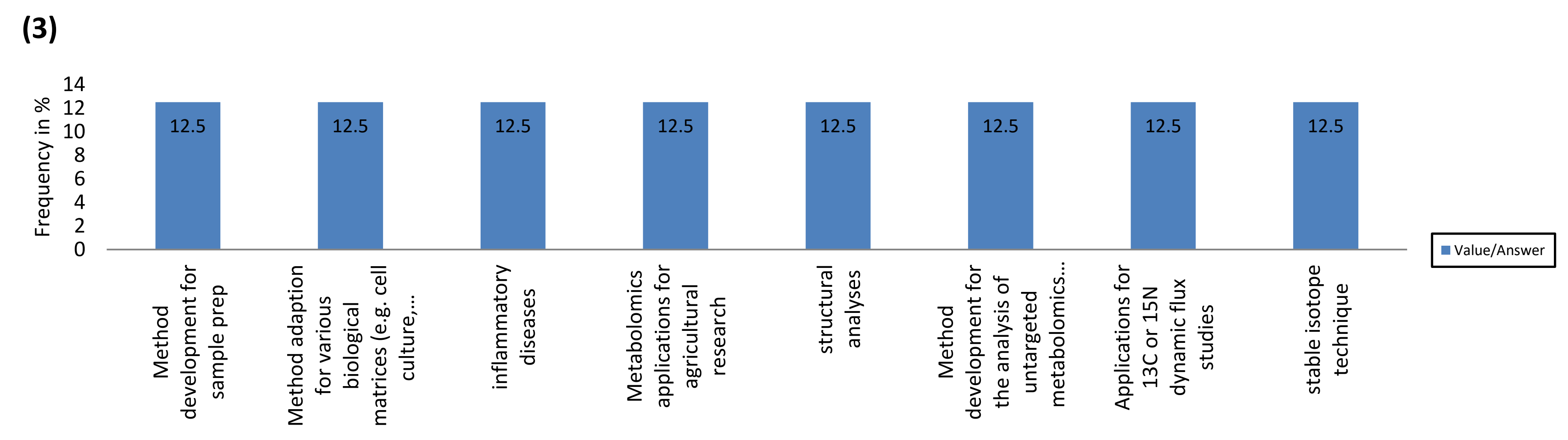

Question 15 - In-house Methods

Status: July 18, 2024, 13:37, Survey: "DGMet-Survey"

Number of participants evaluated: 29 (all participants)

Status data

of 29 participants	Number	Percent
Question seen	23	79.31%
Question answered	8	27.59%
Question not answered	21	72.41%

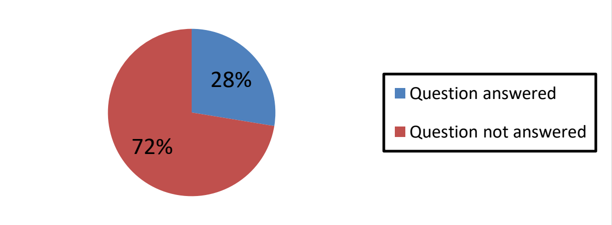

Detailed results for (1)

Number Answers	8	Number unique	8

Value/Answer	Number	Frequency
10.1007/978-1-4939-7592-1_12	1	12.50%
10.1021/acs.analchem.2c04396	1	12.50%
10.1039/d2ay01588a	1	12.50%
10.1093/bioinformatics/bti236	1	12.50%
Doi: 10.1093/bioinformatics/btz005	1	12.50%
Https://doi.org/10.1007/s10565-023-09809-6	1	12.50%
Https://doi.org/10.1016/j.talanta.2022.123298	1	12.50%
Https://doi.org/10.1111/nph.17608	1	12.50%
Total	8	100%

Question 15 - In-house Methods

Status: July 18, 2024, 13:37, Survey: "DGMet-Survey"

Number of participants evaluated: 29 (all participants)

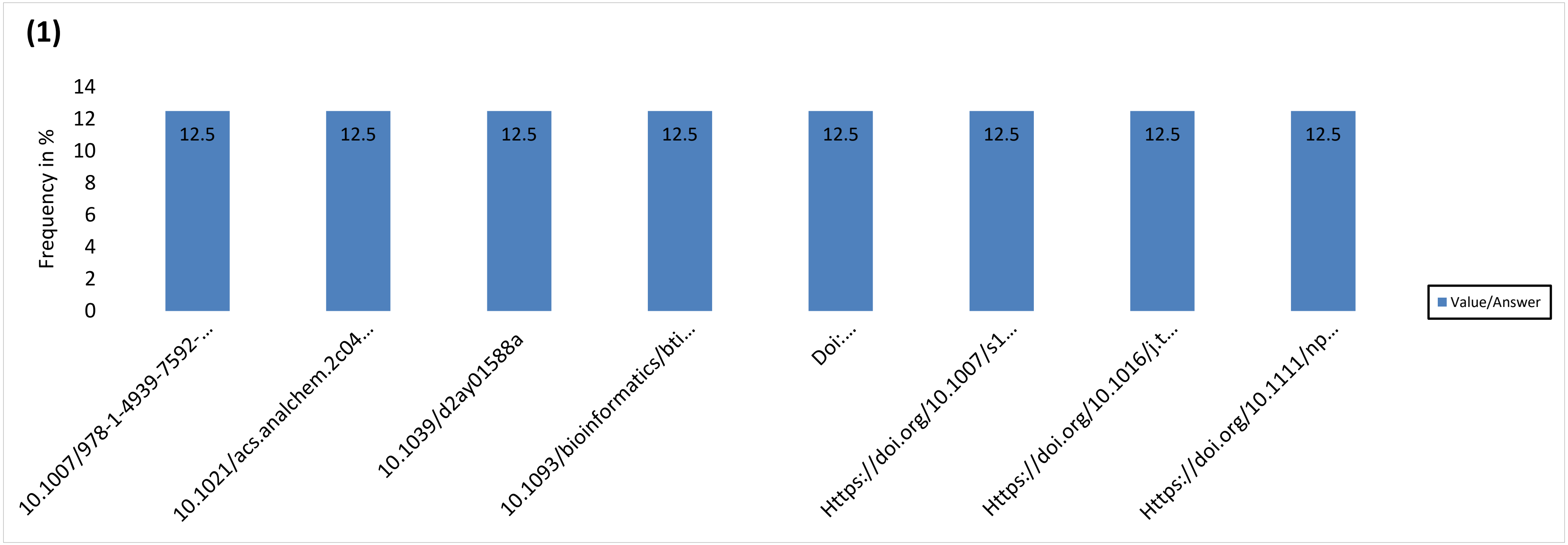

Question 15 - In-house Methods

Status: July 18, 2024, 13:37, Survey: "DGMet-Survey"

Number of participants evaluated: 29 (all participants)

Detailed results for (2)

Number Answers	8	Number unique	8

Value/Answer	Number	Frequency
10.1021/acs.analchem.1c05224	1	12.50%
10.1093/bioinformatics/btn023	1	12.50%
10.1186/gb-2011-12-1-r8	1	12.50%
10.1681/asn.2022030378	1	12.50%
10.3389/fmolb.2022.968643	1	12.50%
Doi: 10.1021/acs.analchem.8b03586	1	12.50%
Https://doi.org/10.1016/j.toxlet.2014.07.021	1	12.50%
Https://doi.org/10.1093/treephys/tpad087	1	12.50%
**Total**	**8**	**100%**

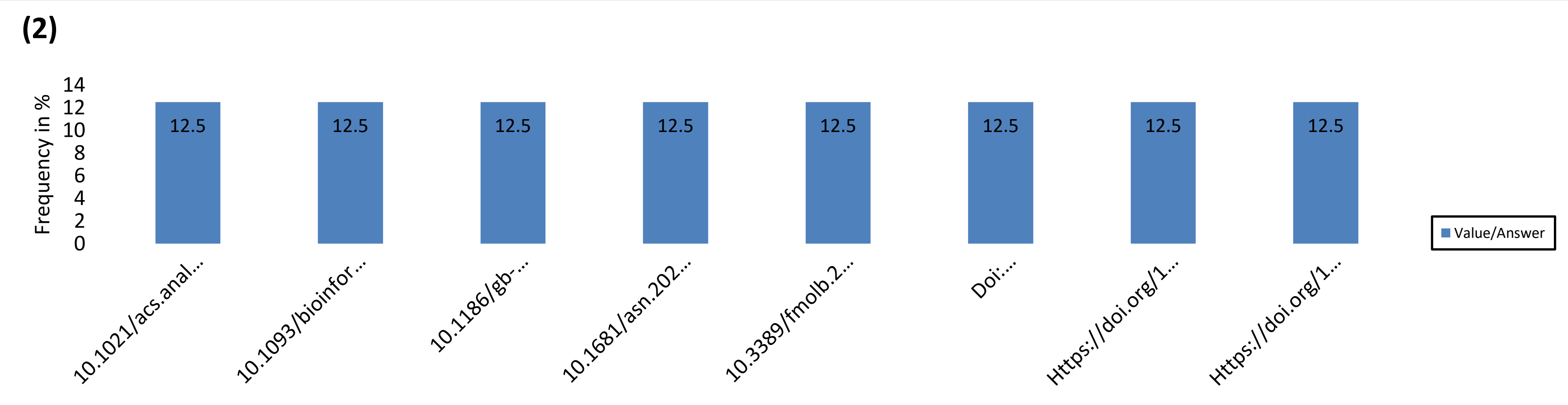

Question 15 - In-house Methods

Status: July 18, 2024, 13:37, Survey: "DGMet-Survey"

Number of participants evaluated: 29 (all participants)

Detailed results for (3)

Number Answers	7	Number unique	7

Value/Answer	Number	Frequency
10.1007/978-1-0716-0660-5_15	1	14.29%
10.1038/s41467-020-15633-x	1	14.29%
10.1194/jlr.d700041-jlr200	1	14.29%
10.3390/metabo11010012	1	14.29%
Doi: 10.1104/pp.114.236018	1	14.29%
Https://doi.org/10.1101/2023.03.26.532286	1	14.29%
Https://doi.org/10.3390/metabo11120888	1	14.29%
**Total**	**7**	**100%**

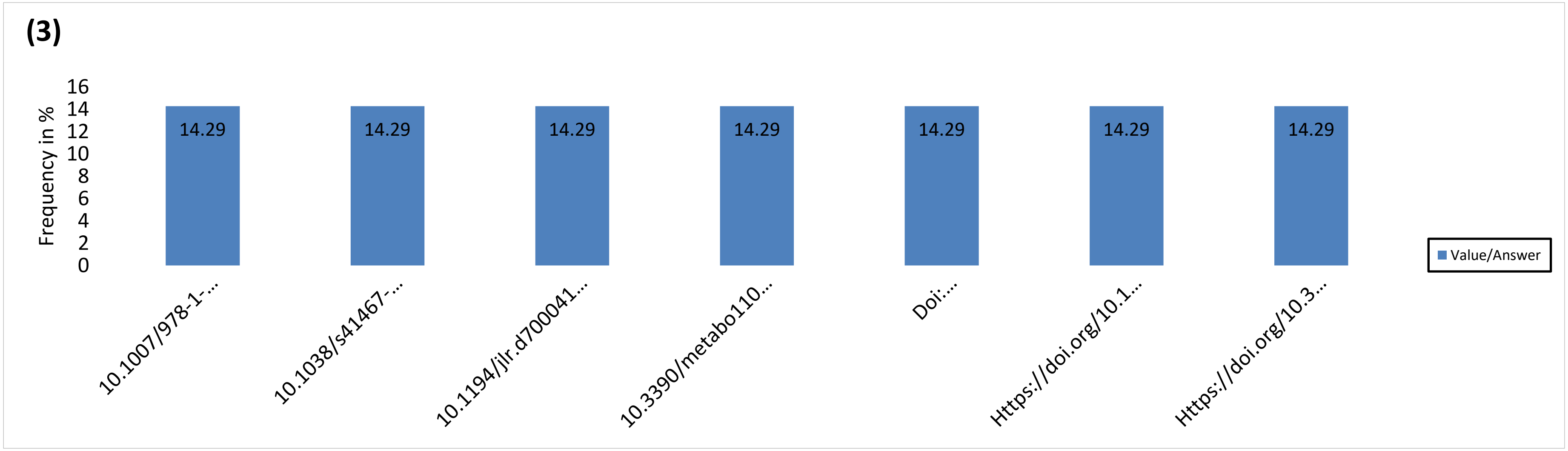

Question 16 - Commercial Kits (covering the complete workflow)

Status: July 18, 2024, 13:37, Survey: "DGMet-Survey"

Number of participants evaluated: 29 (all participants)

Status data

of 29 participants	Number	Percent
Question seen	23	79.31%
Question answered	8	27.59%
Question not answered	21	72.41%

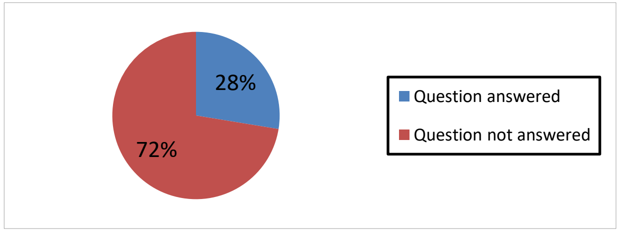

Results

Options	Variable	Code	Number	Frequency by participant	Frequency by answers
AbsoluteIDQ(R) p180	V24	1	3	37.50%	14.29%
AbsoluteIDQ(R) p400 HR	V25	1	1	12.50%	4.76%
MxP(R) Quant 500	V26	1	6	75%	28.57%
MxP(R) Quant HR XpressTM	V27	1	2	25%	9.52%
AbsoluteIDQ(R) Stero 17	V28	1	2	25%	9.52%
AbsoluteIDQ(R) Bile acids	V100	1	3	37.50%	14.29%
LipidyzerTM platform	V101	1	2	25%	9.52%
Other/further/comments	V126	1	2	25%	9.52%
**Total**			21 Answers	8 Participants	

Created with LamaPoll | https://www.lamapoll.de

Question 16 - Commercial Kits (covering the complete workflow)

Status: July 18, 2024, 13:37, Survey: "DGMet-Survey"

Number of participants evaluated: 29 (all participants)

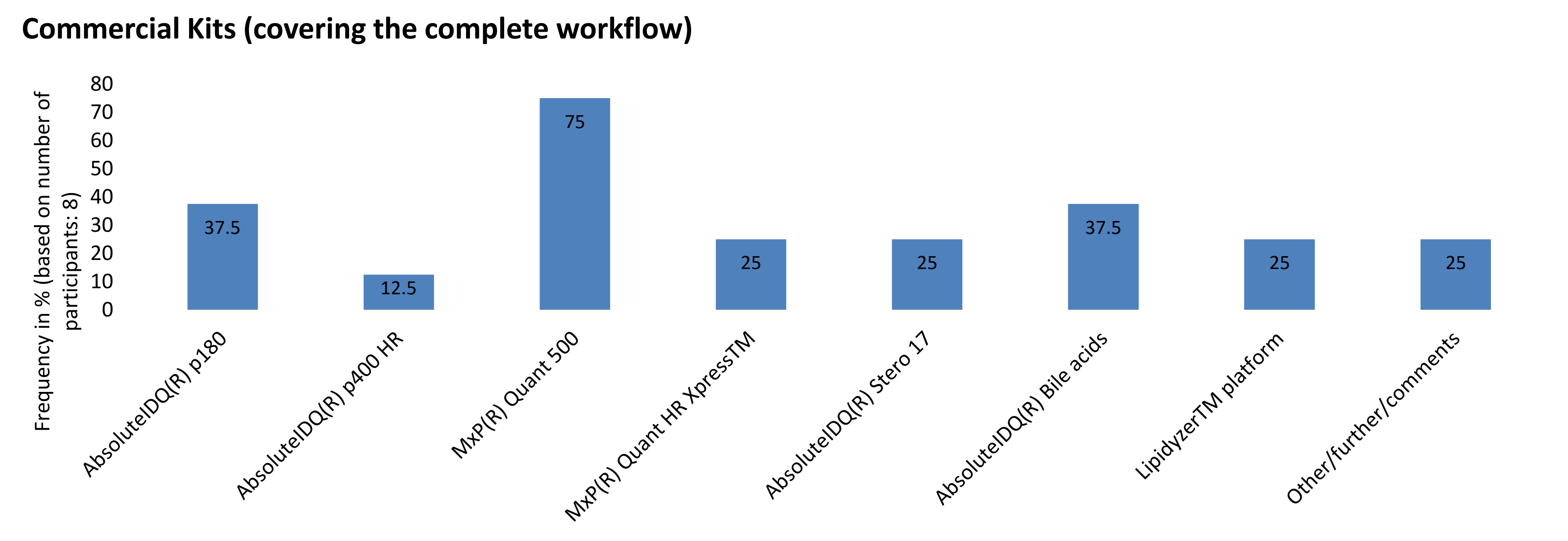

Question 16 - Commercial Kits (covering the complete workflow)

Status: July 18, 2024, 13:37, Survey: "DGMet-Survey"

Number of participants evaluated: 29 (all participants)

Detailed results for entry field of Other/further/comments

Number Answers	2	Number unique	2

Value/Answer	Number	Frequency
No commercial kits in use	1	50%
Bruker IVDr	1	50%
**Total**	**2**	**100%**

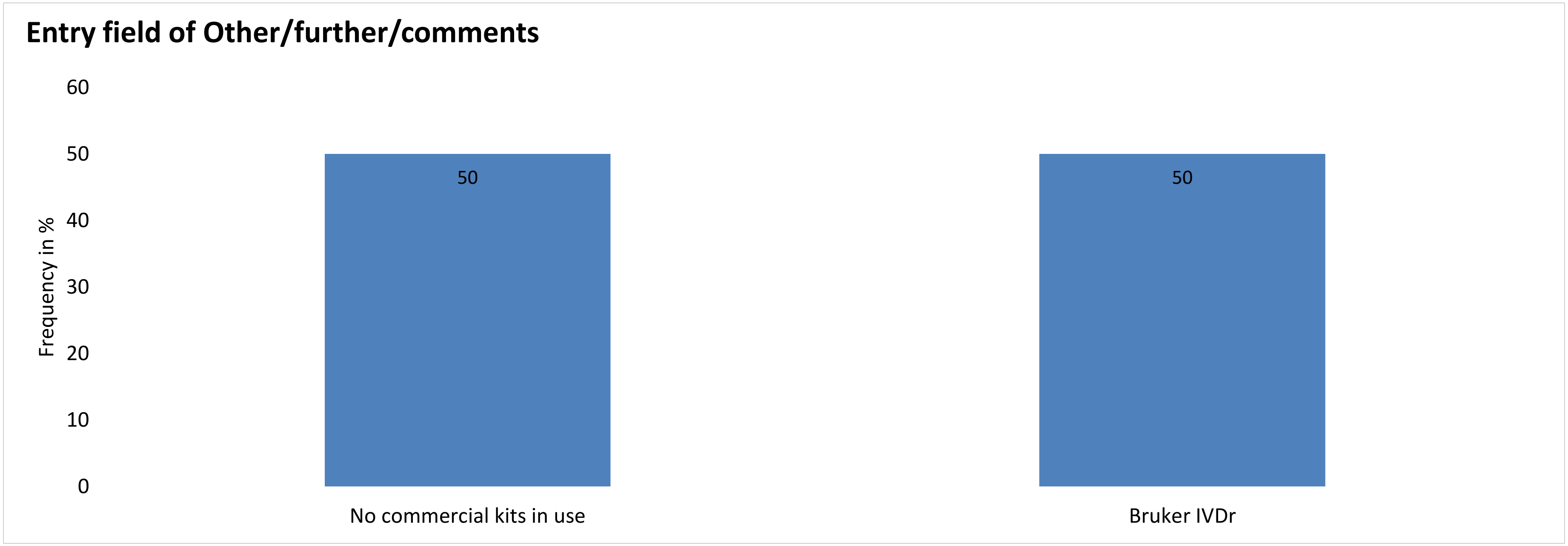

Question 17 - Do you use a modified kit protocol?

Status: July 18, 2024, 13:37, Survey: "DGMet-Survey"

Number of participants evaluated: 29 (all participants)

Status data

of 29 participants	Number	Percent
Question seen	8	27.59%
Question answered	7	24.14%
Question not answered	22	75.86%

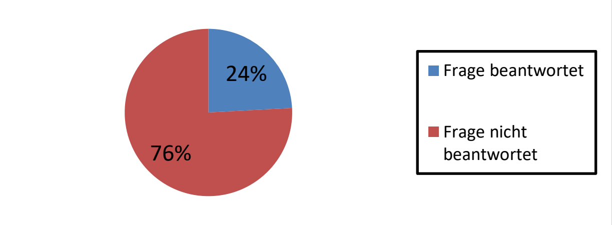

Results

Options	Variable	Code	Number	Frequency by participant	Frequency by answers
Yes	V89	1	3	42.86%	42.86%
No	V90	1	4	57.14%	57.14%
**Total**			7 Answers	7 Participants	

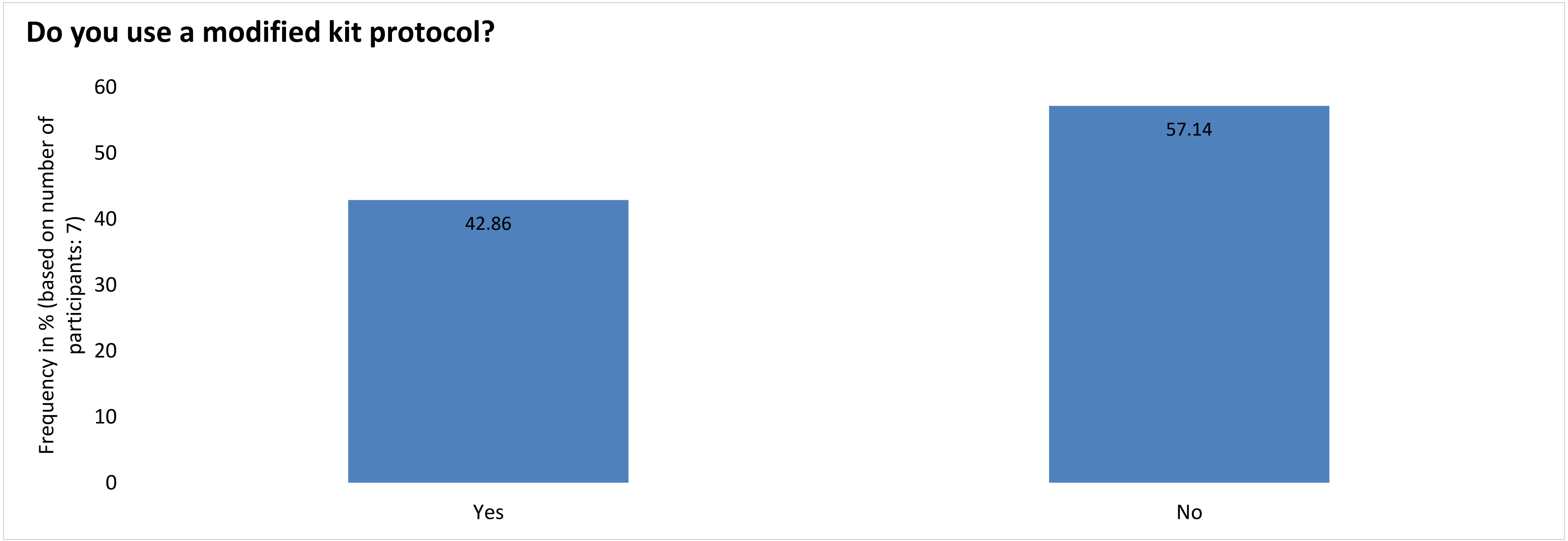

Question 18 - Please let us know, what part of the protocol you modified

Status: July 18, 2024, 13:37, Survey: "DGMet-Survey"

Number of participants evaluated: 29 (all participants)

Status data

of 29 participants	Number	Percent
Question seen	3	10.34%
Question answered	3	10.34%
Question not answered	26	89.66%

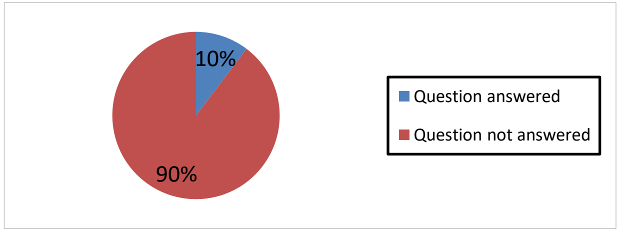

Results

Options	Variable	Code	Number	Frequency by participant	Frequency by answers
Mass spectrometric parameters (e.g. collision energy, gases)	V127	1	1	33.33%	20%
Chromatographic parameters (e.g. injection volume, flow rate)	V128	1	1	33.33%	20%
Sample preparation	V129	1	3	100%	60%
Other/further/comments	V130	1	0	0%	0%
**Total**			5 Answers	3 Participants	

Question 18 - Please let us know, what part of the protocol you modified

Status: July 18, 2024, 13:37, Survey: "DGMet-Survey"

Number of participants evaluated: 29 (all participants)

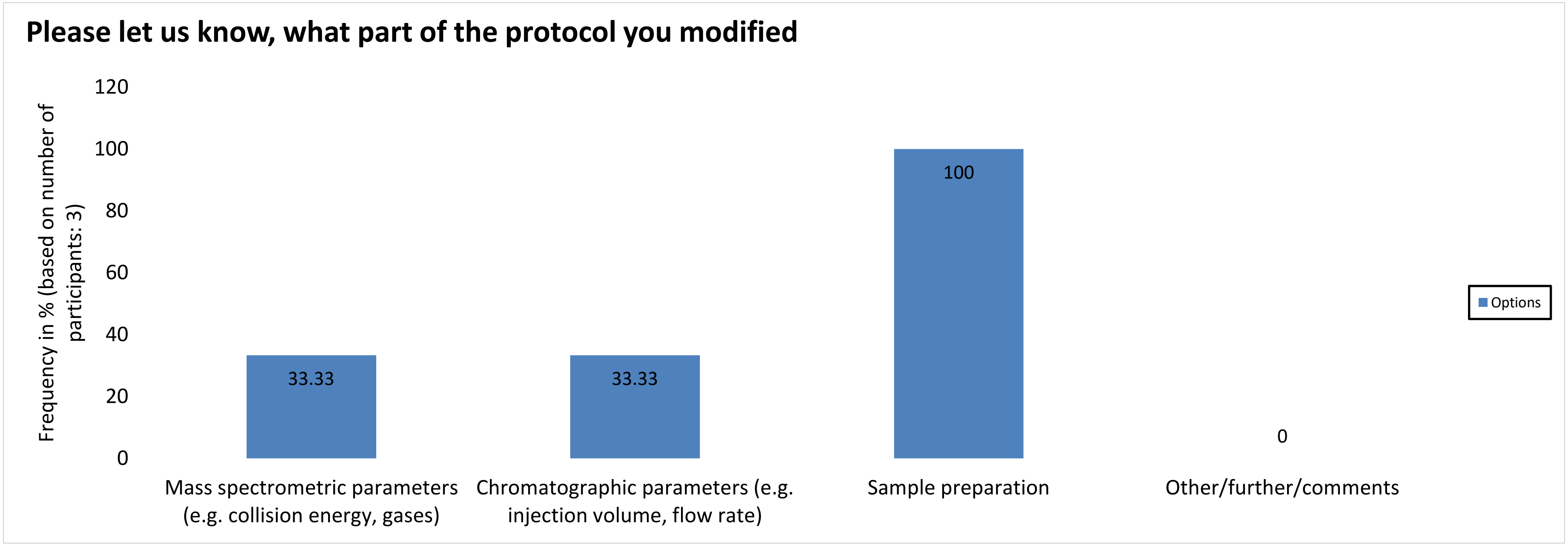

Question 18 - Please let us know, what part of the protocol you modified

Status: July 18, 2024, 13:37, Survey: "DGMet-Survey"

Number of participants evaluated: 29 (all participants)

Detailed results for entry field of Other/further/comments

Number Answers	0	Number unique	0

Value/Answer	Number	Frequency

Question 19 - Use of chemical standards

Status: July 18, 2024, 13:37, Survey: "DGMet-Survey"

Number of participants evaluated: 29 (all participants)

Status data

of 29 participants	Number	Percent
Question seen	23	79.31%
Question answered	22	75.86%
Question not answered	7	24.14%

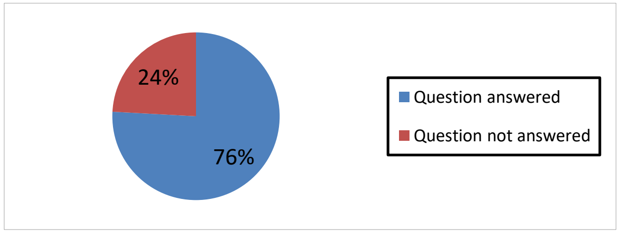

Results

Options	Variable	Code	Number	Frequency by participant	Frequency by answers
Instrument qualification (e.g. mass calibration with tune mix)	V33	1	19	86.36%	17.43%
System suitability tests (e.g. test mix of standards)	V34	1	14	63.64%	12.84%
Analytical method validation	V35	1	14	63.64%	12.84%
Quality control (QC) purposes	V45	1	12	54.55%	11.01%
Calibration standards for quantification	V46	1	18	81.82%	16.51%
Metabolite identification	V47	1	17	77.27%	15.60%
Bridging across study sample data	V48	1	4	18.18%	3.67%
Data pre-processing (e.g. normalization)	V64	1	11	50%	10.09%
Other/further/comments	V65	1	0	0%	0%
**Total**			109 Answers	22 Participants	

Question 19 - Use of chemical standards

Status: July 18, 2024, 13:37, Survey: "DGMet-Survey"

Number of participants evaluated: 29 (all participants)

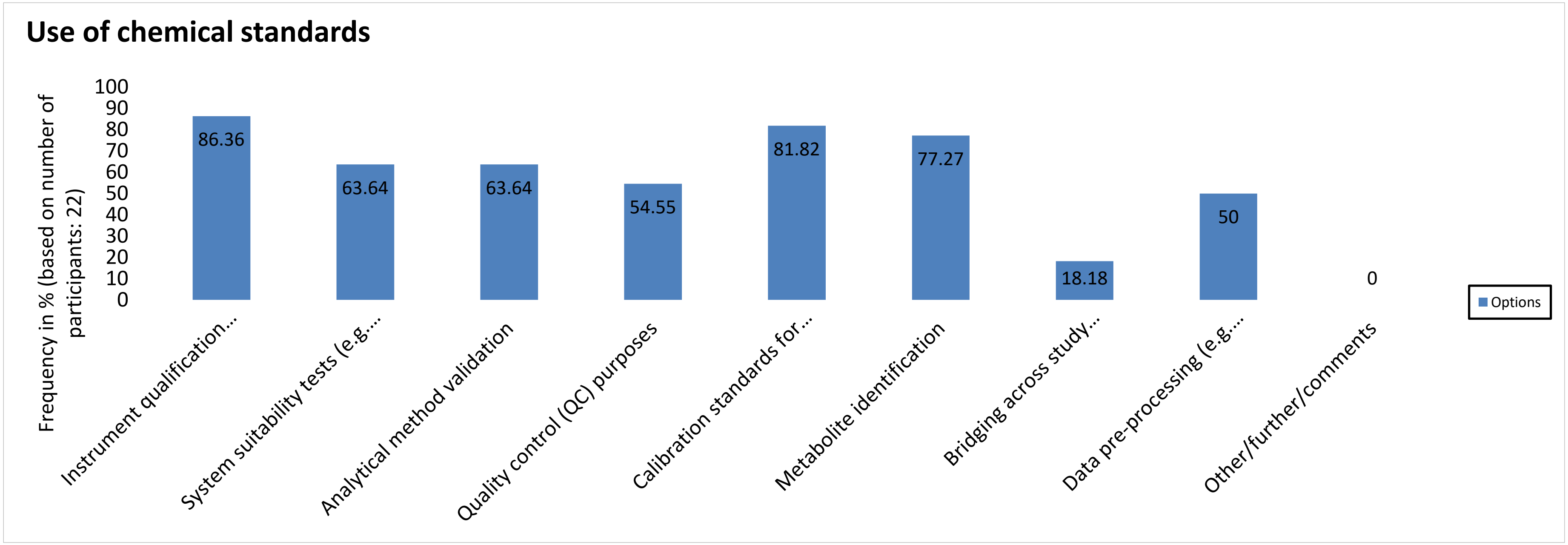

Question 19 - Use of chemical standards

Status: July 18, 2024, 13:37, Survey: "DGMet-Survey"

Number of participants evaluated: 29 (all participants)

Detailed results for entry field of Other/further/comments

Number Answers	0	Number unique	0

Value/Answer	Number	Frequency

Question 20 - Chemical standard mixtures used in your lab

Status: July 18, 2024, 13:37, Survey: "DGMet-Survey"

Number of participants evaluated: 29 (all participants)

Status data

of 29 participants	Number	Percent
Question seen	23	79.31%
Question answered	21	72.41%
Question not answered	8	27.59%

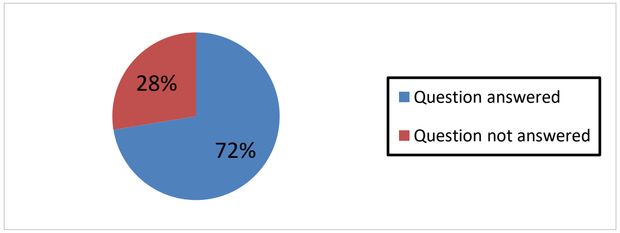

Results

Options	Variable	Code	Number	Frequency by participant	Frequency by answers
Commercially available compound mixtures or libraries	V102	1	19	90.48%	52.78%
In-house prepared compound mixtures	V103	1	17	80.95%	47.22%
**Total**			36 Answers	21 Participants	

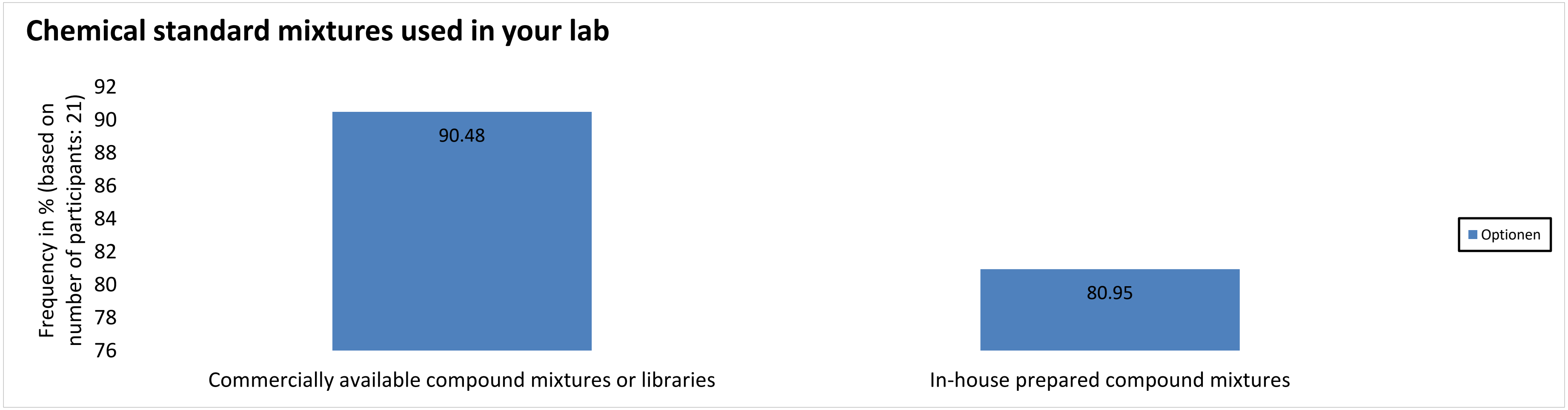

Question 21 - Commercially available standard mixtures

Status: July 18, 2024, 13:37, Survey: "DGMet-Survey"

Number of participants evaluated: 29 (all participants)

Status data

of 29 participants	Number	Percent
Question seen	23	79.31%
Question answered	19	65.52%
Question not answered	10	34.48%

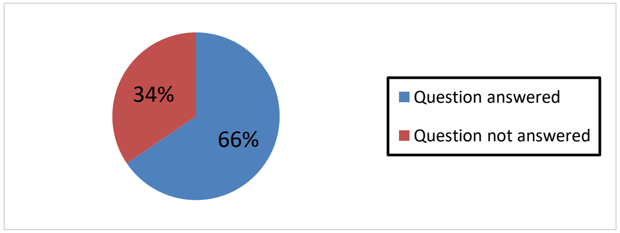

Results

Options	Variable	Code	Number	Frequency by participant	Frequency by answers
Cambridge Isotope Laboratories, Inc.	V104	1	9	47.37%	17.31%
IROA Technologies	V105	1	3	15.79%	5.77%
Merck/Sigma	V106	1	11	57.89%	21.15%
biocrates life science ag	V107	1	6	31.58%	11.54%
MetaSci	V108	1	3	15.79%	5.77%
Avanti polar lipids, Inc.	V118	1	12	63.16%	23.08%
Sciex	V119	1	1	5.26%	1.92%
Metrological institutes (e.g. JRC, NIST)	V120	1	1	5.26%	1.92%
Other/further/comments	V132	1	6	31.58%	11.54%
**Total**			52 Answers	19 Participants	

Question 21 - Commercially available standard mixtures

Status: July 18, 2024, 13:37, Survey: "DGMet-Survey"

Number of participants evaluated: 29 (all participants)

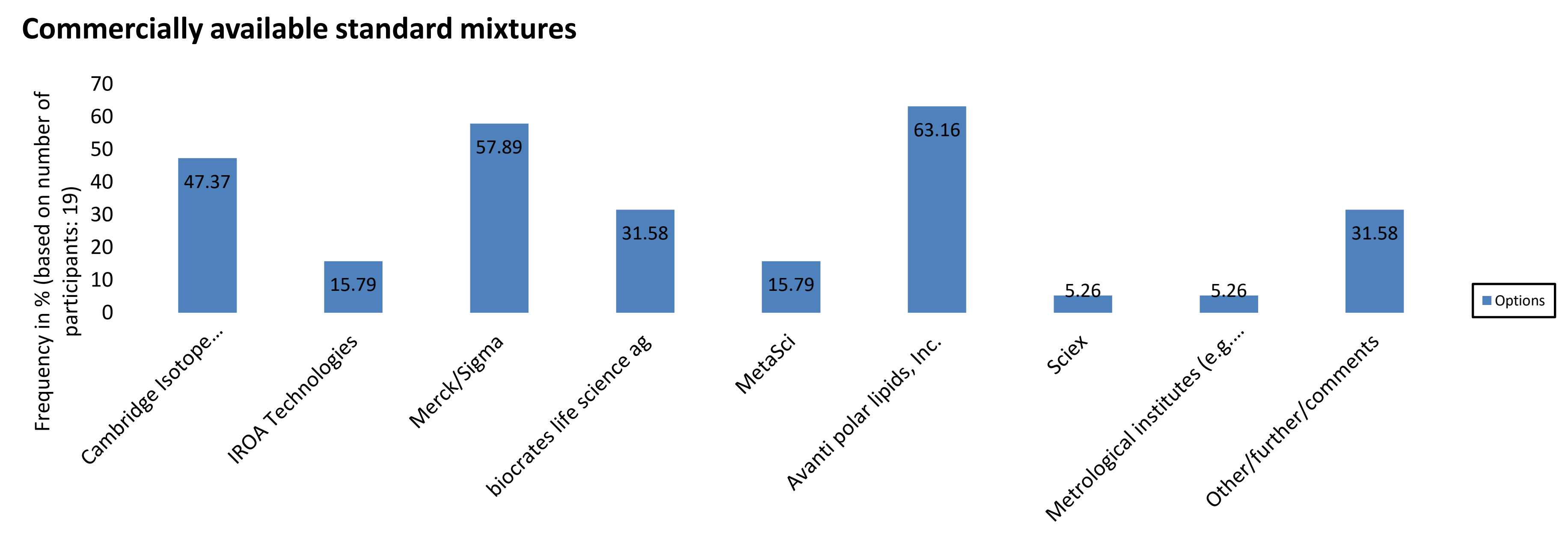

Question 21 - Commercially available standard mixtures

Status: July 18, 2024, 13:37, Survey: "DGMet-Survey"

Number of participants evaluated: 29 (all participants)

Detailed results for entry field of Other/further/comments

Number Answers	6	Number unique	6

Value/Answer	Number	Frequency
Instrument vendor mixes	1	16.67%
Agilent Technologies	1	16.67%
Cayman Chemicals	1	16.67%
Bruker	1	16.67%
TRC	1	16.67%
Apel Environmental	1	16.67%
**Total**	**6**	**100%**

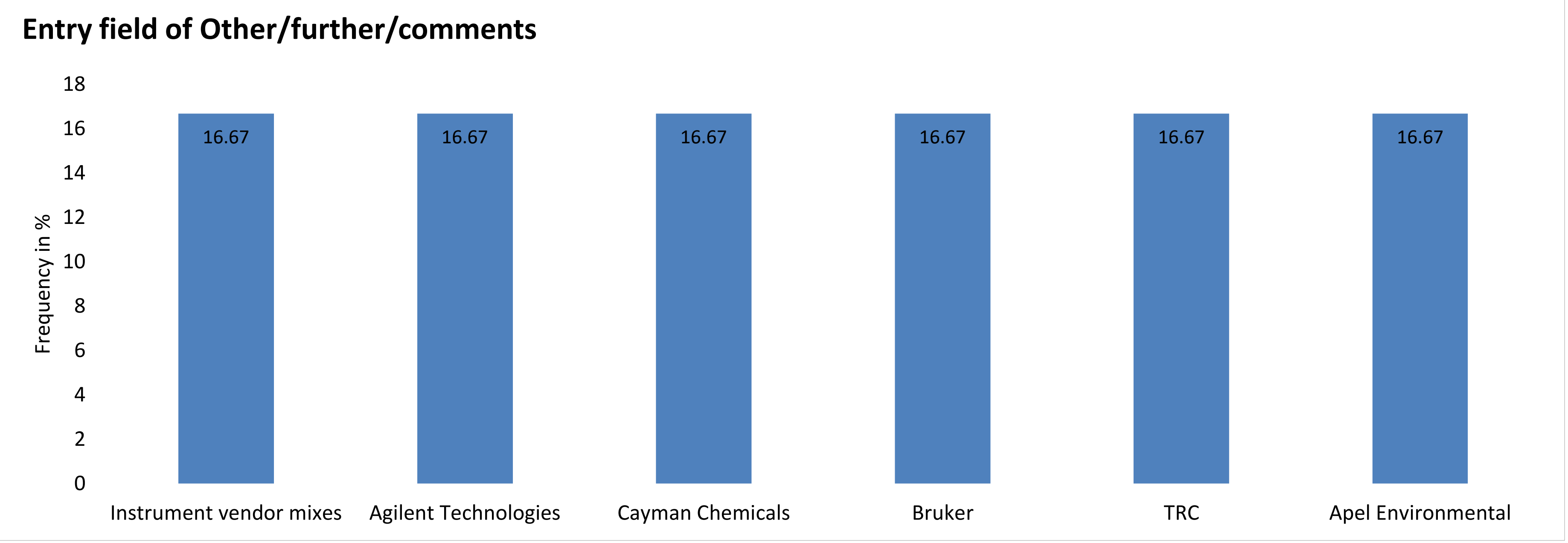

Created with LamaPoll | https://www.lamapoll.de

Question 22 - Commercially available compound mixtures

Status: July 18, 2024, 13:37, Survey: "DGMet-Survey"

Number of participants evaluated: 29 (all participants)

Status data

of 29 participants	Number	Percent
Question seen	19	65.52%
Question answered	8	27.59%
Question not answered	21	72.41%

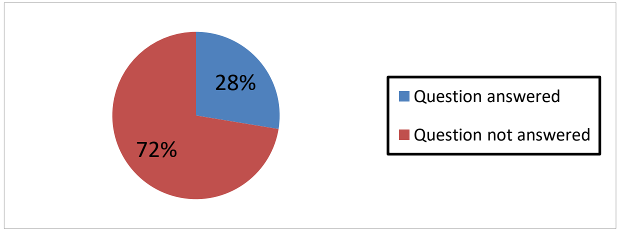

Detailed results for entry field of Commercially available compound mixtures - Row 1 / Column 1

Variable	V133.C1		
Number Answers	8	Number unique	7

Value/Answer	Number	Frequency
Cer/sph mixture 1	1	12.50%
Cer/sph mixture i , avantis	1	12.50%
Instrument vendor calibration mixes	1	12.50%
Msmls	1	12.50%
Ribitol	1	12.50%
Splash	2	25%
Ultimate splash i	1	12.50%
**Total**	**8**	**100%**

Question 22 - Commercially available compound mixtures

Status: July 18, 2024, 13:37, Survey: "DGMet-Survey"

Number of participants evaluated: 29 (all participants)

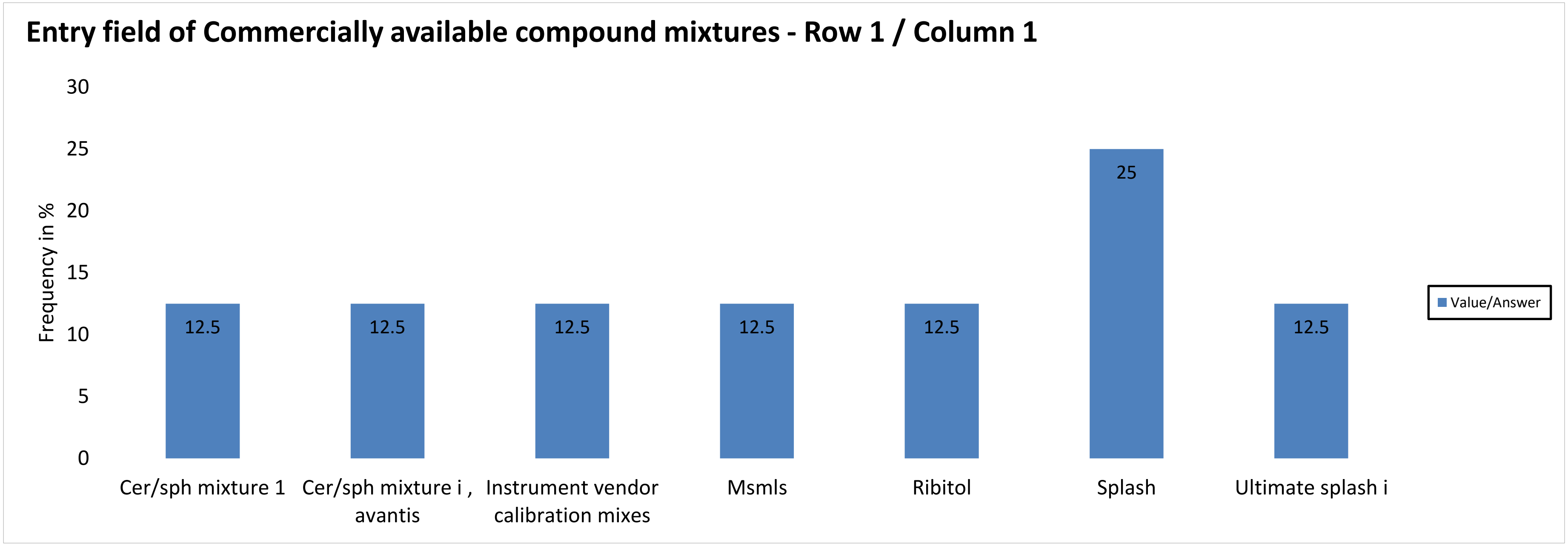

Question 22 - Commercially available compound mixtures

Status: July 18, 2024, 13:37, Survey: "DGMet-Survey"

Number of participants evaluated: 29 (all participants)

Detailed results for entry field of Commercially available compound mixtures - Row 2 / Column 1

Variable	V134.C1		
Number Answers	7	Number unique	7

Value/Answer	Number	Frequency
Biocrates standards	1	14.29%
Carbohydrates mix (sigma)	1	14.29%
Equisplash	1	14.29%
Mixture of 16 labeled aa	1	14.29%
Mycolic acid from m. tuberculosis	1	14.29%
Qress	1	14.29%
Splash lipidomix	1	14.29%
**Total**	**7**	**100%**

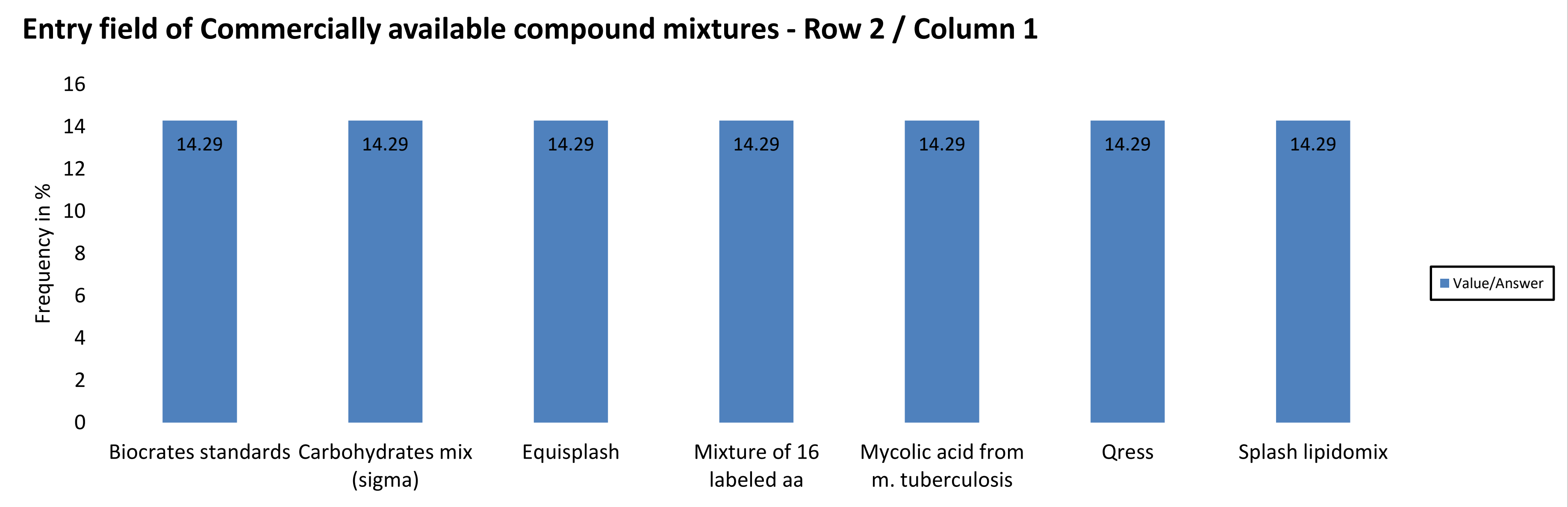

Question 22 - Commercially available compound mixtures

Status: July 18, 2024, 13:37, Survey: "DGMet-Survey"

Number of participants evaluated: 29 (all participants)

Detailed results for entry field of Commercially available compound mixtures - Row 3 / Column 1

Variable	V135.C1		
Number Answers	6	Number unique	6

Value/Answer	Number	Frequency
Acylcarnitines	1	16.67%
Amino acids mix (sigma)	1	16.67%
Cardiolipin mixture i	1	16.67%
Gc standard for sherlock microbial identification system	1	16.67%
Iroa	1	16.67%
Thermo pierce™ ltq velos esi positive	1	16.67%
**Total**	**6**	**100%**

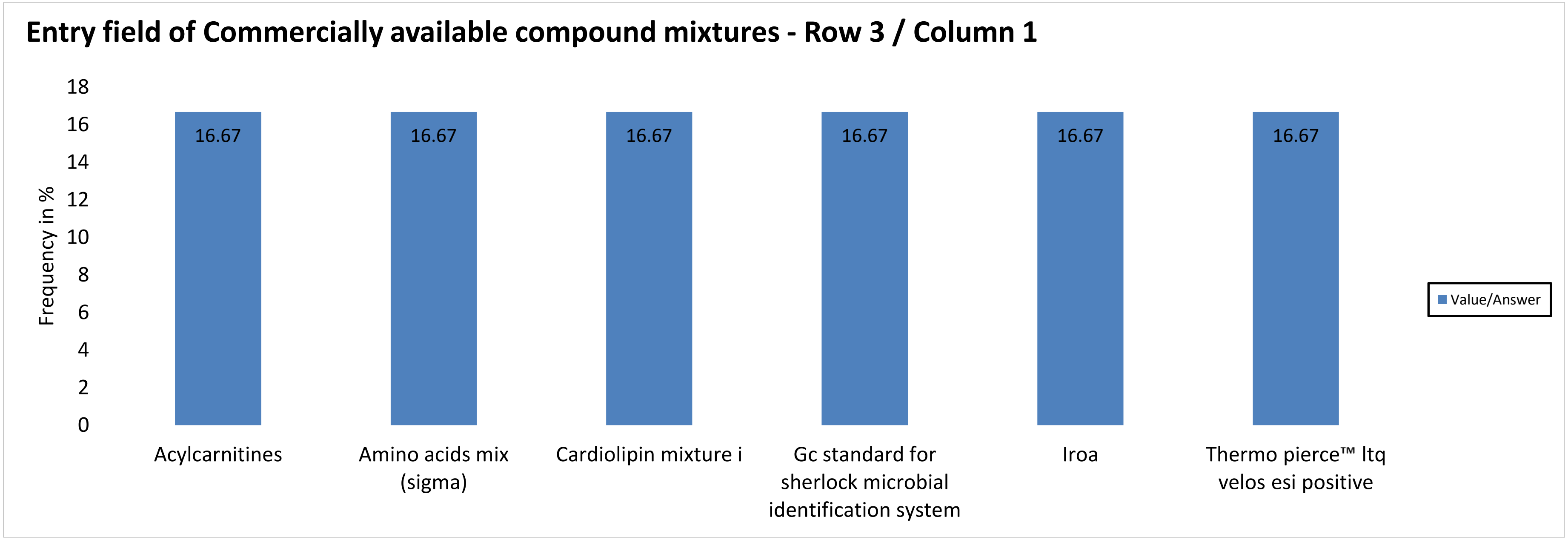

Question 22 - Commercially available compound mixtures

Status: July 18, 2024, 13:37, Survey: "DGMet-Survey"

Number of participants evaluated: 29 (all participants)

Detailed results for entry field of Commercially available compound mixtures - Row 4 / Column 1

Variable	V136.C1		
Number Answers	3	Number unique	3

Value/Answer	Number	Frequency
Fatty acid mix (sigma)	1	33.33%
Supelco 37 component fame mix	1	33.33%
Thermo pierce™ ltq velos esi negative	1	33.33%
**Total**	**3**	**100%**

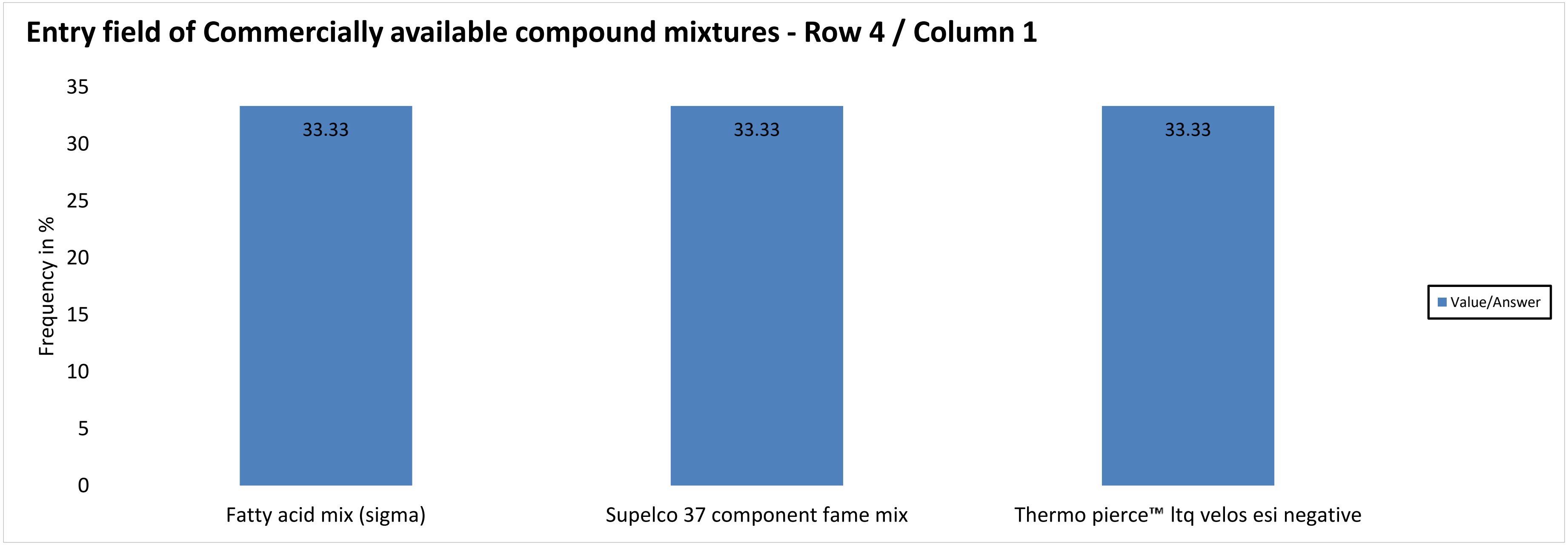

Question 22 - Commercially available compound mixtures

Status: July 18, 2024, 13:37, Survey: "DGMet-Survey"

Number of participants evaluated: 29 (all participants)

Detailed results for entry field of Commercially available compound mixtures - Row 5 / Column 1

Variable	V137.C1		
Number Answers	2	Number unique	2

Value/Answer	Number	Frequency
Api-tof reference mass solution kit	1	50%
Pharma mix (neochema)	1	50%
**Total**	**2**	**100%**

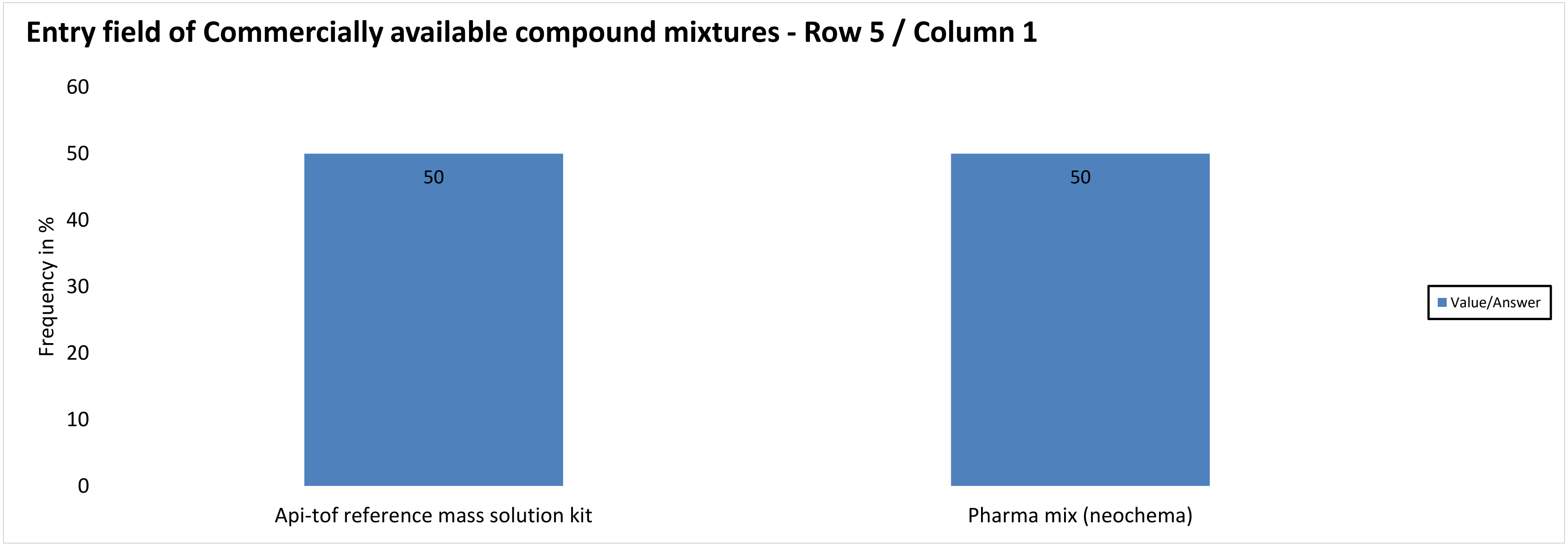

Question 22 - Commercially available compound mixtures

Status: July 18, 2024, 13:37, Survey: "DGMet-Survey"

Number of participants evaluated: 29 (all participants)

Detailed results for entry field of Commercially available compound mixtures - Row 6 / Column 1

Variable	V144.C1		
Number Answers	2	Number unique	2

Value/Answer	Number	Frequency
Cambridge metabolomics qc mix	1	50%
Equisplash™ lipidomix® quantitative mass spec internal standard	1	50%
**Total**	**2**	**100%**

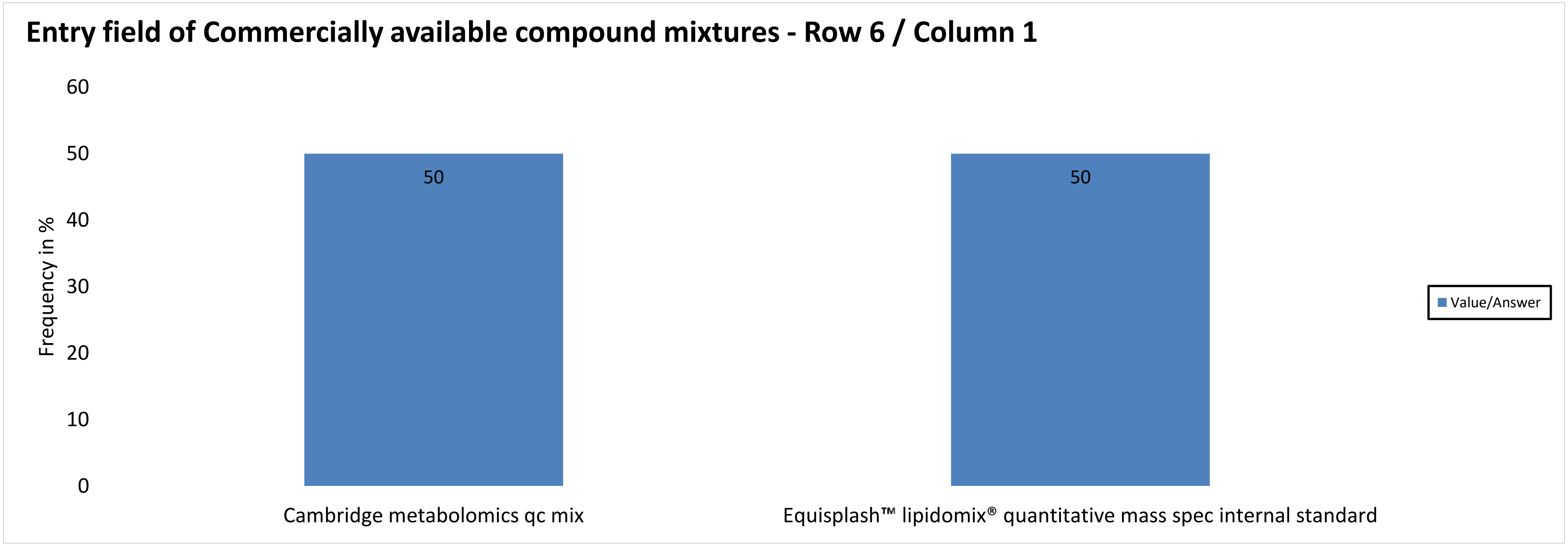

Question 22 - Commercially available compound mixtures

Status: July 18, 2024, 13:37, Survey: "DGMet-Survey"

Number of participants evaluated: 29 (all participants)

Results Isotope labelling (13C, 15N, ...) (Columns 2-3)

Frequency in %		Native	Labelled	Total
	Variable	C2	C5	
Row 1	V133	55.56%	44.44%	9
Row 2	V134	28.57%	71.43%	7
Row 3	V135	57.14%	42.86%	7
Row 4	V136	100%	0%	3
Row 5	V137	100%	0%	2
Row 6	V144	0%	100%	2

Number of answers		Native	Labelled	Total
	Variable	C2	C5	
Row 1	V133	5	4	9
Row 2	V134	2	5	7
Row 3	V135	4	3	7
Row 4	V136	3	0	3
Row 5	V137	2	0	2
Row 6	V144	0	2	2

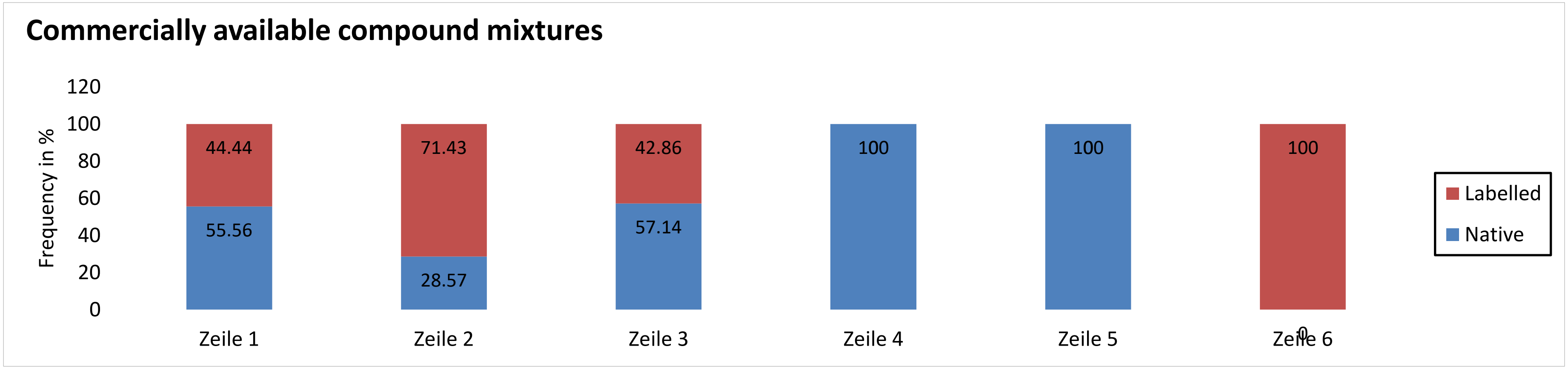

Question 23 - In-house prepared compound mixtures

Status: July 18, 2024, 13:37, Survey: "DGMet-Survey"

Number of participants evaluated: 29 (all participants)

Status data

of 29 participants	Number	Percent
Question seen	17	58.62%
Question answered	11	37.93%
Question not answered	18	62.07%

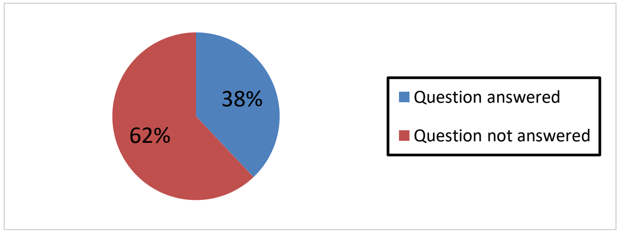

Detailed results for entry field of In-house prepared compound mixtures - Row 1 / Column 1

Variable	V109.C3		
Number Answers	10	Number unique	10

Value/Answer	Number	Frequency
"megamix" 80+ compounds for system suitability	1	10%
Adjusted mixtures of lipid standards	1	10%
Amino acids	1	10%
Bile acid mix	1	10%
Mixture of plant metabolites mm14	1	10%
Mm8 (rutin, iaa-valine, cinnamic acid,...)	1	10%
Oxylipin mix for calibration	1	10%
Primary metabolite mix	1	10%
Steroids mix	1	10%
Tryptophan metabolite mix	1	10%
**Total**	**10**	**100%**

Created with LamaPoll | https://www.lamapoll.de

Question 23 - In-house prepared compound mixtures

Status: July 18, 2024, 13:37, Survey: "DGMet-Survey"

Number of participants evaluated: 29 (all participants)

Entry field of In-house prepared compound mixtures - Row 1 / Column 1

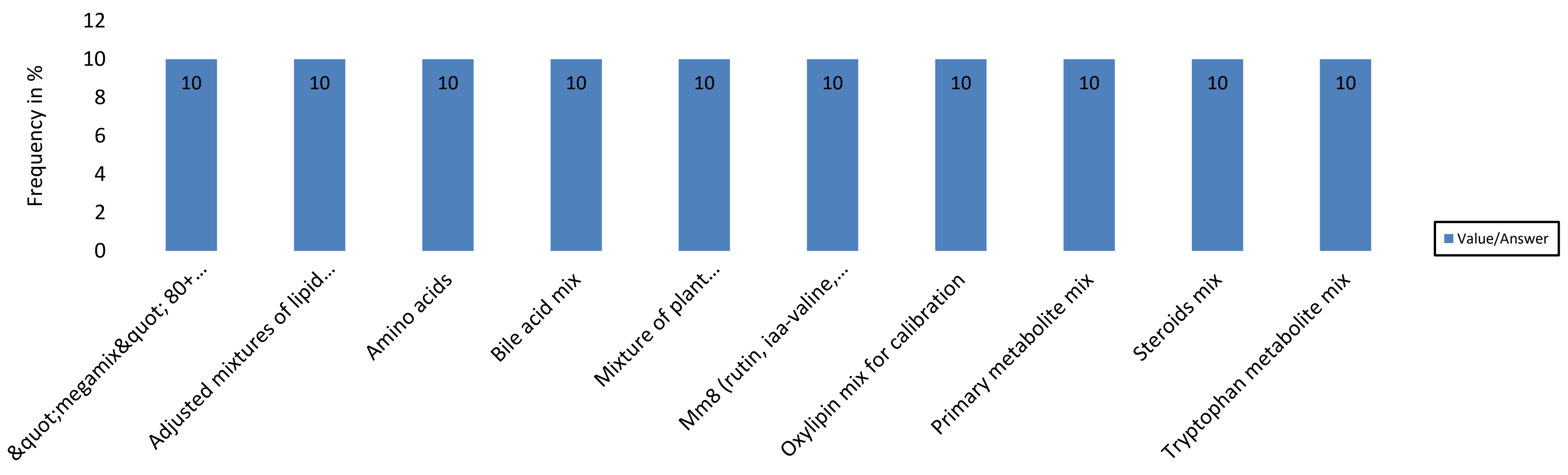

Question 23 - In-house prepared compound mixtures

Status: July 18, 2024, 13:37, Survey: "DGMet-Survey"

Number of participants evaluated: 29 (all participants)

Detailed results for entry field of In-house prepared compound mixtures - Row 2 / Column 1

Variable	V110.C3		
Number Answers	7	Number unique	7

Value/Answer	Number	Frequency
Acyl carnitines	1	14.29%
Antibiotics	1	14.29%
Esi tune mix	1	14.29%
Fatty acids mix	1	14.29%
Oxylipin mix internal standard mix	1	14.29%
Scfa mix	1	14.29%
Spike-in internal standard (6 compounds)	1	14.29%
**Total**	**7**	**100%**

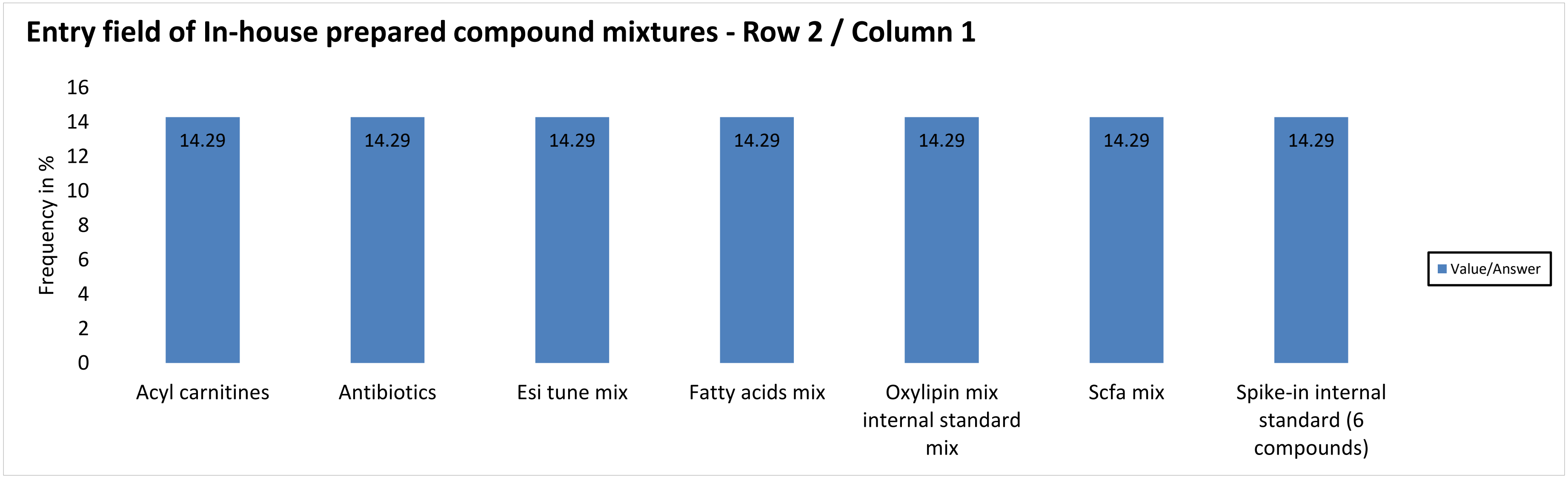

Question 23 - In-house prepared compound mixtures

Status: July 18, 2024, 13:37, Survey: "DGMet-Survey"

Number of participants evaluated: 29 (all participants)

Detailed results for entry field of In-house prepared compound mixtures - Row 3 / Column 1

Variable	V111.C3		
Number Answers	4	Number unique	4

Value/Answer	Number	Frequency
Chloramphenicol	1	25%
Extraction internal standard mix	1	25%
Scfa	1	25%
Short chain fatty acids mix	1	25%
Total	4	100%

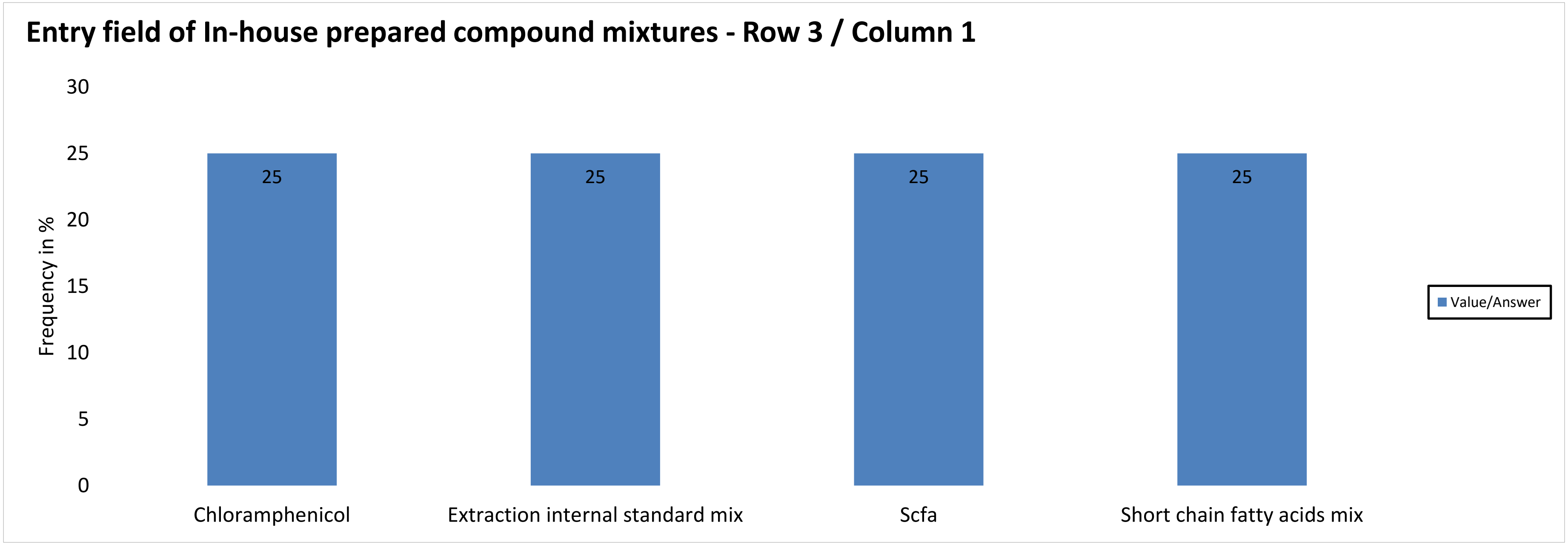

Question 23 - In-house prepared compound mixtures

Status: July 18, 2024, 13:37, Survey: "DGMet-Survey"

Number of participants evaluated: 29 (all participants)

Detailed results for entry field of In-house prepared compound mixtures - Row 4 / Column 1

Variable	V138.C3		
Number Answers	3	Number unique	3

Value/Answer	Number	Frequency
Bile acids	1	33.33%
Glutamine, uracil, arginine, proline, valine	1	33.33%
Recovery internal standard mix	1	33.33%
**Total**	**3**	**100%**

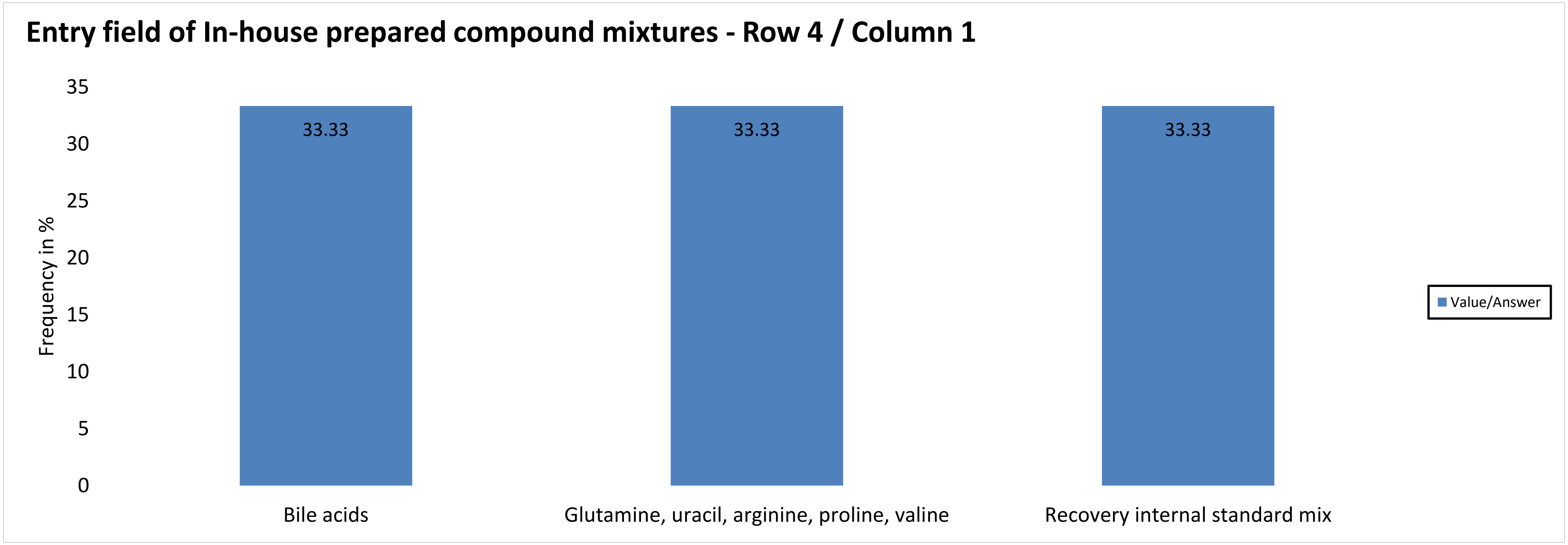

Question 23 - In-house prepared compound mixtures

Status: July 18, 2024, 13:37, Survey: "DGMet-Survey"

Number of participants evaluated: 29 (all participants)

Detailed results for entry field of In-house prepared compound mixtures - Row 5 / Column 1

Variable	V139.C3		
Number Answers	1	Number unique	1

Value/Answer	Number	Frequency
Nucelotides	1	100%
**Total**	**1**	**100%**

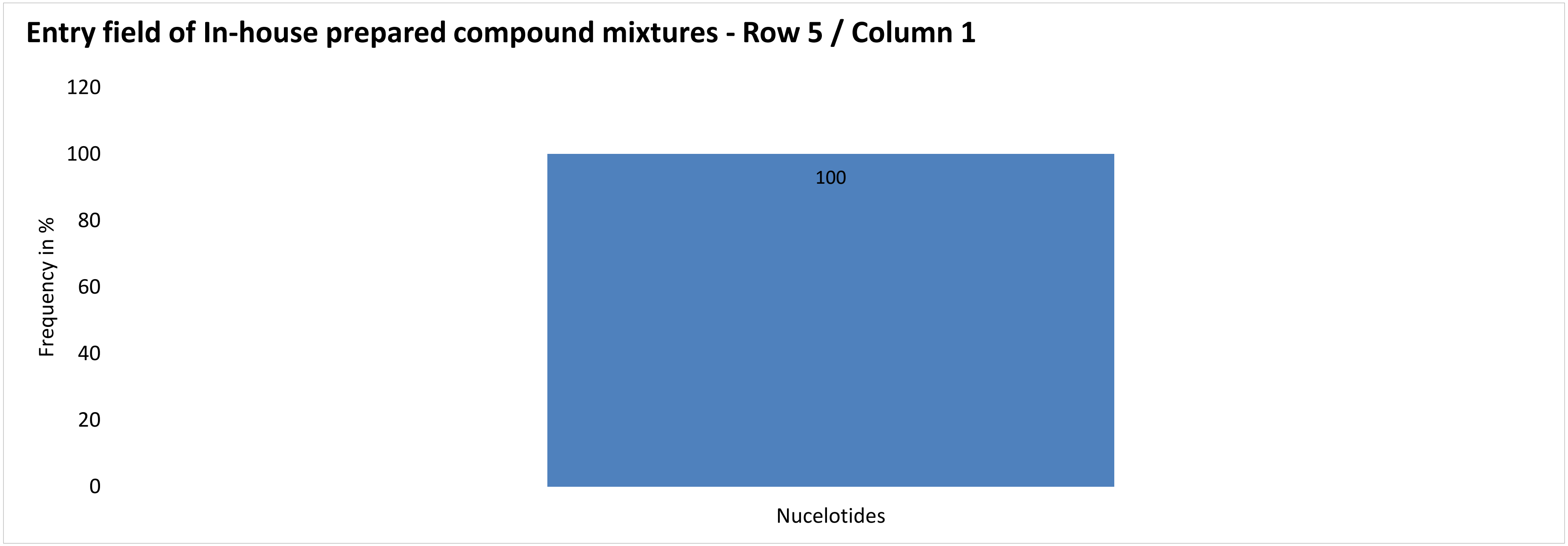

Question 23 - In-house prepared compound mixtures

Status: July 18, 2024, 13:37, Survey: "DGMet-Survey"

Number of participants evaluated: 29 (all participants)

Detailed results for entry field of In-house prepared compound mixtures - Row 6 / Column 1

Variable	V143.C3		
Number Answers	1	Number unique	1

Value/Answer	Number	Frequency
Fatty acids	1	100%
**Total**	**1**	**100%**

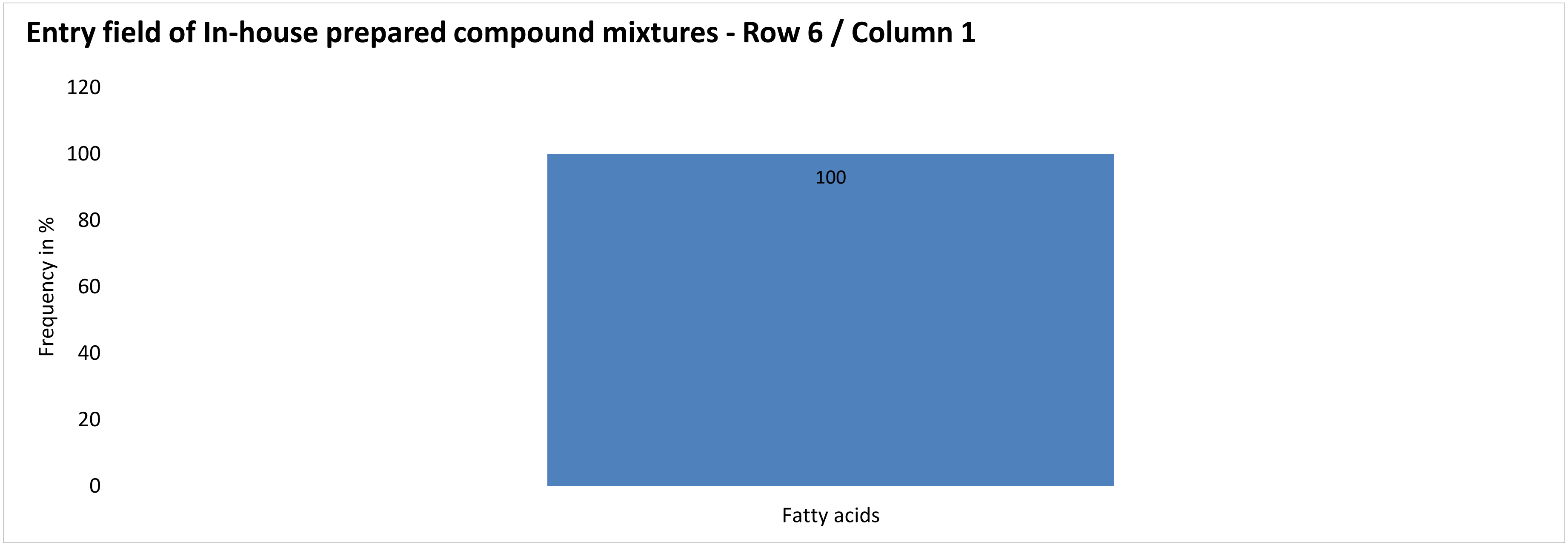

Question 23 - In-house prepared compound mixtures

Status: July 18, 2024, 13:37, Survey: "DGMet-Survey"

Number of participants evaluated: 29 (all participants)

Results Isotope labelling (13C, 15N, ...) (Columns 2-3)

Frequency in %		Native	Labelled	Total
	Variable	C4	C6	
Row 1	V109	75%	25%	12
Row 2	V110	60%	40%	10
Row 3	V111	60%	40%	5
Row 4	V138	25%	75%	4
Row 5	V139	50%	50%	2
Row 6	V143	50%	50%	2

Number of answers		Native	Labelled	Total
	Variable	C4	C6	
Row 1	V109	9	3	12
Row 2	V110	6	4	10
Row 3	V111	3	2	5
Row 4	V138	1	3	4
Row 5	V139	1	1	2
Row 6	V143	1	1	2

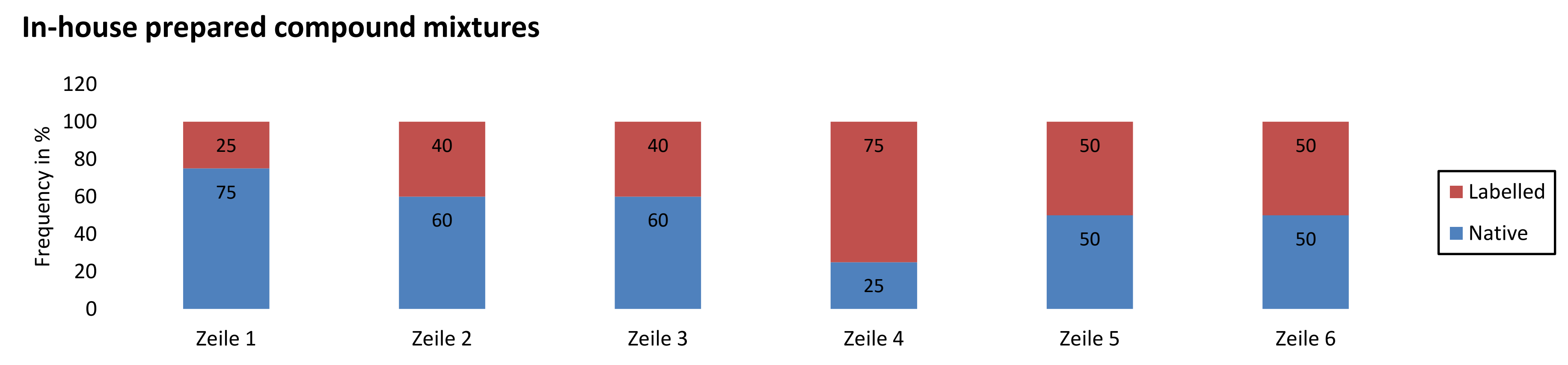

Question 24 - Do you use custom synthesis of standard compounds?

Status: July 18, 2024, 13:37, Survey: "DGMet-Survey"

Number of participants evaluated: 29 (all participants)

Status data

of 29 participants	Number	Percent
Question seen	23	79.31%
Question answered	20	68.97%
Question not answered	9	31.03%

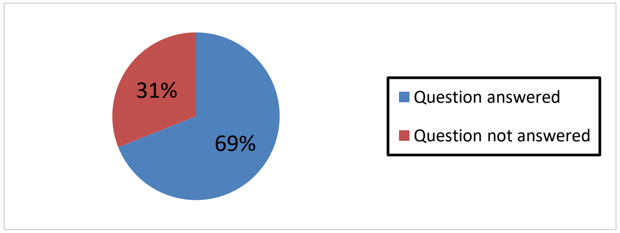

Results

Options	Variable	Code	Number	Frequency by participant	Frequency by answers
Yes, I have compounds synthesized by a commercial lab.	V149	1	4	20%	20%
Yes, I use the synthesis facility at my institute.	V150	1	1	5%	5%
No, I don't use custom chemical synthesis.	V151	1	15	75%	75%
**Total**			20 Answers	20 Participants	

Question 24 - Do you use custom synthesis of standard compounds?

Status: July 18, 2024, 13:37, Survey: "DGMet-Survey"

Number of participants evaluated: 29 (all participants)

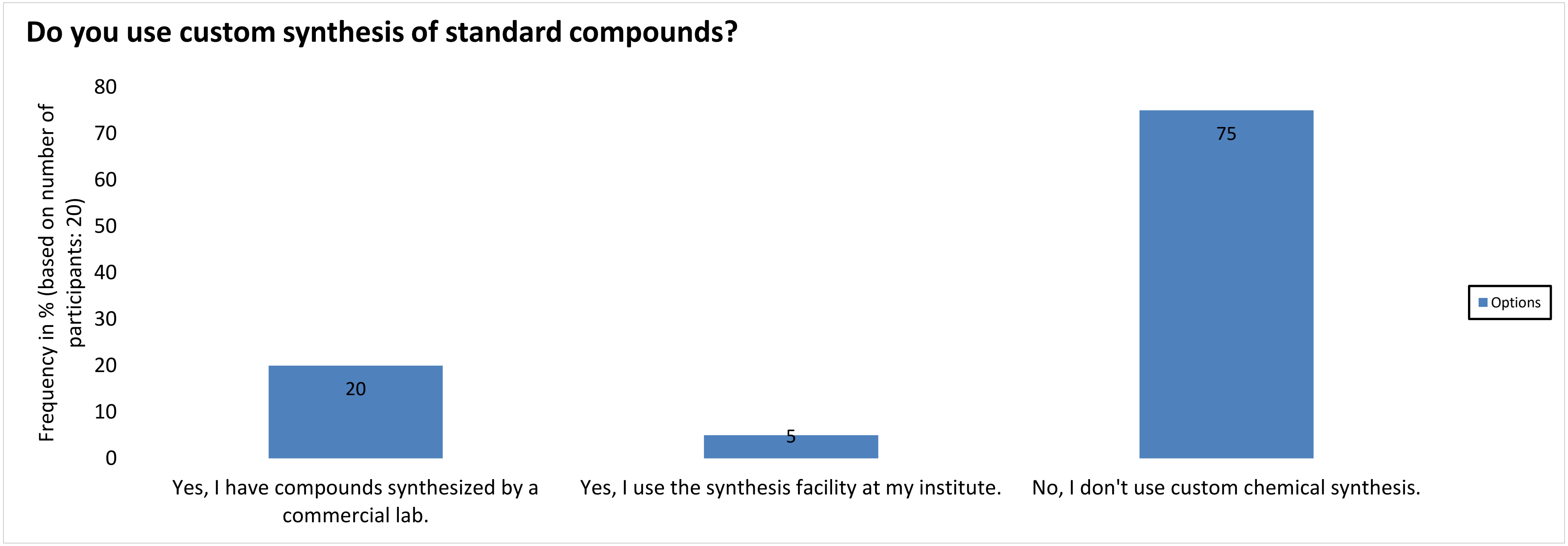

Created with LamaPoll | https://www.lamapoll.de

Question 25 - Use of matrix reference materials

Status: July 18, 2024, 13:37, Survey: "DGMet-Survey"

Number of participants evaluated: 29 (all participants)

Status data

of 29 participants	Number	Percent
Question seen	22	75.86%
Question answered	16	55.17%
Question not answered	13	44.83%

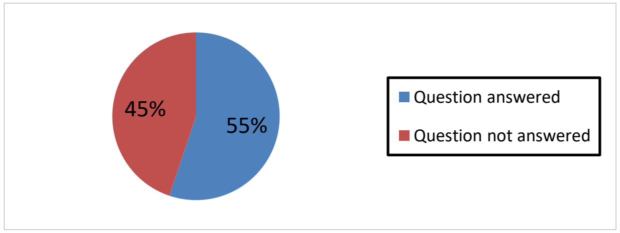

Results

Options	Variable	Code	Number	Frequency by participant	Frequency by answers
Instrument qualification (e.g. mass calibration)	V152	1	3	18.75%	5.45%
System suitability tests	V153	1	5	31.25%	9.09%
Quality control (QC) purposes	V154	1	12	75%	21.82%
Analytical method validation	V155	1	10	62.50%	18.18%
Calibration standards for quantification	V156	1	4	25%	7.27%
Metabolite identification	V157	1	6	37.50%	10.91%
Bridging across study sample data	V158	1	6	37.50%	10.91%
Data pre-processing (e.g. normalization)	V159	1	8	50%	14.55%
Other/further/comments	V160	1	1	6.25%	1.82%
**Total**			55 Answers	16 Participants	

Created with LamaPoll | https://www.lamapoll.de

Question 25 - Use of matrix reference materials

Status: July 18, 2024, 13:37, Survey: "DGMet-Survey"

Number of participants evaluated: 29 (all participants)

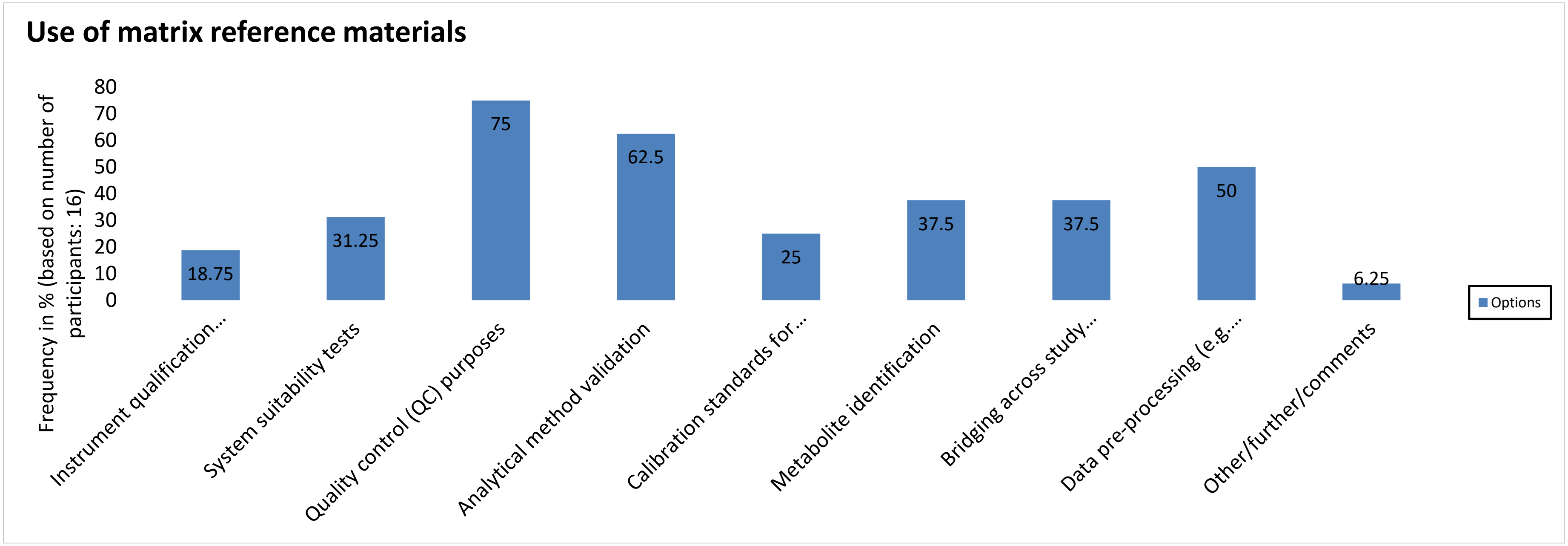

Question 25 - Use of matrix reference materials

Status: July 18, 2024, 13:37, Survey: "DGMet-Survey"

Number of participants evaluated: 29 (all participants)

Detailed results for entry field of Other/further/comments

Number Answers	1	Number unique	0

Value/Answer	Number	Frequency

Question 26 - Reference materials used in your lab

Status: July 18, 2024, 13:37, Survey: "DGMet-Survey"

Number of participants evaluated: 29 (all participants)

Status data

of 29 participants	Number	Percent
Question seen	22	75.86%
Question answered	16	55.17%
Question not answered	13	44.83%

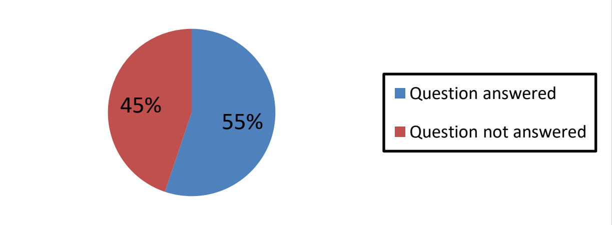

Results

Options	Variable	Code	Number	Frequency by participant	Frequency by answers
Certified reference material (CRM, e.g. NIST SRM 1950)	V29	1	7	43.75%	29.17%
Non-certified reference material (including 'research grade testing materials')	V36	1	5	31.25%	20.83%
In-house prepared reference material	V37	1	12	75%	50%
**Total**			24 Answers	16 Participants	

Question 26 - Reference materials used in your lab

Status: July 18, 2024, 13:37, Survey: "DGMet-Survey"

Number of participants evaluated: 29 (all participants)

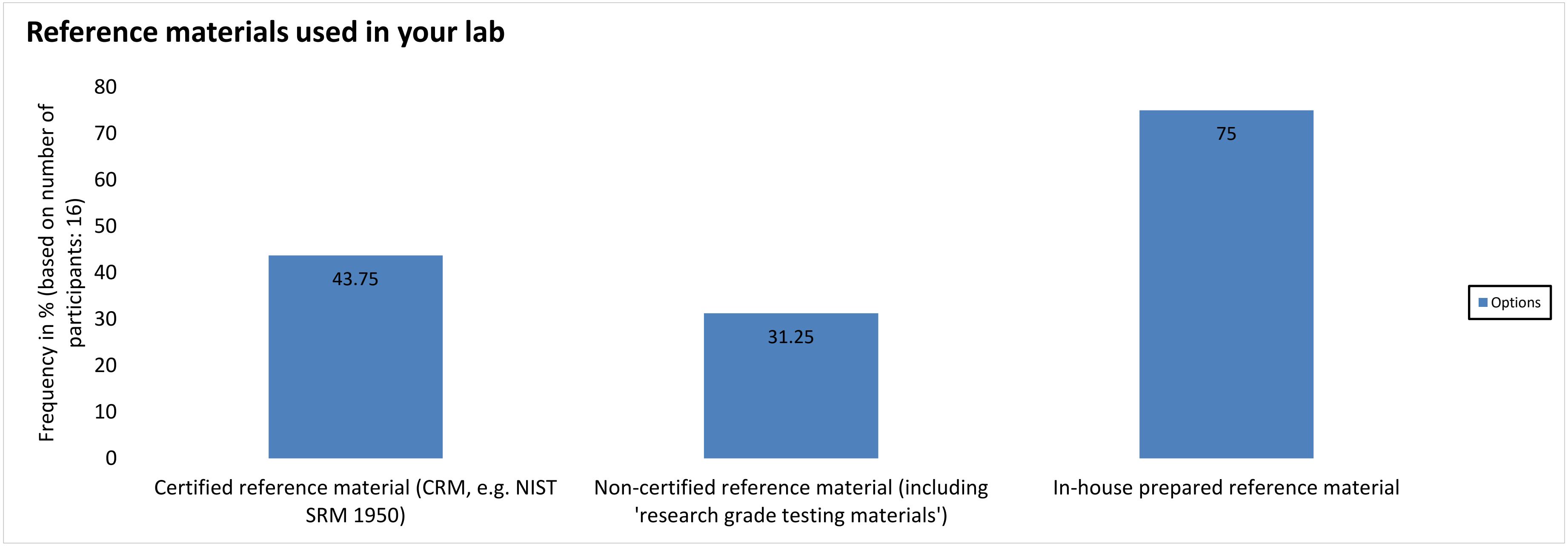

Created with LamaPoll | https://www.lamapoll.de

Question 27 - Certified reference materials used in your lab

Status: July 18, 2024, 13:37, Survey: "DGMet-Survey"

Number of participants evaluated: 29 (all participants)

Status data

of 29 participants	Number	Percent
Question seen	7	24.14%
Question answered	7	24.14%
Question not answered	22	75.86%

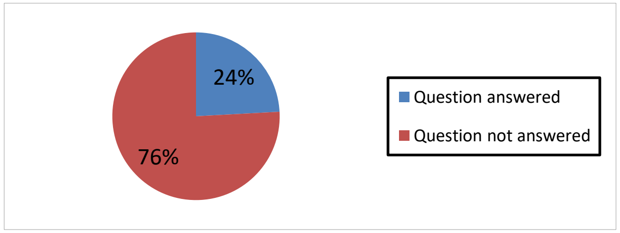

Results

Options	Variable	Code	Number	Frequency by participant	Frequency by answers
NIST SRM1950 human plasma pool	V112	1	7	100%	87.50%
Other/further/comments	V113	1	1	14.29%	12.50%
**Total**			8 Answers	7 Participants	

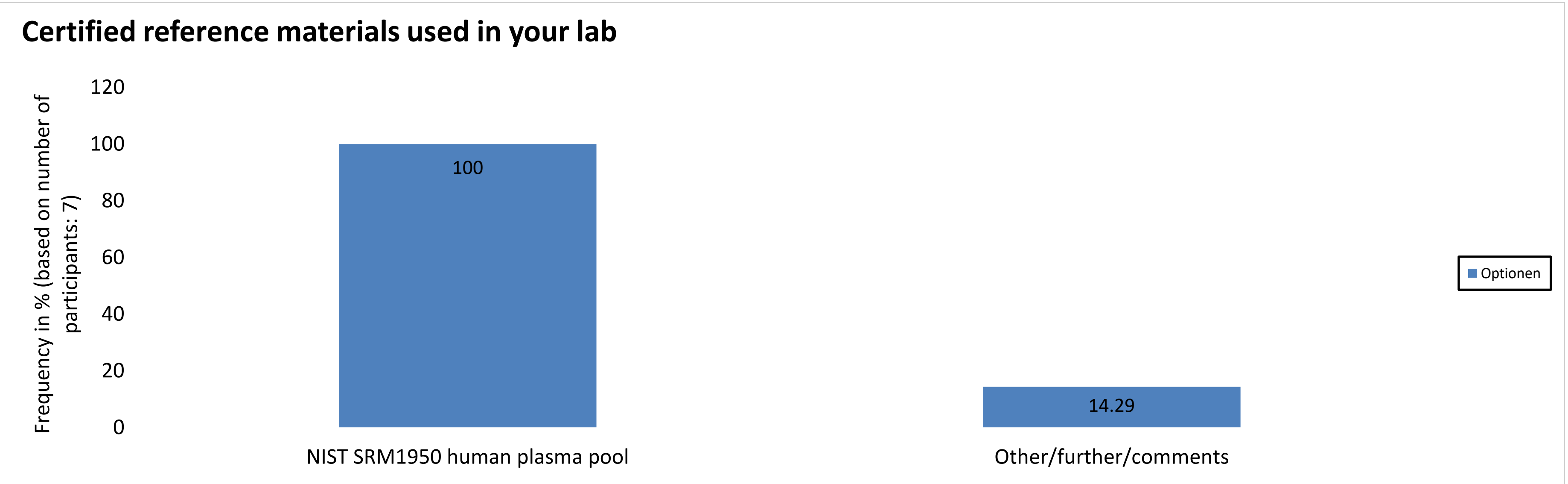

Question 27 - Certified reference materials used in your lab

Status: July 18, 2024, 13:37, Survey: "DGMet-Survey"

Number of participants evaluated: 29 (all participants)

Detailed results for entry field of Other/further/comments

Number Answers	1	Number unique	1

Value/Answer	Number	Frequency
Non-clinical RMs	1	100%
**Total**	**1**	**100%**

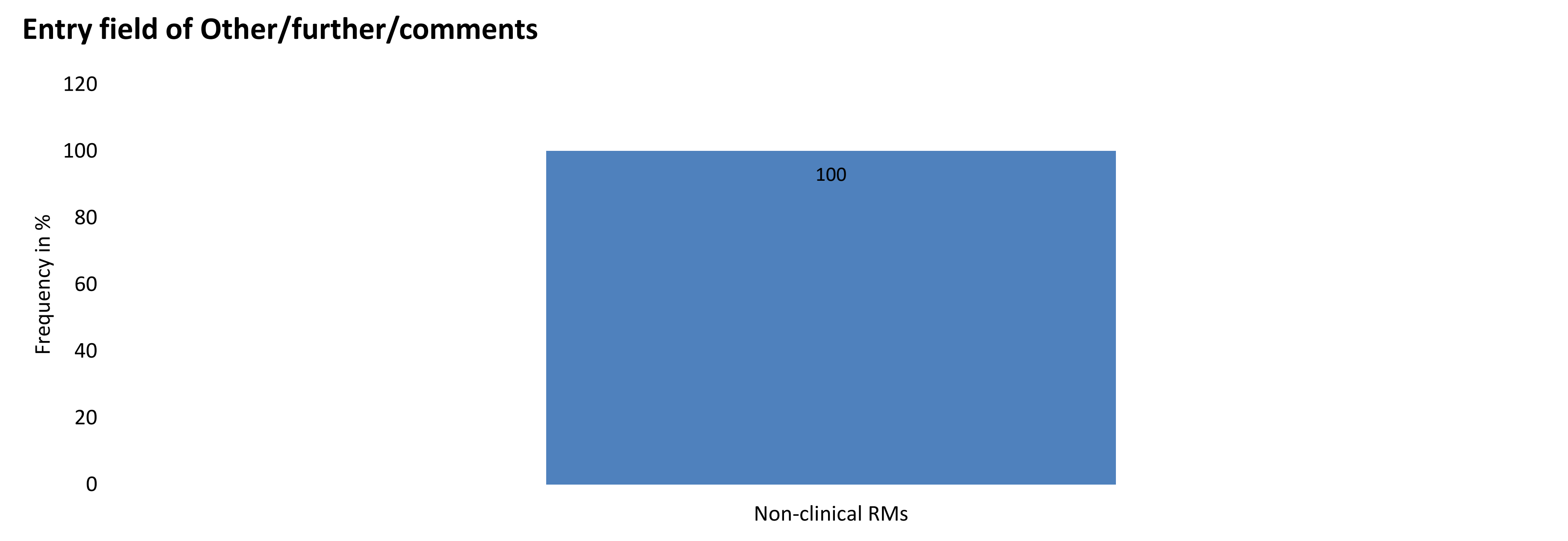

Question 28 - Certified reference materials used in your lab - applications

Status: July 18, 2024, 13:37, Survey: "DGMet-Survey"

Number of participants evaluated: 29 (all participants)

Status data

of 29 participants	Number	Percent
Question seen	7	24.14%
Question answered	6	20.69%
Question not answered	23	79.31%

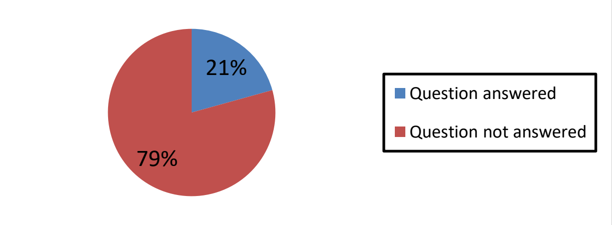

Results

Options	Variable	Code	Number	Frequency by participant	Frequency by answers
Quantification of measurands that CRM has been certified for	V164	1	2	33.33%	16.67%
Quantification of measurands that CRM has not been certified for	V167	1	1	16.67%	8.33%
General method quality control	V168	1	6	100%	50%
Comparability within or between labs	V170	1	3	50%	25%
Other/further	V199	1	0	0%	0%
**Total**			12 Answers	6 Participants	

Question 28 - Certified reference materials used in your lab - applications

Status: July 18, 2024, 13:37, Survey: "DGMet-Survey"

Number of participants evaluated: 29 (all participants)

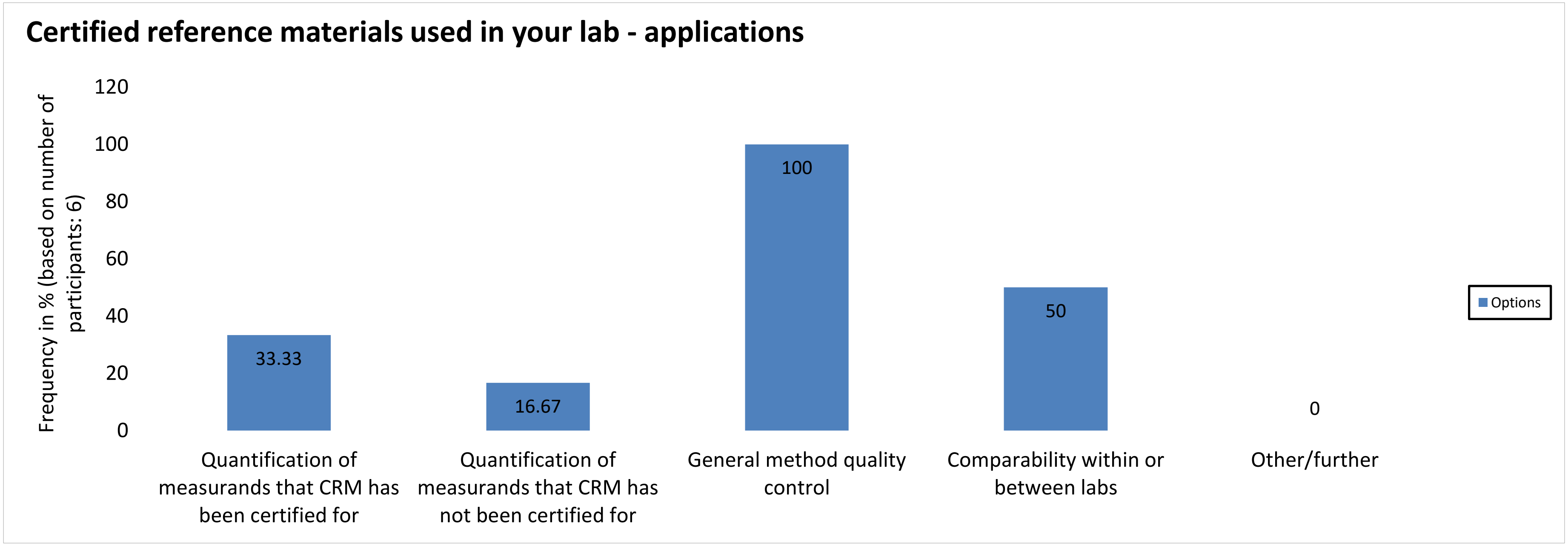

Question 28 - Certified reference materials used in your lab - applications

Status: July 18, 2024, 13:37, Survey: "DGMet-Survey"

Number of participants evaluated: 29 (all participants)

Detailed results for entry field of Other/further

Number Answers	0	Number unique	0

Value/Answer	Number	Frequency

Question 29 - Non-certified reference materials (including 'research grade testing materials')

Status: July 18, 2024, 13:37, Survey: "DGMet-Survey"

Number of participants evaluated: 29 (all participants)

Status data

of 29 participants	Number	Percent
Question seen	5	17.24%
Question answered	3	10.34%
Question not answered	26	89.66%

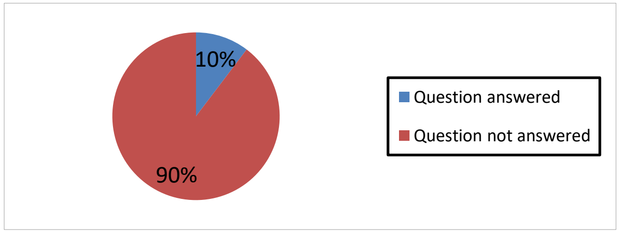

Detailed results for entry field of Non-certified reference materials (including 'research grade testing materials') - Row 1 / Name

Variable	V122.C7		
Number Answers	3	Number unique	3

Value/Answer	Number	Frequency
Iso-1 (isotopic solutions, vienna)	1	33.33%
Reference plasma	1	33.33%
Sigma citrat plasma	1	33.33%
**Total**	**3**	**100%**

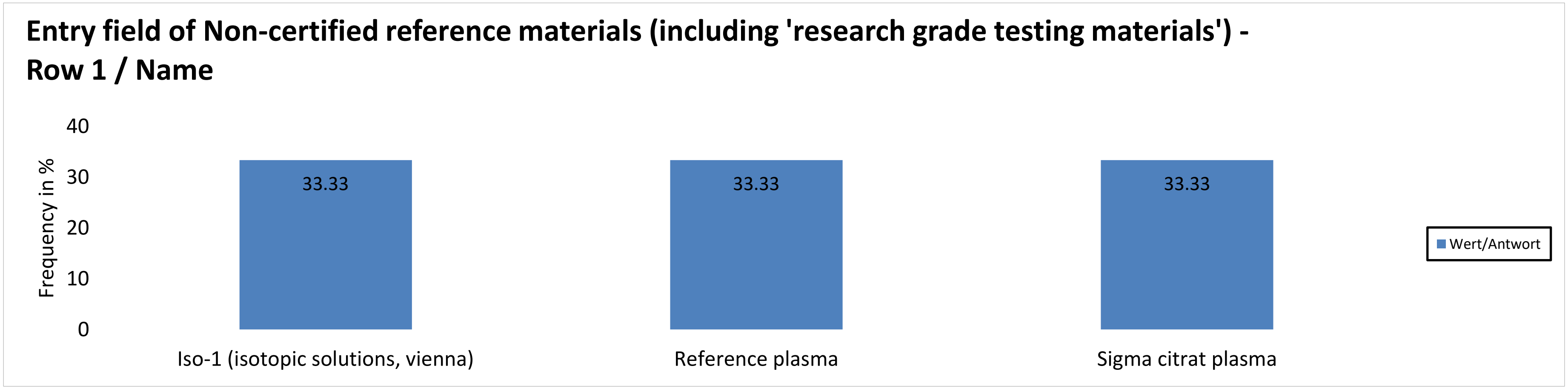

Question 29 - Non-certified reference materials (including 'research grade testing materials')

Status: July 18, 2024, 13:37, Survey: "DGMet-Survey"

Number of participants evaluated: 29 (all participants)

Detailed results for entry field of Non-certified reference materials (including 'research grade testing materials') - Row 1 / Species/Matrix

Variable	V122.C8		
Number Answers	3	Number unique	3

Value/Answer	Number	Frequency
Human	1	33.33%
Human plasma	1	33.33%
U13c yeast extract	1	33.33%
**Total**	**3**	**100%**

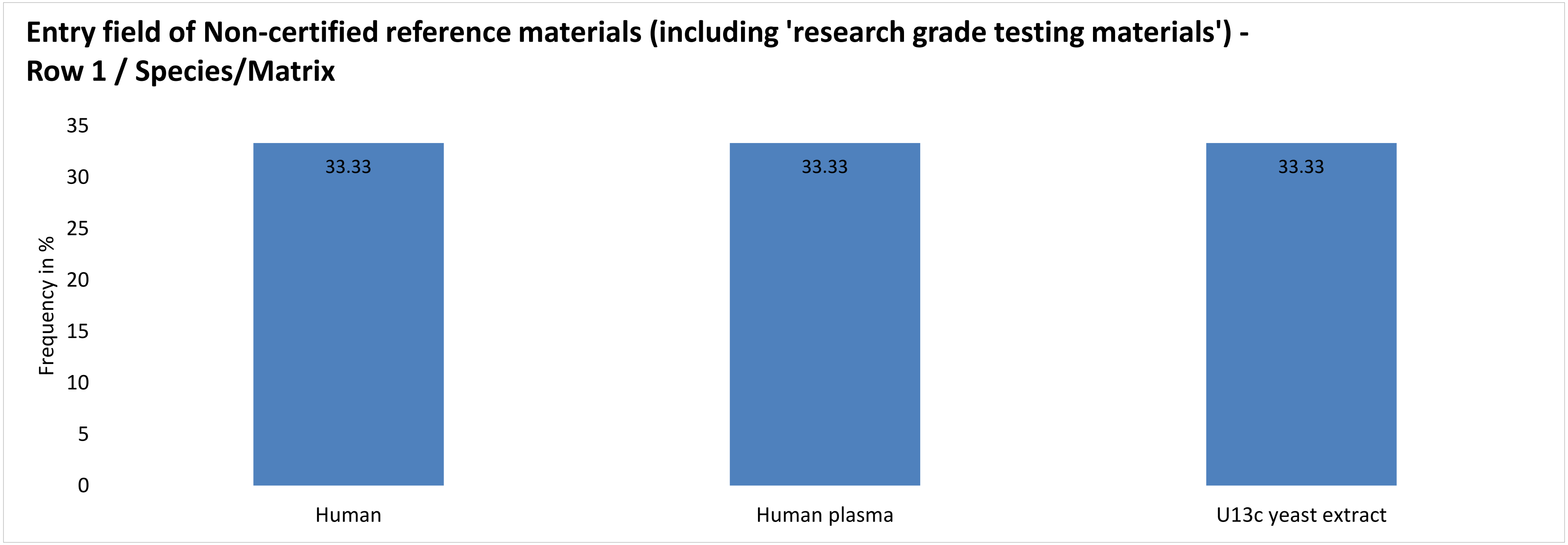

Question 29 - Non-certified reference materials (including 'research grade testing materials')

Status: July 18, 2024, 13:37, Survey: "DGMet-Survey"

Number of participants evaluated: 29 (all participants)

Detailed results for entry field of Non-certified reference materials (including 'research grade testing materials') - Row 2 / Name

Variable	V161.C7		
Number Answers	1	Number unique	1

Value/Answer	Number	Frequency
Reference plasma	1	100%
**Total**	**1**	**100%**

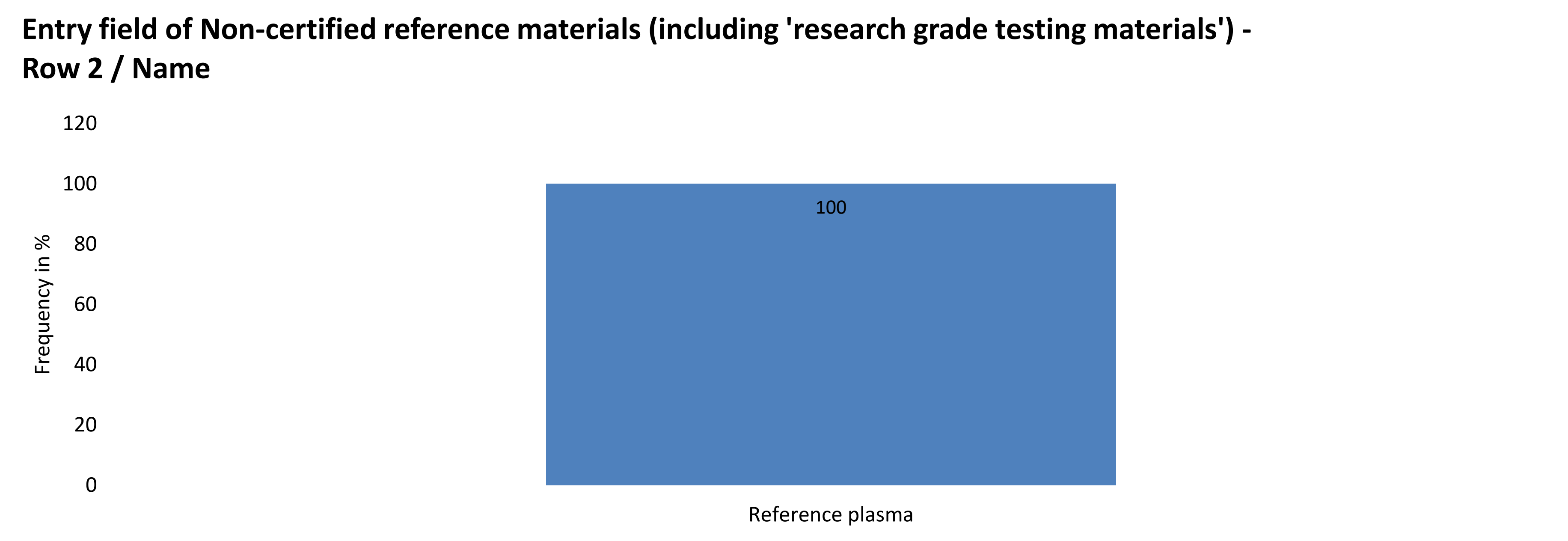

Question 29 - Non-certified reference materials (including 'research grade testing materials')

Status: July 18, 2024, 13:37, Survey: "DGMet-Survey"

Number of participants evaluated: 29 (all participants)

Detailed results for entry field of Non-certified reference materials (including 'research grade testing materials') - Row 2 / Species/Matrix

Variable	V161.C8		
Number Answers	1	Number unique	1

Value/Answer	Number	Frequency
Mouse	1	100%
**Total**	**1**	**100%**

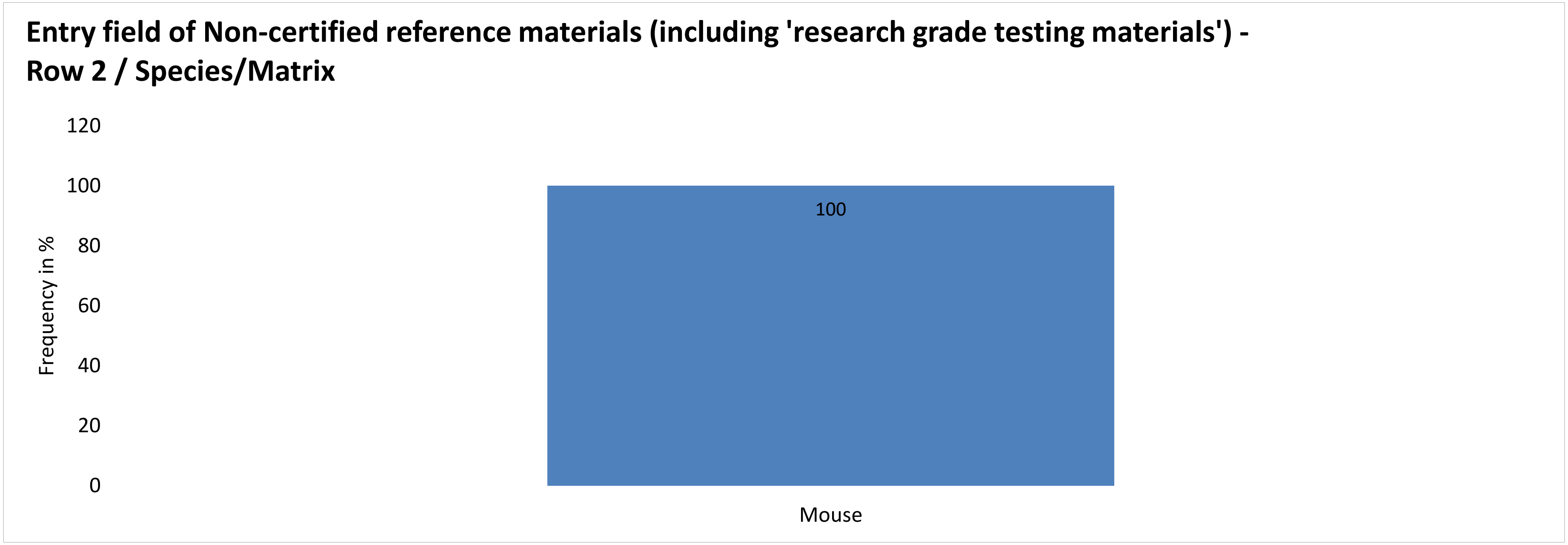

Question 29 - Non-certified reference materials (including 'research grade testing materials')

Status: July 18, 2024, 13:37, Survey: "DGMet-Survey"

Number of participants evaluated: 29 (all participants)

Detailed results for entry field of Non-certified reference materials (including 'research grade testing materials') - Row 3 / Name

Variable	V162.C7		
Number Answers	0	Number unique	0

Value/Answer	Number	Frequency

Question 29 - Non-certified reference materials (including 'research grade testing materials')

Status: July 18, 2024, 13:37, Survey: "DGMet-Survey"

Number of participants evaluated: 29 (all participants)

Detailed results for entry field of Non-certified reference materials (including 'research grade testing materials') - Row 3 / Species/Matrix

Variable	V162.C8		
Number Answers	0	Number unique	0

Value/Answer	Number	Frequency

Question 29 - Non-certified reference materials (including 'research grade testing materials')

Status: July 18, 2024, 13:37, Survey: "DGMet-Survey"

Number of participants evaluated: 29 (all participants)

Detailed results for entry field of Non-certified reference materials (including 'research grade testing materials') - Row 4 / Name

Variable	V163.C7		
Number Answers	0	Number unique	0

Value/Answer	Number	Frequency

Question 29 - Non-certified reference materials (including 'research grade testing materials')

Status: July 18, 2024, 13:37, Survey: "DGMet-Survey"

Number of participants evaluated: 29 (all participants)

Detailed results for entry field of Non-certified reference materials (including 'research grade testing materials') - Row 4 / Species/Matrix

Variable	V163.C8		
Number Answers	0	Number unique	0

Value/Answer	Number	Frequency

Question 29 - Non-certified reference materials (including 'research grade testing materials')

Status: July 18, 2024, 13:37, Survey: "DGMet-Survey"

Number of participants evaluated: 29 (all participants)

Detailed results for entry field of Non-certified reference materials (including 'research grade testing materials') - Row 5 / Name

Variable	V165.C7		
Number Answers	0	Number unique	0

Value/Answer	Number	Frequency

Created with LamaPoll | https://www.lamapoll.de

Question 29 - Non-certified reference materials (including 'research grade testing materials')

Status: July 18, 2024, 13:37, Survey: "DGMet-Survey"

Number of participants evaluated: 29 (all participants)

Detailed results for entry field of Non-certified reference materials (including 'research grade testing materials') - Row 5 / Species/Matrix

Variable	V165.C8		
Number Answers	0	Number unique	0

Value/Answer	Number	Frequency

Question 29 - Non-certified reference materials (including 'research grade testing materials')

Status: July 18, 2024, 13:37, Survey: "DGMet-Survey"

Number of participants evaluated: 29 (all participants)

Results Isotope labelling (13C, 15N, ...) (Columns 3-4)

Frequency in %		Native	Labelled	Total
	Variable	C9	C10	
Row 1	V122	66.67%	33.33%	3
Row 2	V161	100%	0%	1
Row 3	V162	0%	0%	0
Row 4	V163	0%	0%	0
Row 5	V165	0%	0%	0

Number of answers		Native	Labelled	Total
	Variable	C9	C10	
Row 1	V122	2	1	3
Row 2	V161	1	0	1
Row 3	V162	0	0	0
Row 4	V163	0	0	0
Row 5	V165	0	0	0

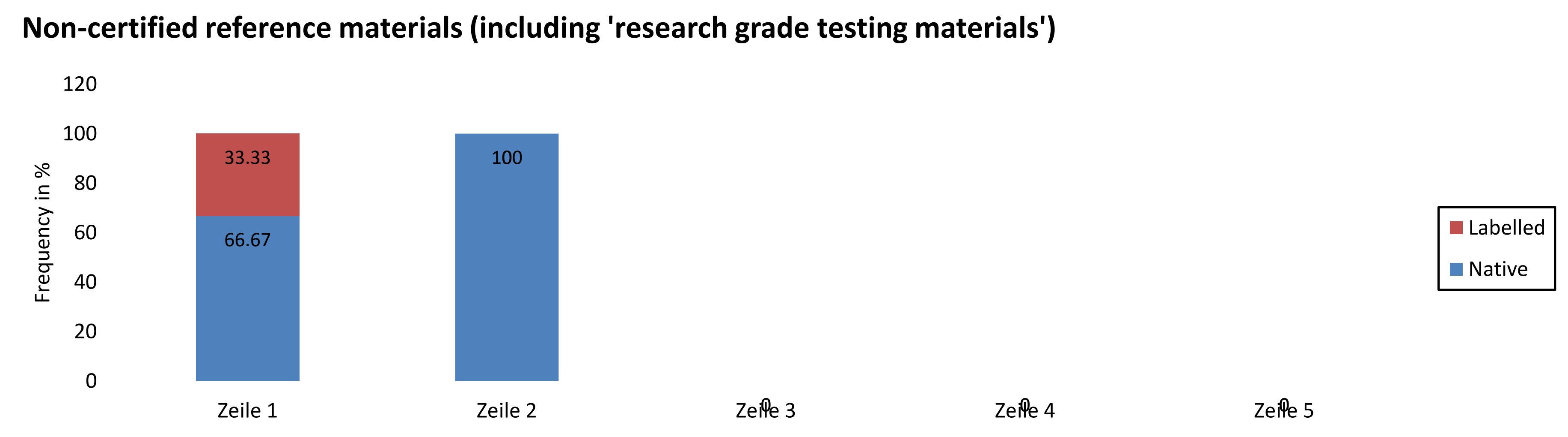

Question 30 - In-house prepared reference materials

Status: July 18, 2024, 13:37, Survey: "DGMet-Survey"

Number of participants evaluated: 29 (all participants)

Status data

of 29 participants	Number	Percent
Question seen	12	41.38%
Question answered	5	17.24%
Question not answered	24	82.76%

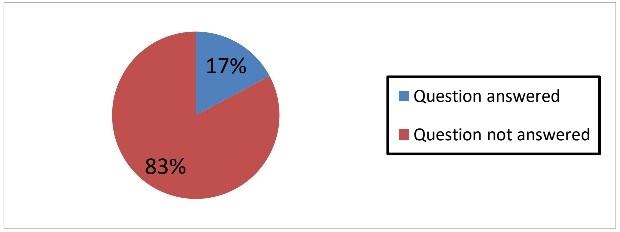

Detailed results for entry field of In-house prepared reference materials - Row 1 / Name

Variable	V166.C11		
Number Answers	5	Number unique	5

Value/Answer	Number	Frequency
Charcoal stripped plasma	1	20%
Complex matrix	1	20%
Kuddelmuddel	1	20%
Miv ultrapool	1	20%
Qc sample for large studies	1	20%
**Total**	**5**	**100%**

Question 30 - In-house prepared reference materials

Status: July 18, 2024, 13:37, Survey: "DGMet-Survey"

Number of participants evaluated: 29 (all participants)

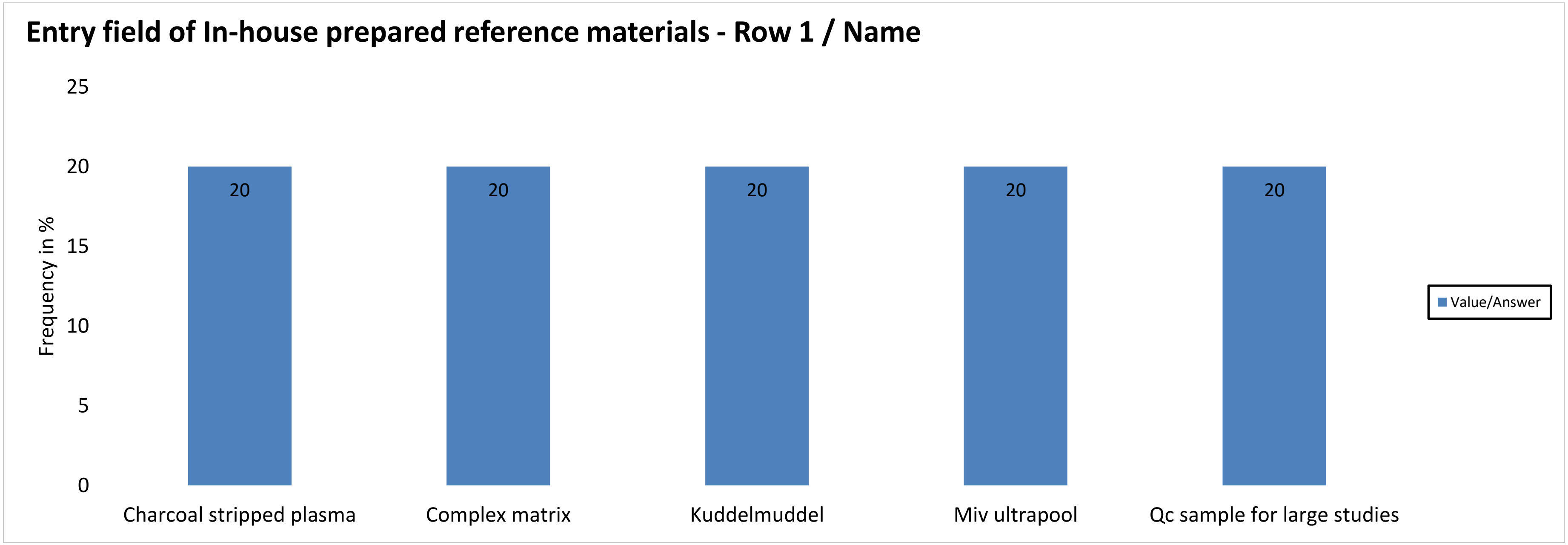

Question 30 - In-house prepared reference materials

Status: July 18, 2024, 13:37, Survey: "DGMet-Survey"

Number of participants evaluated: 29 (all participants)

Detailed results for entry field of In-house prepared reference materials - Row 1 / Species/Matrix

Variable	V166.C12		
Number Answers	5	Number unique	5

Value/Answer	Number	Frequency
For each specific matrix	1	20%
Hepg2	1	20%
Human	1	20%
Liver	1	20%
Mix of murine liver and heart in h2o:meoh:acn	1	20%
**Total**	**5**	**100%**

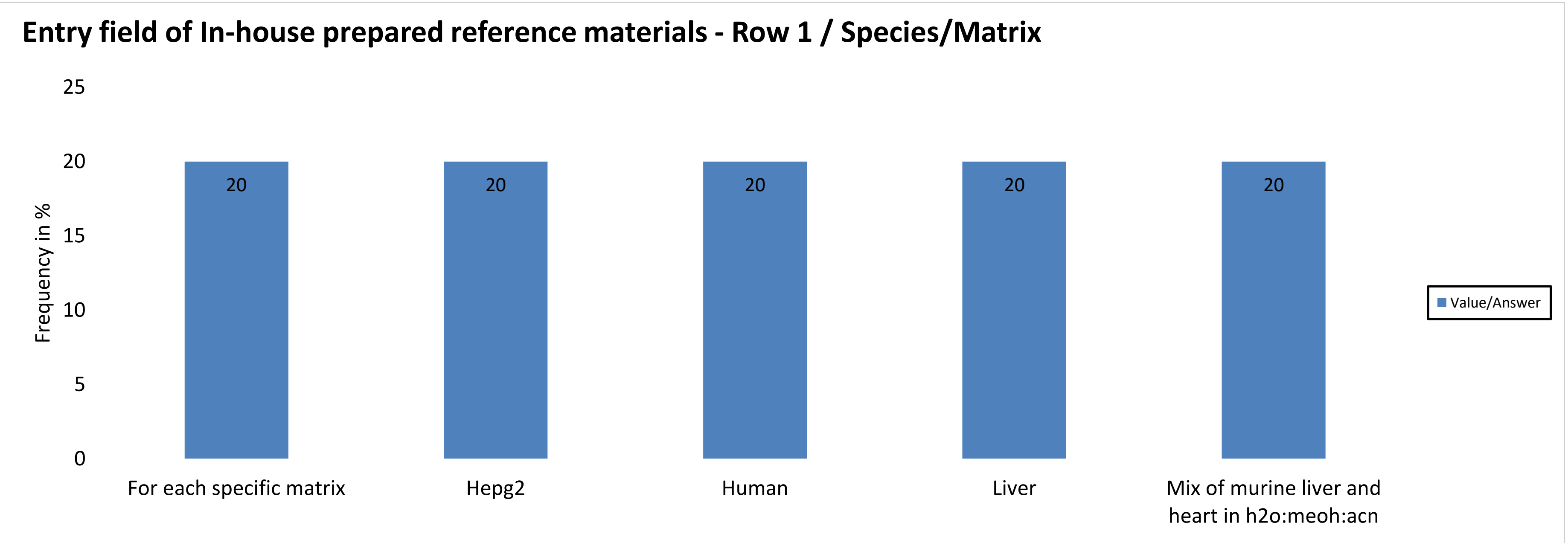

Question 30 - In-house prepared reference materials

Status: July 18, 2024, 13:37, Survey: "DGMet-Survey"

Number of participants evaluated: 29 (all participants)

Detailed results for entry field of In-house prepared reference materials - Row 2 / Name

Variable	V169.C11		
Number Answers	1	Number unique	1

Value/Answer	Number	Frequency
Study pool	1	100%
**Total**	**1**	**100%**

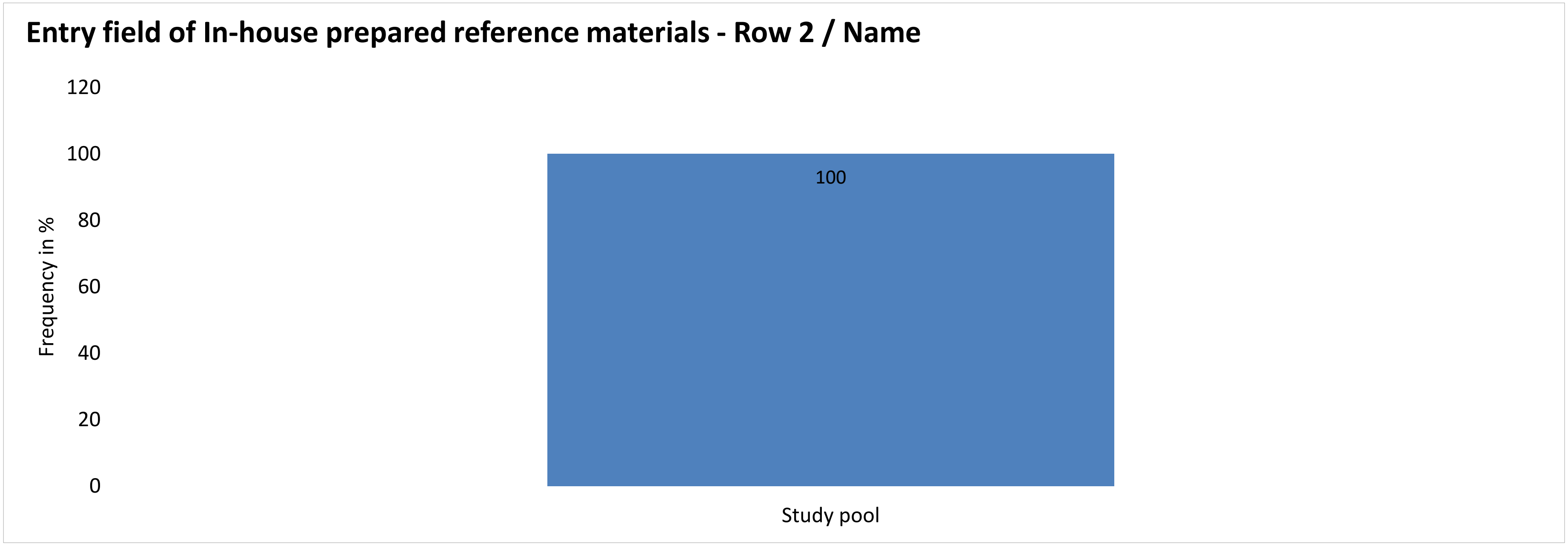

Question 30 - In-house prepared reference materials

Status: July 18, 2024, 13:37, Survey: "DGMet-Survey"

Number of participants evaluated: 29 (all participants)

Detailed results for entry field of In-house prepared reference materials - Row 2 / Species/Matrix

Variable	V169.C12		
Number Answers	1	Number unique	1

Value/Answer	Number	Frequency
Experiment-specific matrix	1	100%
**Total**	**1**	**100%**

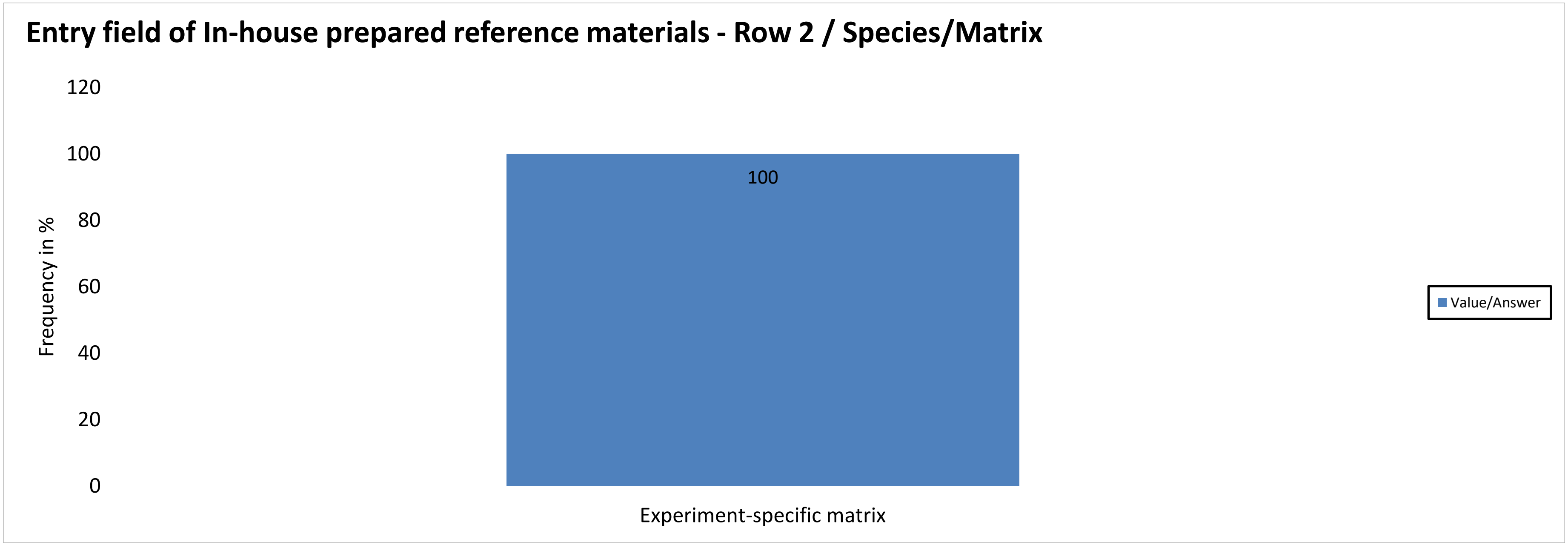

Question 30 - In-house prepared reference materials

Status: July 18, 2024, 13:37, Survey: "DGMet-Survey"

Number of participants evaluated: 29 (all participants)

Detailed results for entry field of In-house prepared reference materials - Row 3 / Name

Variable	V178.C11		
Number Answers	0	Number unique	0

Value/Answer	Number	Frequency

Created with LamaPoll | https://www.lamapoll.de

Question 30 - In-house prepared reference materials

Status: July 18, 2024, 13:37, Survey: "DGMet-Survey"

Number of participants evaluated: 29 (all participants)

Detailed results for entry field of In-house prepared reference materials - Row 3 / Species/Matrix

Variable	V178.C12		
Number Answers	0	Number unique	0

Value/Answer	Number	Frequency

Question 30 - In-house prepared reference materials

Status: July 18, 2024, 13:37, Survey: "DGMet-Survey"

Number of participants evaluated: 29 (all participants)

Detailed results for entry field of In-house prepared reference materials - Row 4 / Name

Variable	V179.C11		
Number Answers	0	Number unique	0

Value/Answer	Number	Frequency

Question 30 - In-house prepared reference materials

Status: July 18, 2024, 13:37, Survey: "DGMet-Survey"

Number of participants evaluated: 29 (all participants)

Detailed results for entry field of In-house prepared reference materials - Row 4 / Species/Matrix

Variable	V179.C12		
Number Answers	0	Number unique	0

Value/Answer	Number	Frequency

Question 30 - In-house prepared reference materials

Status: July 18, 2024, 13:37, Survey: "DGMet-Survey"

Number of participants evaluated: 29 (all participants)

Detailed results for entry field of In-house prepared reference materials - Row 5 / Name

Variable	V180.C11		
Number Answers	0	Number unique	0

Value/Answer	Number	Frequency

Question 30 - In-house prepared reference materials

Status: July 18, 2024, 13:37, Survey: "DGMet-Survey"

Number of participants evaluated: 29 (all participants)

Detailed results for entry field of In-house prepared reference materials - Row 5 / Species/Matrix

Variable	V180.C12		
Number Answers	0	Number unique	0

Value/Answer	Number	Frequency

Created with LamaPoll | https://www.lamapoll.de

Question 30 - In-house prepared reference materials

Status: July 18, 2024, 13:37, Survey: "DGMet-Survey"

Number of participants evaluated: 29 (all participants)

Results Isotope labelling (13C, 15N, ...) (Columns 3-4)

Frequency in %		Native	Labelled	Total
	Variable	C13	C14	
Row 1	V166	100%	0%	5
Row 2	V169	100%	0%	1
Row 3	V178	0%	0%	0
Row 4	V179	0%	0%	0
Row 5	V180	0%	0%	0

Number of answers		Native	Labelled	Total
	Variable	C13	C14	
Row 1	V166	5	0	5
Row 2	V169	1	0	1
Row 3	V178	0	0	0
Row 4	V179	0	0	0
Row 5	V180	0	0	0

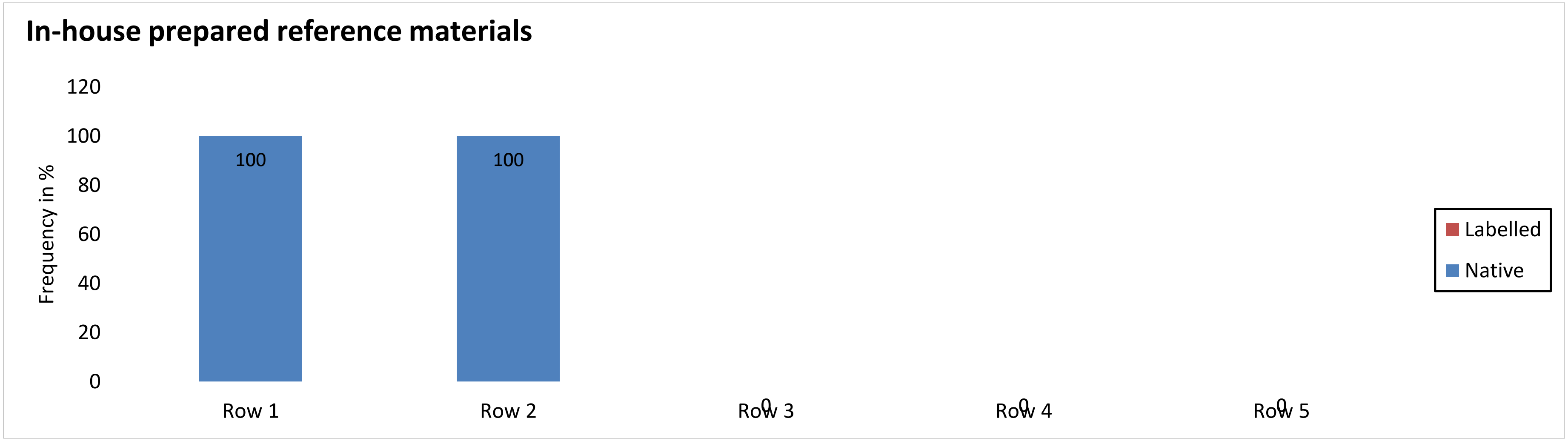

Question 31 - Are you missing standards, standard mixtures or reference materials that could support your metabolomics tools?

Status: July 18, 2024, 13:37, Survey: "DGMet-Survey"

Number of participants evaluated: 29 (all participants)

Status data

of 29 participants	Number	Percent
Question seen	22	75.86%
Question answered	20	68.97%
Question not answered	9	31.03%

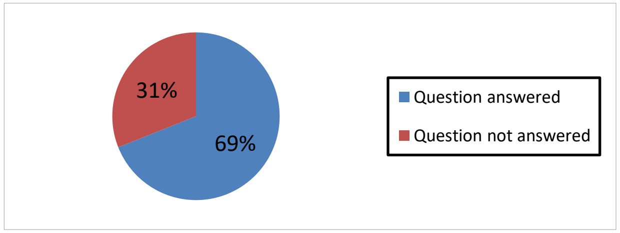

Rate from 1 = no need to 5 = strong need

Results (Total)

Frequency in %	Variable	Value 1	Value 2	Value 3	Value 4	Value 5	Total
Instrument qualification	V42	64.71%	5.88%	11.76%	11.76%	5.88%	17
System suitability tests	V43	47.06%	11.76%	5.88%	11.76%	23.53%	17
Quality control (QC) purposes	V44	35.29%	0%	35.29%	11.76%	17.65%	17
Analytical method validation	V68	11.76%	11.76%	47.06%	11.76%	17.65%	17
Calibration standards for quantification	V114	0%	11.76%	23.53%	23.53%	41.18%	17
Metabolite identification	V115	15%	5%	25%	10%	45%	20
Bridging across study sample data	V116	27.78%	11.11%	27.78%	22.22%	11.11%	18
Data pre-processing (e.g. normalization)	V117	31.25%	25%	25%	18.75%	0%	16

Frequency Number	Variable	Value 1	Value 2	Value 3	Value 4	Value 5	Total	Average	Median
Instrument qualification	V42	11	1	2	2	1	17	1.88	1
System suitability tests	V43	8	2	1	2	4	17	2.53	2
Quality control (QC) purposes	V44	6	0	6	2	3	17	2.76	3
Analytical method validation	V68	2	2	8	2	3	17	3.12	3

Question 31 - Are you missing standards, standard mixtures or reference materials that could support your metabolomics tools?

Status: July 18, 2024, 13:37, Survey: "DGMet-Survey"

Number of participants evaluated: 29 (all participants)

Calibration standards for quantification	V114	0	2	4	4	7	17	3.94	4
Metabolite identification	V115	3	1	5	2	9	20	3.65	4
Bridging across study sample data	V116	5	2	5	4	2	18	2.78	3
Data pre-processing (e.g. normalization)	V117	5	4	4	3	0	16	2.31	2
**Total**		40	14	35	21	29	**139**	**2.89**	**3**

Question 31 - Are you missing standards, standard mixtures or reference materials that could support your metabolomics tools?

Status: July 18, 2024, 13:37, Survey: "DGMet-Survey"

Number of participants evaluated: 29 (all participants)

Rate from 1 = no need to 5 = strong need

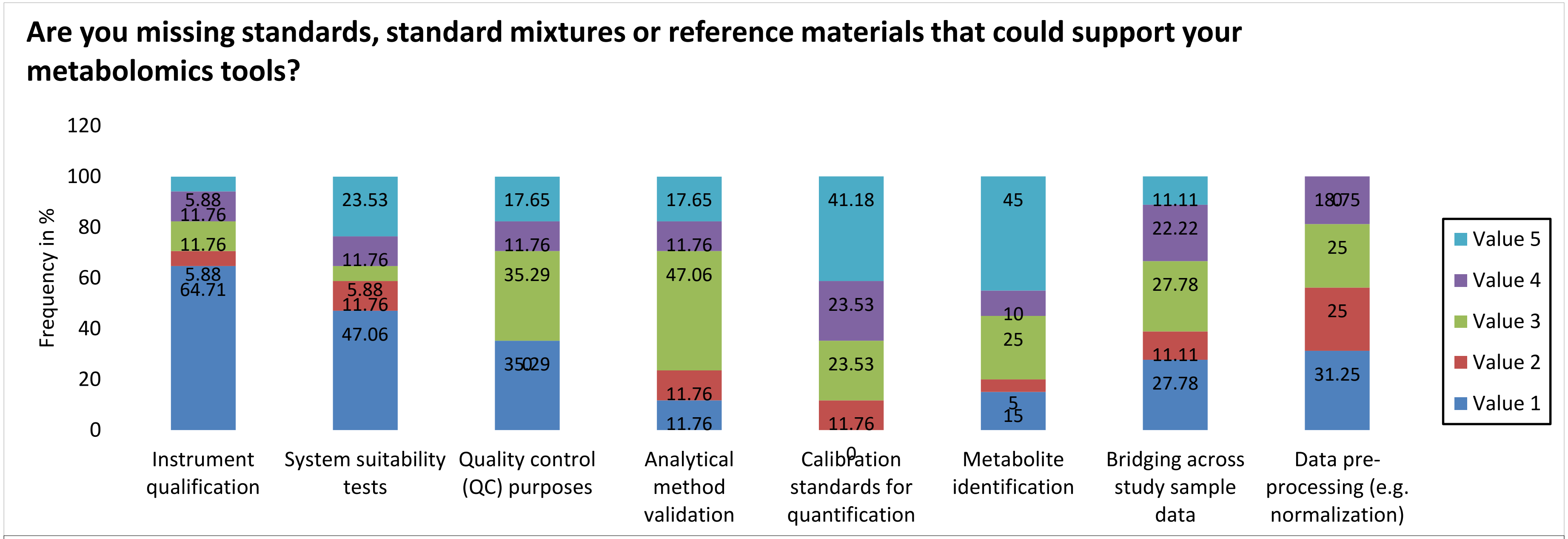

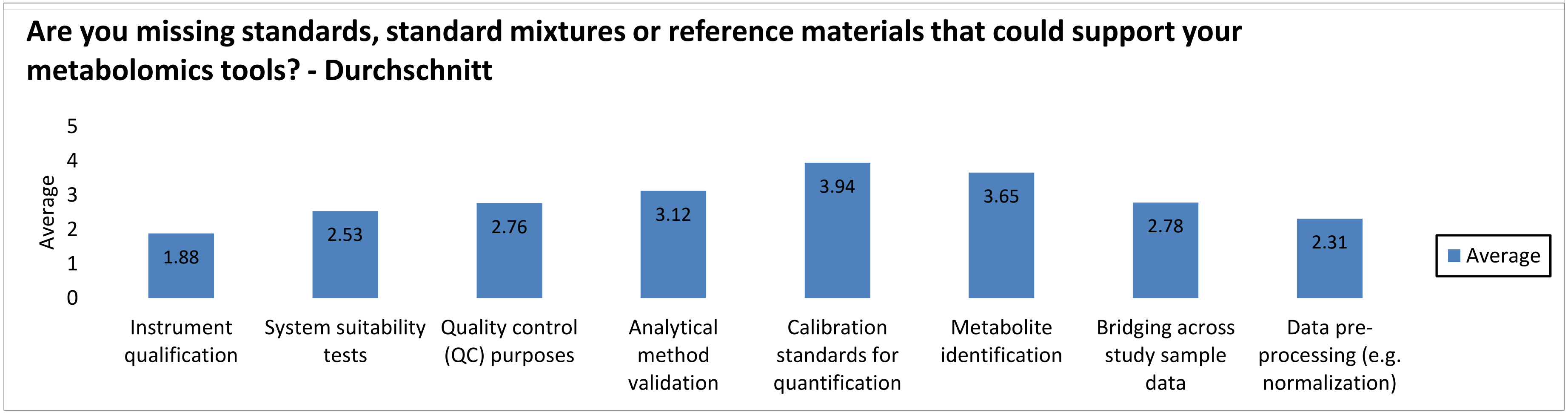

Question 31 - Are you missing standards, standard mixtures or reference materials that could support your metabolomics tools?

Status: July 18, 2024, 13:37, Survey: "DGMet-Survey"

Number of participants evaluated: 29 (all participants)

Detailed results for Instrument qualification

Variable	V42	Number of answers	17
Average	1.88	Median	1
Variance	1.75	Standard deviation	1.32
Smallest Value	1	Highest Value	5

Value/Answer	Number	Frequency
1	11	55%
2	1	5%
3	2	10%
4	2	10%
5	1	5%
**Total**	**17**	**58.62%**

Rate from 1 = no need to 5 = strong need

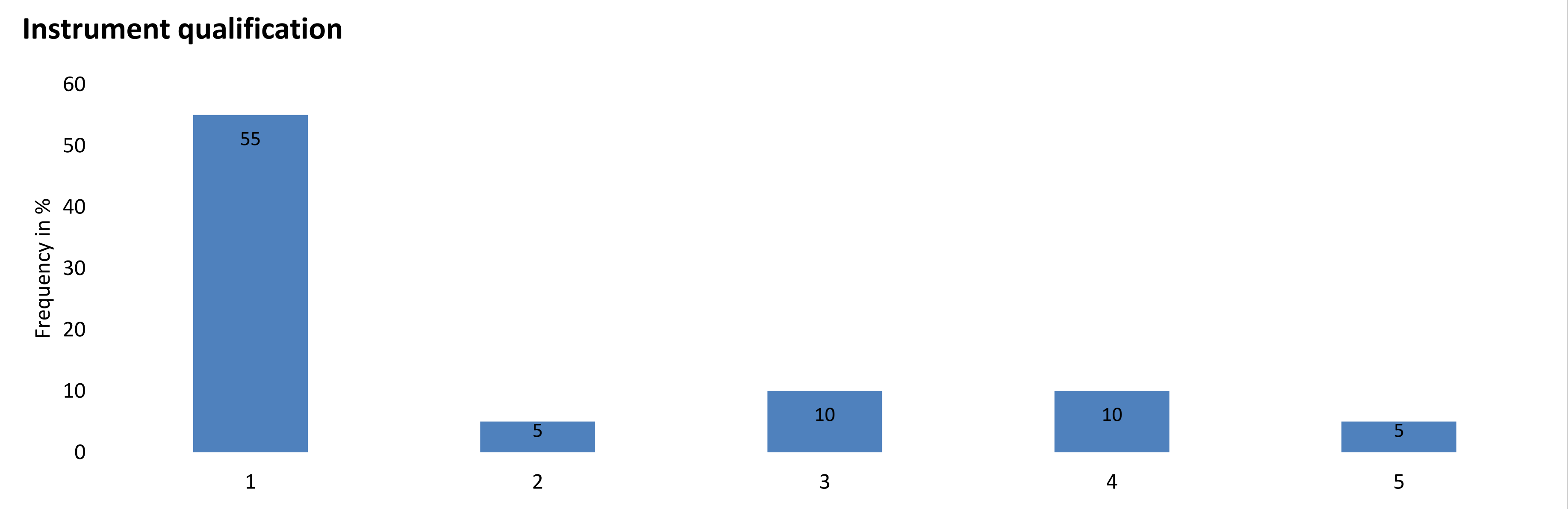

Question 31 - Are you missing standards, standard mixtures or reference materials that could support your metabolomics tools?

Status: July 18, 2024, 13:37, Survey: "DGMet-Survey"

Number of participants evaluated: 29 (all participants)

Detailed results for System suitability tests

Variable	V43	Number of answers	17
Average	2.53	Median	2
Variance	2.84	Standard deviation	1.68
Smallest Value	1	Highest Value	5

Value/Answer	Number	Frequency
1	8	40%
2	2	10%
3	1	5%
4	2	10%
5	4	20%
**Total**	**17**	**58.62%**

Rate from 1 = no need to 5 = strong need

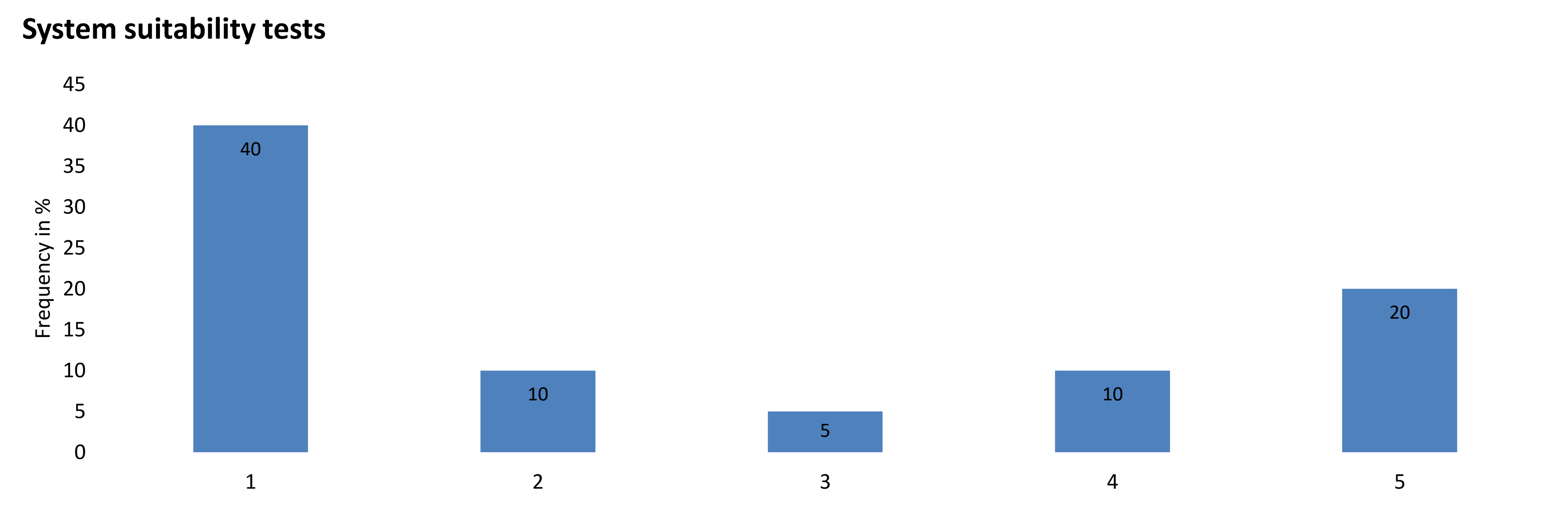

Question 31 - Are you missing standards, standard mixtures or reference materials that could support your metabolomics tools?

Status: July 18, 2024, 13:37, Survey: "DGMet-Survey"

Number of participants evaluated: 29 (all participants)

Detailed results for Quality control (QC) purposes

Variable	V44	Number of answers	17
Average	2.76	Median	3
Variance	2.18	Standard deviation	1.48
Smallest Value	1	Highest Value	5

Value/Answer	Number	Frequency
1	6	30%
2	0	0%
3	6	30%
4	2	10%
5	3	15%
**Total**	**17**	**58.62%**

Rate from 1 = no need to 5 = strong need

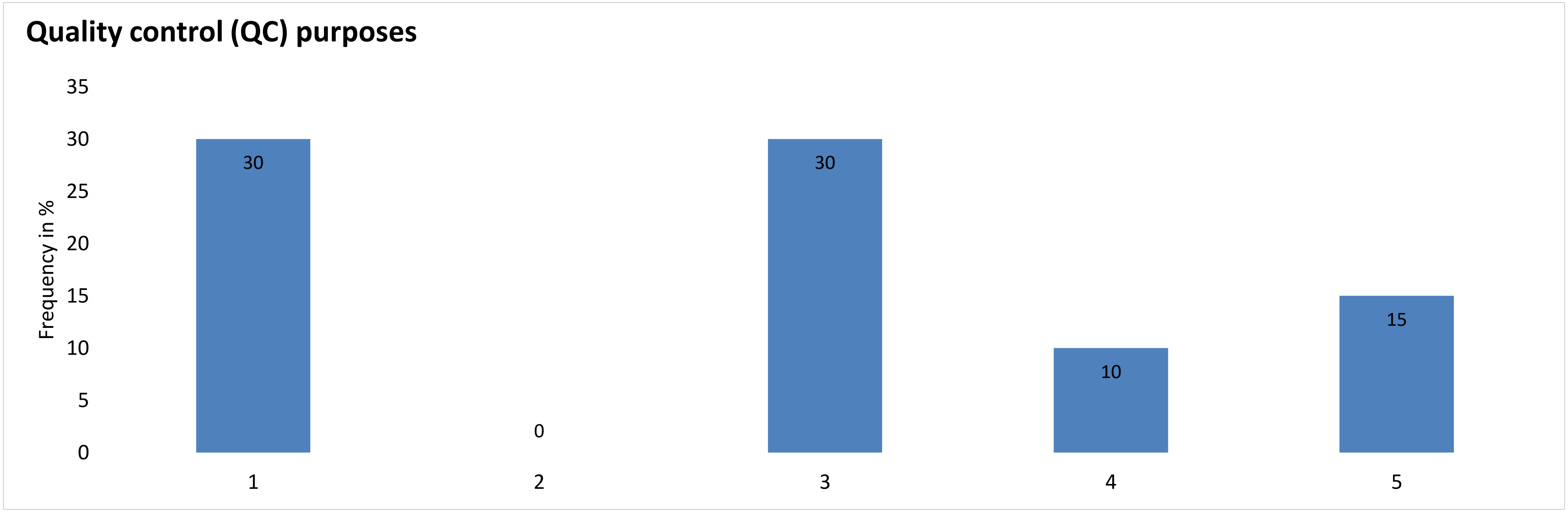

Question 31 - Are you missing standards, standard mixtures or reference materials that could support your metabolomics tools?

Status: July 18, 2024, 13:37, Survey: "DGMet-Survey"

Number of participants evaluated: 29 (all participants)

Detailed results for Analytical method validation

Variable	V68	Number of answers	17
Average	3.12	Median	3
Variance	1.40	Standard deviation	1.18
Smallest Value	1	Highest Value	5

Value/Answer	Number	Frequency
1	2	10%
2	2	10%
3	8	40%
4	2	10%
5	3	15%
**Total**	**17**	**58.62%**

Rate from 1 = no need to 5 = strong need

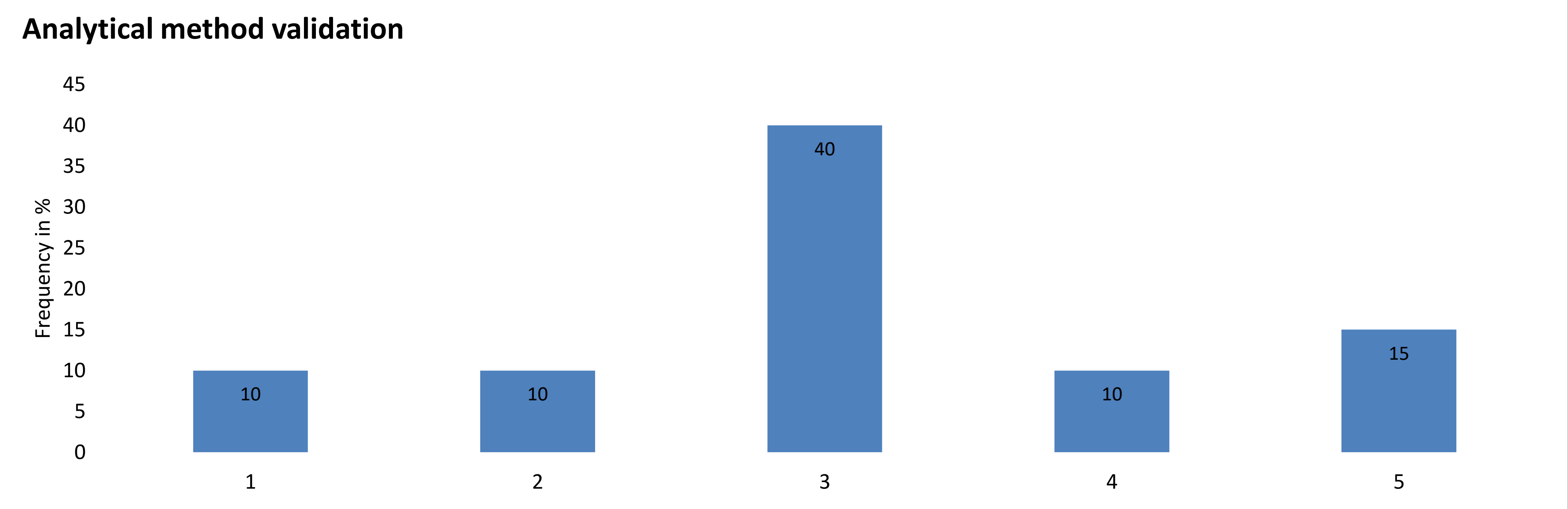

Question 31 - Are you missing standards, standard mixtures or reference materials that could support your metabolomics tools?

Status: July 18, 2024, 13:37, Survey: "DGMet-Survey"

Number of participants evaluated: 29 (all participants)

Detailed results for Calibration standards for quantification

Variable	V114	Number of answers	17
Average	3.94	Median	4
Variance	1.11	Standard deviation	1.06
Smallest Value	2	Highest Value	5

Value/Answer	Number	Frequency
1	0	0%
2	2	10%
3	4	20%
4	4	20%
5	7	35%
**Total**	**17**	**58.62%**

Rate from 1 = no need to 5 = strong need

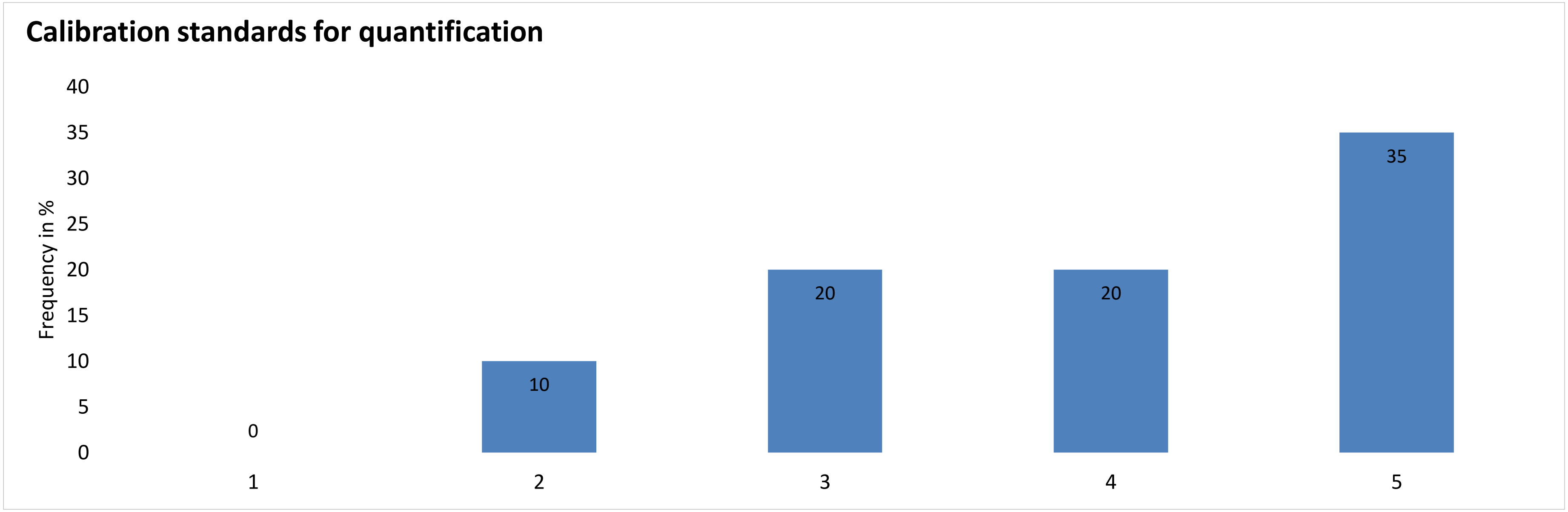

Question 31 - Are you missing standards, standard mixtures or reference materials that could support your metabolomics tools?

Status: July 18, 2024, 13:37, Survey: "DGMet-Survey"

Number of participants evaluated: 29 (all participants)

Detailed results for Metabolite identification

Variable	V115	Number of answers	20
Average	3.65	Median	4
Variance	2.13	Standard deviation	1.46
Smallest Value	1	Highest Value	5

Value/Answer	Number	Frequency
1	3	15%
2	1	5%
3	5	25%
4	2	10%
5	9	45%
**Total**	**20**	**68.97%**

Rate from 1 = no need to 5 = strong need

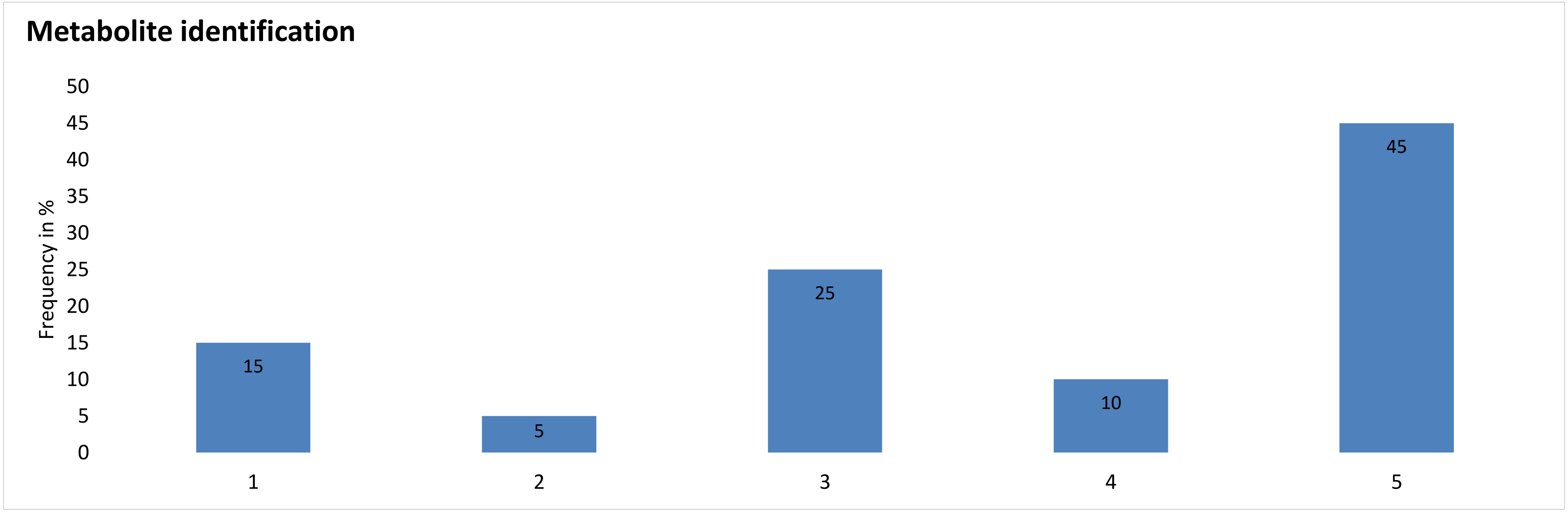

Question 31 - Are you missing standards, standard mixtures or reference materials that could support your metabolomics tools?

Status: July 18, 2024, 13:37, Survey: "DGMet-Survey"

Number of participants evaluated: 29 (all participants)

Detailed results for Bridging across study sample data

Variable	V116	Number of answers	18
Average	2.78	Median	3
Variance	1.84	Standard deviation	1.36
Smallest Value	1	Highest Value	5

Value/Answer	Number	Frequency
1	5	25%
2	2	10%
3	5	25%
4	4	20%
5	2	10%
**Total**	**18**	**62.07%**

Rate from 1 = no need to 5 = strong need

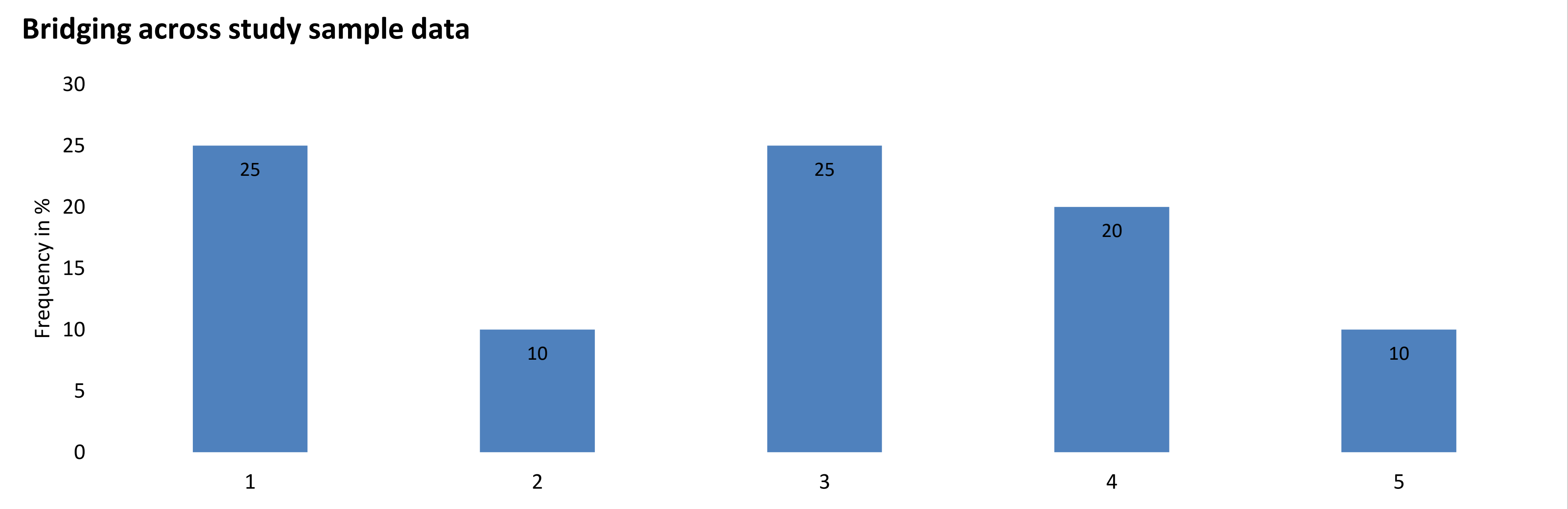

Question 31 - Are you missing standards, standard mixtures or reference materials that could support your metabolomics tools?

Status: July 18, 2024, 13:37, Survey: "DGMet-Survey"

Number of participants evaluated: 29 (all participants)

Detailed results for Data pre-processing (e.g. normalization)

Variable	V117	Number of answers	16
Average	2.31	Median	2
Variance	1.21	Standard deviation	1.10
Smallest Value	1	Highest Value	4

Value/Answer	Number	Frequency
1	5	25%
2	4	20%
3	4	20%
4	3	15%
5	0	0%
**Total**	**16**	**55.17%**

Rate from 1 = no need to 5 = strong need

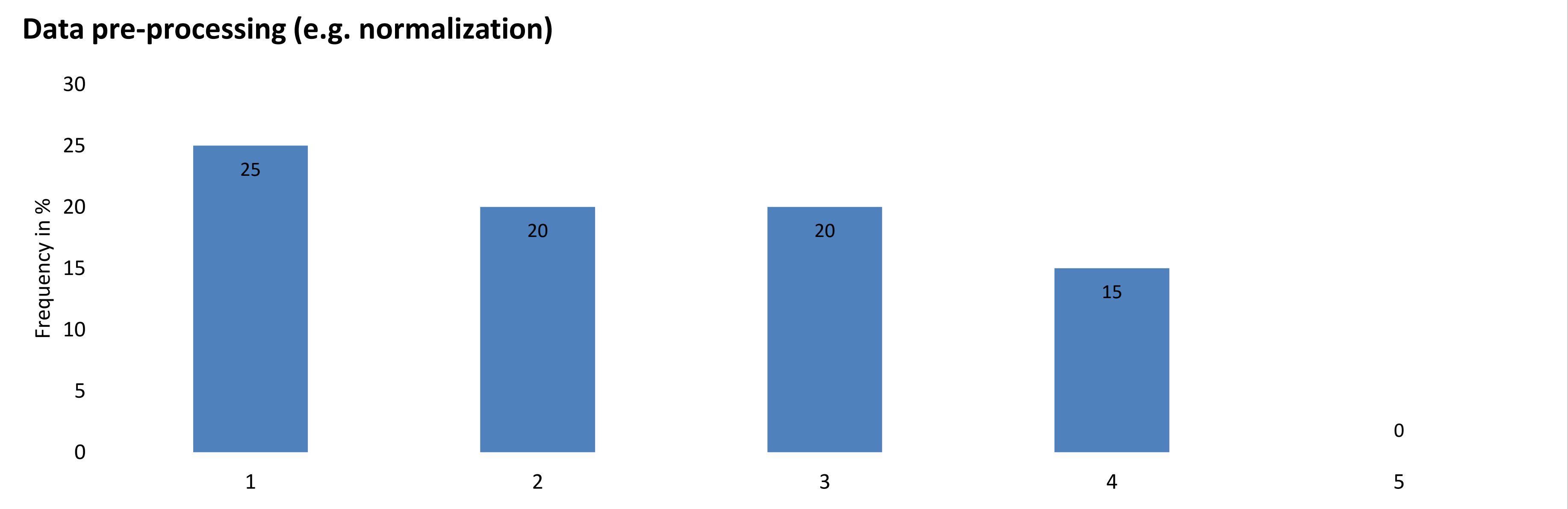

Question 32 - Please indicate the type of new standards or reference materials that you feel are needed.

Status: July 18, 2024, 13:37, Survey: "DGMet-Survey"

Number of participants evaluated: 29 (all participants)

Rate from 1 = no need to 5 = strong need

Status data

of 29 participants	Number	Percent
Question seen	22	75.86%
Question answered	18	62.07%
Question not answered	11	37.93%

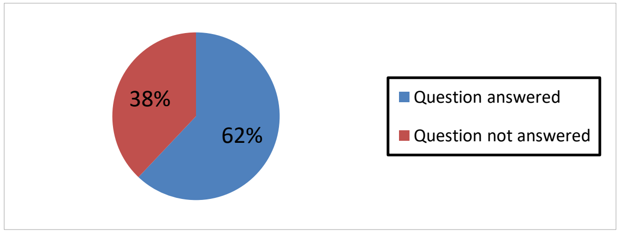

Results (Total)

Frequency in %	Variable	Value 1	Value 2	Value 3	Value 4	Value 5	Total
Single chemical standards - unlabelled	V172	25%	6.25%	12.50%	25%	31.25%	16
Single chemical standards - isotope labelled	V173	17.65%	5.88%	17.65%	17.65%	41.18%	17
Compound mixtures or libraries - unlabelled	V174	11.76%	11.76%	17.65%	23.53%	35.29%	17
Compound mixtures or libraries - isotope labelled	V175	11.11%	11.11%	22.22%	11.11%	44.44%	18
Reference material (including research grade testing materials)	V176	5.88%	23.53%	17.65%	17.65%	35.29%	17
Certified reference material (CRM)	V181	29.41%	17.65%	23.53%	5.88%	23.53%	17

Frequency Number	Variable	Value 1	Value 2	Value 3	Value 4	Value 5	Total	Average	Median
Single chemical standards - unlabelled	V172	4	1	2	4	5	16	3.31	4
Single chemical standards - isotope labelled	V173	3	1	3	3	7	17	3.59	4
Compound mixtures or libraries - unlabelled	V174	2	2	3	4	6	17	3.59	4

Question 32 - Please indicate the type of new standards or reference materials that you feel are needed.

Status: July 18, 2024, 13:37, Survey: "DGMet-Survey"

Number of participants evaluated: 29 (all participants)

Compound mixtures or libraries - isotope labelled	V175	2	2	4	2	8	18	3.67	4
Reference material (including research grade testing materials)	V176	1	4	3	3	6	17	3.53	4
Certified reference material (CRM)	V181	5	3	4	1	4	17	2.76	3
**Total**		17	13	19	17	36	**102**	**3.41**	**4**

Question 32 - Please indicate the type of new standards or reference materials that you feel are needed.

Status: July 18, 2024, 13:37, Survey: "DGMet-Survey"

Number of participants evaluated: 29 (all participants)

Rate from 1 = no need to 5 = strong need

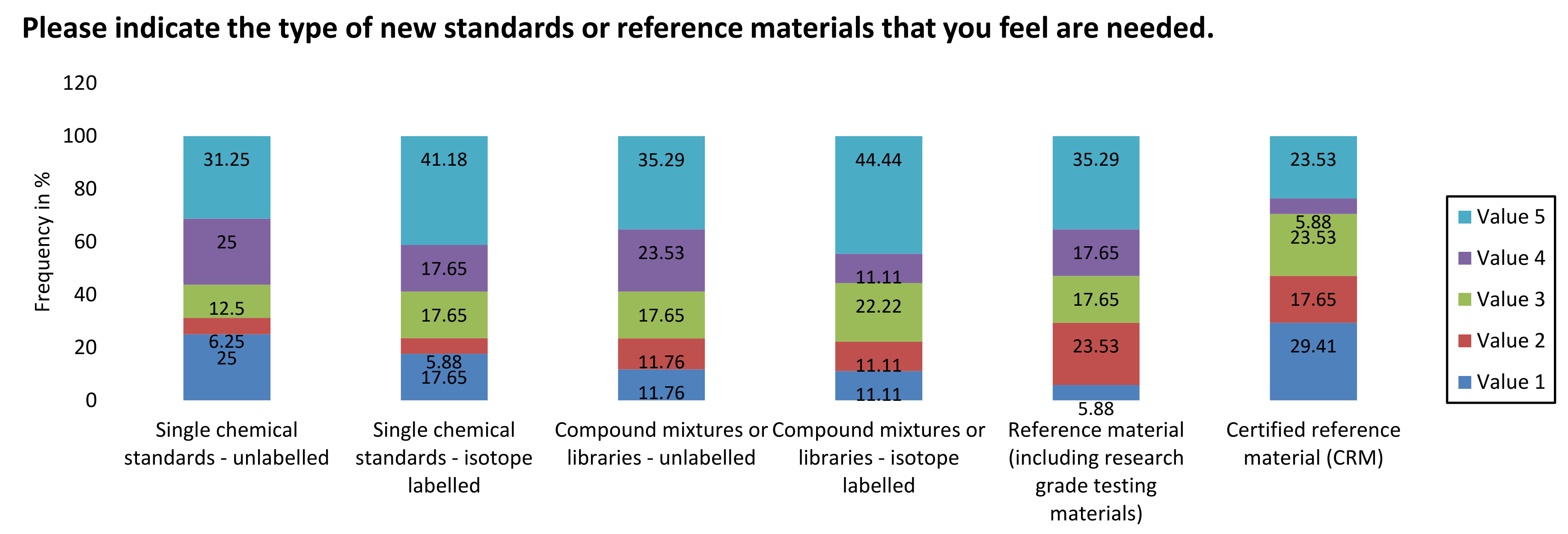

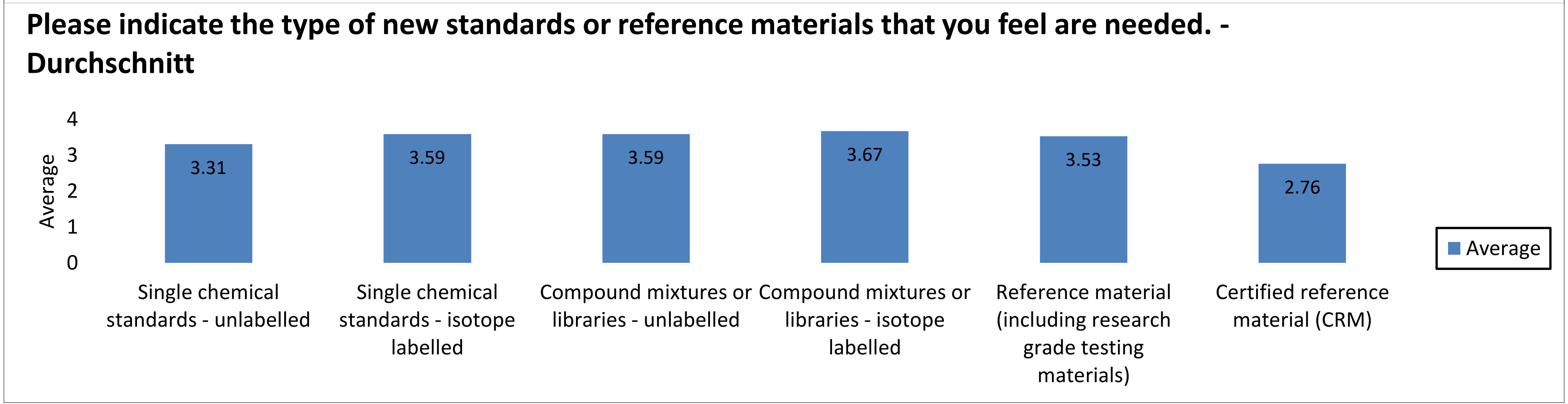

Question 32 - Please indicate the type of new standards or reference materials that you feel are needed.

Status: July 18, 2024, 13:37, Survey: "DGMet-Survey"

Number of participants evaluated: 29 (all participants)

Detailed results for Single chemical standards - unlabelled

Variable	V172	Number of answers	16
Average	3.31	Median	4
Variance	2.46	Standard deviation	1.57
Smallest Value	1	Highest Value	5

Value/Answer	Number	Frequency
1	4	22.22%
2	1	5.56%
3	2	11.11%
4	4	22.22%
5	5	27.78%
**Total**	**16**	**55.17%**

Rate from 1 = no need to 5 = strong need

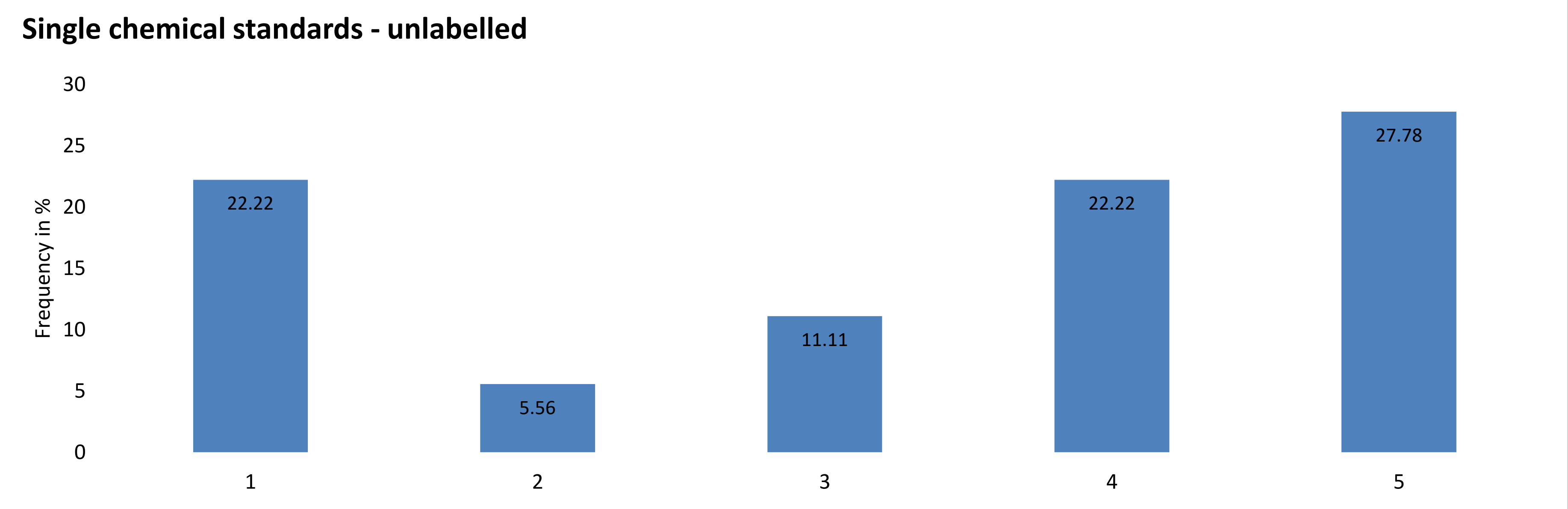

Question 32 - Please indicate the type of new standards or reference materials that you feel are needed.

Status: July 18, 2024, 13:37, Survey: "DGMet-Survey"

Number of participants evaluated: 29 (all participants)

Detailed results for Single chemical standards - isotope labelled

Variable	V173	Number of answers	17
Average	3.59	Median	4
Variance	2.24	Standard deviation	1.50
Smallest Value	1	Highest Value	5

Value/Answer	Number	Frequency
1	3	16.67%
2	1	5.56%
3	3	16.67%
4	3	16.67%
5	7	38.89%
**Total**	**17**	**58.62%**

Rate from 1 = no need to 5 = strong need

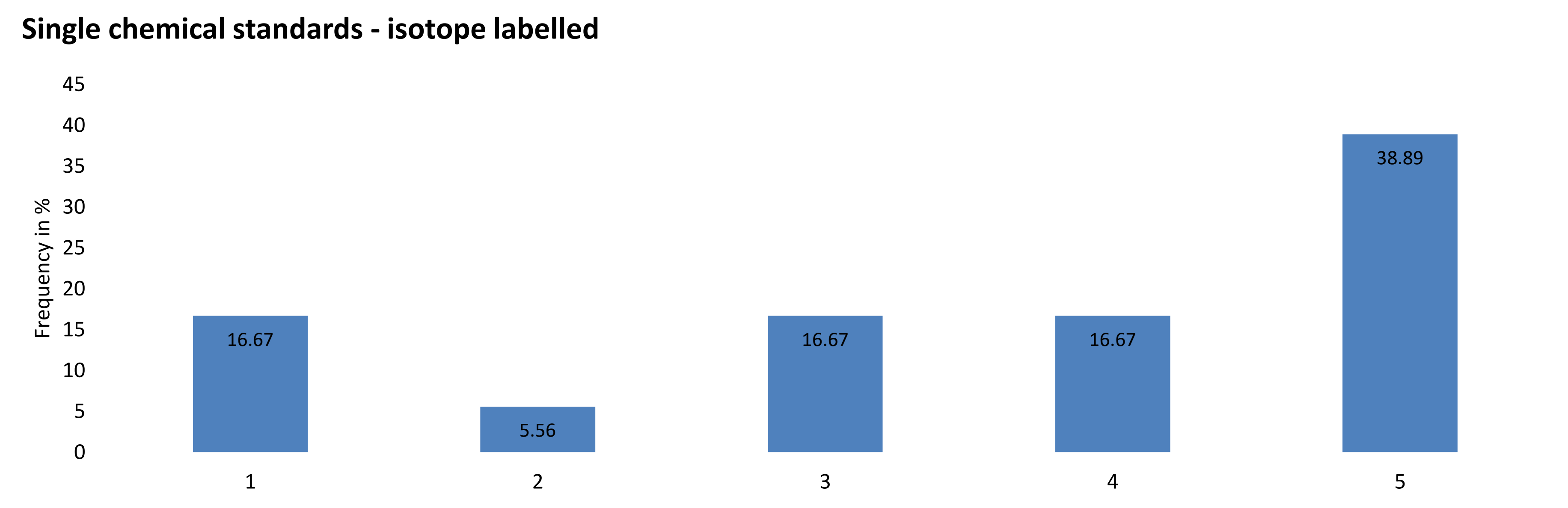

Question 32 - Please indicate the type of new standards or reference materials that you feel are needed.

Status: July 18, 2024, 13:37, Survey: "DGMet-Survey"

Number of participants evaluated: 29 (all participants)

Detailed results for Compound mixtures or libraries - unlabelled

Variable	V174	Number of answers	17
Average	3.59	Median	4
Variance	1.89	Standard deviation	1.37
Smallest Value	1	Highest Value	5

Value/Answer	Number	Frequency
1	2	11.11%
2	2	11.11%
3	3	16.67%
4	4	22.22%
5	6	33.33%
**Total**	**17**	**58.62%**

Rate from 1 = no need to 5 = strong need

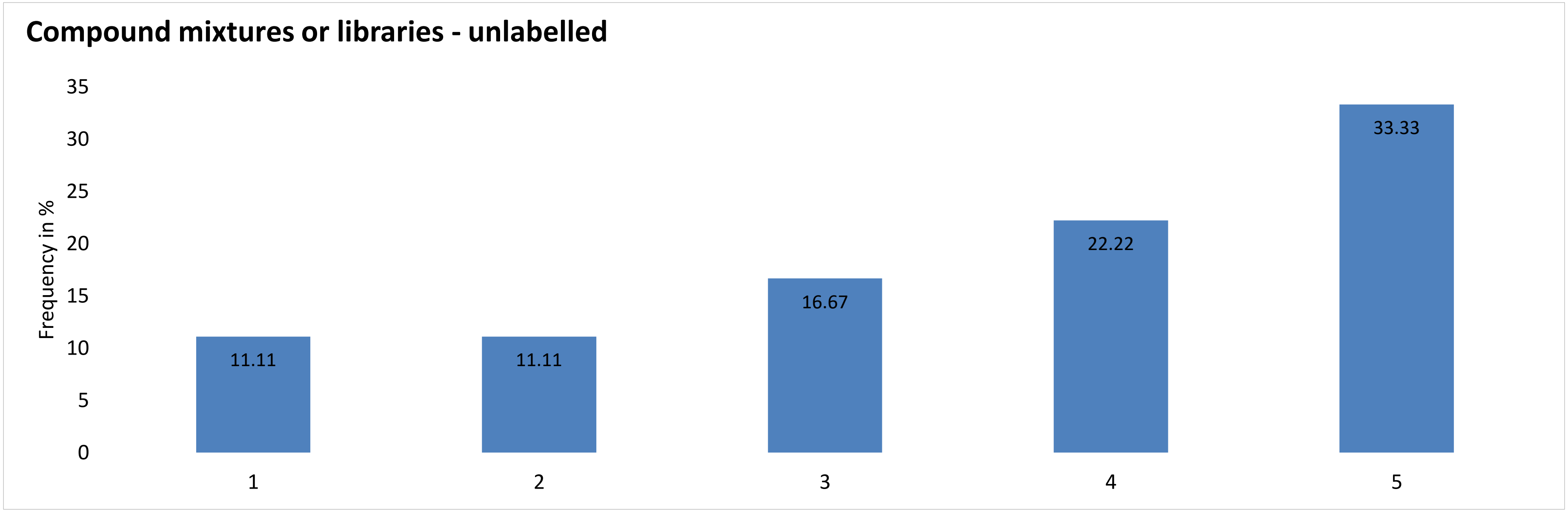

Question 32 - Please indicate the type of new standards or reference materials that you feel are needed.

Status: July 18, 2024, 13:37, Survey: "DGMet-Survey"

Number of participants evaluated: 29 (all participants)

Detailed results for Compound mixtures or libraries - isotope labelled

Variable	V175	Number of answers	18
Average	3.67	Median	4
Variance	2	Standard deviation	1.41
Smallest Value	1	Highest Value	5

Value/Answer	Number	Frequency
1	2	11.11%
2	2	11.11%
3	4	22.22%
4	2	11.11%
5	8	44.44%
**Total**	**18**	**62.07%**

Rate from 1 = no need to 5 = strong need

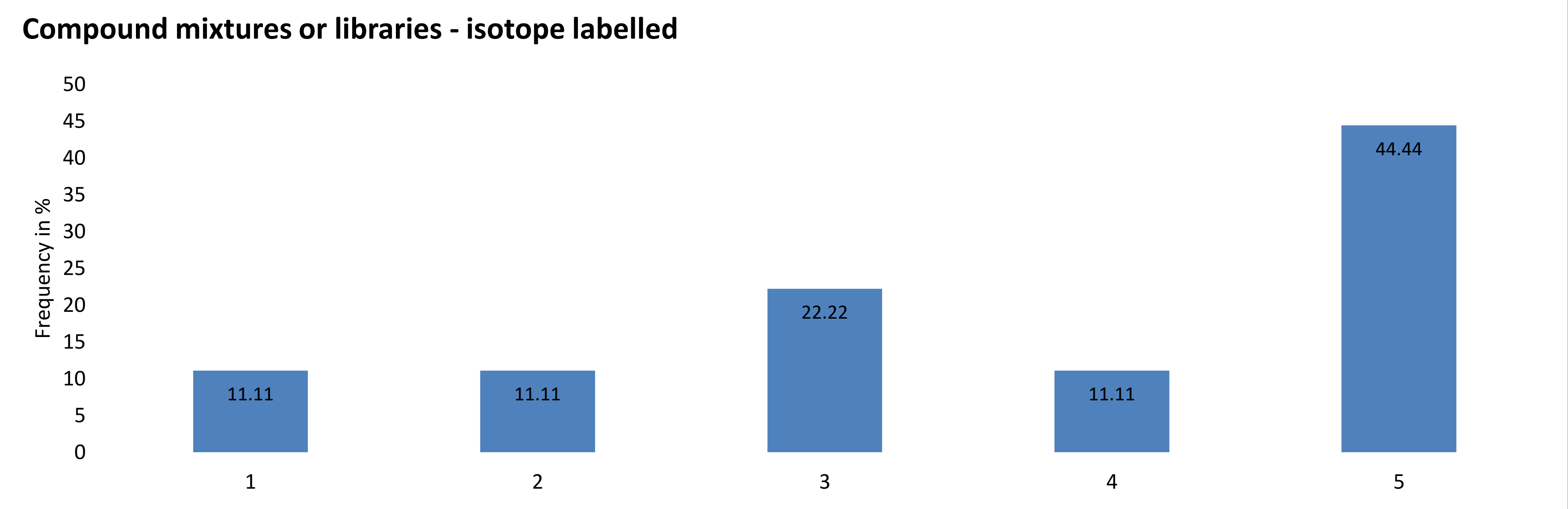

Question 32 - Please indicate the type of new standards or reference materials that you feel are needed.

Status: July 18, 2024, 13:37, Survey: "DGMet-Survey"

Number of participants evaluated: 29 (all participants)

Detailed results for Reference material (including research grade testing materials)

Variable	V176	Number of answers	17
Average	3.53	Median	4
Variance	1.78	Standard deviation	1.33
Smallest Value	1	Highest Value	5

Value/Answer	Number	Frequency
1	1	5.56%
2	4	22.22%
3	3	16.67%
4	3	16.67%
5	6	33.33%
**Total**	**17**	**58.62%**

Rate from 1 = no need to 5 = strong need

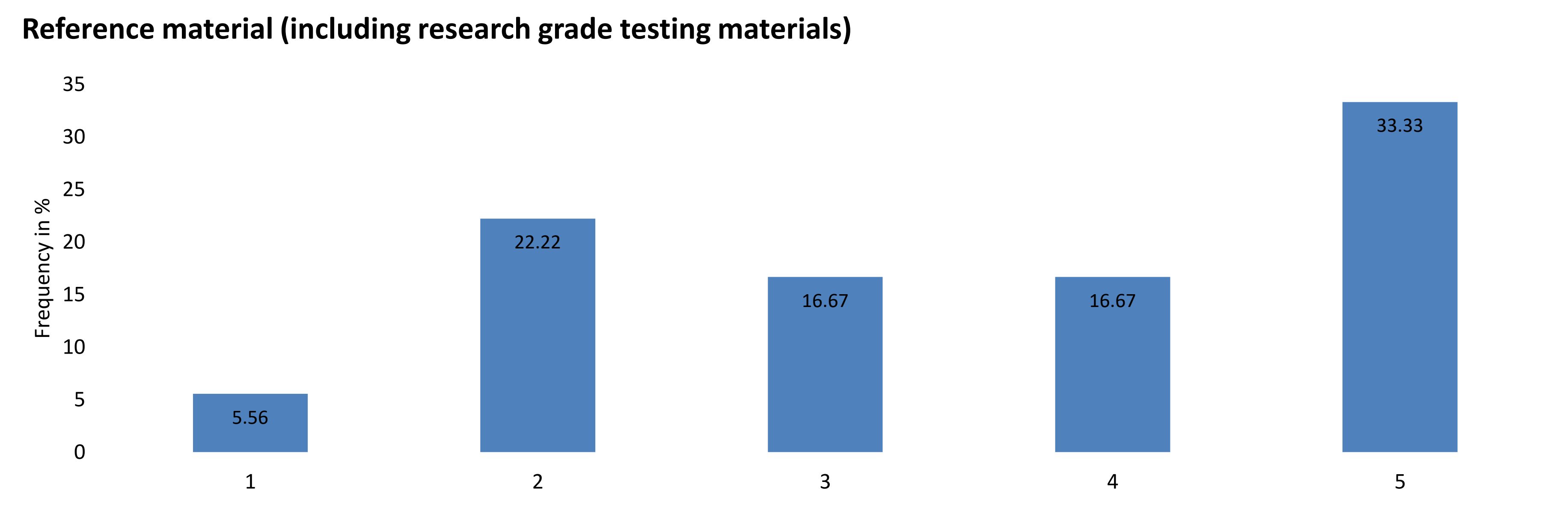

Question 32 - Please indicate the type of new standards or reference materials that you feel are needed.

Status: July 18, 2024, 13:37, Survey: "DGMet-Survey"

Number of participants evaluated: 29 (all participants)

Detailed results for Certified reference material (CRM)

Variable	V181	Number of answers	17
Average	2.76	Median	3
Variance	2.30	Standard deviation	1.52
Smallest Value	1	Highest Value	5

Value/Answer	Number	Frequency
1	5	27.78%
2	3	16.67%
3	4	22.22%
4	1	5.56%
5	4	22.22%
**Total**	**17**	**58.62%**

Rate from 1 = no need to 5 = strong need

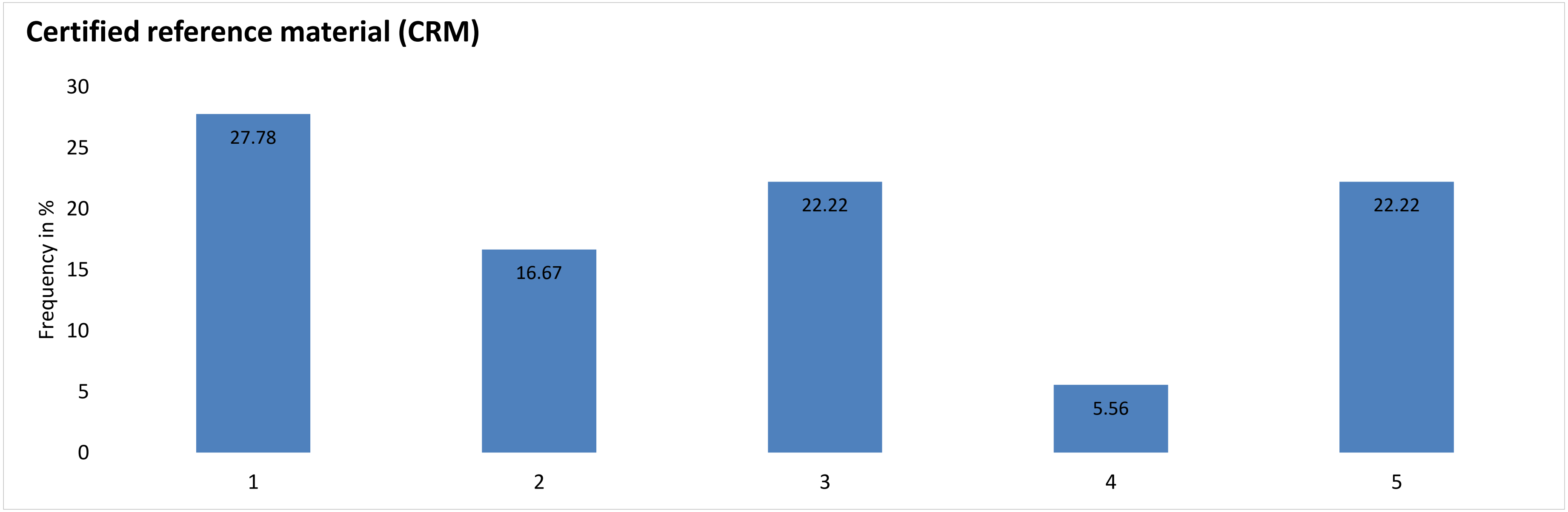

Question 33 - Is cost prohibiting you from using chemical standards or reference materials in your metabolomics workflow?

Status: July 18, 2024, 13:37, Survey: "DGMet-Survey"

Number of participants evaluated: 29 (all participants)

Rate from 1 = cost is no issue to 5 = cost is problematic

Status data

of 29 participants	Number	Percent
Question seen	22	75.86%
Question answered	17	58.62%
Question not answered	12	41.38%

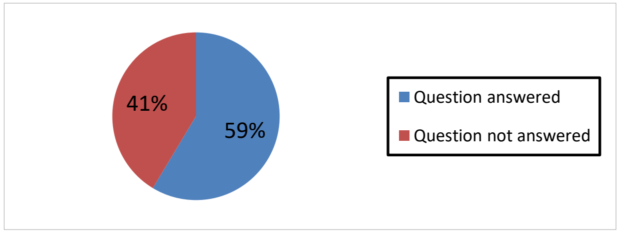

Results (Total)

Frequency in %	Variable	Value 1	Value 2	Value 3	Value 4	Value 5	Total
Single chemical standards - unlabelled	V189	29.41%	23.53%	29.41%	11.76%	5.88%	17
Single chemical standards - isotope labelled	V190	6.25%	0%	12.50%	56.25%	25%	16
Compound mixtures or libraries - unlabelled	V191	0%	17.65%	35.29%	29.41%	17.65%	17
Compound mixtures or libraries - isotope labelled	V195	5.88%	0%	11.76%	29.41%	52.94%	17
Reference material (including research grade testing materials)	V196	0%	14.29%	35.71%	35.71%	14.29%	14
Certified reference material (CRM)	V197	16.67%	8.33%	16.67%	33.33%	25%	12

Frequency Number	Variable	Value 1	Value 2	Value 3	Value 4	Value 5	Total	Average	Median
Single chemical standards - unlabelled	V189	5	4	5	2	1	17	2.41	2
Single chemical standards - isotope labelled	V190	1	0	2	9	4	16	3.94	4
Compound mixtures or libraries - unlabelled	V191	0	3	6	5	3	17	3.47	3

Created with LamaPoll | https://www.lamapoll.de

Question 33 - Is cost prohibiting you from using chemical standards or reference materials in your metabolomics workflow?

Status: July 18, 2024, 13:37, Survey: "DGMet-Survey"

Number of participants evaluated: 29 (all participants)

Compound mixtures or libraries - isotope labelled	V195	1	0	2	5	9	17	4.24	5
Reference material (including research grade testing materials)	V196	0	2	5	5	2	14	3.50	3.50
Certified reference material (CRM)	V197	2	1	2	4	3	12	3.42	4
**Total**		9	10	22	30	22	**93**	**3.49**	**4**

Created with LamaPoll | https://www.lamapoll.de

Question 33 - Is cost prohibiting you from using chemical standards or reference materials in your metabolomics workflow?

Status: July 18, 2024, 13:37, Survey: "DGMet-Survey"

Number of participants evaluated: 29 (all participants)

Rate from 1 = cost is no issue to 5 = cost is problematic

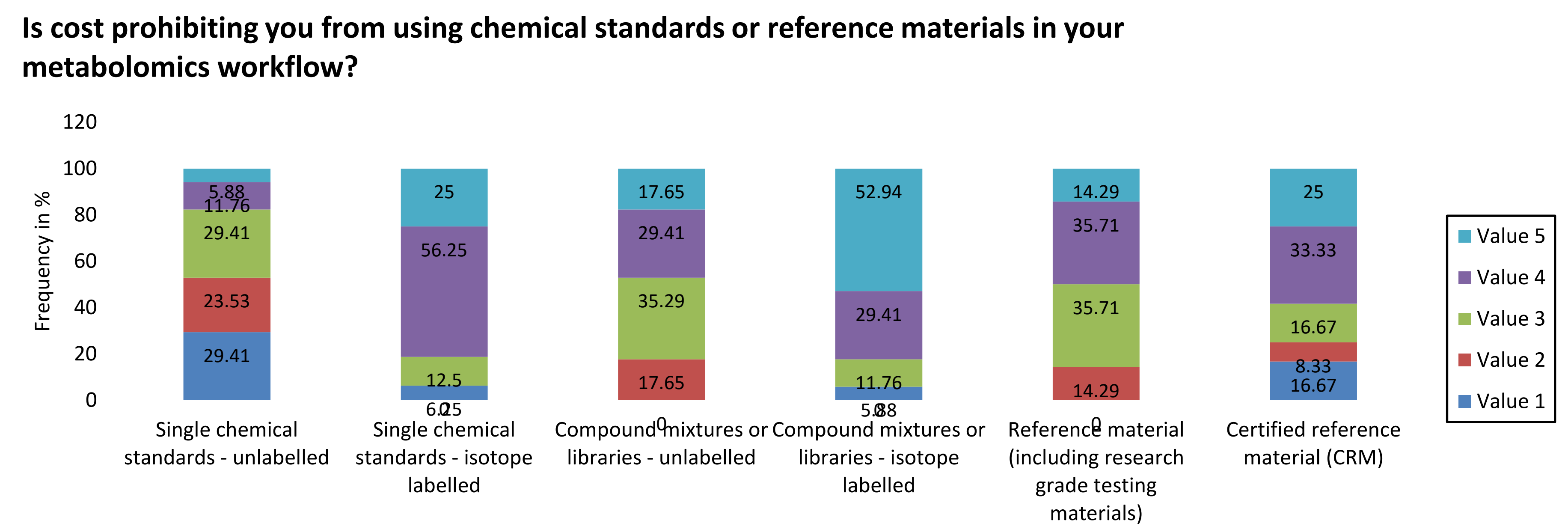

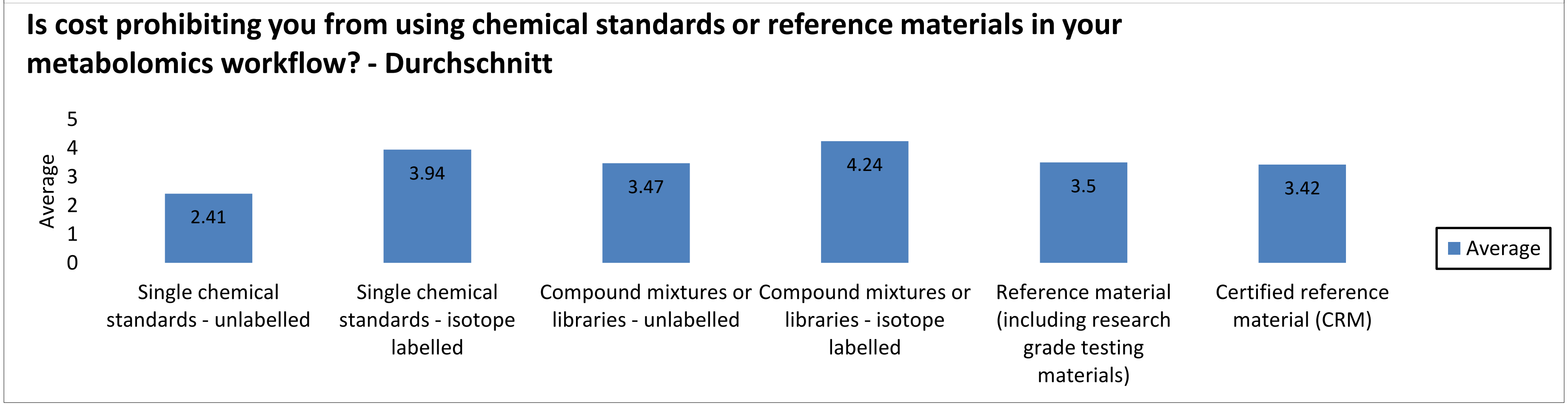

Question 33 - Is cost prohibiting you from using chemical standards or reference materials in your metabolomics workflow?

Status: July 18, 2024, 13:37, Survey: "DGMet-Survey"

Number of participants evaluated: 29 (all participants)

Detailed results for Single chemical standards - unlabelled

Variable	V189	Number of answers	17
Average	2.41	Median	2
Variance	1.42	Standard deviation	1.19
Smallest Value	1	Highest Value	5

Value/Answer	Number	Frequency
1	5	29.41%
2	4	23.53%
3	5	29.41%
4	2	11.76%
5	1	5.88%
**Total**	**17**	**58.62%**

Rate from 1 = cost is no issue to 5 = cost is problematic

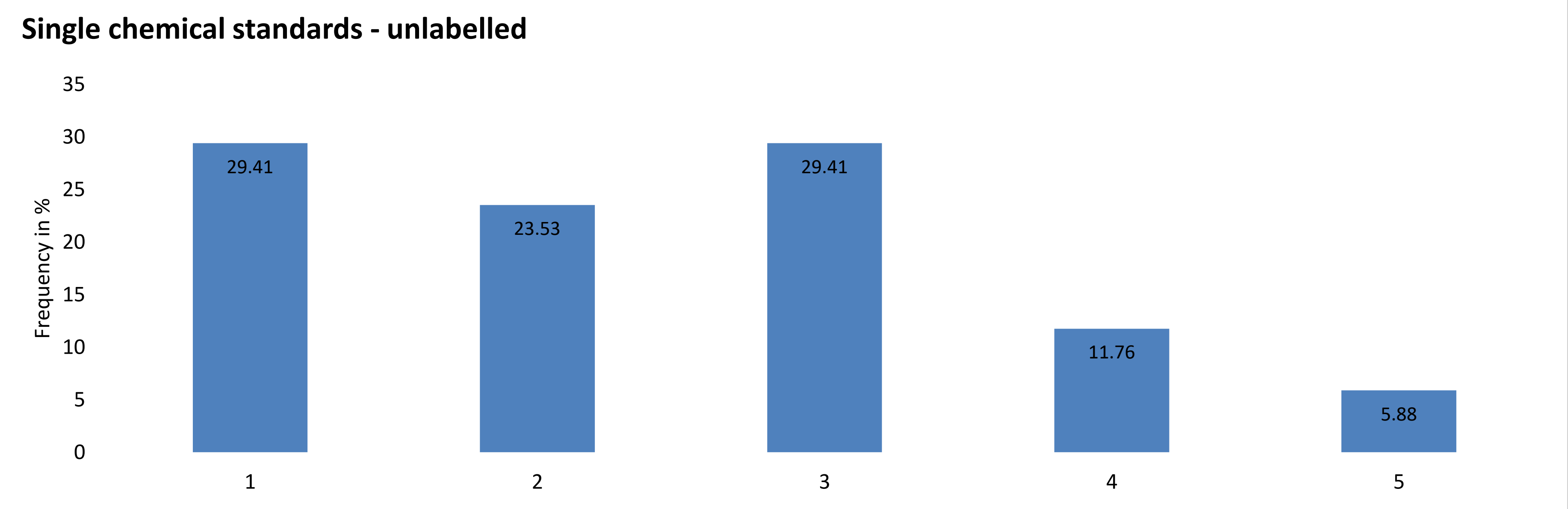

Question 33 - Is cost prohibiting you from using chemical standards or reference materials in your metabolomics workflow?

Status: July 18, 2024, 13:37, Survey: "DGMet-Survey"

Number of participants evaluated: 29 (all participants)

Detailed results for Single chemical standards - isotope labelled

Variable	V190	Number of answers	16
Average	3.94	Median	4
Variance	0.93	Standard deviation	0.97
Smallest Value	1	Highest Value	5

Value/Answer	Number	Frequency
1	1	5.88%
2	0	0%
3	2	11.76%
4	9	52.94%
5	4	23.53%
**Total**	**16**	**55.17%**

Rate from 1 = cost is no issue to 5 = cost is problematic

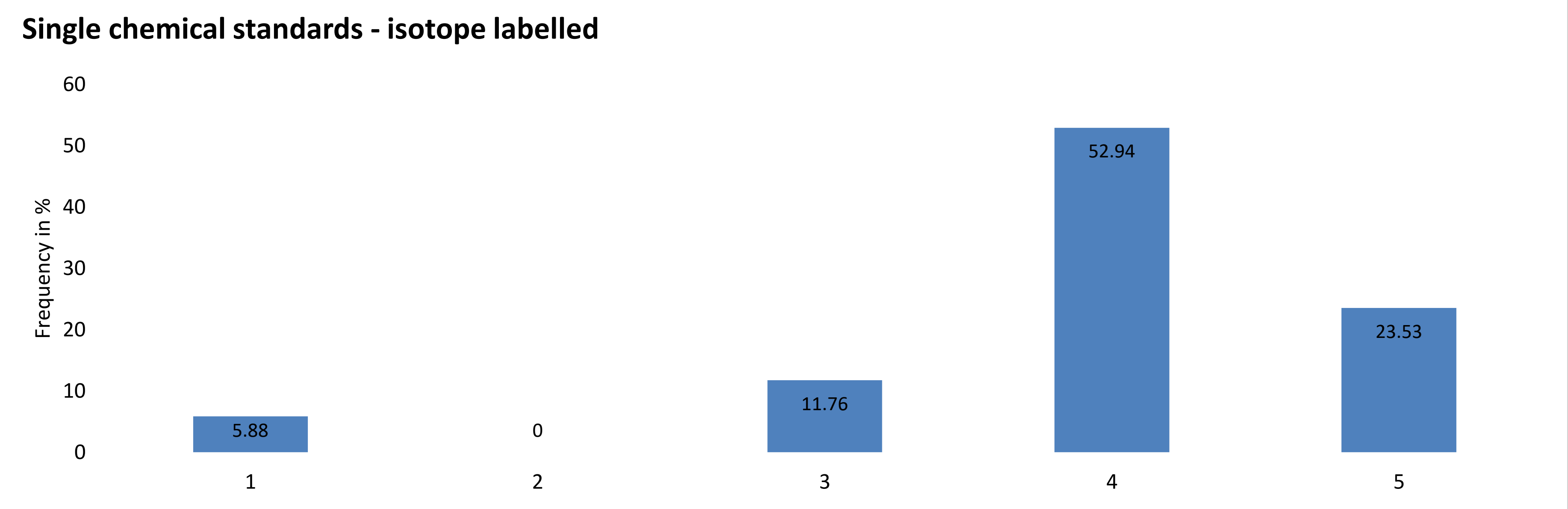

Question 33 - Is cost prohibiting you from using chemical standards or reference materials in your metabolomics workflow?

Status: July 18, 2024, 13:37, Survey: "DGMet-Survey"

Number of participants evaluated: 29 (all participants)

Detailed results for Compound mixtures or libraries - unlabelled

Variable	V191	Number of answers	17
Average	3.47	Median	3
Variance	0.96	Standard deviation	0.98
Smallest Value	2	Highest Value	5

Value/Answer	Number	Frequency
1	0	0%
2	3	17.65%
3	6	35.29%
4	5	29.41%
5	3	17.65%
**Total**	**17**	**58.62%**

Rate from 1 = cost is no issue to 5 = cost is problematic

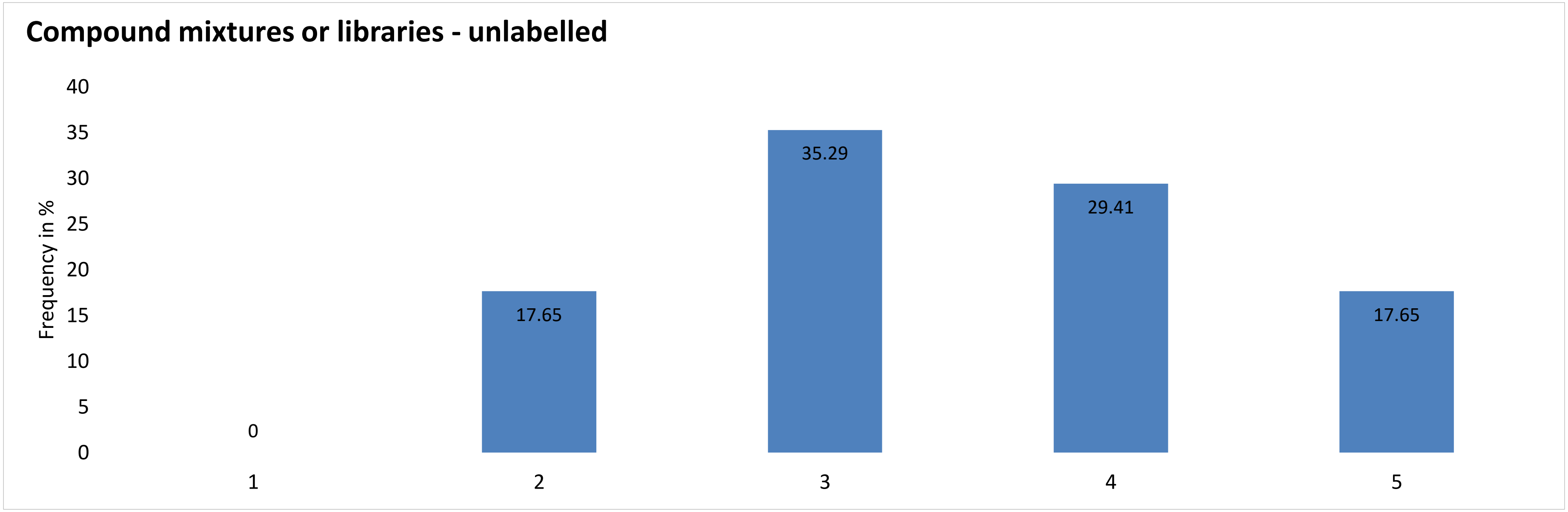

Question 33 - Is cost prohibiting you from using chemical standards or reference materials in your metabolomics workflow?

Status: July 18, 2024, 13:37, Survey: "DGMet-Survey"

Number of participants evaluated: 29 (all participants)

Detailed results for Compound mixtures or libraries - isotope labelled

Variable	V195	Number of answers	17
Average	4.24	Median	5
Variance	1.12	Standard deviation	1.06
Smallest Value	1	Highest Value	5

Value/Answer	Number	Frequency
1	1	5.88%
2	0	0%
3	2	11.76%
4	5	29.41%
5	9	52.94%
**Total**	**17**	**58.62%**

Rate from 1 = cost is no issue to 5 = cost is problematic

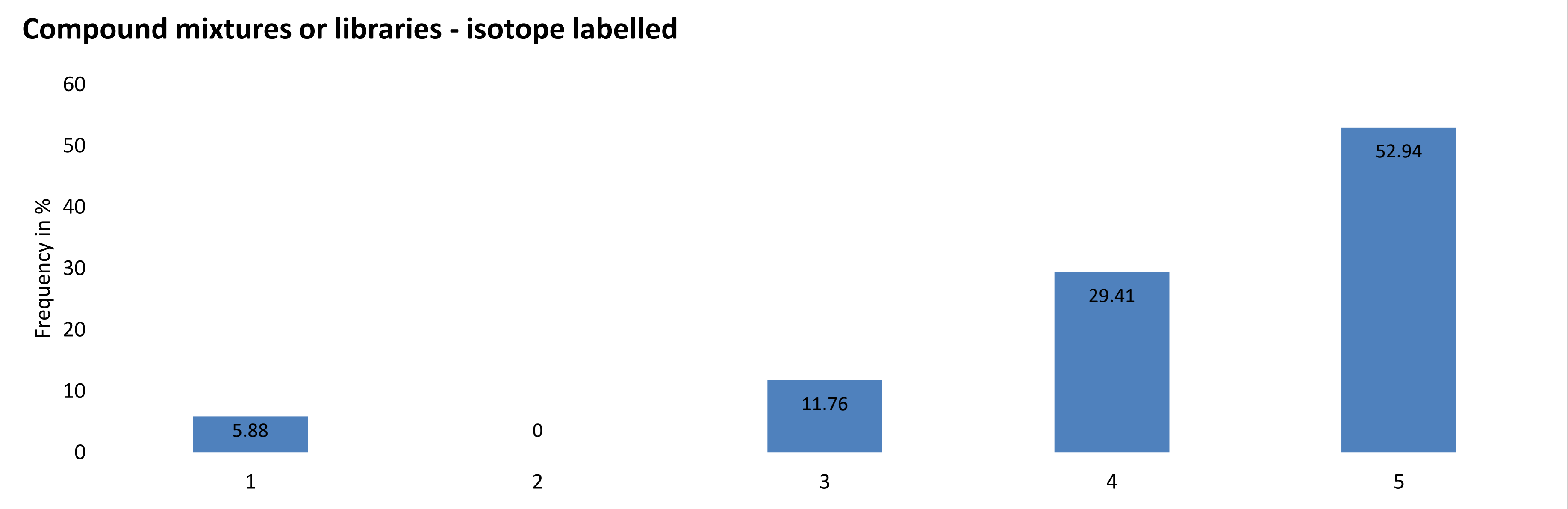

Question 33 - Is cost prohibiting you from using chemical standards or reference materials in your metabolomics workflow?

Status: July 18, 2024, 13:37, Survey: "DGMet-Survey"

Number of participants evaluated: 29 (all participants)

Detailed results for Reference material (including research grade testing materials)

Variable	V196	Number of answers	14
Average	3.50	Median	3.50
Variance	0.82	Standard deviation	0.91
Smallest Value	2	Highest Value	5

Rate from 1 = cost is no issue to 5 = cost is problematic

Value/Answer	Number	Frequency
1	0	0%
2	2	11.76%
3	5	29.41%
4	5	29.41%
5	2	11.76%
**Total**	**14**	**48.28%**

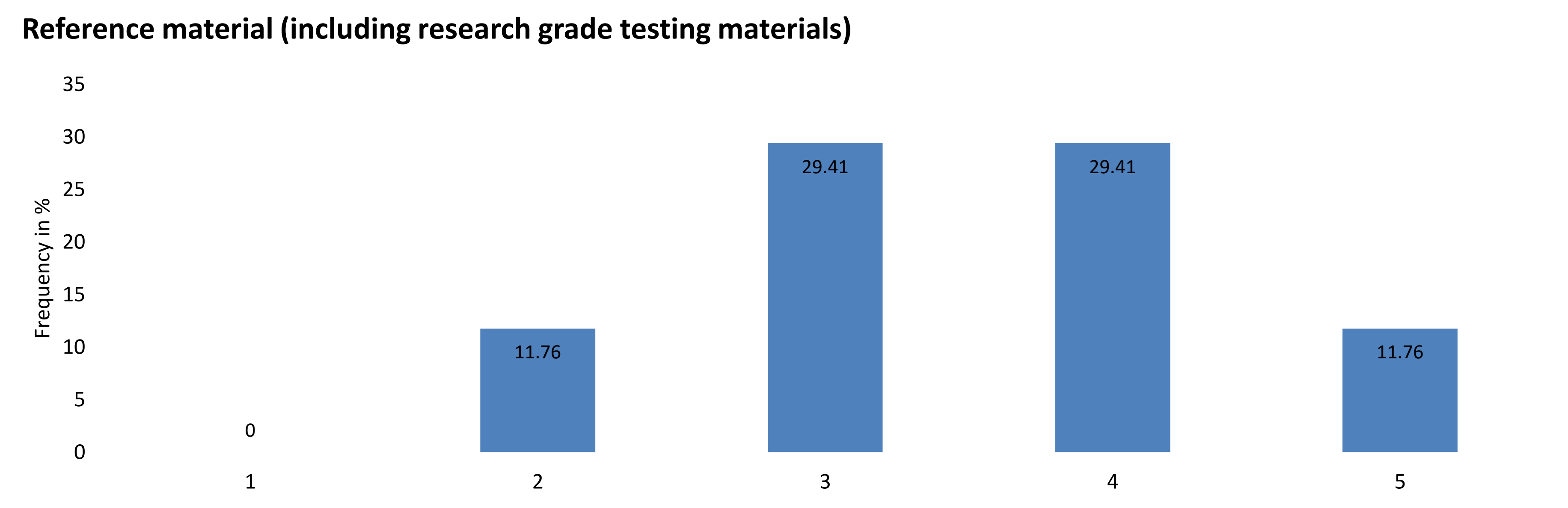

Question 33 - Is cost prohibiting you from using chemical standards or reference materials in your metabolomics workflow?

Status: July 18, 2024, 13:37, Survey: "DGMet-Survey"

Number of participants evaluated: 29 (all participants)

Detailed results for Certified reference material (CRM)

Variable	V197	Number of answers	12
Average	3.42	Median	4
Variance	1.91	Standard deviation	1.38
Smallest Value	1	Highest Value	5

Value/Answer	Number	Frequency
1	2	11.76%
2	1	5.88%
3	2	11.76%
4	4	23.53%
5	3	17.65%
**Total**	**12**	**41.38%**

Rate from 1 = cost is no issue to 5 = cost is problematic

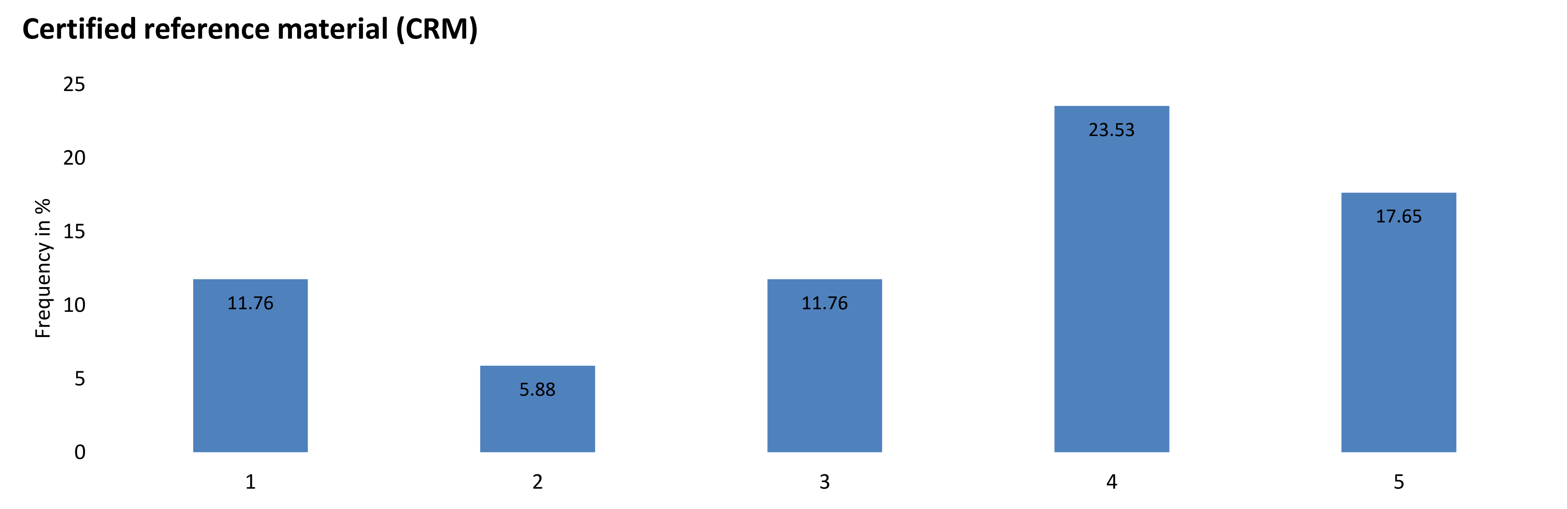

Created with LamaPoll | https://www.lamapoll.de

Question 34 - Are there particular standard mixtures or reference materials that you are interested in?

Status: July 18, 2024, 13:37, Survey: "DGMet-Survey"

Number of participants evaluated: 29 (all participants)

Status data

of 29 participants	Number	Percent
Question seen	22	75.86%
Question answered	7	24.14%
Question not answered	22	75.86%

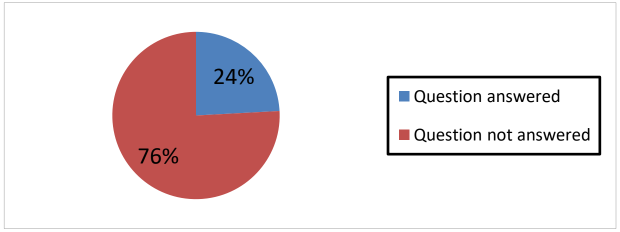

Detailed results for (1)

Number Answers	7	Number unique	7

Value/Answer	Number	Frequency
Bacterial metabolites and lipids	1	14.29%
Defined libraries of metabolite mixtures - all primary metabolites	1	14.29%
Fecal standard	1	14.29%
Gut metabolites in general (microbiome)	1	14.29%
Pooled organ specific plant materials	1	14.29%
Primary metabolite mix	1	14.29%
U13c labeled extract of something that is more similar to mammalians than yeast is.	1	14.29%
**Total**	**7**	**100%**

Question 34 - Are there particular standard mixtures or reference materials that you are interested in?

Status: July 18, 2024, 13:37, Survey: "DGMet-Survey"

Number of participants evaluated: 29 (all participants)

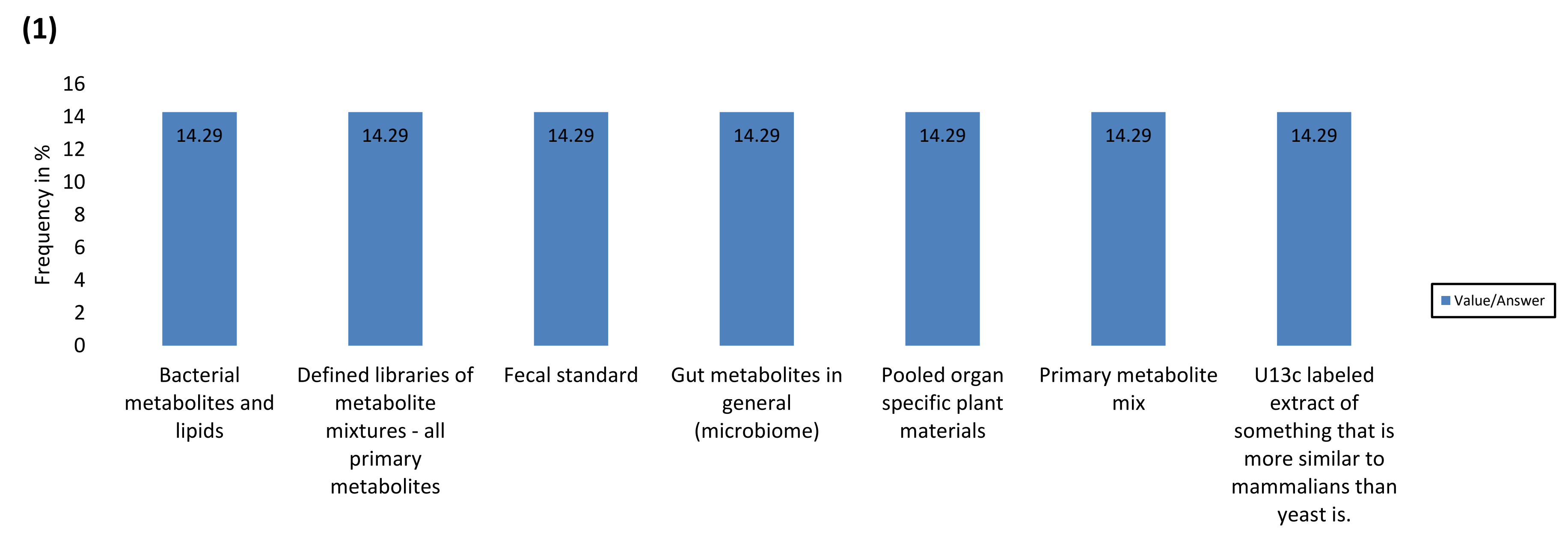

Question 34 - Are there particular standard mixtures or reference materials that you are interested in?

Status: July 18, 2024, 13:37, Survey: "DGMet-Survey"

Number of participants evaluated: 29 (all participants)

Detailed results for (2)

Number Answers	5	Number unique	5

Value/Answer	Number	Frequency
Alternative to srm1950 - less expensive	1	20%
International blood serum standard	1	20%
Pathway specific mixtures (e.g. tryptophan degradation)	1	20%
Pooled photosynthetic microbe specific materials	1	20%
Standard mixture for system suitability tests	1	20%
**Total**	**5**	**100%**

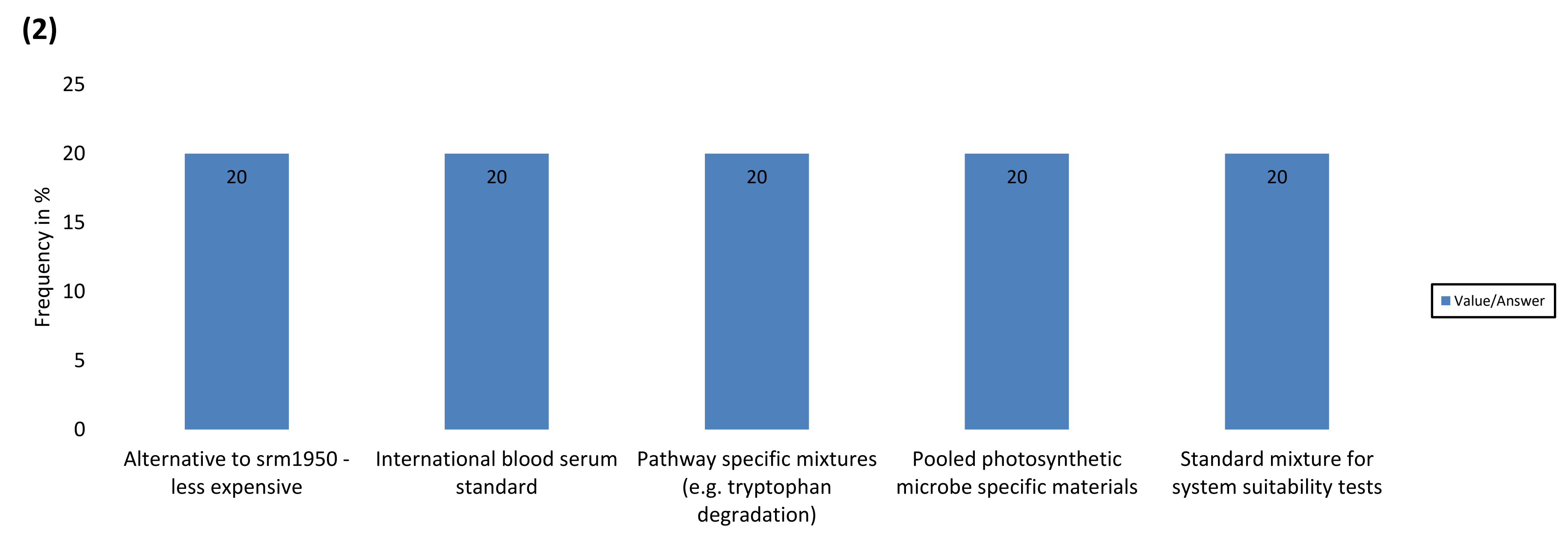

Question 34 - Are there particular standard mixtures or reference materials that you are interested in?

Status: July 18, 2024, 13:37, Survey: "DGMet-Survey"

Number of participants evaluated: 29 (all participants)

Detailed results for (3)

Number Answers	3	Number unique	3

Value/Answer	Number	Frequency
Many lipid species	1	33.33%
More comprehensive lipidomics mixtures	1	33.33%
Mouse serum/plasma	1	33.33%
**Total**	**3**	**100%**

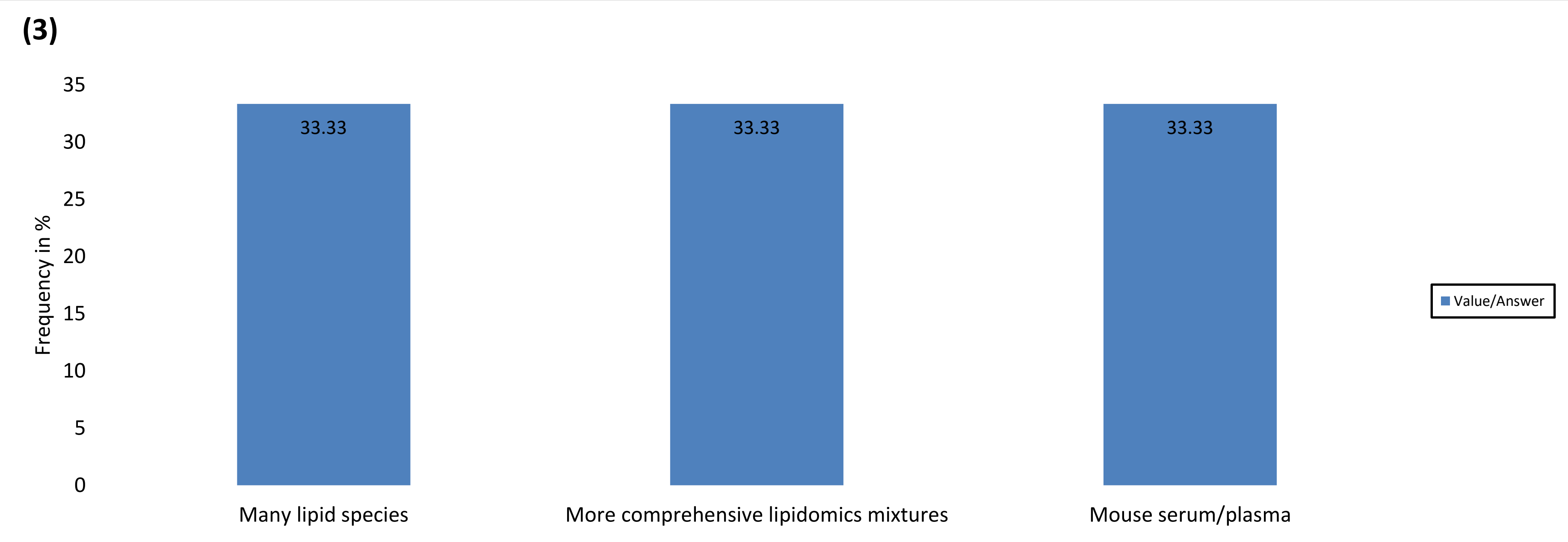

Question 34 - Are there particular standard mixtures or reference materials that you are interested in?

Status: July 18, 2024, 13:37, Survey: "DGMet-Survey"

Number of participants evaluated: 29 (all participants)

Detailed results for (4)

Number Answers	2	Number unique	2

Value/Answer	Number	Frequency
Bacterial secondary metabolites mixture	1	50%
Depleted/stripped plasma for method validation and blanks	1	50%
Total	2	100%

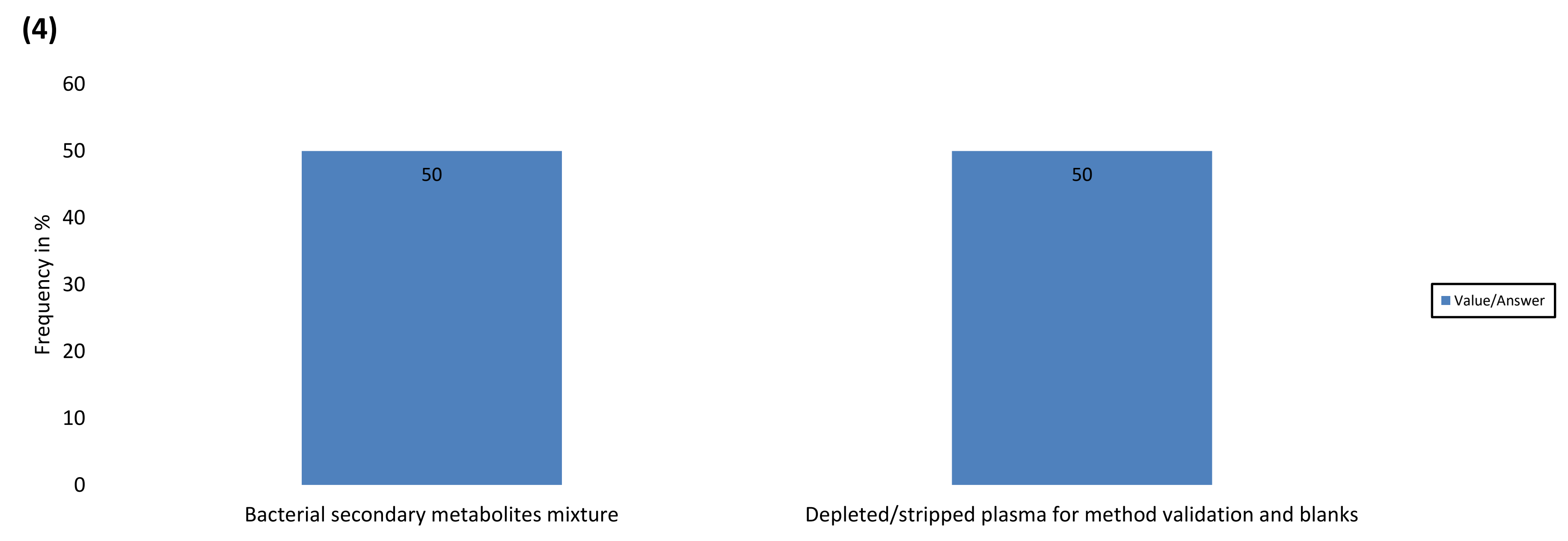

Question 34 - Are there particular standard mixtures or reference materials that you are interested in?

Status: July 18, 2024, 13:37, Survey: "DGMet-Survey"

Number of participants evaluated: 29 (all participants)

Detailed results for (5)

Number Answers	1	Number unique	1

Value/Answer	Number	Frequency
Artificial matrices	1	100%
**Total**	**1**	**100%**

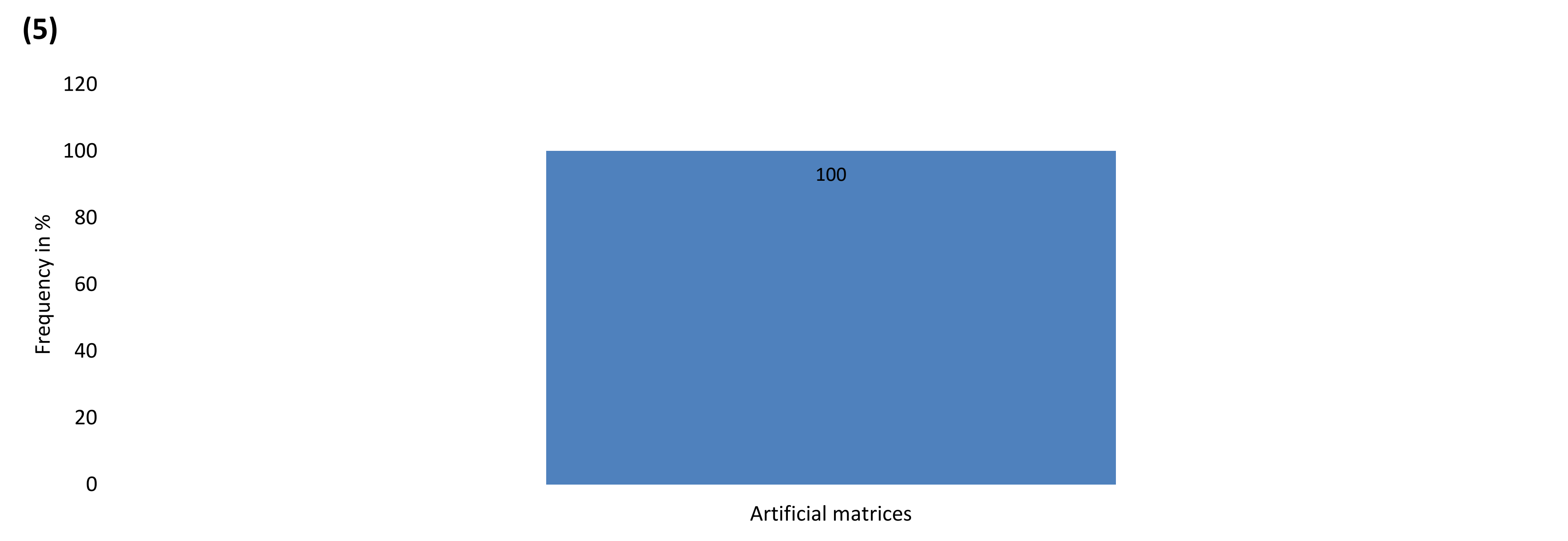

Question 35 - Ring Trial Participation

Status: July 18, 2024, 13:37, Survey: "DGMet-Survey"

Number of participants evaluated: 29 (all participants)

Status data

of 29 participants	Number	Percent
Question seen	21	72.41%
Question answered	20	68.97%
Question not answered	9	31.03%

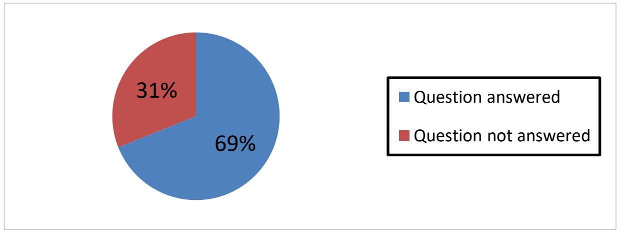

Results

Options	Variable	Code	Number	Frequency by participant	Frequency by answers
Yes	V192	1	12	60%	57.14%
No	V193	1	1	5%	4.76%
Don't know yet	V194	1	8	40%	38.10%
**Total**			21 Answers	20 Participants	

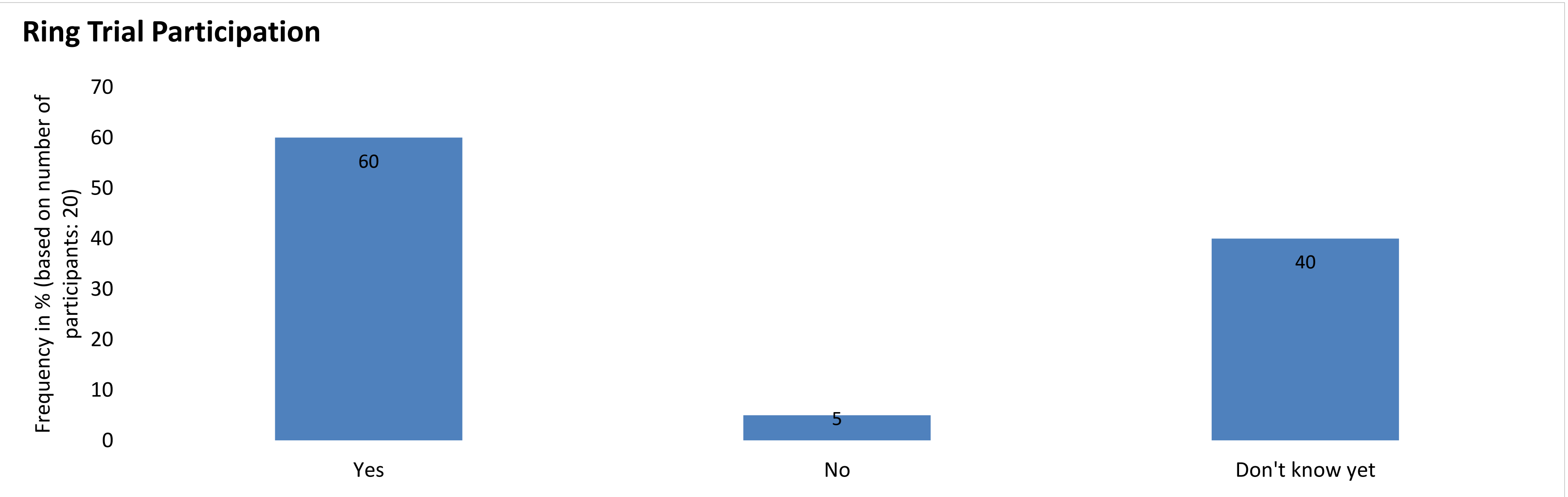

Question 36 - Motivation

Status: July 18, 2024, 13:37, Survey: "DGMet-Survey"

Number of participants evaluated: 29 (all participants)

Status data

of 29 participants	Number	Percent
Question seen	12	41.38%
Question answered	11	37.93%
Question not answered	18	62.07%

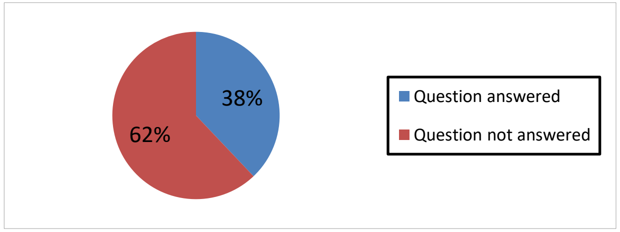

Detailed results for (1)

Number Answers	11	Number unique	11

Value/Answer	Number	Frequency
Benchmarking	1	9.09%
Comparing of our method with other ones	1	9.09%
Comparison of method performance	1	9.09%
Evaluate performance of own machines and methods	1	9.09%
Evaluating the performance of own analytical methods	1	9.09%
Evaluating the performance of own methods	1	9.09%
Evaluation of the performance of own developed methods	1	9.09%
Harmonizations of methods to yield comparable results	1	9.09%
Improving quality	1	9.09%
Learn how/where to improve my own methods	1	9.09%
Test reproducibility and applicability of our analytical data	1	9.09%
**Total**	**11**	**100%**

Question 36 - Motivation

Status: July 18, 2024, 13:37, Survey: "DGMet-Survey"

Number of participants evaluated: 29 (all participants)

(1)

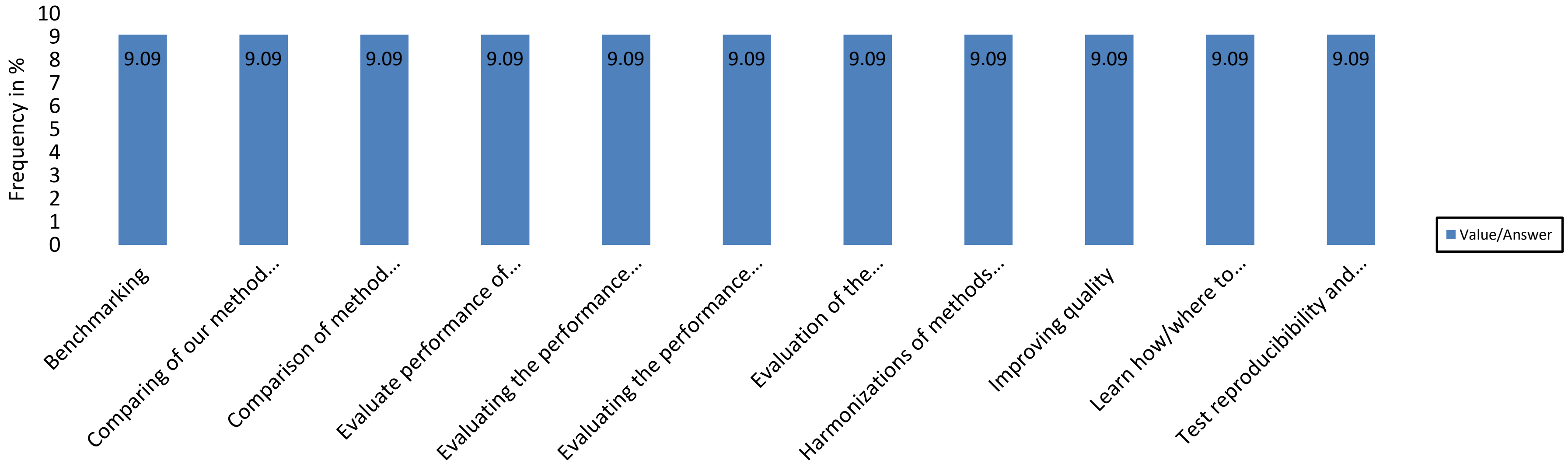

Question 36 - Motivation

Status: July 18, 2024, 13:37, Survey: "DGMet-Survey"

Number of participants evaluated: 29 (all participants)

Detailed results for (2)

Number Answers	10	Number unique	10

Value/Answer	Number	Frequency
Contibution to standardization and harmonization	1	10%
Contributing to harmonization	1	10%
Determine cross lab variation	1	10%
Establishing new cooperations	1	10%
Evaluation of own methods	1	10%
Exchange about methods	1	10%
Improve own workflows	1	10%
Improving inter-laboratory comparability of data	1	10%
Networking	1	10%
Require better interlaboratory comparability	1	10%
**Total**	**10**	**100%**

Question 36 - Motivation

Status: July 18, 2024, 13:37, Survey: "DGMet-Survey"

Number of participants evaluated: 29 (all participants)

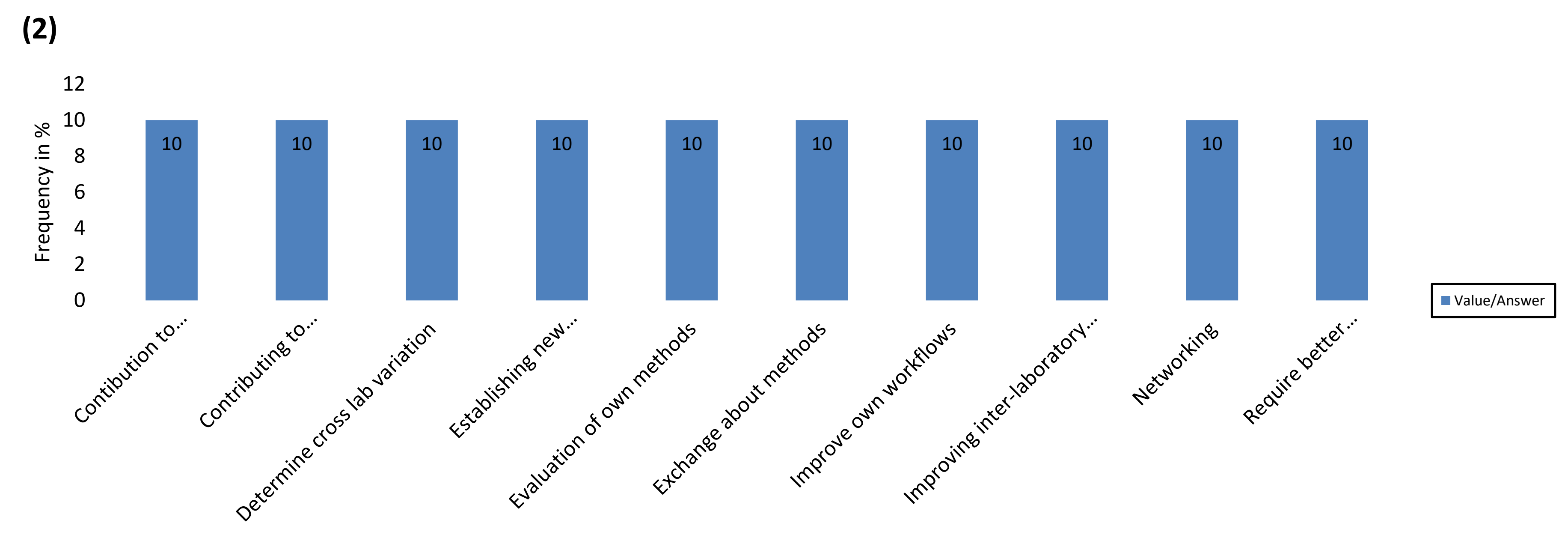

Question 36 - Motivation

Status: July 18, 2024, 13:37, Survey: "DGMet-Survey"

Number of participants evaluated: 29 (all participants)

Detailed results for (3)

Number Answers	6	Number unique	6

Value/Answer	Number	Frequency
Contributing to acceptance of metabolomics in a regulatory context	1	16.67%
Contribution to harmonization	1	16.67%
Get new ideas for new methods	1	16.67%
Hope for more harmonized chromatography methods in the field (maybe naive)	1	16.67%
Networking	1	16.67%
Networking within the communities	1	16.67%
**Total**	**6**	**100%**

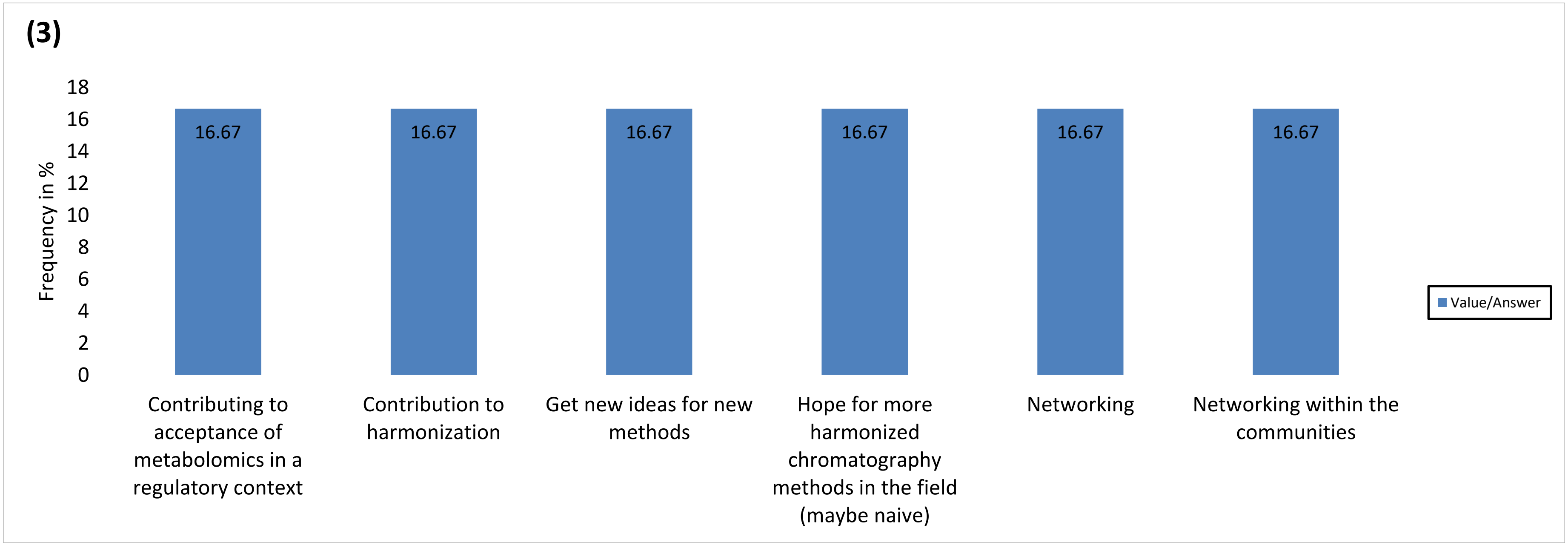

Question 37 - Did you participate in ring trials in the past?

Status: July 18, 2024, 13:37, Survey: "DGMet-Survey"

Number of participants evaluated: 29 (all participants)

Status data

of 29 participants	Number	Percent
Question seen	21	72.41%
Question answered	20	68.97%
Question not answered	9	31.03%

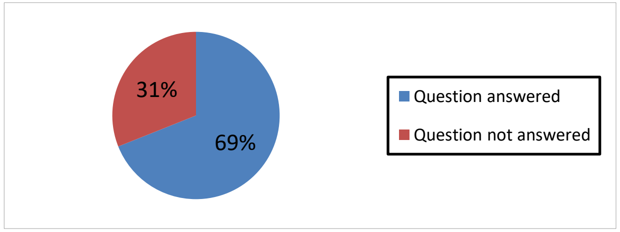

Results

Options	Variable	Code	Number	Frequency
Yes	V198	1	9	45%
No	V198	2	11	55%
**Total**			20 Answers	20 Participants

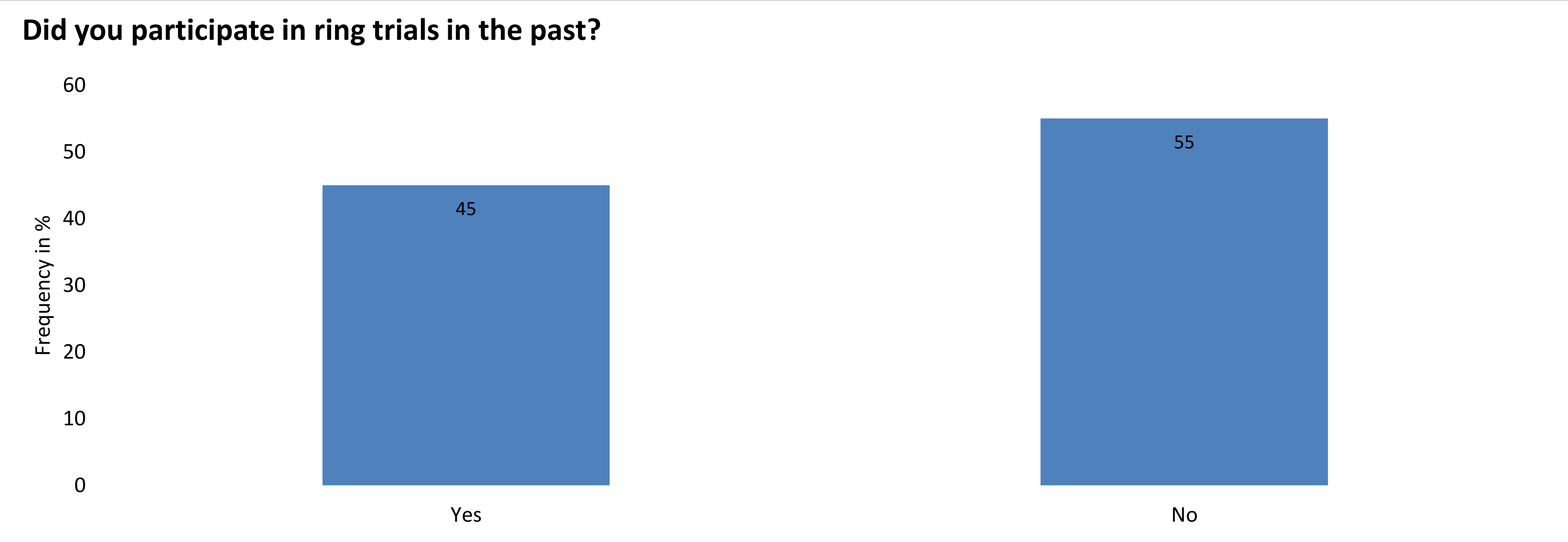

Question 38 - Workshop

Status: July 18, 2024, 13:37, Survey: "DGMet-Survey"

Number of participants evaluated: 29 (all participants)

Status data

of 29 participants	Number	Percent
Question seen	21	72.41%
Question answered	20	68.97%
Question not answered	9	31.03%

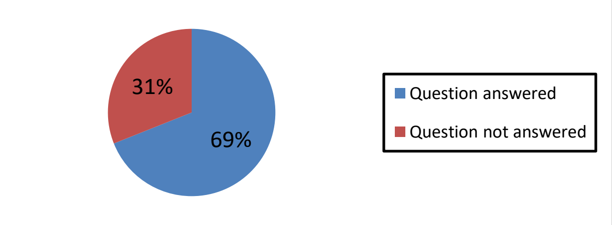

Results

Options	Variable	Code	Number	Frequency
Yes	V202	1	15	75%
No	V202	2	5	25%
**Total**			20 Answers	20 Participants

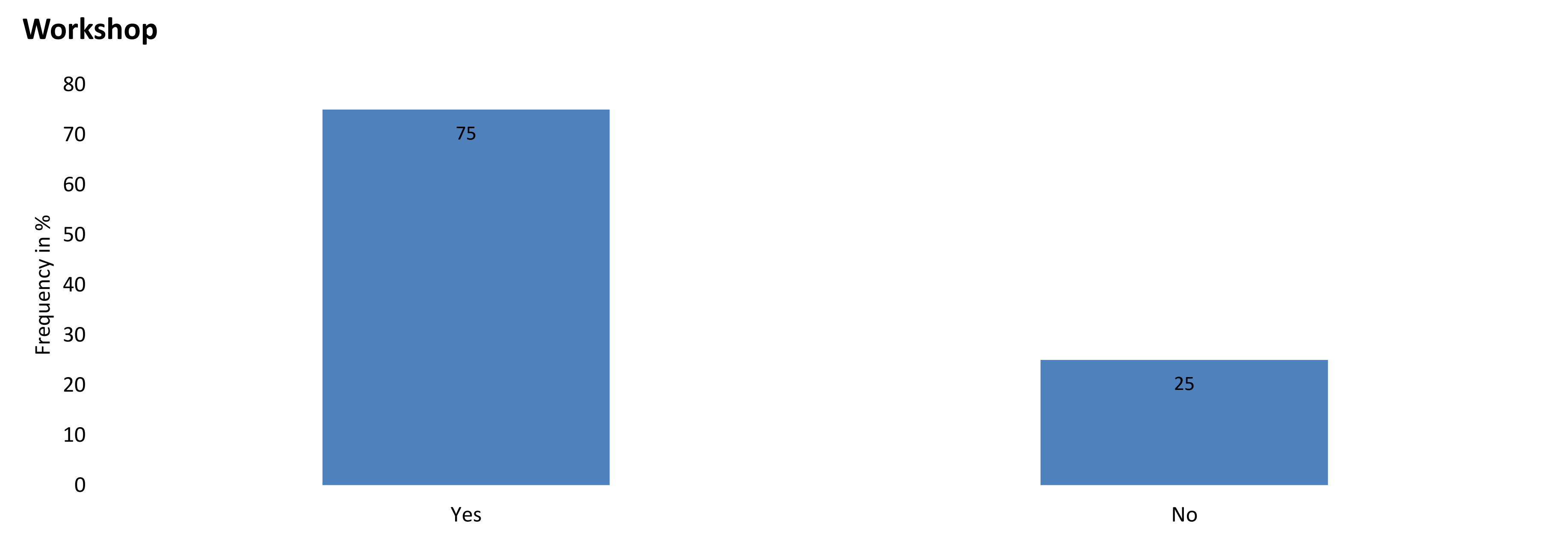

Question 40 - Position of the Contact Person

Status: July 18, 2024, 13:37, Survey: "DGMet-Survey"

Number of participants evaluated: 29 (all participants)

Status data

of 29 participants	Number	Percent
Question seen	21	72.41%
Question answered	18	62.07%
Question not answered	11	37.93%

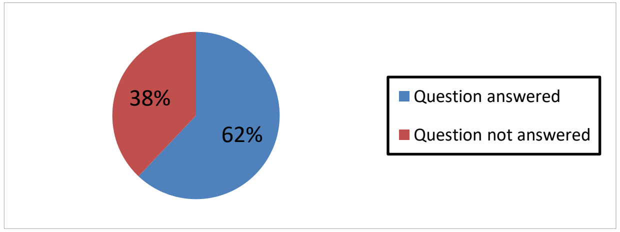

Results

Options	Variable	Code	Number	Frequency
Group leader	V49	1	10	55.56%
Project leader	V49	2	1	5.56%
(Senior) Scientist	V49	3	6	33.33%
Technician	V49	4	0	0%
Postdoc	V49	5	1	5.56%
Doctoral Student	V49	6	0	0%
Student	V49	7	0	0%
Other	V49	8	0	0%
**Total**			18 Answers	18 Participants

Question 40 - Position of the Contact Person

Status: July 18, 2024, 13:37, Survey: "DGMet-Survey"

Number of participants evaluated: 29 (all participants)

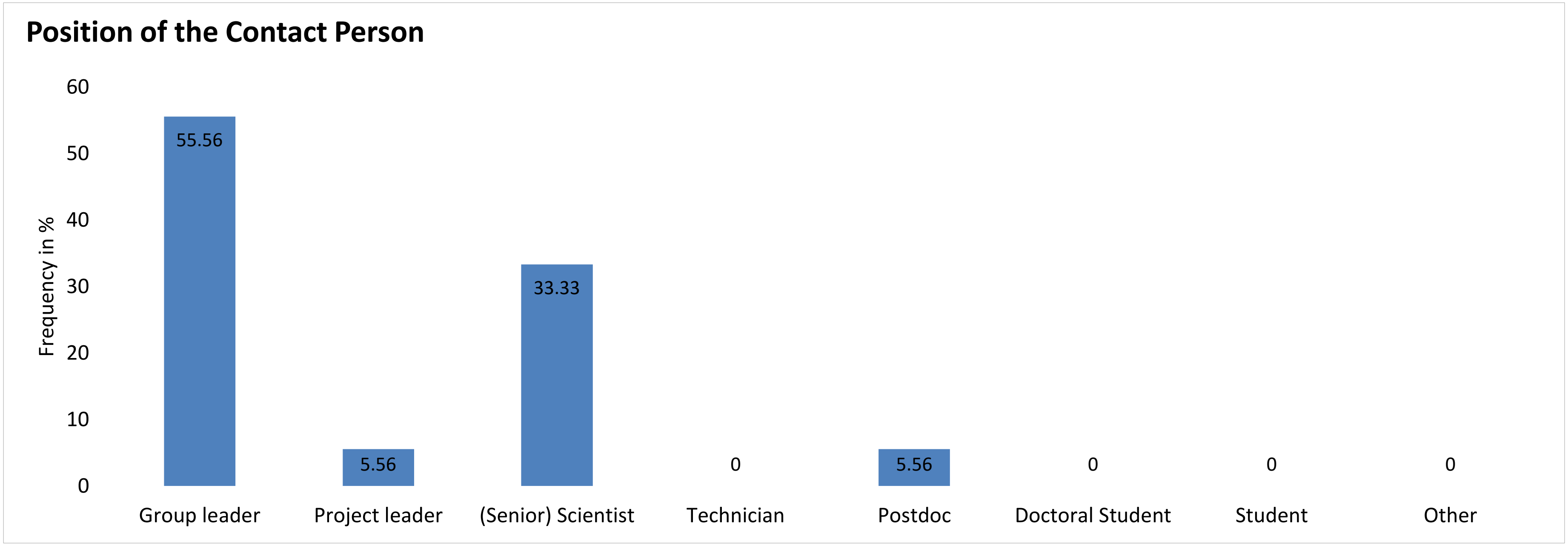

Supplement: Supplementary file 2 — Supplementary Material 2 [file 11306_2025_2360_MOESM2_ESM.pdf]
